# How do donor and acceptor substituents change the photophysical and photochemical behavior of dithienylethenes? The search for a water-soluble visible-light photoswitch†

**DOI:** 10.1039/d3sc01458d

**Published:** 2023-07-31

**Authors:** Sili Qiu, Andrew T. Frawley, Kathryn G. Leslie, Harry L. Anderson

**Affiliations:** a Department of Chemistry, Chemistry Research Laboratory, University of Oxford Oxford OX1 3TA UK andrew.frawley@chem.ox.ac.uk harry.anderson@chem.ox.ac.uk

## Abstract

Dithienylethenes are a type of diarylethene and they constitute one of the most widely studied classes of photoswitch, yet there have been no systematic studies of how electron-donor or -acceptor substituents affect their properties. Here we report eight dithienylethenes bearing push–push, pull–pull and push–pull substitution patterns with different lengths of conjugation in the backbone and investigate their photophysical and photochemical properties. Donor–acceptor interactions in the closed forms of push–pull dithienylethenes shift their absorption spectra into the near-infrared region (*λ*_max_ ≈ 800 nm). The push–pull systems also exhibit low quantum yields for photochemical electrocyclization, and computational studies indicate that this can be attributed to stabilization of the parallel, rather than anti-parallel, conformations. The pull–pull systems have the highest quantum yields for switching in both directions, ring-closure and ring-opening. The chloride salt of a pull–pull DTE, with alkynes on both arms, is the first water-soluble dithienylethene that can achieve >95% photostationary state distribution in both directions with visible light. It has excellent fatigue resistance: in aqueous solution on irradiation at 365 nm, the photochemical quantum yields for switching and decomposition are 0.15 and 2.6 × 10^−5^ respectively, *i.e.* decomposition is more than 5000 times slower than photoswitching. These properties make it a promising candidate for biological applications such as super-resolution microscopy and photopharmacology.

## Abstract 2

A systematic investigation of a family of photo-switches with electron-donating and -accepting substituents reveals that the switch with two electron-accepting 4-ethynyl-pyridinium groups exhibits excellent switching behavior in aqueous solution.

## Graphical abstract

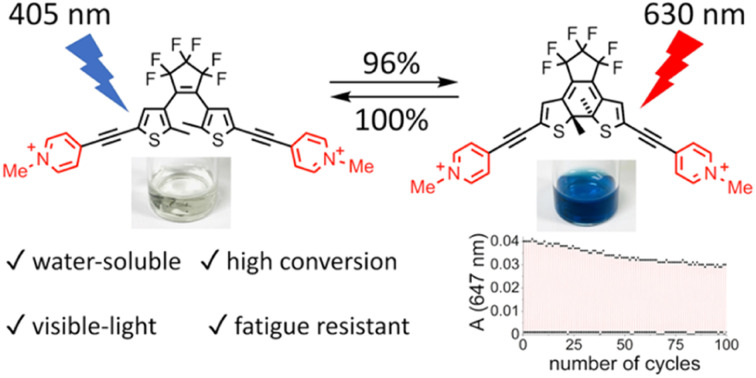

## Introduction

Photoswitches are compounds that can be reversibly interconverted between two isomers by light.^1–3^ The structural difference between the two photoisomers results in distinctive physical and chemical properties such as UV-vis absorption,^4^ fluorescence,^5–7^ conductance,^8–10^ end-to-end distance and rigidity,^11–13^ and chemical reactivity,^14,15^ leading to diverse applications in optical data storage,^16,17^ photopharmacology,^18,19^ super-resolution microscopy,^20–23^ and sensing of species such as mercury and cysteine.^24,25^ These various applications require photoswitches with optimized properties, such as high quantum yields of photoswitching, high conversion between isomers and good fatigue resistance. Photoswitches for use in biological applications should be switchable with visible light, rather than UV light, to avoid phototoxicity and to ensure compatibility with microscope optics.

The photoisomerization of molecular switches is commonly achieved by *E*/*Z* isomerization about a double bond (*e.g.* stilbenes, azobenzenes, thioindigos, hemithioindigos), photo-induced electrocyclizations (diarylethenes), or a mixed mechanism (spiropyrans, fulgides, fulgimides *etc.*).^26,27^

**Scheme 1:**
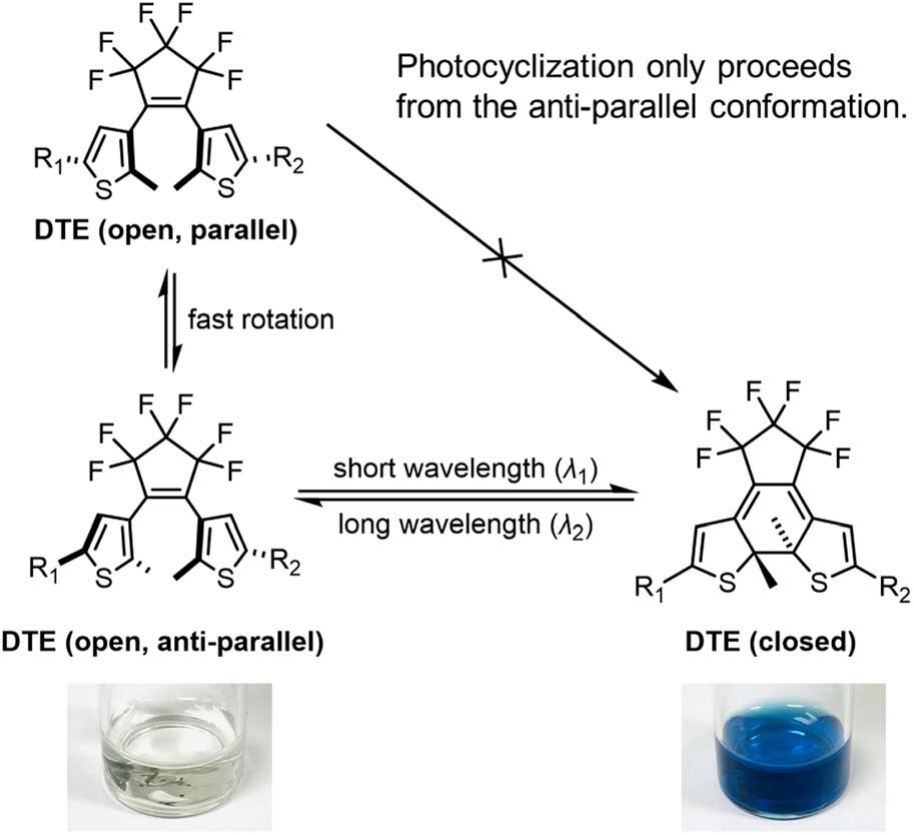
The photoisomerization of a dithienylethene from its anti-parallel conformer and the associated color change.

Dithienylethenes (DTEs), a sub-class of diarylethenes, consisting of two thiophenes connected *via* a cyclopentene bridge, were pioneered by Irie and co-workers^28–32^ and they are now among the most widely used photochromic dyes with some of the best properties of all classes of photoswitches (Scheme 1). Although hundreds of DTEs have been synthesized,^33–37^ there are few systematic structure–property studies of their photophysical and photochemical properties.^38,39^ Moreover, most reports focus on symmetrical DTEs (R_1_ = R_2_ in Scheme 1). To the best of our knowledge, only a few push–pull DTEs bearing an electron donor on one arm (R_1_) and an electron acceptor on the other (R_2_) have been explored^8,9,35,40–42^ and there is little information on the impact of electronic substitution patterns on the photophysical and photochemical properties of DTEs. In this study, we have systematically investigated the electronic effect of different substituents on the photoswitching properties in a family of DTEs, in order to optimize these photoswitches for potential biological applications.

Organic π-systems end-capped with an electron donor (D) and an electron acceptor (A) are intriguing because the D–A interaction, or intramolecular charge transfer (ICT), reduces the HOMO–LUMO energy gap, leading to lower-energy π–π* transitions.^43^ Incorporating a photoswitchable π-system between the donor and acceptor would allow control of the ICT interaction. In push–pull DTEs, ICT from one arm to another can only occur in the closed form, because the donor–acceptor pathway is cross-conjugated in the open form, but fully conjugated in the closed form. The extent of ICT controls the wavelength of absorption, making it possible to shift the absorption band of the closed form, while leaving the absorption of the open form unaffected, potentially leading to better control of the optimal wavelengths for switching in each direction.

DTEs undergo reversible photoinduced electrocyclization at the central hexatriene unit (Scheme 1). There are several structural features that affect the efficiency of this transformation, including the nature of the bridge between the thiophenes, the substituent at the beta-thiophene position, and the nature of the arms. In previous work, DTEs with central perfluoro-cyclopentene bridges and methyl-substituted thiophenes or benzothiophenes have been shown to be more fatigue resistant.^44^ For this reason, we have chosen to keep these components constant across the whole family of molecules in this study, and focus our efforts on varying the arms (R_1_ and R_2_ in Scheme 1).

DTEs are flexible in their open form due to free rotation about the thiophene–pentene bond, but rigidify upon switching to the closed form. Conjugation throughout the whole molecule is only established in the closed form. The difference in conjugation results in a large separation of the major π–π* transitions, with the open form typically absorbing in the UV (*λ*_max_ 300–400 nm), while the closed form absorbs in the red or near infrared (*λ*_max_ 600–800 nm). DTEs cyclize photochemically in a conrotatory fashion, in agreement with the Woodward–Hoffmann rules.^45^ The open-form DTE can be in a parallel or anti-parallel conformation depending on the relative orientation of the two thiophene arms (Scheme 1). DTEs in the parallel conformation cannot photocyclize due to the steric hindrance when the substituted arms clash into the DTE core. DTEs in which the anti-parallel conformation of the open form is favored by electrostatic or steric interactions can have exceptionally high quantum yields of photocyclization.^46^

**Fig. 1:**
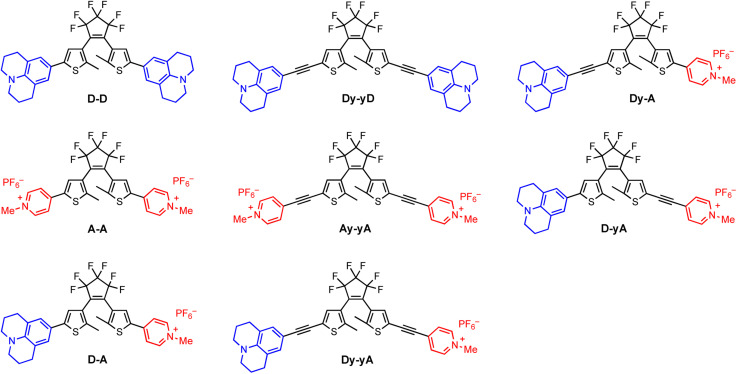
Structures of the open-form DTEs synthesized, bearing donor–donor, acceptor–acceptor or donor–acceptor substitution patterns. Electron donor and acceptor are julolidine (blue) and *N*-methylpyridinium (red), respectively. D, A, y and – represent the donor, the acceptor, the alkyne, and the central DTE core, respectively.

We selected julolidine (blue, Fig. 1) to be a representative electron donor because the lone pair of electrons on the nitrogen is delocalized into the aromatic ring to a larger extent compared to other aniline derivatives. Charged *N*-methylpyridinium (red, Fig. 1) was selected as the electron acceptor. This ionic functional group has the added benefit of increasing the solubility of DTEs in polar solvents, facilitating biologically-relevant applications. Previous work by Irie and co-workers^46^ has shown that A–A is the DTE with the highest photocyclization quantum yield reported to date, due to the coulombic repulsion between the two cationic moieties, which forces the diarylethene to adopt a photoactive anti-parallel conformation. The monocationic analogue with a *N*-methylpyridinium group on one arm and a phenyl group on the other arm has a moderate photochemical quantum yield, three-times smaller than A–A.

## Key properties of photoswitches

Photoswitching can be quantified by several parameters. Prolonged irradiation at a specific wavelength (*λ*) leads to a dynamic equilibrium between the two photoisomers, known as the photostationary state (PSS). The composition of the PSS is commonly expressed as the mole fraction of one photoisomer, which is the photostationary distribution (PSD_*λ*_, eqn (1)).^27^ The photochemical quantum yield (*Φ*) quantifies the number of molecules which undergo a photochemical reaction per photon absorbed.^47^ Experimentally, a combination of high molar absorption coefficient, *ε*, and a high photochemical quantum yield leads to a fast photoreaction. The photodynamic equilibrium constant, *K*, is given by eqn (2), where *k*_o–c_ and *k*_c–o_ are the effective first-order rate constants of the photochemical ring closing and opening reactions, *ε*_open,*λ*_ and *ε*_closed,*λ*_ are the molar absorption coefficients of the two isomers at the excitation wavelength, and *Φ*_o–c_ and *Φ*_c–o_ are the photochemical quantum yields for the cyclization and ring-opening reactions. Combining eqn (1) and (2) gives eqn (3).^48^1
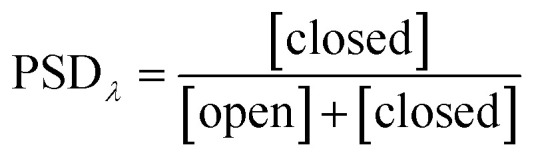
2
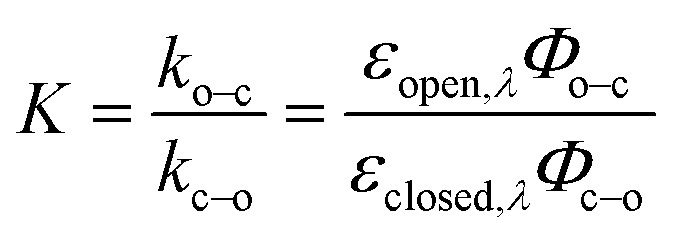
3
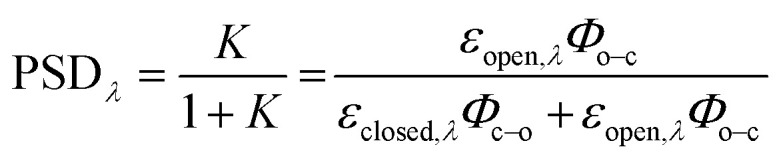


Reversible photoswitching of any compound can be divided into two extreme scenarios. If there is a good separation between absorption bands, one isomer of the compound can be irradiated at a wavelength where the other isomer does not absorb, leading to a clean conversion from one to the other (eqn (3); PSD_*λ*_ = 1 or 0). Alternatively, if there is poor band separation, forward and reverse reactions take place simultaneously under light irradiation. Assuming the same absorption coefficient for the two isomers at the wavelength of irradiation, a quantitative (>99%) PSD can only be achieved if the quantum yield of forward reaction is at least 100 times greater than for the reverse reaction. In this work, we measured these parameters to elucidate how donor and acceptor substituents change the photophysical and photochemical behavior of DTEs.

## Results and discussion

### Design and synthesis of photoswitches

**Scheme 2:**
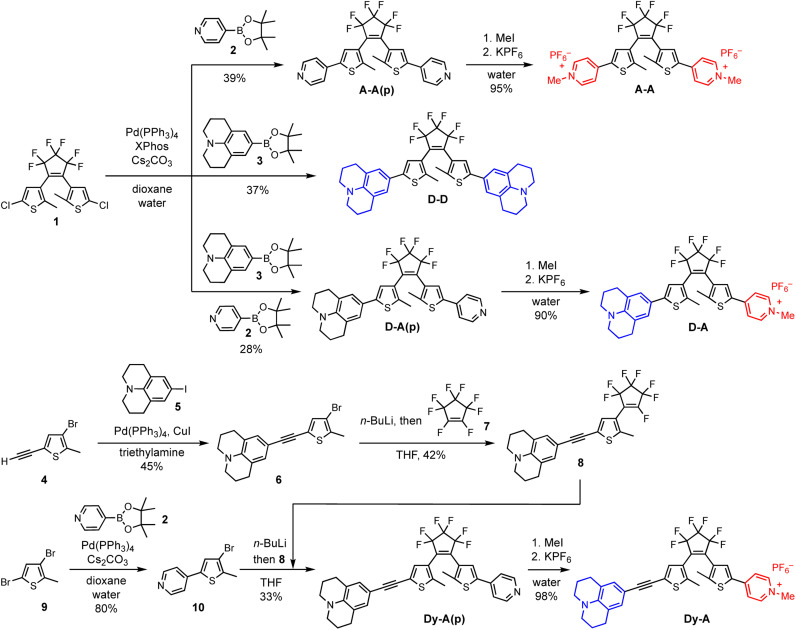
(Top) Synthesis of D–D, A–A and D–A starting with the construction of DTE dichloride core 1 followed by functionalization of the arm by Suzuki coupling, methylation and ion exchange. D–A(p) can be synthesized by Suzuki coupling with two different boronate esters in one pot and then separating the three compounds by column chromatography. (Bottom) Synthetic route to Dy–A starting with functionalization of the two arms followed by the construction of DTE core at a later stage.

The synthesis of DTEs generally involves two key steps: formation of the DTE hexatriene core and attachment of the arms. The order of these two steps is not critical, provided that the functional groups on the thiophene arm are chemically compatible with the process of forming the DTE core (which typically requires *n*-BuLi). Here we take the synthesis of D–D, A–A and D–A as examples of forming the DTE core at an early stage and the synthesis of Dy–A as an example of forming the DTE core at a later stage (Scheme 2). Both pathways permit straightforward synthesis of DTEs with functionalized arms.

Symmetric D–D, A–A and asymmetric D–A were synthesized starting from the DTE dichloride 1. This key intermediate was synthesized following an established procedure,^49^ which involves lithiation of 4-bromo-2-chloro-5-methylthiophene followed by an addition–elimination with octafluorocyclopentene (Scheme S1†). The DTE dichloride 1 can be further functionalized with aryl groups by Suzuki coupling (Scheme S2†).^49^ In the syntheses of D–D and A–A(p), julolidine-Bpin 3 and 4-pyridyl-Bpin 2 were used as the boronate ester partners, respectively. Synthesizing D–A(p) was more challenging because the two chlorides in 1 are equivalent, making it difficult to create the required asymmetry. Instead of attaching the two different functional groups in a stepwise manner, we performed the Suzuki coupling reaction with two different boronate ester partners in one pot, which led to the formation of D–D, A–A(p) and D–A(p). The three compounds were easily separated by column chromatography on silica. Finally, the pyridine groups were methylated with iodomethane and the counterion was changed from iodide to hexafluorophosphate to increase the solubility in organic solvents. Despite having reduced atom efficiency, one-pot Suzuki coupling allowed easy access the DTE substituted with two different functional groups.

The DTEs with additional alkynes on both sides (Dy–yD, Ay–yA and Dy–yA) were synthesized *via* a similar route starting with the formation of DTE core (Schemes S3 and S4†). The TMS-protected alkynes were coupled to the thiophene before forming the DTE core. Once the photoswitchable hexatriene unit was formed, the TMS protecting groups were removed so that Sonogashira coupling reactions could be used to further extend the arms of the DTE.

Dy–A and D–yA could only be synthesized by constructing the arms first and then forming the DTE core at a later stage (Schemes 2, S5 and S6†). The donor arm 6 and acceptor arm 10 were constructed by Sonogashira coupling or Suzuki coupling, respectively, before forming the DTE core. The donor arm was attached to the perfluorocyclopentene first to form compound 8. The acceptor arm was lithiated and then reacted with 8 at the remaining site susceptible to nucleophilic attack to afford Dy–A(p). Finally, the pyridine group was methylated and the counterion was changed from iodide to hexafluorophosphate.

### Photochromism

The photophysical and photochemical properties of each DTE were analyzed in two solvents of different polarity. Neutral push–push D–D and Dy–yD and monocationic push–pull D–A, Dy–yA, Dy–A and D–yA were analyzed in THF as the less polar solvent and in MeCN as the more polar solvent. The photophysical properties of dicationic A–A and Ay–yA were evaluated in MeCN but not in THF due to poor solubility. When the counterions of A–A and Ay–yA are exchanged from hexafluorophosphate to chloride (Scheme S7†), both compounds become soluble in water. Therefore, their properties were also evaluated in water as the more polar solvent.

**Fig. 2:**
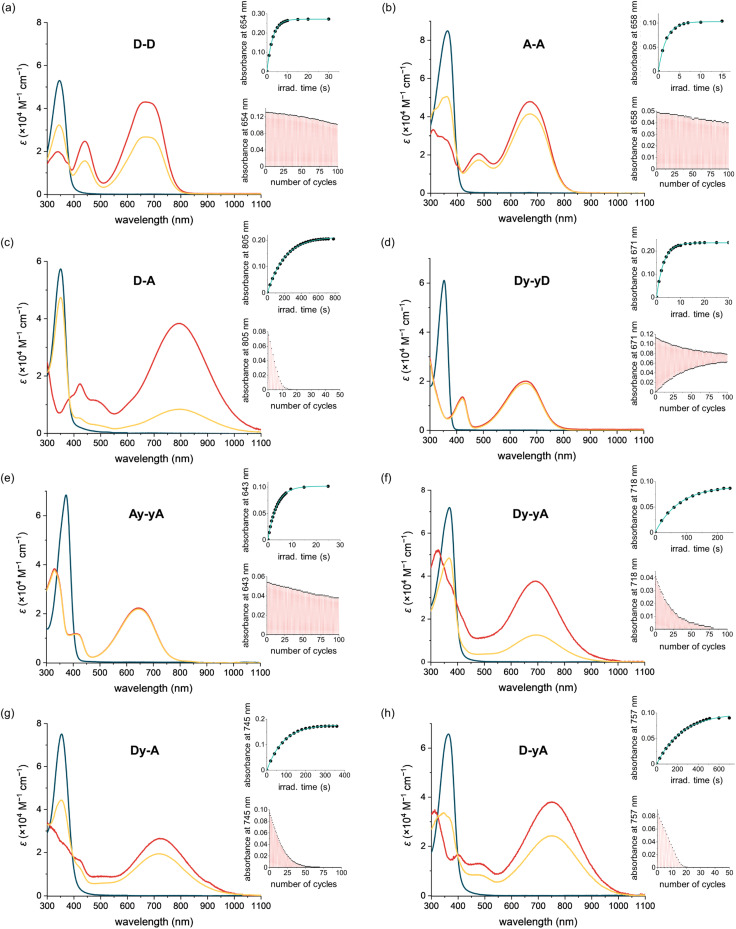
UV-vis-NIR absorption spectra of DTEs in acetonitrile. The blue, yellow and red lines indicate the experimental absorption spectra of the open form, at PSD (365 nm), and the calculated spectra of the closed form, respectively. The top-right inset shows the kinetic trace for photocyclization (365 nm). The lower-right inset shows the fatigue resistance. The kinetic trace and fatigue curve of D–D, D–A, Dy–yD, Dy–yA, Dy–A, and D–yA were measured in THF and those of A–A and Ay–yA were measured in MeCN. The power setting of 365 nm light irradiation is 18.9 mW for D–D and Dy–yD, 12.3 mW for A–A and Ay–yA, and 159 mW for D–A, Dy–yA, Dy–A and D–yA.

Initial assessment of the photochromism of the DTEs was performed by UV-vis spectroscopy. UV-vis spectra of the compounds in their open forms were recorded in two solvents before irradiation with 365 nm light (Fig. 2 and S3†). All compounds exhibit strong absorption bands around 360 nm which gradually disappear upon UV light irradiation. In all cases, irradiation at 365 nm leads to the emergence of broad absorption bands in the visible to near-IR regions, consistent with the flatter structure of the closed DTEs having extended conjugation. The switching causes a color change from colorless to green/blue, except for D–A, which changes to pale yellow. Switching most of these DTEs in the two different solvents usually results in similar colors, indistinguishable by eye. However, for Dy–yA and D–yA, only very faint color is achieved in acetonitrile while a much darker color is achieved in THF despite similar absorption coefficients in these two solvents, indicative of a large difference in PSD with lower conversion in acetonitrile.

Irradiation at 630 nm was used to drive the reverse ring-opening reaction. UV-vis spectra showed that the absorption bands of all DTEs at >600 nm, which corresponds to the closed form, completely disappeared after irradiation, indicating 100% conversion to the open form.

### Structural impact on UV-vis absorption spectra

**Fig. 3:**
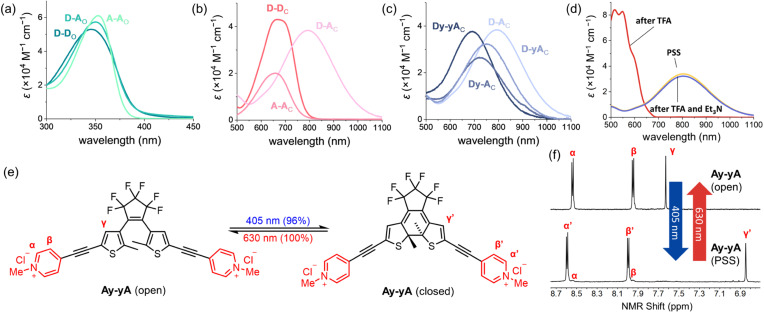
Absorption spectra of (a) open-form and (b) closed-form D–D, A–A and D–A in MeCN. (c) Absorption spectra of closed-form D–A, Dy–A, D–yA and Dy–yA in MeCN. (d) Absorption spectra of D–A at PSD in MeCN (yellow), MeCN + 0.5% TFA (red), and MeCN + 0.5% TFA + excess Et_3_N (blue). (e) Photoswitching process of the chloride salt of Ay–yA in water triggered by 405 nm light. (f) ^1^H NMR spectra showing the reversible switching of Ay–yA (chloride salt) in D_2_O between open-form and the PSS containing 96% closed-form.

The absorption spectra of all the compounds were recorded in MeCN, as summarized in Table 1, allowing comparison to deduce structure–property relationships between the absorption wavelengths and the substitution pattern, as well as the impact of additional alkynes in the conjugated backbone. As shown in Fig. 3a, the open isomers of all the DTEs absorb between 350 nm and 400 nm. The substitution pattern does not significantly affect the absorption spectra of the open forms of the DTEs, as D–D, A–A, and D–A absorb at 346 nm, 357 nm, and 350 nm, respectively. The additional alkyne units in Dy–yD, Ay–yA and Dy–yA increase the length of conjugation in the open form and lead to a 15–20 nm bathochromic shift of the absorption spectra compared to D–D, A–A and D–A. This red-shift caused by the presence of an additional alkyne on both arms significantly increases the absorption coefficient at 405 nm (from 271 M^−1^ cm^−1^ for A–A to 11 700 M^−1^ cm^−1^ for Ay–yA), allowing the compounds to be switched with 405 nm light to higher PSDs (96% for Ay–yA*vs.* 71% for A–A as shown in Table 1).

Summary of photophysical and photochemical properties of the DTEs

**Table:** 

	D–D		A–A		D–A		Dy–A	
Solvent^a^	THF	MeCN	MeCN	Water	THF	MeCN	THF	MeCN
*λ* ^O^_max_ b (nm)	346	346	353	357	352	350	356	354
*ε* (10^3^ M^−1^ cm^−1^)	52.4	53.0	61.2	61.5	57.4	57.2	76.1	74.2
*λ* ^C1^_max_ c (nm)	439	439	421	424	425	423	418	417
*ε* (10^3^ M^−1^ cm^−1^)	24.8	25.1	12.8	12.7	17.4	17.8	20.2	16.6
*λ* ^C2^_max_ d (nm)	654	667	658	659	805	792	745	724
*ε* (10^3^ M^−1^ cm^−1^)	42.7	43.1	19.1	19.0	38.2	38.1	29.4	26.5
PSD^e^ (%)	>99	69	>99	>99/71^f^	71	21	85	64
*Φ*_O–C_ (%)	7.7 ± 0.4	0.18 ± 0.01	31 ± 2	38 ± 3	0.020 ± 0.001	1.2 ± 0.2 × 10^−3^	0.059 ± 0.004	0.015 ± 0.001
*Φ*_C–O_ (%)	0.043 ± 0.003	0.039 ± 0.001	0.16 ± 0.01	0.16 ± 0.01	0.015 ± 0.002	0.014 ± 0.001	0.041 ± 0.002	0.043 ± 0.003

a Measured in acetonitrile or THF as PF_6_ salt and in water as chloride salt.

b Absorption maximum of the open-form DTE.

c First absorption maximum of the closed-form DTE between 400 and 500 nm.

d Second absorption maximum of the closed-form DTE at >600 nm.

e PSD achieved at 365 nm except the ones labelled with (f).

f PSD achieved by 405 nm light irradiation.

**Table:** 

	Dy–yD		Ay–yA		Dy–yA		D–yA	
Solvent	THF	MeCN	MeCN	Water	THF	MeCN	THF	MeCN
*aλ* ^O^_max_ (nm)	362	363	371	377	371	369	364	364
*ε* (10^3^ M^−1^ cm^−1^)	85.1	84.8	67.1	69.5	71.0	72.4	58.6	55.7
*λ* ^C1^_max_ (nm)	483	479	411	415	—	—	484	484
*ε* (10^3^ M^−1^ cm^−1^)	26.7	27.4	11.8	11.6	—	—	10.8	10.4
*λ* ^C2^_max_ (nm)	671	673	643	650	718	693	757	753
*ε* (10^3^ M^−1^ cm^−1^)	47.3	41.9	22.3	23.1	37.8	37.6	32.3	35.1
PSD (%)	>99	86	>99	>99/96^f^	80	33	66	26
*Φ*_O–C_ (%)	5.5 ± 0.2	0.11 ± 0.01	9.2 ± 0.5	17 ± 1	0.077 ± 0.004	0.012 ± 0.001	0.028 ± 0.002	5.0 ± 0.3 × 10^−3^
*Φ*_C–O_ (%)	0.026 ± 0.002	0.026 ± 0.001	0.13 ± 0.01	0.12 ± 0.01	0.039 ± 0.003	0.039 ± 0.004	0.047 ± 0.003	0.040 ± 0.003

The impact of additional alkynes on the closed push–push and pull–pull DTEs is subtle, with only a 6 nm red-shift of the lowest energy absorption of Dy–yD observed compared to D–D and 15 nm blue-shift of Ay–yA compared to A–A. The red-shift of Dy–yD compared to D–D can be attributed to the extended length of π conjugation. In contrast, for A–A and Ay–yA, ICT can readily take place between the electron-rich central thiophene groups and the electron-deficient *N*-methylpyridinium groups. The charge transfer is strongest when the electron acceptor is directly connected to the electron donor, and becomes weaker with additional alkynes.^50^ Therefore, the excited state of Ay–yA is less stabilized by the polar MeCN compared to A–A, resulting in the hypsochromic shift of the absorption band.

Despite having no clear impact on the absorption maximum of the open DTEs, the push–pull substitution pattern significantly red-shifts the absorption maximum of the closed DTE, as illustrated by the closed D–A absorbing at 792 nm while D–D and A–A absorb at 667 nm and 658 nm, respectively. The >100 nm bathochromic shift of the absorption band is attributed to the ICT between the electron-donating julolidine arm and the electron-accepting *N*-methylpyridinium arm. The absorption band of closed D–A is blue-shifted by >100 nm upon protonation by trifluoroacetic acid, but recovered when excess triethylamine is added (Fig. 3d). A similar red-shift of the closed DTE absorption due to donor–acceptor interaction is observed in the series of compounds with two additional alkynes (one on each arm). However, the red-shift of Dy–yA (50 nm red-shift compared to Ay–yA) is less profound than that of D–A. The closed-form absorption maxima of Dy–A and D–yA with one alkyne in the backbone lie in between the absorption of D–A and Dy–yA, at 724 nm and 753 nm, respectively, indicative of the insulating effect of triple bonds in the donor–π–acceptor conjugated system.

**Fig. 4:**
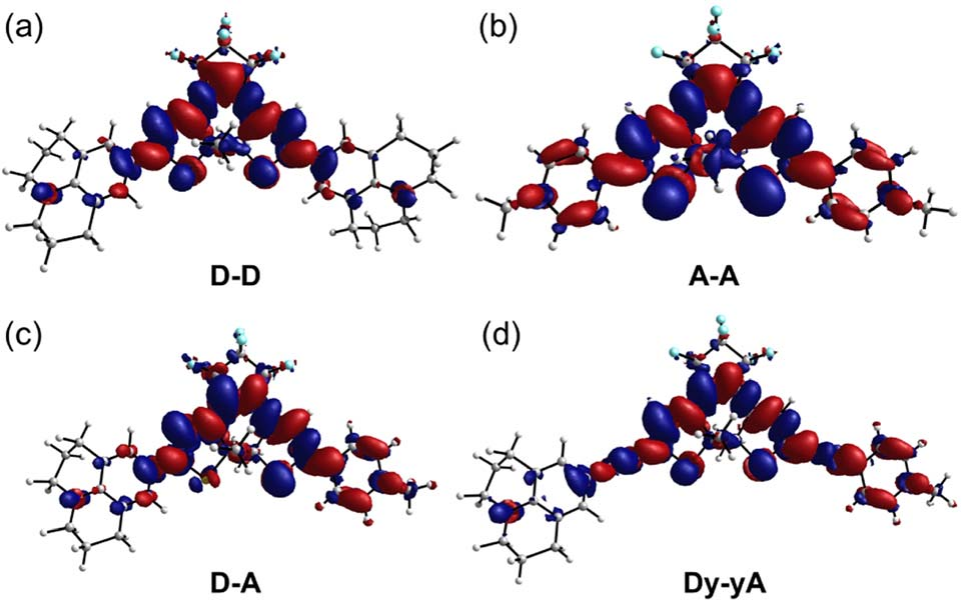
Electron density difference map (contour value: 0.0008) of (a) D–D, (b) A–A, (c) D–A and (d) Dy–yA (all closed isomers) upon excitation. Blue and red colors indicate the regions of the molecule that donate and accept electron density, respectively.

In an attempt to understand the redistribution of electron density upon excitation, we used density functional theory (DFT) and time-dependent DFT (TD-DFT) to simulate the vertical transition energies. We first performed a systematic benchmarking of density functional approximations, based on the experimental UV-vis absorption maxima and published X-ray structures of related DTEs.^46,51,52^ We found that geometries optimized using the ωB97X-D functional best resemble published X-ray structures (Section S13†), and that TD-DFT calculations using this functional (with SMD solvent model and acetonitrile as the solvent; Def2-SVP basis set) best reproduce the experimental *λ*_max_ (Section S13†). The ωB97X-D long-range corrected hybrid density functional employs 100% Hartree–Fock (HF) exchange for long-range electron–electron interactions, which makes it suitable for modeling charge transfer states. TD-DFT results demonstrate that, for all open DTEs, the HOMO–LUMO transition is the dominant contribution to the longest wavelength absorption band. The electron density of both ground state and excited states was then calculated and the electron density difference (EDD) maps were produced by subtracting the ground state density cube from the excited state density cube (contour value: 0.0008; Fig. 4). The EDD maps show that the ICT within DTEs is more complex than in a conventional D–π–A system. While a conventional π-linker only acts as a pathway for electron transfer, the tetracycle of a cyclized DTE itself can act as an electron donor or acceptor, depending on electronics of the substituents. When both arms are substituted with electron-rich julolidine, the electron density transfers to the central perfluorinated ring upon excitation. For A–A (Fig. 4b), the central tetracycle donates electron density into the electron-deficient *N*-methylpyridinium arms upon transition. Thus, A–D–A is a better model for describing the compound as electron density transfers from the electron-rich thiophene core to the arms. The EDD map of D–A (Fig. 4c) indicates that the julolidine acts as an electron donor. However, the central tetracycle also donates electron density into the *N*-methylpyridinium group. With additional alkynes in the arm, Dy–yA (Fig. 4d), the alternating color at the alkyne site indicates the electron density is donated into the carbon–carbon single bond during the transition and the alkyne arms gain cumulenic character upon excitation.

### Photostationary state distribution

After an initial assessment of photochromism, we measured the PSD on irradiation at 365 nm for each compound in two different solvents (Table 1), *i.e.*, THF and MeCN for neutral and monocationic species; MeCN (as hexafluorophosphate salts) and water (as chloride salts) for pull–pull DTEs. The ratios of the open and closed isomers were determined by comparing the ^1^H NMR integrations of the thiophene protons before and after irradiation at 365 nm. For push–pull DTEs, the PSD cannot be easily achieved in MeCN at NMR concentrations due to slow switching. Thus, their PSDs were characterized by UV-vis spectroscopy (see Section S7†).

For the forward photocyclization reaction (365 nm), exceedingly high PSDs (>99%) are achieved by push–push D–D and Dy–yD in THF, and by pull–pull A–A and Ay–yA in MeCN and water (Table 1). Changing from THF to more polar MeCN has a significant impact on the PSDs of push–push DTEs, illustrated by a reduction of over 10% in PSDs of both compounds. Solvent polarity has negligible impact on the PSDs of A–A and Ay–yA, and they can be quantitatively switched in both MeCN and water. Push–pull DTEs have lower PSDs compared to push–push or pull–pull DTEs. The highest PSD of 85% is achieved by Dy–A in THF and the lowest PSD of 21% is achieved by D–A in MeCN. The PSDs of push–pull compounds are strongly affected by the solvent polarity, manifested by a reduction from 71% to 21% of D–A when changing from THF to MeCN. While the PSDs for photocyclization on irradiation at 365 nm vary for different DTEs and in different solvents, the PSD of the photoreversion reaction on irradiation at 630 nm is quantitative for all compounds in all solvents, due to lack of absorption by the open DTE at 630 nm.

To investigate whether the additional alkyne, and the resulting red-shift of the open-form absorption band, could permit DTEs to be switched with visible light, we measured the PSD of the chloride salts of A–A and Ay–yA in water after irradiation at 405 nm. The results in Table 1 demonstrate that A–A can achieve 71% PSD while Ay–yA can achieve 96% PSD, *i.e.* the additional alkynes on each arm leads to a 25% boost in PSD under 405 nm visible light irradiation. To the best of our knowledge, the chloride salt of Ay–yA is the first example of water-soluble non-fluorescent DTE that can be switched to over 95% PSD in the forward direction and quantitatively in the reverse reaction with visible light.

The extent of spectral separation between the open and closed DTEs is a key contributing factor that affects the PSDs. For the push–push and push–pull DTEs, the band separation is not optimal at 365 nm, the wavelength of irradiation. Generally, the molar absorption coefficient of the open form is two to five times greater than the closed form. Thus, in the small band separation scenario, the predominant factor leading to the 99% PSD of D–D and Dy–yD in THF is the much greater quantum yield of photocyclization compared to the quantum yield of photoreversion. The sensitivity of the PSD to the polarity of solvent environment can be mainly attributed to the different ratio of photocyclization quantum yield to photoreversion quantum yield in different solvents. For A–A at 365 nm, the molar absorption coefficient of the open form (42 300 M^−1^ cm^−1^) is approximately nine times greater than that of the closed form (4800 M^−1^ cm^−1^). Thus, the band separation plays a more important role leading to >99% PSD after 365 nm light irradiation compared to D–D. The process of photocyclization of Ay–yA triggered by 405 nm light is an intriguing example of a photoswitch in the small band separation scenario, where the closed form (11 800 M^−1^ cm^−1^) absorbs more strongly than the open form (9800 M^−1^ cm^−1^). Nevertheless, a high PSD of 96% is achieved due to the exceedingly high photocyclization quantum yield and low photoreversion quantum yield. This example breaks the traditional rule for photoswitch design, which states that large band separation is crucial for a photoswitch to achieve high PSD.

### Quantum yield of photoswitching

To quantify the efficiency of the forward and reverse photoswitching processes of the DTEs, we measured the photocyclization and photoreversion quantum yields of all eight DTEs using the initial rate of switching in dilute solution, as reported by Ozcoban and co-workers.^53^ In order to determine the reaction quantum yields of photocyclization and photoreversion, the excited state of the DTEs should not enter other irreversible side-reaction pathways (Section S5†). This assumption is valid for most DTEs, as the most well-defined decomposition pathway starts with the UV-excitation of the closed DTE, leading to the formation of an annulated ring.^54^ This side-reaction is slow and is likely to have a negligible effect when 5–10% of open form DTE is converted to the closed form to obtain the initial slope.

The quantum yields of UV-induced photocyclization are generally high (*Φ*_O–C_ = 5–38%) for push–push DTEs (in non-polar solvents like THF) and pull–pull DTEs, but over two orders of magnitude lower for push–pull DTEs (in THF) (Table 1). A–A has two positive charges on the arms (which favors the anti-parallel conformation due to coulombic repulsion) and it has the highest photocyclization quantum yield (38% in water), which is over five-times higher than D–D in THF (7.7%). Similarly, the cyclization quantum yield of dicationic Ay–yA in water (17%) is approximately three-times higher than Dy–yD (5.5%) in THF. We observe a reduction in cyclization quantum yield with insertion of alkynes into the backbone for push–push and pull–pull compounds, and this can be attributed to the smaller orbital contribution of the singlet excited state to the photoactive central hexatriene moiety, with extended π-conjugation,^55^ however the cyclization quantum yields of push–pull DTEs increase from 0.020% (D–A) to 0.077% (Dy–yA). The wide range of photocyclization quantum yields of DTEs with different substituents leads to a wide variation in the irradiation times required to reach the photostationary state. In the concentration range of 2.5–5 μM (Fig. 2 and S4†), under irradiation at 365 nm, A–A reaches the PSS in 15 s in MeCN with 12.33 mW irradiation (0.56 mol of photons), whereas D–A takes 800 s in THF with 159 mW irradiation (388 mol of photons).

Solvent polarity also significantly affects the cyclization quantum yields of the DTEs. We observe a reduction of more than an order of magnitude in the cyclization quantum yield for push–push compounds when changing from THF to MeCN, which is more polar. A smaller two-fold reduction is observed for push–pull DTEs. In contrast, pull–pull DTEs demonstrate an increase in quantum yield when changing from MeCN to water (accompanied by counterion exchange, hexafluorophosphate to chloride). The reduced cyclization quantum yields of DTEs in polar solvents have been reported previously and attributed to the formation of twisted intramolecular charge transfer (TICT) states.^56,57^ The most thorough investigation comes from a study of fluorescent DTEs with a maleic anhydride central bridge.^57^ The fluorescence signal of the compounds demonstrated a two-component decay and the contribution of the slower decaying component increased with the increasing solvent polarity. Together with a bathochromic shift of fluorescence spectra and a reduction in fluorescence intensity in more polar solvents, the authors postulated a TICT state in the excited state that does not lead to cyclization. A related study found that a TICT state can be formed in DTEs when one or both thiophene arms are substituted with electron-rich *N*,*N*-dimethylaniline.^57^ As julolidine is an extremely good electron donor and all our DTEs have perfluorinated central bridges, the formation of a TICT state could explain the lower cyclization quantum yield of both push–push and push–pull DTEs in MeCN. Other possible explanations for the low quantum yields of the julolidine-functionalized DTEs in polar solvents include the formation of other types of intramolecular or intermolecular charge-separated states.

**Fig. 5:**
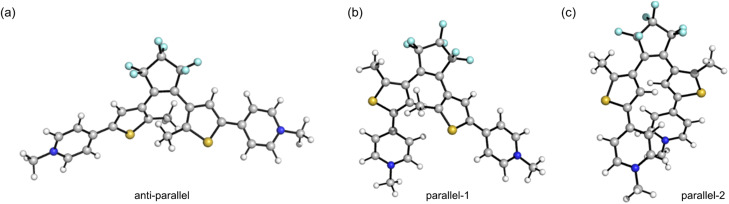
Schematic illustration of the (a) anti-parallel, (b) parallel-1 and (c) parallel-2 conformations of DTE molecules.

We utilized DFT at the ω-B97XD/Def2SVP level of theory (with SMD solvent model and acetonitrile as the solvent) and CREST-xtb conformational search (Section S16†)^58^ to explore the impact of ground-state geometry on the photocyclization quantum yield, taking D–D, A–A, D–A and Dy–yA as representative push–push, pull–pull and push–pull molecules. The geometries of the local minima can be categorized into three main groups for D–D, D–A and Dy–yA (Scheme 1 and Fig. 5): one anti-parallel geometry (the geometry for cyclization) and two parallel geometries. For A–A, the parallel-2-type conformer was not found due to the electronic repulsion between two positively charged groups. The energies (sum of electronic and zero-point) and Boltzmann distributions of these conformers at 298 K are listed in Table 2; see Section S16† for details. For neutral D–D and mono-cationic D–A and Dy–yA, the parallel-2 conformation with the two DTE arms stacking with each other has the lowest energy. This conformation accounts for 97.8% of the population for D–A, 93.0% for Dy–yA and 87.3% for D–D at room temperature, negatively correlating with the photocyclization quantum yield. This result demonstrates that in molecules where the most stable parallel-2 conformer is heavily populated, photocyclization is disfavored. For dicationic A–A, the anti-parallel conformation is most stable. A Boltzmann distribution of >99% in the anti-parallel conformation indicates that A–A exists in a conformation predisposed for photocyclization.

**Table:** Relative energies (from the sum of electronic and thermal free energies) and Boltzmann distributions (%) of different conformers of D–D, A–A, D–A and Dy–yA

Conformation	D–D		A–A		D–A		Dy–yA	
	Energy (kJ mol^−1^)	Distribution (%)	Energy (kJ mol^−1^)	Distribution (%)	Energy (kJ mol^−1^)	Distribution (%)	Energy (kJ mol^−1^)	Distribution (%)
Anti-parallel	4.9	12.1	0	>99.9	11.2	0.8	7.1	3.9
Parallel 1	14.2	0.6	20.8	<0.1	12.2	1.4	9.9	3.1
Parallel 2	0	87.3	—a	0	0	97.8	0	93.0

a Local energy minimum was not found for A–A in the parallel-2 conformation.

The cycloreversion (closed to open) quantum yields of the DTEs are generally smaller than the cyclization quantum yield, in agreement with majority of the DTEs in the literature.^46^ We found that pull–pull DTEs generally have higher cycloreversion quantum yields than push–push or push–pull DTEs. The cycloreversion quantum yields of A–A and Ay–yA are approximately four-times higher than those of D–D and Dy–yD, respectively. Cycloreversion quantum yields of push–pull DTEs are comparable to those of push–push DTEs. For push–push and pull–pull DTEs, a decline in cycloreversion quantum yield is observed with increased length of conjugation due to smaller orbital contribution to the photoactive core. However, for push–pull DTEs, the cycloreversion quantum yield increases by three-times when incorporating additional alkynes in both arms. The cycloreversion quantum yields of the DTEs show no dependence on the solvent polarity. Experimentally, using our setup for irradiation at 630 nm, the cycloreversion in the concentration range of 2.5–5 μM can be completed within two minutes for all push–push and pull–pull systems. In contrast, it takes over one hour to fully switch D–A back from the closed form to the open form. The slow reverse reaction can be explained by the low absorption coefficient of D–A at 630 nm, due to the bathochromic shift of its closed-form absorption maximum to 792 nm.

### Fatigue resistance

All the DTEs can be switched reversibly between the open form and the closed form with 365 nm (photocyclization) and 630 nm (photoreversion) light. The fatigue resistance of each DTE was characterized by repeatedly switching between the open and closed forms and monitoring the change of the UV-vis signal maximum corresponding to the closed DTE (Fig. 2 insets). Among the eight DTEs, D–D, A–A and Ay–yA are highly fatigue resistant as they remain switchable after a hundred cycles of photoswitching. By comparing the intensity of absorption with the initial intensity, we found that 33% of D–D, 21% of A–A and 24% of Ay–yA decomposed after a hundred switching cycles.

Push–pull DTEs generally have much worse fatigue resistance. The most fatigue resistant push–pull DTE, Dy–yA has a photoswitching half-life of 18 cycles while D–A has a photoswitching half-life of only 5 cycles, at the same irradiation power. Instead of indicating fast decomposition per photon (high decomposition quantum yield), the poor fatigue resistance of push–pull DTEs actually originates from the low photocyclization quantum yield because the fatigue resistance curve is a measure of how many cycles a photoswitch can be switched. While the PSS of pull–pull DTEs can be achieved in ten seconds, it takes over ten minutes to reach the PSS of D–A. Thus, the push–pull samples are exposed to more photons during the process of measuring fatigue resistance curve. Even if the decomposition quantum yields of the pull–pull and push–pull DTEs were the same, we would expect much worse fatigue in the push–pull DTEs due to slower cyclization.

Most DTEs decompose into by-products that do not absorb in the visible region as the absorbance decays (Fig. 2). However, Dy–yD shows a distinctive decomposition pathway as the absorbance after 630 nm light irradiation gradually increases, indicating the formation of a by-product that resembles the closed DTE. This by-product cannot be switched back by visible light (as discussed in the next section).

**Fig. 6:**
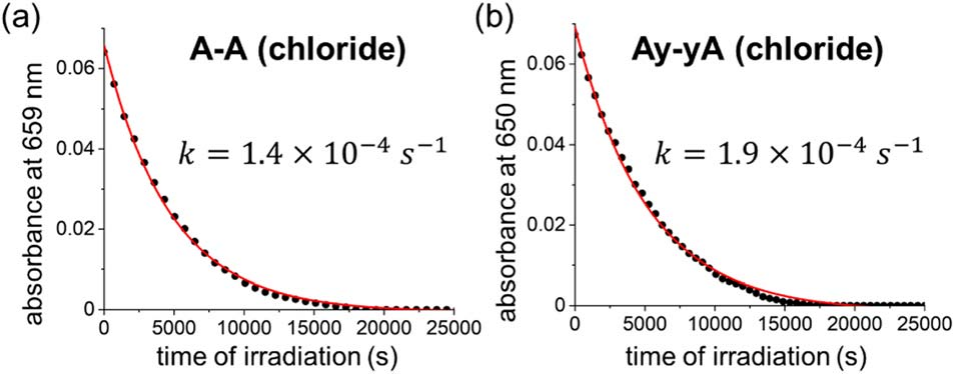
Decomposition kinetic trace of closed (a) A–A and (b) Ay–yA under continuous 365 nm light irradiation (112 mW) in water monitored at 659 nm and 650 nm, respectively.

We measured the quantum yield of photoinduced decomposition for two of the best-performing DTEs, A–A and Ay–yA (as chloride salts) in water, assuming that the decomposition only takes place from the closed DTE after UV light irradiation (Fig. 6). The decomposition curve was measured by continuously irradiating the closed DTE with 365 nm light at 112 mW until full decomposition. The decomposition quantum yields of 0.0020% ± 0.0003% and 0.0026% ± 0.0006% for A–A and Ay–yA, respectively, were extracted from the kinetic data after exponential decay curve fitting. These quantum yields for decomposition are 13 000 and 5700-times smaller than the cyclization quantum yields from irradiation at the same wavelength (365 nm), respectively, which indicates that these DTEs can undergo >5000 switching cycles before decomposition predominates.

### Decomposition of Dy–yD

**Fig. 7:**
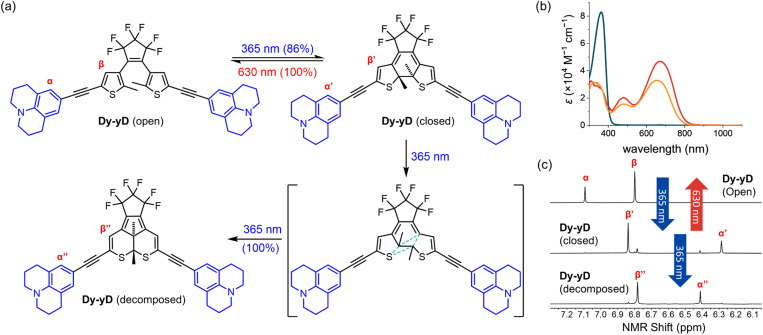
(a) Proposed decomposition pathway of Dy–yD. (b) Absorption spectra of open-form (blue), closed-form (red), and decomposed product (orange) of Dy–yD recorded in THF. (c) Partial ^1^H NMR spectra of open-form, closed-form, and decomposed product of Dy–yD (d_8_-THF, 500 MHz, 298 K).

In order to characterize the decomposition, samples of Dy–yD at the PSD (*i.e.* >99% closed, as determined by NMR after irradiation at 365 nm), were further irradiated with 365 nm light for 3 hours in THF (see Section S11†). Dy–yD is converted into a single clean product, according to NMR and TLC, with an absorption band resembling the closed form Dy–yD but hypsochromically shifted by approximately 20 nm (Fig. 7b), indicating that the length of π-conjugation resembles that of closed Dy–yD. ^1^H NMR spectra (Fig. S33 and S35†) show the two methyl groups on the thiophene moieties are equivalent for Dy–yD (open) and Dy–yD (closed) but become inequivalent (two proton signals) after decomposition. Moreover, the two quaternary thiophene carbon centers and the two methyl carbons of closed Dy–yD are equivalent according to ^13^C NMR (Fig. S34 and S36†), but become inequivalent after decomposition (Fig. S36 and S38†). We conclude that Dy–yD decomposes in a pathway shown in Fig. 7a, similar to the previous report by Irie and co-workers,^54^ which was further investigated by the Hecht group.^44^ This decomposition pathway was rationalized by Jacquemin's computational study, which used all molecular mechanics-valence bond (MMVB), complete active space self-consistent field (CASSCF) and complete active-space second-order perturbation theory (CASPT2) methods predict a canonical insertion that leads to the formation of the annulated by-product as shown in Fig. 7a.^59^

As only Dy–yD (but not D–D) among the eight DTEs cleanly decomposes into the annulated product, we conclude that the electron-donating ability of the aryl substituent is not the only key factor contributing to the by-product formation, although Hecht and co-workers reported that the yield of annulated by-product formation is generally higher for DTEs with electron-donating substituents.^44^

### Solvatochromism of D–A

**Fig. 8:**
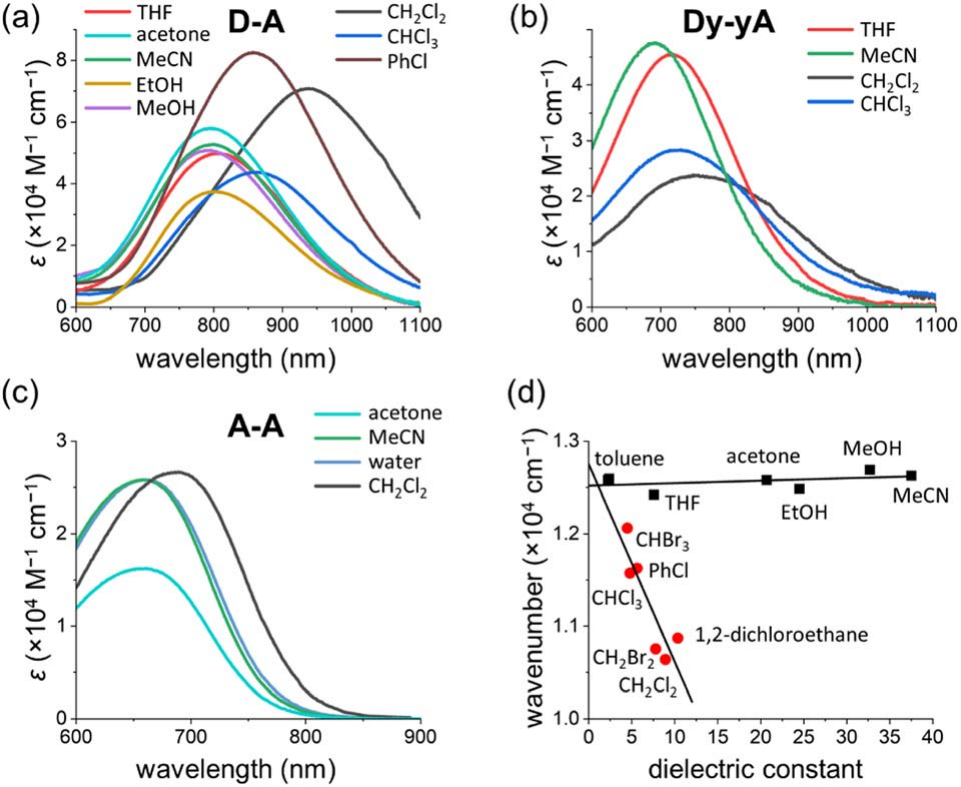
Solvatochromism of (a) D–A, (b) Dy–yA and (c) A–A. (d) Linear fits of solvatochromic behavior in non-halogenated solvents (black squares) and halogenated solvents (red dots). The intercept dielectric constant of the two linear fits is 1.08.

The UV-vis absorption spectra of D–D, A–A, D–A and Dy–yA were recorded in a range of solvents with different polarities to test the solvatochromic behavior (Fig. 8 and S39†). No clear solvatochromic behavior is observed for D–D and A–A in THF, MeCN and acetone. For push–pull D–A, a small hypsochromic shift is observed in polar solvents (∼8 nm shift from THF to MeCN). The hypsochromic shifts are greater for Dy–yA (25 nm shift from THF to MeCN). The negative solvatochromism of D–A and Dy–yA (hypsochromic shift with increasing polarity) is not surprising because formation of the ICT state moves positive charge away from the pyridinium acceptor, which becomes more difficult when this charge is well solvated.^60–63^

A more intriguing solvatochromic behavior is observed with halogenated solvents. Bathochromic shifts of the absorption spectra are observed in halogenated solvents for A–A, D–A and Dy–yA. The shift is most profound for D–A in dichloromethane, where the absorption maxima is over 150 nm red-shifted compared to the absorption in non-halogenated solvents. Similar bathochromic shifts are observed in other halogenated solvents such as chloroform, dibromomethane, iodomethane and chlorobenzene but not in carbon tetrachloride (Fig. S39†). A plot of energy of absorption *vs.* dielectric constant (Fig. 8d) demonstrates that the absorption maxima of D–A show linear dependence on the solvent polarity but the gradients of linear fits are different between non-halogenated and halogenated solvents. Thus, the abnormal solvatochromic behavior in halogenated solvents must originate from a specific interaction between the solvent and the molecule. After fitting the measured absorption maxima with the Kamlet–Abboud–Taft model, a multi-regression solvent model that takes solvent polarity, hydrogen-bond donating ability, and hydrogen-bond accepting ability into consideration, we ruled out all three factors being responsible for the abnormal red-shift of the absorption of pull–pull and push–pull DTEs.^64,65^ Comparing the extent of absorption peak shift in dichloromethane, dibromomethane and diiodomethane, we found that the spectral shift is most prominent in chlorinated solvents and least prominent in iodinated solvents. This observation is not consistent with the rationalization provided by Ponnusamy and co-workers, who concluded that halogen bonding between the counterion and the halogenated solvents leads to the abnormal shift of absorption.^66^ It appears that halogenated solvents have special impact on the ion pairing between the ionic DTE and counterion but the exact nature of this interaction is not yet clear.

## Conclusions

We have described the synthesis, photochemistry, photophysics, and DFT and TD-DFT calculations of eight DTEs bearing push–push, pull–pull and push–pull substitution patterns with different conjugation lengths in the backbone. The DTEs with symmetrical conjugated patterns (with or without alkynes) on both arms can be synthesized starting with the formation of the DTE core, and then functionalizing with the arms using Suzuki or Sonogashira coupling, while DTEs with an alkyne on one arm can be prepared with DTE core formation at a late stage. Both routes permit straightforward synthesis of DTEs bearing versatile substituents.

The photocyclization and photoreversion abilities of DTEs critically depend on the substitution pattern. The cationic pull–pull DTEs generally demonstrate higher photocyclization quantum yields than push–push or push–pull DTEs due to Coulomb repulsion between the two positive charges, forcing the DTE to adopt an anti-parallel conformation in the ground state that can cyclize in a conrotatory fashion, leading to extremely high PSDs. In contrast, the parallel conformations of push–pull DTEs are stabilized by interactions between the two arms, inhibiting photocyclization. Solvent polarity also affects the quantum yield of photoswitching. The impact of solvent polarity is most profound on push–push DTEs where TICT states may be accessed by rotation around the thiophene–phenyl bond.

Fatigue resistance measurements show that the fatigue does not simply relate to the substitution pattern. D–D, A–A and Ay–yA are highly fatigue resistant. However, Dy–yD decomposes in a distinct mechanism leading to the formation of an annulated ring. Push–pull DTEs generally show poor fatigue resistance due to slow photocyclization, *i.e.* the compounds need to be irradiated longer in each cycle of switching, resulting in decomposition.

We have completed a systematic study on the structure–property relationship of DTEs bearing push–push, pull–pull and push–pull substitution patterns, offering guidance for future DTE design for different applications. Ay–yA is the first reported DTE that is water-soluble, can be switched in both directions with visible light, reaching over 95% PSD in the forward reaction and 100% PSD in the reverse reaction. These properties make Ay–yA a promising candidate for potential application in biological contexts such as super-resolution microscopy.

## Data availability

All the relevant data are provided as part of the ESI.†

## Author contributions

S. Q. synthesized and characterized all compounds. S. Q. and A. T. F. conducted the measurements of photophysical and photochemical properties. S. Q. and K. G. L. carried out DFT and TD-DFT calculations. A. T. F. and H. L. A. conceptualized the project. H. L. A. managed the project and acquired the funding. S. Q., A. T. F. and H. L. A. wrote the manuscript. All authors contributed to editing and reviewing the manuscript.

## Conflicts of interest

There are no conflicts to declare.

## Supplementary Material

SC-014-D3SC01458D-s001
