# Supplementary material for: How do donor and acceptor substituents change the photophysical and photochemical behavior of dithienylethenes? The search for a water-soluble visible-light photoswitch
Source: Chem Sci. 2023 Jul 31;14(34):9123–35. doi: 10.1039/d3sc01458d (PMC10466371; doi:10.1039/d3sc01458d)
Supplement: SC-014-D3SC01458D-s001 [file SC-014-D3SC01458D-s001.pdf]

Electronic Supplementary Material (ESI) for Chemical Science.
This journal is © The Royal Society of Chemistry 2023

Electronic Supporting Information

How do donor and acceptor substituents change the photophysical and photochemical behavior of dithienylethenes? The search for a water-soluble visible-light photoswitch

Sili Qiu, Andrew T. Frawley,* Kathryn G. Leslie and Harry L. Anderson*

Department of Chemistry, University of Oxford, Chemistry Research Laboratory, Oxford, OX1 3TA, UK.

E-mail: andrew.frawley@chem.ox.ac.uk, harry.anderson@chem.ox.ac.uk

Table of Contents

S1. General Procedures....S2

S2. Synthesis of Target Compounds....S4

S3. Acquisition of the UV-Vis Spectra of Open-Form, PSS and Closed-Form....S10

S4. Determination of Molar Absorption Coefficients....S16

S5. Determination of Photocyclization and Photoreversion Quantum Yields....S17

S6. Determination of Percentage Conversion at Photostationary State by ^{1}H NMR Spectroscopy....S25

S7. Determination of Percentage Conversion at Photostationary State by UV-Vis Spectroscopy....S30

S8. Calculation of PSD based on Experimental Molar Absorption Coefficient and Quantum Yield....S32

S9. Determination of Fatigue Resistance by UV-Vis Spectroscopy....S33

S10. Verification of Intramolecular Charge Transfer in **D-A** by Protonation....S36

S11. Characterization of the Decomposition of **Dy-yD**....S37

S12. Absorption Solvatochromism of Closed-Form **D-A**....S42

S13. Functional Benchmarking of DFT and TD-DFT Calculations....S45

S14. Comparison of ωB97X-D and CAM-B3LYP Functionals for Spectral Calculation of DTEs....S49

S15. Electron Density Difference Plot between Ground and Excited State....S51

S16. Conformational Analysis on the DTEs....S52

S17. Synthetic Procedures....S54

S18. Spectral Data....S70

S19. Coordinates of Optimized Geometries of DTEs (ωB97X-D/Def2SVP)....S128

S20. References....S143

S1. General Procedures

All reactions were performed using oven-dried reaction vessels under an argon atmosphere unless stated otherwise. Anhydrous acetonitrile (MeCN), chloroform ($CHCl_3$), dichloromethane (CH_2Cl_2), diethyl ether (Et_2O), tetrahydrofuran (THF), 1,4-dioxane, and triethylamine (Et_3N) were obtained from an MBraun MBSPS-5-BenchTop solvent purification system, having been passed through an activated alumina column under nitrogen. Methanol was dried over 4 Å molecular sieves under argon. All other chemical reagents used were commercially available from Alfa Aesar, Fluorochem, Merck, and TCI Chemicals and were used as supplied.

Thin layer chromatography (TLC) analysis was carried out using Merck aluminum-backed TLC silica gel 60 F_{254} pre-coated plates. Plates were visualized by the quenching of fluorescence under ultraviolet light (λ_{max} = 254 nm), turn-on of fluorescence under ultraviolet light (λ_{max} = 356 nm), or color changes of photoswitches after ultraviolet light (λ_{max} = 365 nm) irradiation. Flash column chromatography and size exclusion chromatography were performed using Merck Geduran® 60 silica gel (particle size 63–200 µm) or Bio-Rad Bio-Beads S-X3 styrene divinylbenzene beads (3% cross linkage, 40–80 µm bead size, ≤2,000 MW limit), respectively, with the solvent system given. All solvents used for chromatography purification were HPLC grade or equivalent and were supplied by Merck, Fisher Scientific or VWR.

Proton (^1H) and carbon (^{13}C) NMR spectra were recorded on either a Bruker AVIII HD 400, a Bruker AVIII HD 500, a Bruker AVII 500 with a $^{13}C(^1H)$ dual cryo-probe, or a Bruker AVIII 600 instrument with a broadband cryo-probe in deuterated solvents. Fluorine (^{19}F) NMR spectra were recorded on either a Bruker AVIII HD 400 or a Bruker AVIII HD 500 instrument in deuterated solvents. Boron (^{11}B) NMR spectra were recorded on a Bruker AVIII HD 400 instrument in deuterated solvents. ^1H NMR chemical shifts were reported in ppm to the nearest 0.01 ppm relative to $SiMe_4$ ($\delta = 0$) and were referenced internally with respect to residual protons in the deuterated solvent (δ = 2.05 for acetone-d_6, 1.94 for acetonitrile-d_3, 7.26 for chloroform-d, 2.50 for DMSO-d_6, 3.31 for methanol-d_4, and 3.58 for tetrahydrofuran-d_8). ^{13}C NMR spectra were recorded with broadband decoupling. ^{13}C chemical shifts were reported in ppm to the nearest 0.1 ppm relative to $SiMe_4$ (δ = 0) and were referenced internally with respect to residual carbons in the solvent (δ = 118.6 for acetonitrile-d_6, 128.1 for benzene-d_6, and 77.2 for chloroform-d). ^{19}F NMR chemical shifts were reported to the nearest 0.1 ppm relative to $CFCl_3$ (δ = 0). ^{11}B chemical shifts were reported in ppm to the nearest 0.1 ppm relative to $BF_3{\cdot}OEt_2$ (δ = 0). Peak assignments were made based on chemical shifts, integrations, coupling constants, comparison to known compounds, and using COSY, HSQC, and HMBC experiments where appropriate.

Multiplets are described as singlet (s), doublet (d), triplet (t), quartet (q), pentet (p) multiplet (m), broad (br), or combinations thereof. Coupling constants (*J*) are reported to the nearest 0.1 Hz.

High resolution mass spectra (HRMS) were recorded by the Chemistry Research Laboratory Mass Spectrometry Facility, University of Oxford, using a Bruker MicroTOF spectrometer or a Thermo Orbitrap Exactive mass spectrometer. The mass reported was that containing the isotopes with the lowest mass, with each value to 7 significant figures and within 5 ppm of the calculated mass. Mass to charge ratios (*m/z*) are reported in Daltons.

Stock solutions of all final photoswitches were prepared by dissolving 5.0−15.0 mg of a photoswitch in tetrahydrofuran-d_8 or acetonitrile-d_3 (1.0 mL) containing dioxane (2.34 mM) as a concentration reference. The ^{1}H NMR spectra of the samples were acquired on a 500 MHz spectrometer with an extended relaxation delay (30 s) for accurate integration. The exact concentration of the DTE was determined by comparing the integration of the thiophene-proton on the DTE with the integration of the dioxane signal, assuming 1% error associated with the NMR integration. The error estimation was based on setting the integration of C(2)H (2 protons) of compound **D-D** to 2.00 (see **Section S6**) and then randomly integrating the baseline where no peak occurs (with the width of 0.05 ppm) ten times, giving an average uncertainty of ±0.02 (1% error in integration value). The stock solution was stored under an argon atmosphere in the dark at −20 °C. The stock solution was diluted to the concentration range of 0.5–3.0 μM in HPLC-grade solvents or deionized water for spectroscopic measurement unless otherwise specified. UV-vis absorption spectra were acquired on a Perkin Elmer Lambda 20 or Lambda 25 spectrometer using quartz glass cuvettes from Starna (10 mm path length). All measurements were conducted at 25 °C unless otherwise stated, with the temperature controlled by a water-cooled PTP-1 Peltier unit from Perkin Elmer. Photoswitching experiments were conducted using Prizmatix collimated modular Mic-LED light sources including mic-LED-365L (centered at 365 nm, FWHM = 14 nm), mic-LED-405L (centered at 405 nm, FWHM = 18 nm), and mic-LED-630 (centered at 630 nm, FWHM = 15 nm) at the given power setting. The intensity of the light sources was controlled by a Benchtop LED Current Controller from Prizmatix. The power of the light source (in mW) was measured by a Coherent® PowerMax™-USB/RS Sensor System, which was calibrated against ferric oxalate chemical actinometry as described previously (**Section S5**).[1] To measure the forward cyclization and the reverse ring-opening processes, two LEDs were joined into one output beam via a beam combiner, which was coupled to a liquid light guide (diameter = 3 mm). The liquid light guide was wired close to the cuvette holder and orthogonal to the beamline of the

spectrometer, as shown in **Figure S1**. The irradiation was performed using MATLAB script to control irradiation time, interval time, and the number of cycles of irradiation.

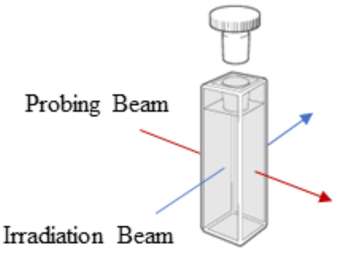

Figure S1 Schematic representation of the geometrical arrangement of the sample holder in the UV-vis spectrometer for measurements of the PSS spectrum, quantum yields of photochemical ring-closure and ring-opening, and fatigue resistance.

S2. Synthesis of Target Compounds

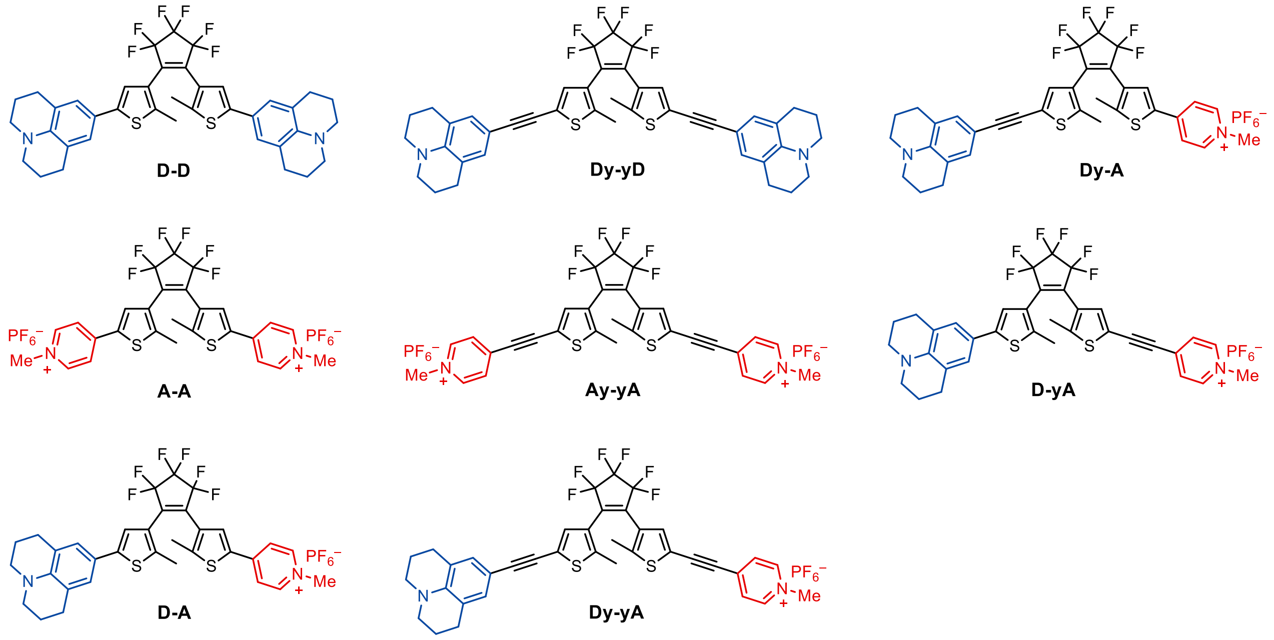

Figure S2 Summary of push-push, pull-pull, and push-pull DTEs with various conjugation lengths synthesized in this study. For **A-A** and **Ay-yA**, the chloride salts were also synthesized (see **Scheme S7**) to increase their water solubility for photophysical and photochemical characterization.

We prepared a collection of push-push, pull-pull, and push-pull DTEs with different lengths of conjugation, as shown in **Figure S2**, intending to understand the impact of electron donor and acceptor, as well as the length of conjugation on the photophysical and photochemical properties of DTEs. Julolidine and *N*-methylpyridinium groups serve as the electron-donating and electron-accepting groups, respectively. Compounds with short conjugation contain a functional end-group directly attached to the thiophene of the

DTE core. The conjugation is extended by incorporating additional alkynes between the thiophene and the functional end-group.

Push-push **D-D**, pull-pull **A-A**, and push-pull **D-A** with functional groups directly linked to the central thiophene were synthesized following the route shown in **Scheme S1** and **Scheme S2**. The synthetic route started with the formation of a photoswitchable core DTE **1** dichloride following a procedure developed by Hermes and co-workers.[2] The boronic acid pinacol ester coupling partner **3** was synthesized by aromatic halogenation following a published procedure[3] with slight modification in the purification method followed by a reported Miyaura borylation procedure with modified palladium catalyst ligand **(Scheme S1)**.[4] The structure of core DTE **1** was then extended by Suzuki-Miyaura coupling with julolidine-Bpin **3**, pyridine-Bpin **2**, or both in one pot to yield **D-D**, **A-A(p)**, and push-pull **D-A(p)**, respectively. To form **A-A** and **D-A**, the pyridine motif of **A-A(p)** and **D-A(p)** was methylated with methyl iodide to generate an electron-withdrawing *N*-methylpyridinium group. To increase the solubility in organic solvents for photophysical and photochemical measurements as well as to avoid the reverse reaction due to iodide ion attacking the methyl group, the counterion was exchanged from iodide to hexafluorophosphate **(Scheme S2)**.

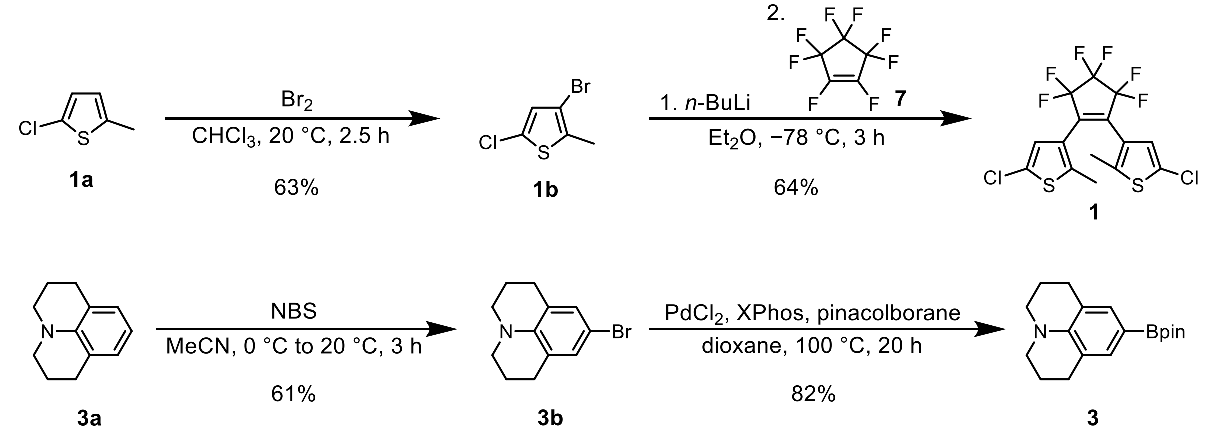

Scheme S1 Synthesis of core DTE dichloride **1** and julolidine boronic acid pinacol ester **3**.

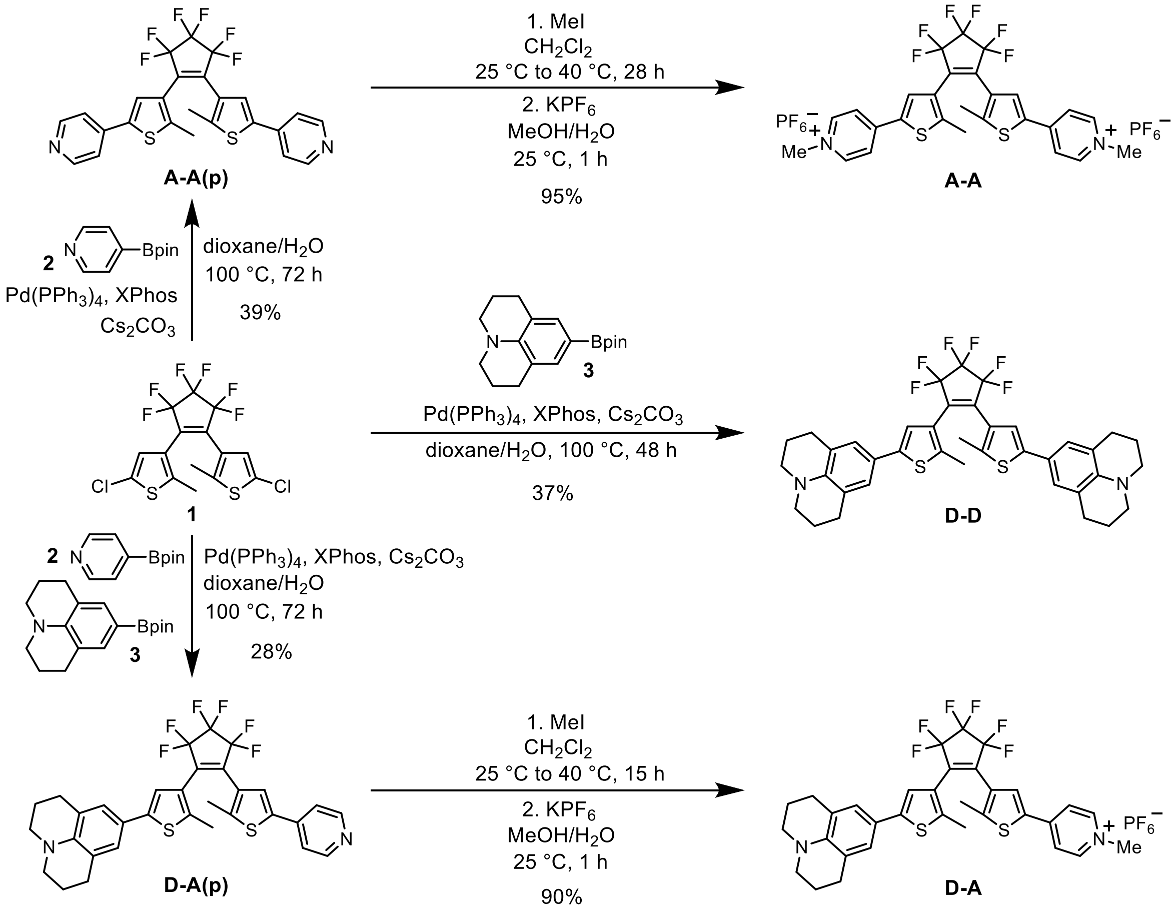

Scheme S2 Divergent core-to-arm synthetic route to **D-D**, **A-A** and **D-A** from DTE dichloride **1**.

Push-push **Dy-yD**, pull-pull **Ay-yA**, and push-pull **Dy-yA** with additional alkyne "arms" between the end functional group and the core thiophene were synthesized following the route shown in **Scheme S3** and **Scheme S4**. The synthetic route started with the formation of a TMS-protected DTE intermediate **11a** following an established procedure developed by Osuka and co-workers.[5] The TMS-protected compound **11b** was then deprotected with potassium carbonate to yield the key DTE intermediate **11** with two free terminal alkynes. The electron-rich Sonogashira-coupling partner **5** was synthesized by iodination of julolidine with iodine in 1:1 dioxane/pyridine solvent. The structure of **11** was extended by a Sonogashira coupling reaction with **5**, 4-iodopyridine **12**, or both in one pot, to form push-push **Dy-yD**, pull-pull **Ay-yA(p)**, and push-pull **Dy-yA(p)**, respectively. For **Ay-yA** and **Dy-yA**, the pyridine motif was methylated to generate an electron-withdrawing *N*-methylpyridinium group. A final ion-exchange from iodide to hexafluorophosphate led to better solubility in organic solvents for photophysical and photochemical analyses.

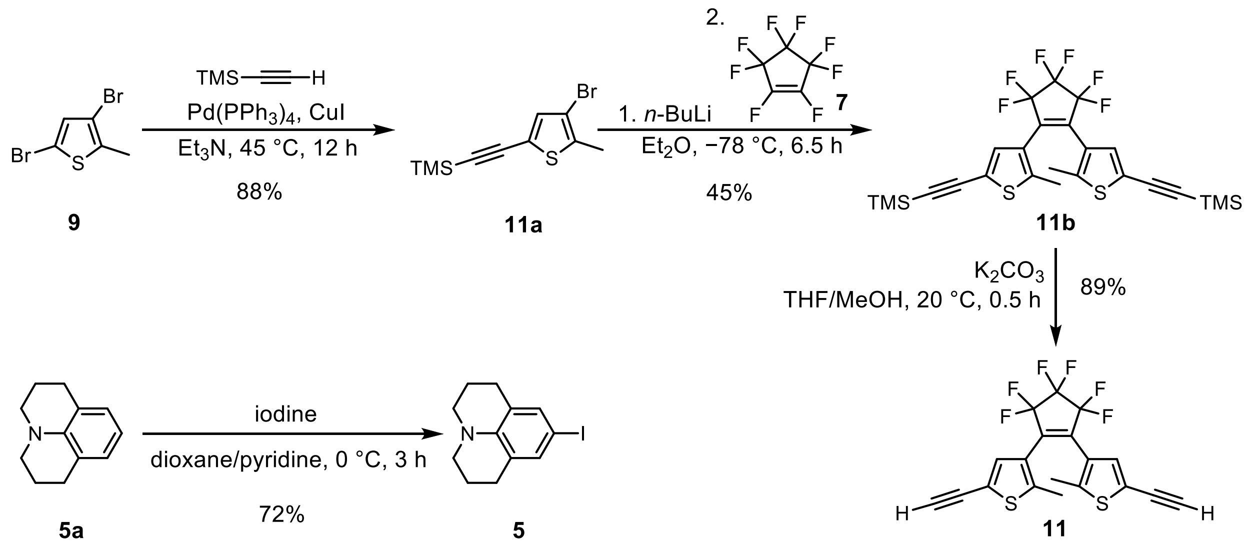

Scheme S3 Synthesis of DTE core **11** bearing two free terminal alkynes and iodination of julolidine.

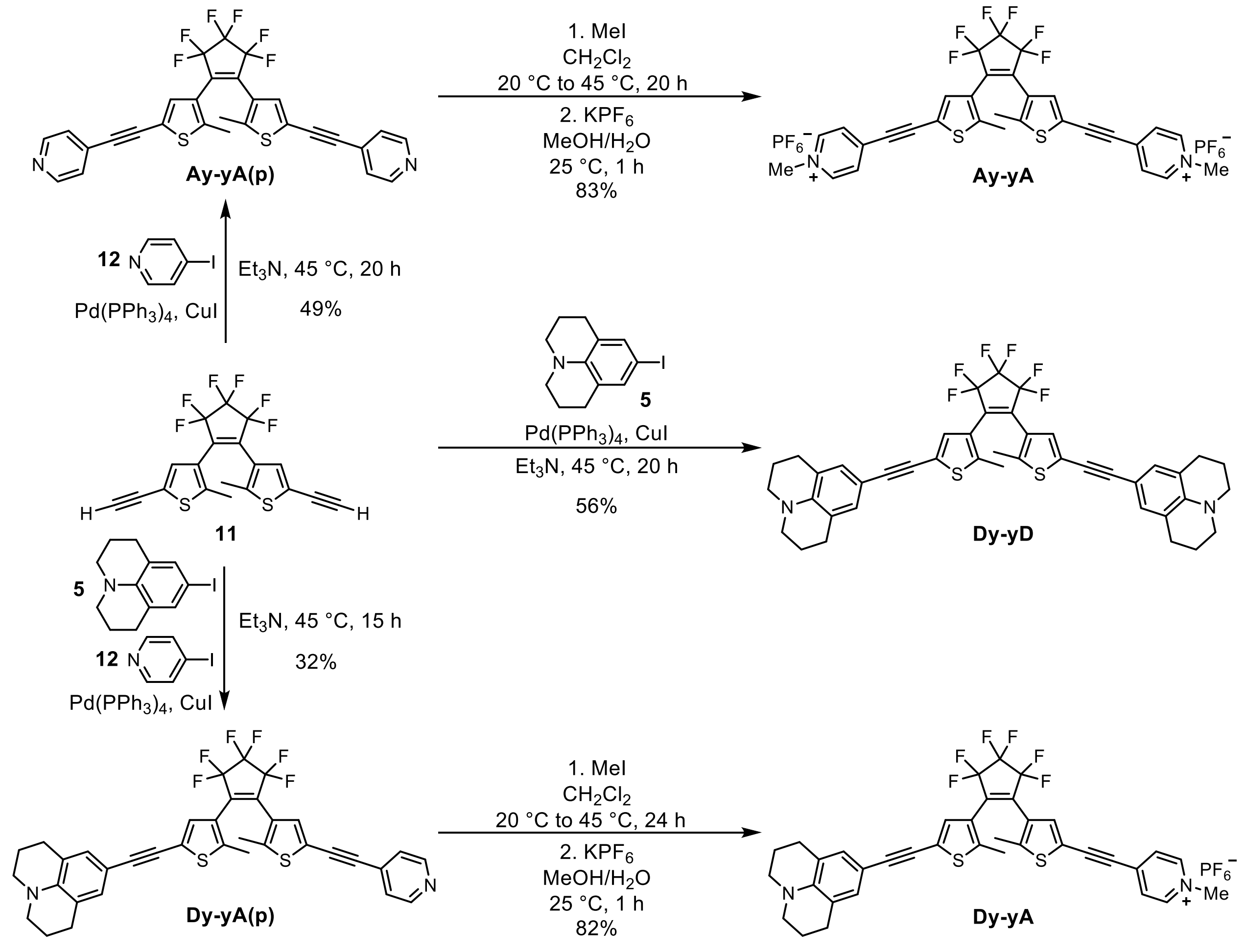

Scheme S4 Divergent core-to-arm synthesis of **Dy-yD**, **Ay-yA** and **Dy-yA** from DTE core **11**.

Due to the asymmetrical conjugation pattern about the central core, **Dy-A** and **D-yA** cannot be easily synthesized by building up from a core DTE by Suzuki or Sonogashira couplings. Therefore, as shown in **Scheme S5** and **Scheme S6**, routes starting with the functionalization of the arms followed by the construction of a photoswitchable DTE core at a later stage were adopted. For **Dy-A**, the electron-rich arm **6** and the electron-poor arm **10** were synthesized by Sonogashira and Suzuki couplings, respectively. The two arms were then attached to the perfluorinated cyclic bridge. Finally, the pyridine was methylated to yield an electron-withdrawing group. **D-yA** was constructed with a similar strategy starting with the functionalization of the arms and then constructing the photoswitchable core as shown in **Scheme S6**.

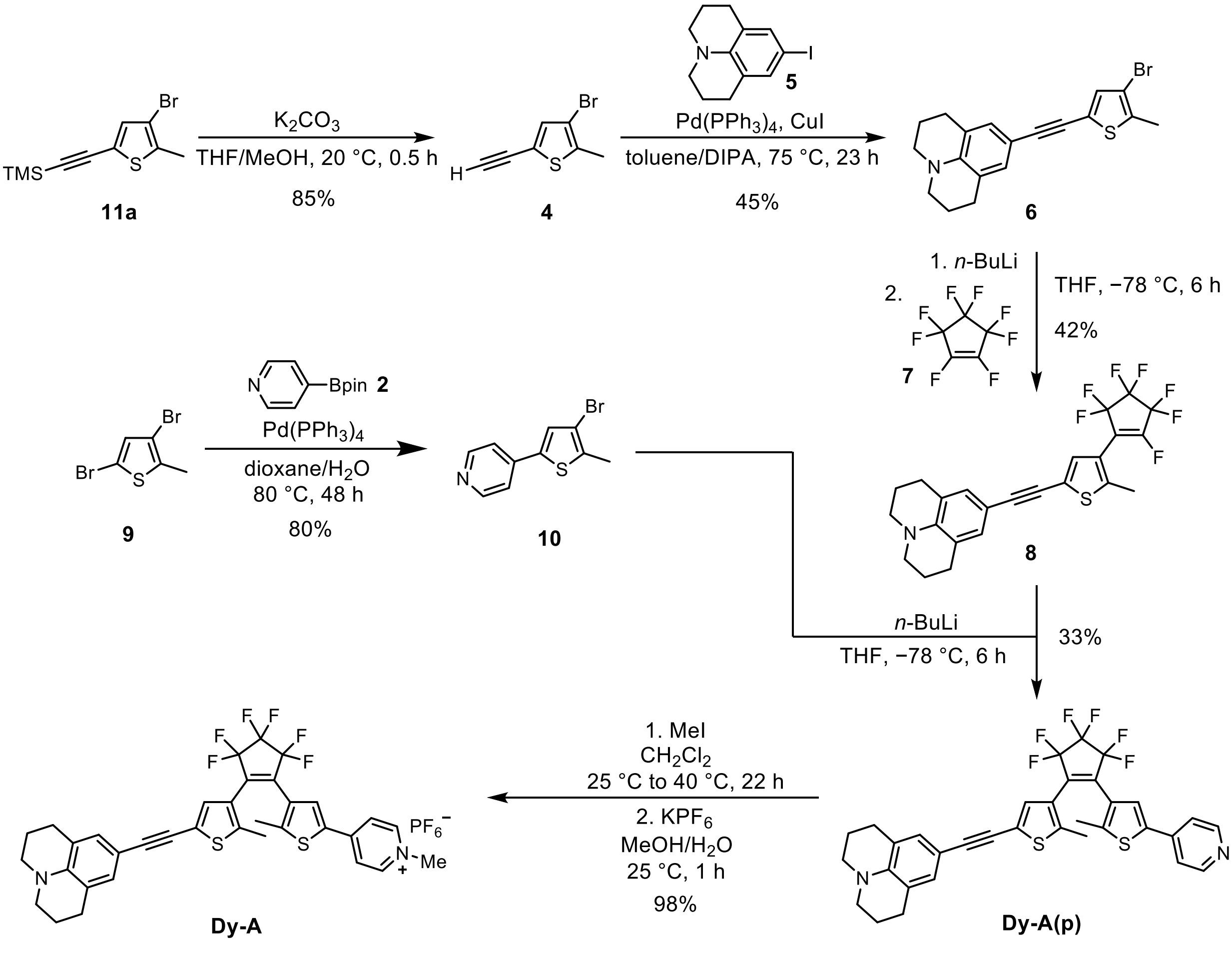

Scheme S5 Convergent synthetic route to **Dy-A**.

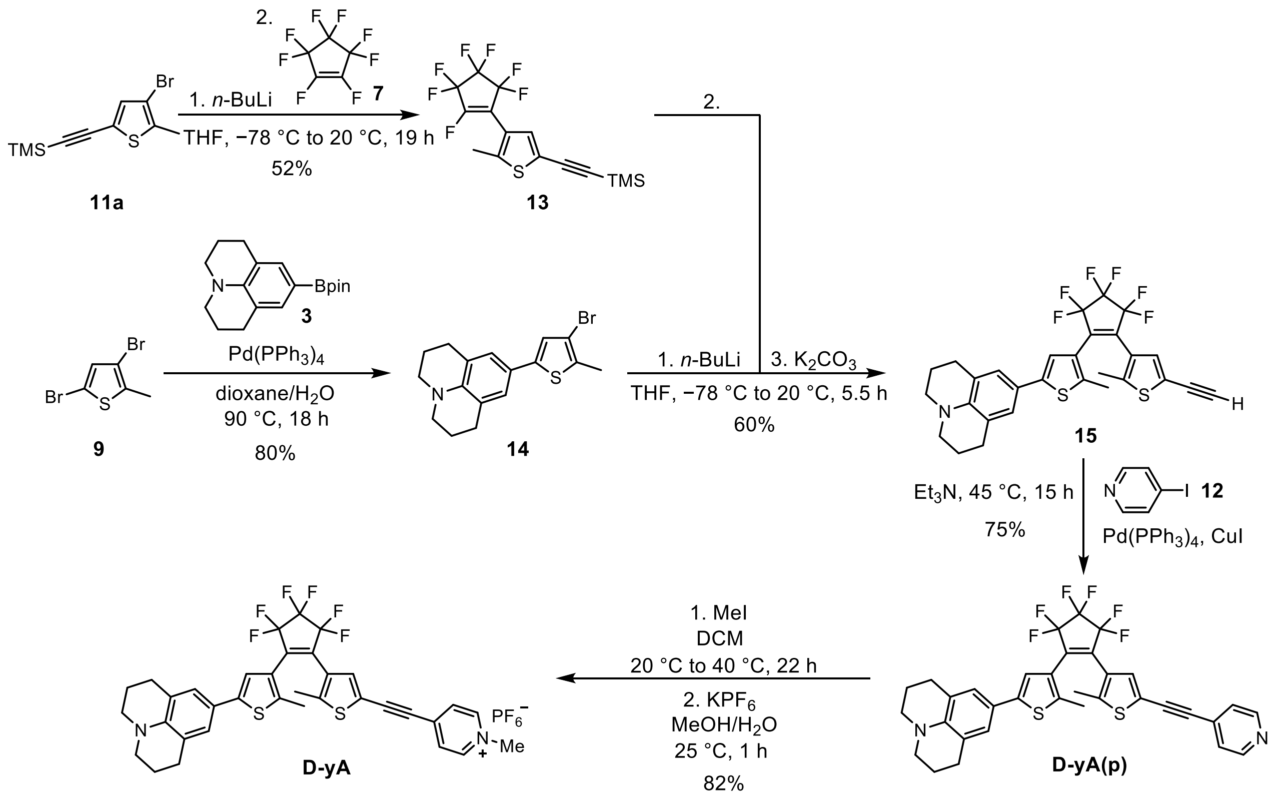

Scheme S6 Convergent synthetic route to **D-yA**.

To increase the water-solubility of **A-A** and **Ay-yA** and enable their photochemical and photophysical properties to be measured in water, the hexafluorophosphate counterion was exchanged to chloride to form **A-A** (chloride) and **Ay-yA** (chloride) following the procedure shown in **Scheme S7** reported by Pace and co-workers, which involves precipitation of the chloride salt from acetone.[6]

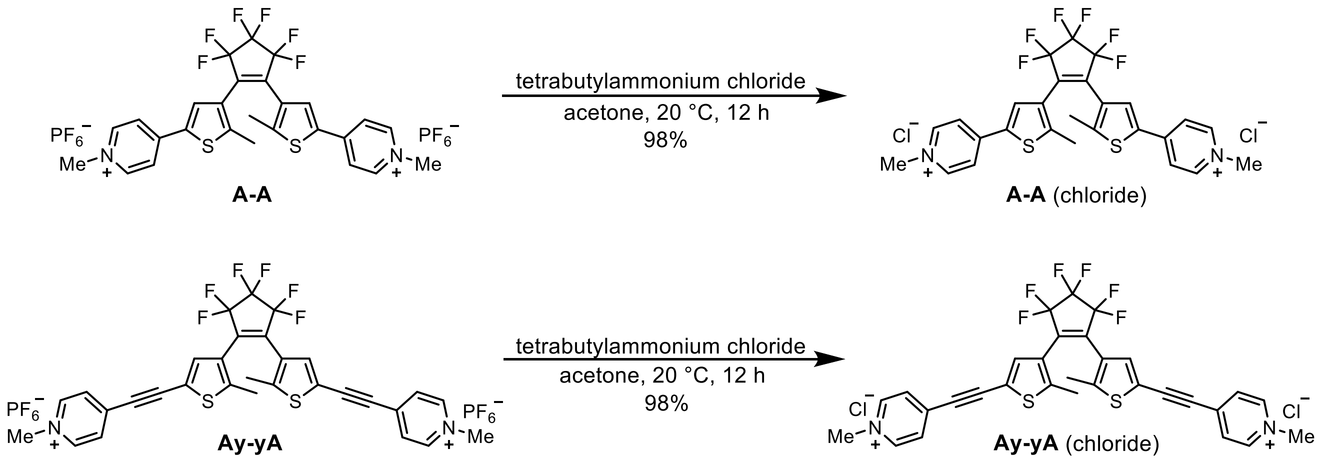

Scheme S7 Anion exchange to form **A-A** (chloride) and **Ay-yA** (chloride).

.

S3. Acquisition of the UV-vis Spectra of Open-Form, PSS and Closed-Form

A typical experiment is as follows: a stock solution of **D-D** in THF-d_8 of known concentration from NMR integration relative to a dioxane standard (as detailed in **Section S1**) was diluted with THF in a cuvette (3.0 mL, 4.2 μM) and then irradiated with 630 nm light (0.11 W, 1 min) to convert all residual closed-form **D-D** into open-form **D-D**. The open-form UV-vis spectrum was recorded between 280 nm and 1100 nm. The solution of **D-D** was then irradiated with cycles of 365 nm LED (18.9 mW, 1 s irradiation, 3 min interval) and the UV-vis spectrum was recorded during the intervals until no further changes in the UV-vis spectrum were observed. The final UV-vis spectrum with no further change after irradiation is the absorption spectrum of the photostationary state (PSS). The UV-vis spectrum of the closed-form **D-D** was then calculated based on the fact that the absorption spectrum of the PSS at a certain wavelength is a linear combination of open-form absorption and the closed-form absorption. The composition of **D-D** solution at PSS under 365 nm light irradiation was measured by ^{1}H NMR as detailed in **Section S6** and **Section S7**.

The photophysical and photochemical properties of each DTE were measured in two different solvents to test the impact of solvent polarity. For uncharged **D-D** and **Dy-yD**, and singly charged **D-A**, **Dy-yA**, **Dy-A** and **D-yA**, the UV-vis spectra were measured in THF and MeCN. The absorption spectra of doubly charged pull-pull **A-A** and **Ay-yA** were measured in MeCN but not THF due to poor solubility. After counterion exchange from hexafluorophosphate to chloride, the UV-vis spectra of **A-A** (chloride) and **Ay-yA** (chloride) were recorded in water.

The "switching" spectra of **D-D**, **D-A**, **Dy-yD**, **Dy-yA**, **Dy-A** and **D-yA** demonstrating the gradual change of the UV-vis spectra from the open-form to PSS upon 365 nm light irradiation shown in **Figure S3** (with the kinetic trace at specified wavelength shown in **Figure S4**) were acquired in THF due to higher photocyclization quantum yield of DTE (as detailed in **Section S5**) and higher conversion to the closed-form at PSS in less polar solvent (as detailed in **Section S6** and **Section S7**). Fast switching and high conversion at PSS lead to a clear emergence of the closed-form absorption band. The "switching" spectra of **A-A** and **Ay-yA** were measured in MeCN due to poor solubility in THF.

To compare the spectra of different compounds in the same solvent environment, the UV-vis spectra of the open-form, at PSS, and the closed-form of all compounds in MeCN are summarized in **Figure S5**. The UV-vis spectra of the open-form, at PSS, and the closed-form of **D-D**, **D-A**, **Dy-yD**, **Dy-yA**, **Dy-A**, and **D-yA** are summarized in **Figure S6**. The UV-vis spectra of the open-form, at PSS, and the closed-form of **A-A** (chloride) and **Ay-yA** (chloride) are summarized in **Figure S7**.

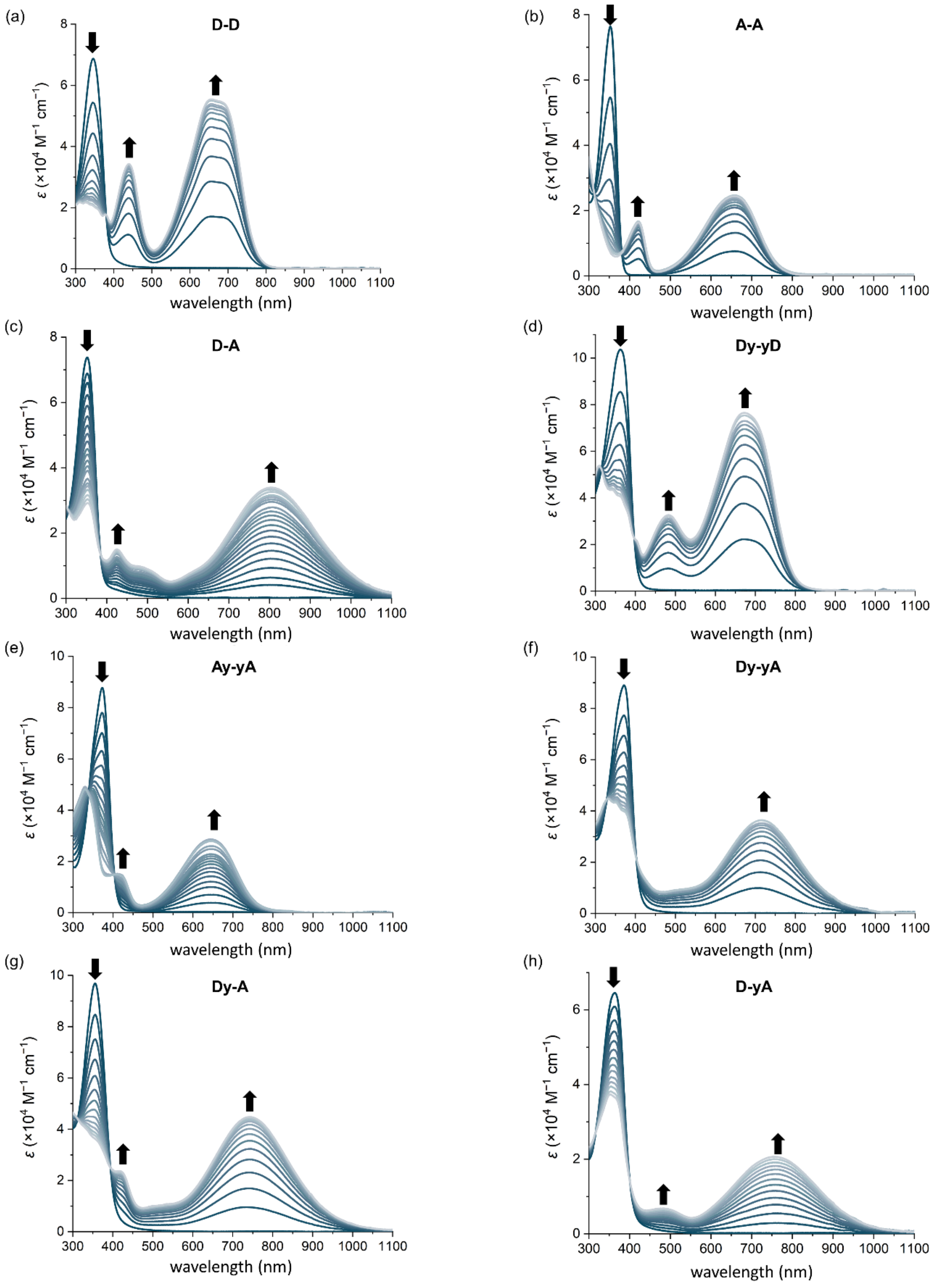

Figure S3 UV-vis-NIR spectra of DTEs showing the photocyclization from open-form to PSS upon 365 nm light irradiation at 25 °C. **D-D**, **D-A**, **Dy-yD**, **Dy-yA**, **Dy-A** and **D-yA** were measured in THF. **A-A** and **Ay-yA** were measured in **MeCN**. Each DTE was switched with a different power setting and irradiation time due to different rate of switching. The power and irradiation time between two consecutive spectra are summarized in **Table S1**.

Table S1 Irradiation power (365 nm) and irradiation time between two consecutive spectra for acquiring the spectra shown in **Figure S3**.

Compound	Solvent	Power (mW)	Irradiation Time (s)
**D-D**	THF	18.86	1
**A-A**	MeCN	12.33	1
**D-A**	THF	159.1	60
**Dy-yD**	THF	18.86	1
**Ay-yA**	MeCN	12.33	1
**Dy-yA**	THF	159.1	20
**Dy-A**	THF	159.1	20
**D-yA**	THF	159.1	30

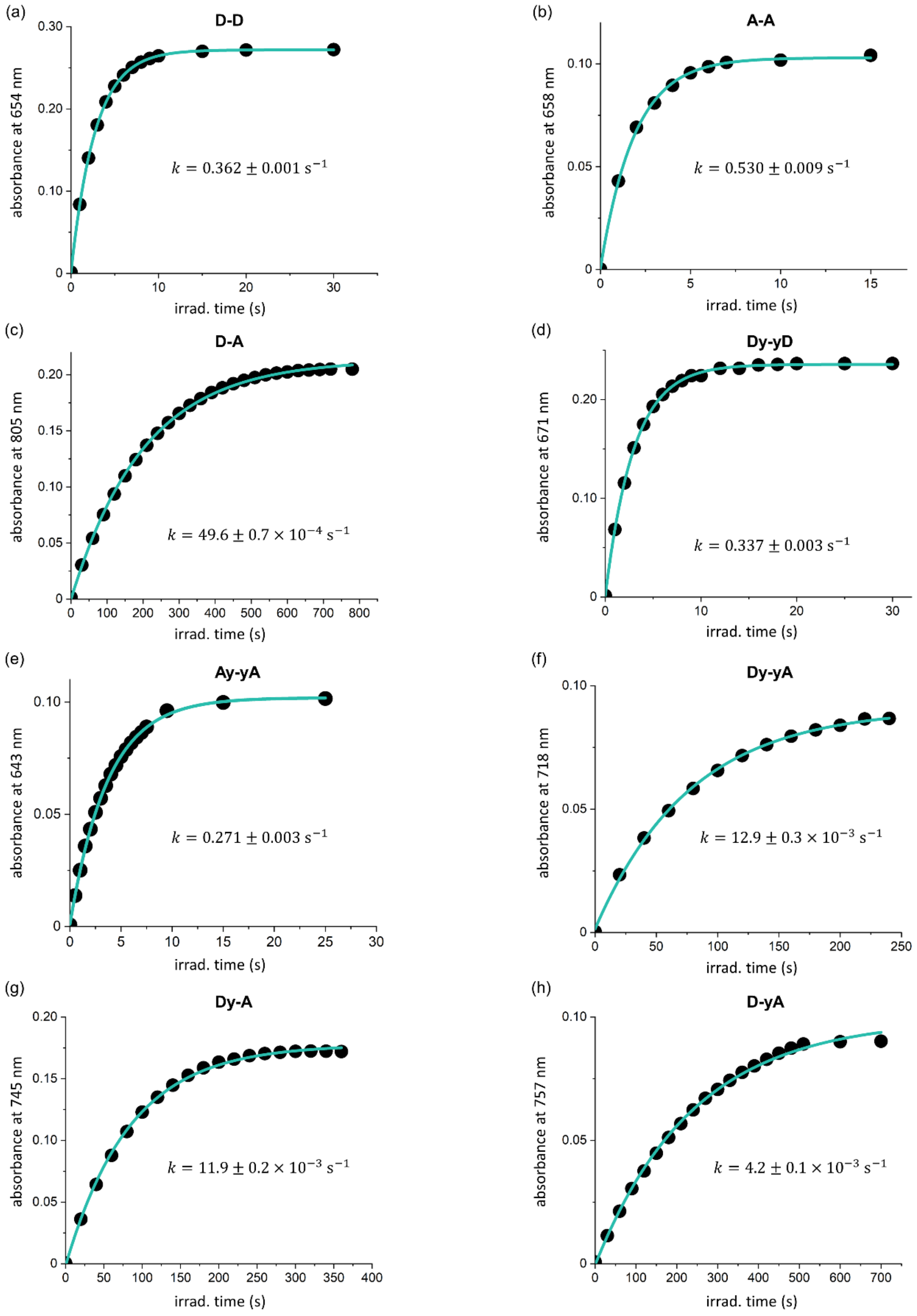

Figure S4 The kinetic trace of photocyclization (monitored at the absorption maxima of the closed-form) extracted from the UV-vis-NIR spectra shown in **Figure S3** and the fitted first-order curve with respect to time of irradiation.

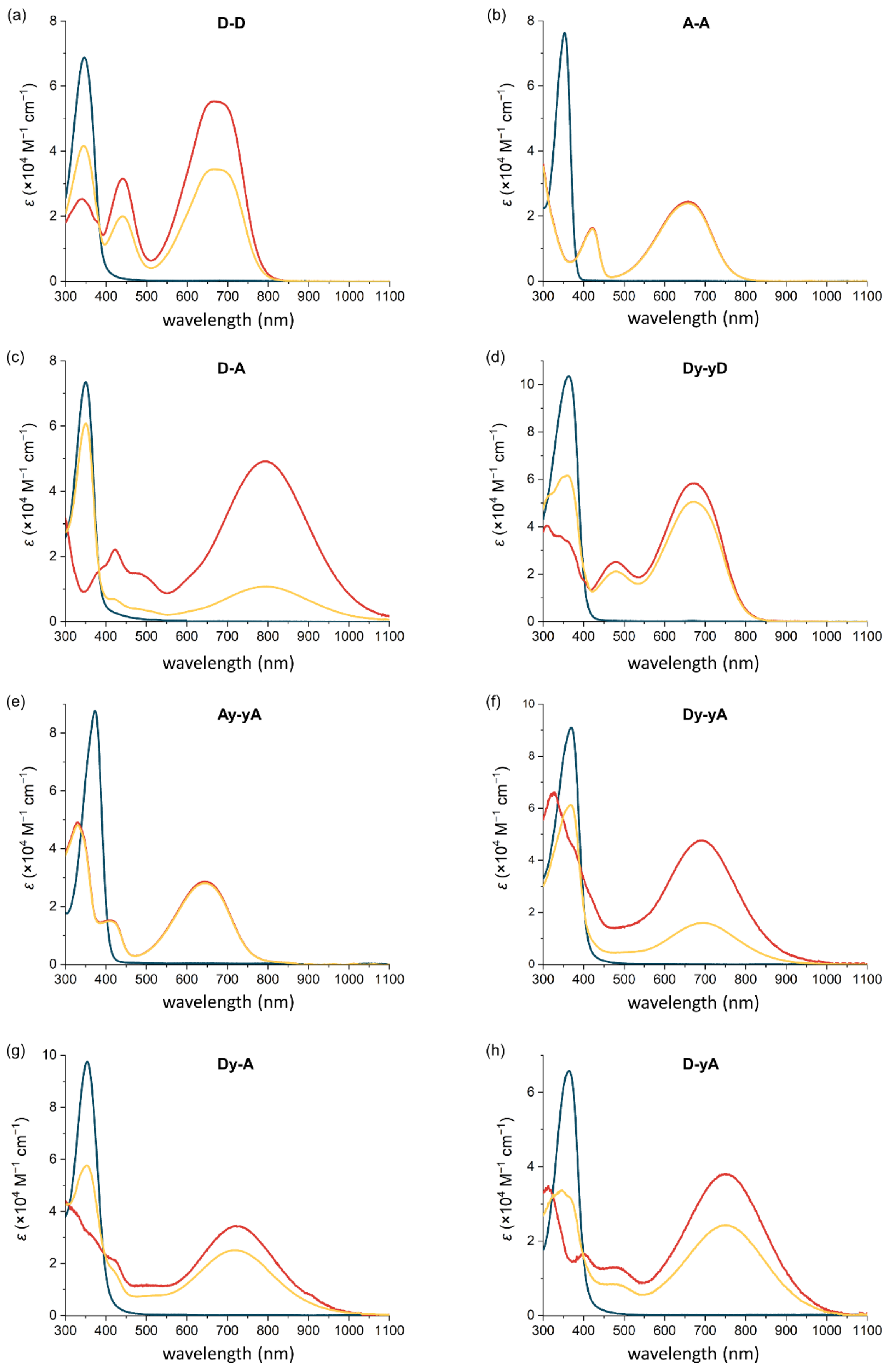

Figure S5 UV-vis-NIR absorption spectra of open-form (blue), PSS reached by 365 nm light irradiation (yellow), and closed-form calculated from the PSD obtained by NMR (red) of all synthesized DTEs measured in MeCN.

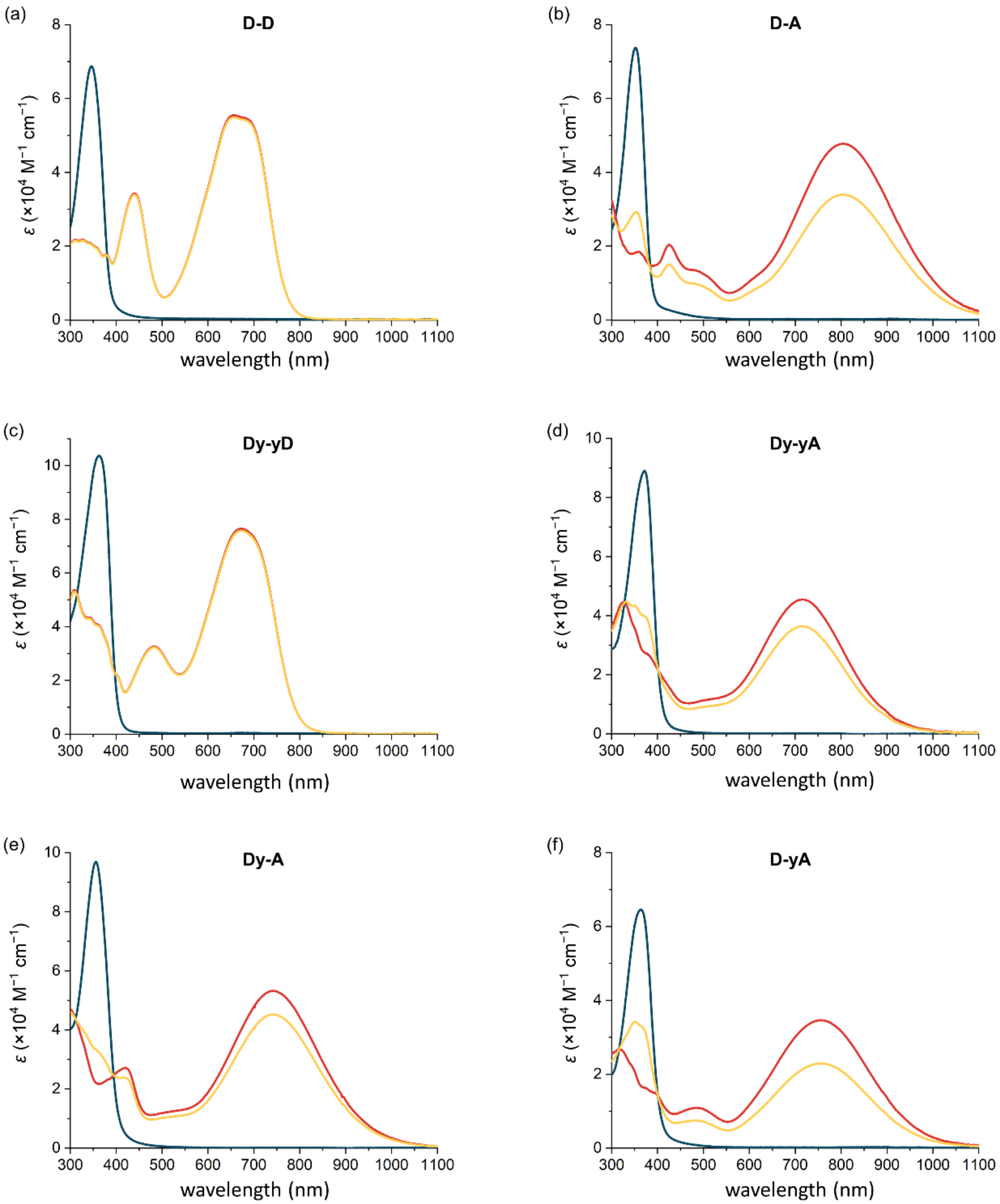

Figure S6 UV-vis-NIR absorption spectra of open-form (blue), PSS reached by 365 nm light irradiation (yellow), and closed-form calculated from the PSD obtained by NMR (red) of **D-D**, **D-A**, **Dy-yD**, **Dy-yA**, **Dy-A** and **D-yA** measured in THF.

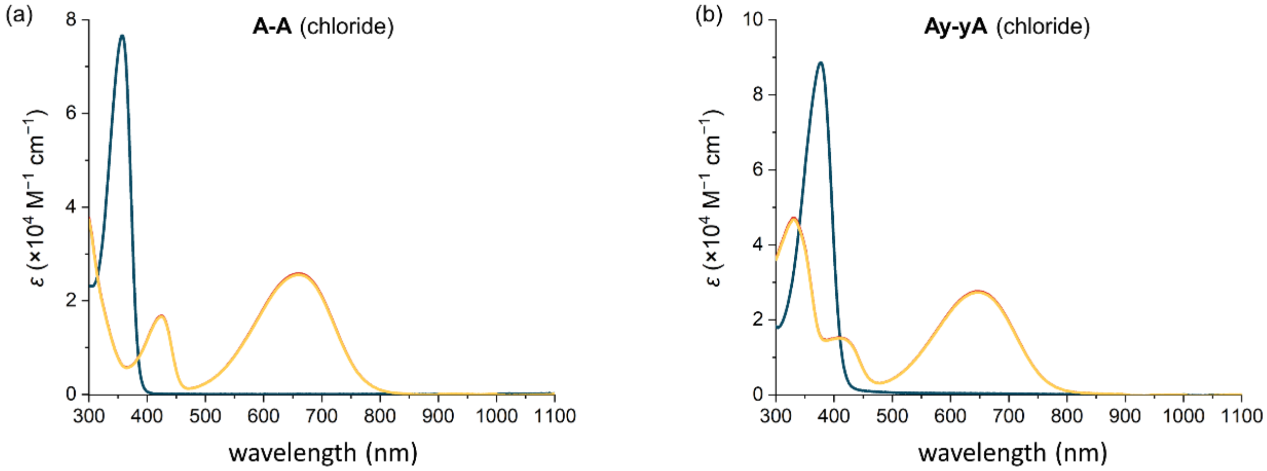

Figure S7 UV-vis-NIR absorption spectra of open-form (blue), PSS reached by 365 nm light irradiation (yellow), and closed-form calculated from the PSD obtained by NMR (red) of **A-A** (chloride) and **Ay-yA** (chloride) measured in water.

S4. Determination of Molar Absorption Coefficients

The concentration of the stock solution was determined by ^{1}H NMR spectroscopy by using a known concentration of dioxane as a reference. An NMR relaxation delay of 30 s was used to measure accurate integrals, as detailed in **Section S1**. A typical experiment for determining the molar absorption coefficient at the band maximum is as follows: a stock solution of **D-D** in THF-d_8 containing 2.24 mM dioxane was prepared and the exact concentration of **D-D** was determined by NMR to be 1.93 mM. The stock solution of **D-D** was diluted into THF and the absorption spectra of various concentrations were recorded following irradiation with 630 nm light at 112.0 mW for 5 minutes. The absorbance at 346 nm was plotted against concentration to give a graph of the form $y = mx$ where m gives the molar absorption coefficient according to the Beer-Lambert Law.

Table S2 Absorption maxima and molar absorption coefficients of open-form and closed-form DTEs in different solvents.

DTE	Solvent	λ_{max}^{o} (nm)	ε (10^3 M^{-1} cm^{-1})	λ_{max}^{c} (nm)	ε (10^3 M^{-1} cm^{-1})	λ_{max}^{c} (nm)	ε (10^3 M^{-1} cm^{-1})
**D-D**	THF	346	52.4	439	24.8	654	42.7
	MeCN	346	53.0	439	25.1	667	43.1
**A-A**[a]	MeCN	353	61.2	421	12.8	658	19.1
	water	357	61.5	424	12.7	659	19.0
**D-A**	THF	352	57.4	425	17.4	805	38.2
	MeCN	350	57.2	423	17.8	792	38.1
**Dy-yD**	THF	362	85.1	483	26.7	671	47.3
	MeCN	363	84.8	479	27.4	673	41.9
**Ay-yA**[a]	MeCN	371	67.1	411	11.8	643	22.3
	water	377	69.5	415	11.6	650	23.1
**Dy-yA**	THF	371	71.0	/	/	718	37.8
	MeCN	369	72.4	/	/	693	37.6
**Dy-A**	THF	356	76.1	418	20.2	745	29.4
	MeCN	354	74.2	417	16.6	724	26.5
**D-yA**	THF	364	58.6	484	10.8	757	32.3
	MeCN	364	55.7	484	10.4	753	35.1

[a] Measured in MeCN as the hexafluorophosphate salt and in water as the chloride salt.

S5. Determination of Photocyclization and Photoreversion Quantum Yields

Quantum yields of photocyclization and photoreversion were measured using the "initial slope" method in the low-concentration regime[1] with the experimental setup reported by Stadler and co-workers.[7] The photochemical quantum yield was calculated using **Equation S1** as reported by Heckel and co-workers,[8] which is a general method for measuring the photochemical quantum yield of P-type (thermally irreversible) photoswitches, where φ is the photochemical quantum yield of the reaction, m is the slope of the linear fit for the time-dependent absorbance change during irradiation for appearance or disappearance of closed-form DTE monitored at the band maxima in the visible region (e.g. monitored at 654 nm for **D-D**), V is the volume of solution under irradiation, N_A is the Avogadro constant, h is the Planck constant, c is the speed of light, P is the irradiation power (in W), λ is the excitation wavelength, A is the absorbance at excitation wavelength, ε_{prod} is the molar absorption coefficient of the formed product at the monitoring wavelength (e.g. at 654 nm for **D-D**), l is the pathlength (1 cm).

$$\varphi = \frac{m \cdot V \cdot N_A \cdot h \cdot c}{P \cdot \lambda \cdot (1 - 10^{-A}) \cdot \varepsilon_{prod} \cdot l} \quad \textbf{(S1)}$$

The powers, P, of the mic-LED-365, mic-LED-405 and mic-LED-630 were first measured with a Coherent® PowerMaxTM-USB/RS Sensor System power meter at various points of indicated power percentage **(Table S3)**. These measurements were carried out at the end of the liquid light guide, immediately before the cuvette, so they give a measure of the light being delivered to the sample.

Table S3 Power of LED light sources measured from a Coherent® PowerMaxTM-USB/RS Sensor System.

Indicated Power Percentage (%)	Power of mic-LED-365 (mW)	Power of mic-LED-405 (mW)	Power of mic-LED-630 (mW)
2	10.4	12.1	7.17
5	16.8	18.6	10.9
10	27.6	29.7	17.3
20	47.2	50.5	28.8
30	68.3	71.1	39.8
40	88.5	90.3	51.7
50	107	109	63.4
60	127	127	75.3
70	147	144	86.5
80	/	160	97.8
90	/	176	108
100	/	190	118

The power of the mic-LED-365 and mic-LED-405 light sources was also confirmed by a chemical actinometry method as described below.[29,30]

Ferric oxalate actinometry: A solution of ferric oxalate (0.0060 M) was prepared by dissolving $K_3[Fe(C_2O_4)_3]\cdot 3H_2O$ (300 mg, 0.60 mmol) in aqueous sulfuric acid (100 mL, 0.050 M). From this stock solution, 2.00 mL of it was transferred to a quartz cuvette, and while stirring, irradiated with mic-LED-365 at the power of the indicated output percentage for 10 s, while an identical control sample was kept in the dark. A sample of the irradiated ferric oxalate sodium (0.20 mL) and a control sample (0.20 mL) were transferred to vials containing a mixture of 0.1% phenanthroline solution (0.90 mL, prepared by dissolving 13.56 g CH_3CO_2Na and 0.100 g of phenanthroline in 100 mL of 0.50 M H_2SO_4) and H_2O (0.90 mL). Samples were kept in the dark for 1 h to allow the complexation to occur and the absorption spectra were acquired in the 250–850 nm range. The LED power intensities and irradiation lengths were chosen to ensure that the photoconversion of ferric oxalate does not exceed 5% and that the absorbance of $Fe(phen)_3^{2+}$ complex is within the range 0.5–1.0.

Upon exposure to light, $K_3[Fe(C_2O_4)_3]$ is converted to Fe^{2+}, and the quantum yield of the formation of Fe^{2+} (Φ_R) is well-known for a range of wavelengths (220–500 nm, Φ_R = 1.16 at 365 nm, Φ_R = 1.14 at 405 nm). The photon flux f was calculated using the equation S2, where Φ_R is the quantum yield of the ferric oxalate conversion at the irradiation wavelength, t is the irradiation time in seconds, $(1-10^{-A})$ is the fraction of light absorbed by the ferric oxalate solution while A is the absorbance of 0.006 M solution at the irradiation wavelength. Formation of the coloured $Fe(phen)_3^{2+}$complex (ε_{510} = 11,100 M^{-1} cm^{-1}) allows quantification of Fe^{2+} generated during the irradiation by measuring the absorbance of the irradiated and non-irradiated samples (Equation S3), where V_1 (mL) is the irradiated volume, V_2 (mL) is the aliquot of the irradiated solution being transferred to the solution of buffered phenanthroline, V_3 (mL) is the final volume of the complexation solution , l (cm) is the optical path length of the quartz cuvette used for irradiation, ΔA is the difference in the absorbance between the irradiated solution and the control stored in dark, and ε (510 nm) is the molar absorption coefficient of $Fe(phen)_3^{2+}$ complex at 510 nm.

$$f = \frac{\text{no. of moles of Fe}^{2+}}{\Phi_\text{R} \cdot t \cdot (1 - 10^{-A})} \qquad \textbf{(S2)}$$

$$\text{no. of moles of Fe}^{2+} = \frac{V_1 \cdot V_3 \cdot \Delta A\ (510\ \text{nm})}{10^3 \cdot V_2 \cdot l \cdot \varepsilon} \qquad \textbf{(S3)}$$

The calculated value of the photon flux was expressed in $E\cdot s^{-1}$ and was converted to mW using equation S4.

$$P(\text{mW}) = 1000 \cdot f\ (\text{E} \cdot \text{s}^{-1}) \cdot N_\text{A} \cdot h \cdot \frac{c}{\lambda} \qquad \textbf{(S4)}$$

A linear correlation between the power (mW) and the indicated output percentage was derived (**Figure S8**). The power obtained from the power meter matches well with that from the chemical actinometry measurement.

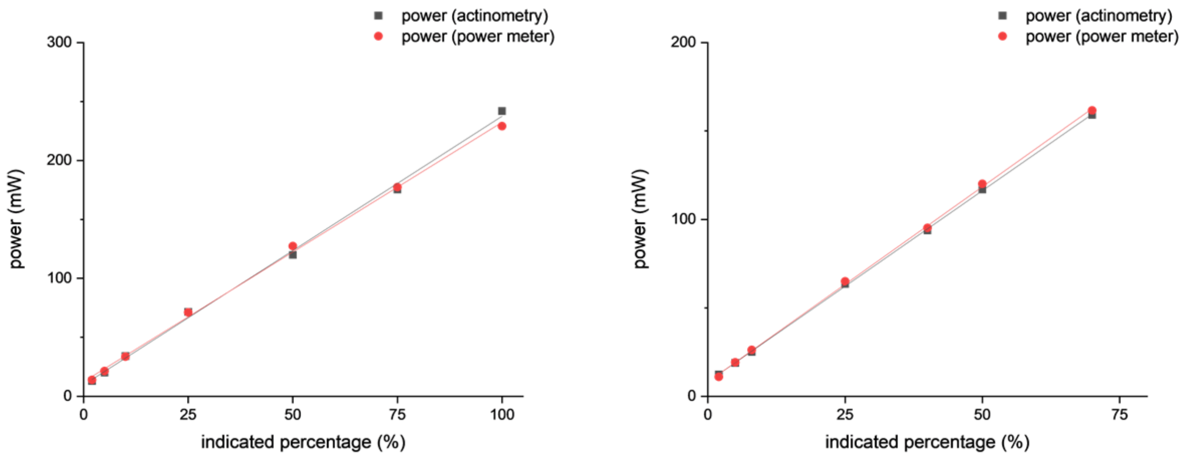

Figure S8 Linear correlation between the output percentage and power from actinometry or the power meter for mic-LED-365 light source (left) and mic-LED-405 light source (right.). For mic-LED-365 light source, the measurement points from actinometry are shown as black squares (linear fit: black line, $y = 2.16x + 8.19$, $R^2 = 0.998$). Measurement points from the power meter are shown as red circles (linear fit: red line, $y = 2.21x + 8.10$, $R^2 = 0.998$). For mic-LED-405 light source, the measurement points from actinometry are shown as black squares (linear fit: black line, $y = 2.29x + 9.64$, $R^2 = 0.997$). Measurement points from the power meter are shown as red circles (linear fit: red line, $y = 2.20x + 12.4$, $R^2 = 0.998$).

To accurately determine the initial slope (*m*) of the photochemical process, the power of the LED light source was turned down sufficiently that the formation of the product is linear (only 5%–10% of the product is formed during the process of measurement). A typical photocyclization experiment is as follows: a THF solution of **D-D** (1.6 μM) was placed in a cuvette in the sample holder of the UV-vis spectrometer and stirred at 25 °C. After irradiating the sample with 365 nm light (18.9 mW, 200 ms irradiation), the absorption intensity at 654 nm corresponding to peak maximum of the formed product was recorded. The irradiation-recording process was repeated for five cycles to acquire a kinetic trace of the photochemical conversion (8% of the open-form **D-D** was converted to the closed-form). The determination of the initial slope was carried out twice for each compound in each solvent, and showed good reproducibility.

Typical results are shown in **Figures S9–16**. The variables involved in calculating the photochemical quantum yield of each compound are listed in **Table S4**. The gradients of linear fits and the photochemical quantum yields of all the DTEs are listed in **Table S5**.

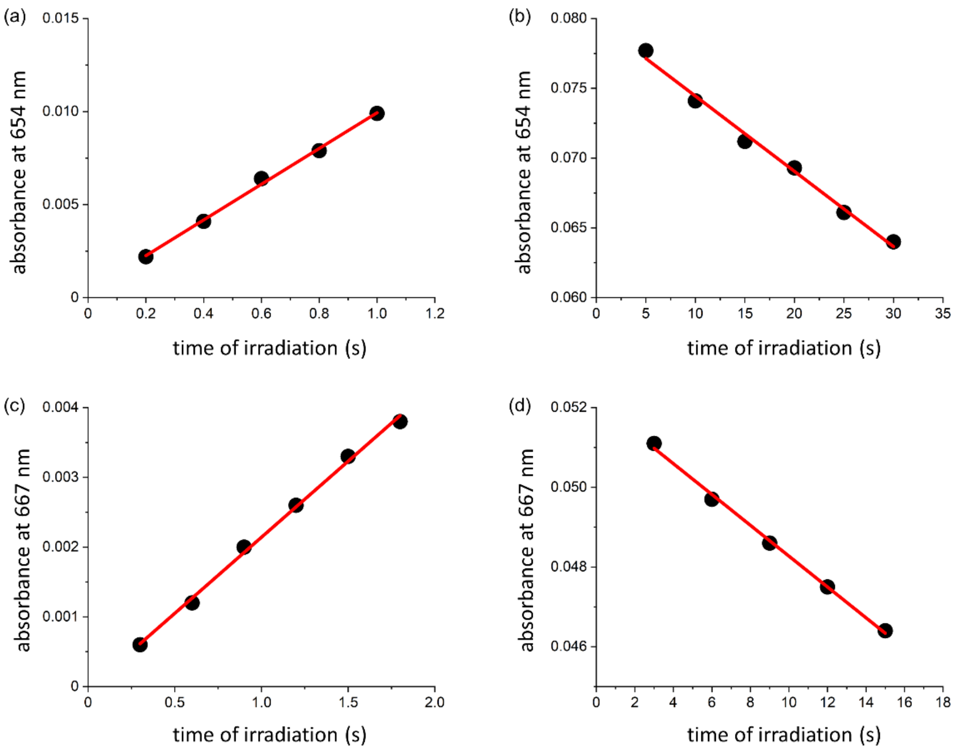

Figure S9 The initial region of the kinetic trace of (a) photocyclization and (b) photoreversion in THF, and (c) photocyclization and (d) photoreversion in MeCN of **D-D** and their linear fits. The gradient m of (a) 0.0100 ± 0.0004 s^{-1}, (b) 0.00057 ± 0.00002 s^{-1}, (c) 0.00222 ± 0.00005 s^{-1} and (d) 0.00036 ± 0.00002 s^{-1} were used in the calculation of photochemical quantum yields.

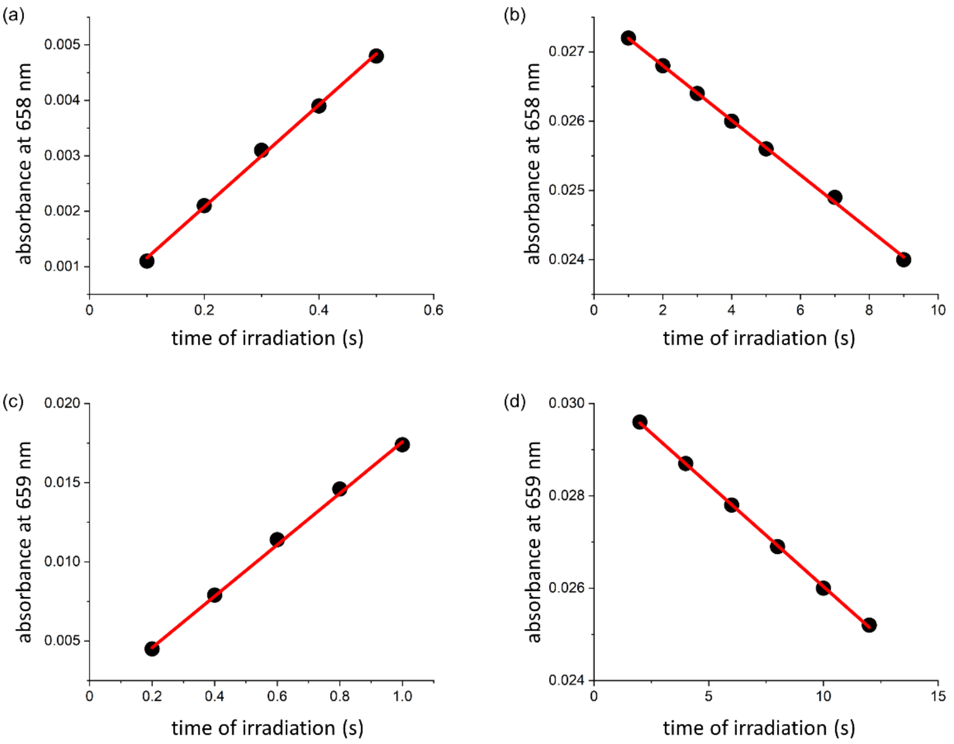

Figure S10 The initial region of the kinetic trace of (a) photocyclization and (b) photoreversion in MeCN of **A-A** as hexafluorophosphate salt, and (c) photocyclization and (d) photoreversion in water of **A-A** as chloride salt and their linear fits. The gradient m of (a) 0.0183 ± 0.0004 s^{-1}, (b) 0.00050 ± 0.00002 s^{-1}, (c) 0.030 ± 0.002 s^{-1} and (d) 0.00043 ± 0.00001 s^{-1} were used in the calculation of photochemical quantum yields.

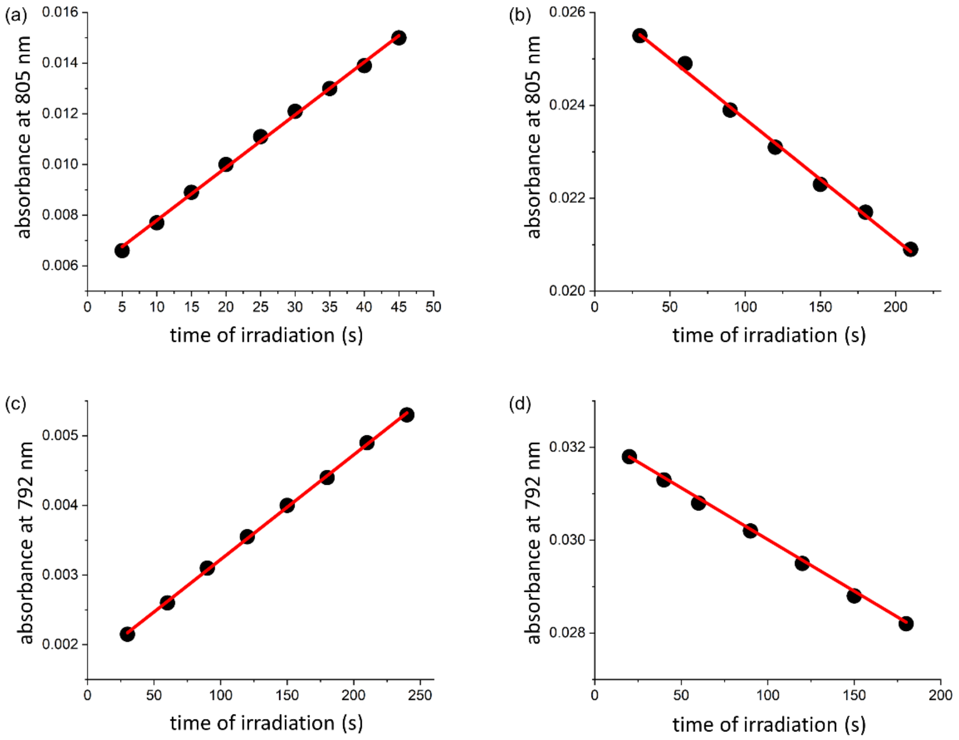

Figure S11 The initial region of the kinetic trace of (a) photocyclization and (b) photoreversion in THF, and (c) photocyclization and (d) photoreversion in MeCN of **D-A** and their linear fits. The gradient m of (a) 0.00021 ± 0.00004 s^{-1}, (b) 0.000026 ± 0.000001 s^{-1}, (c) 0.000015 ± 0.000001 s^{-1} and (d) 0.000023 ± 0.00001 s^{-1} were used in the calculation of photochemical quantum yields.

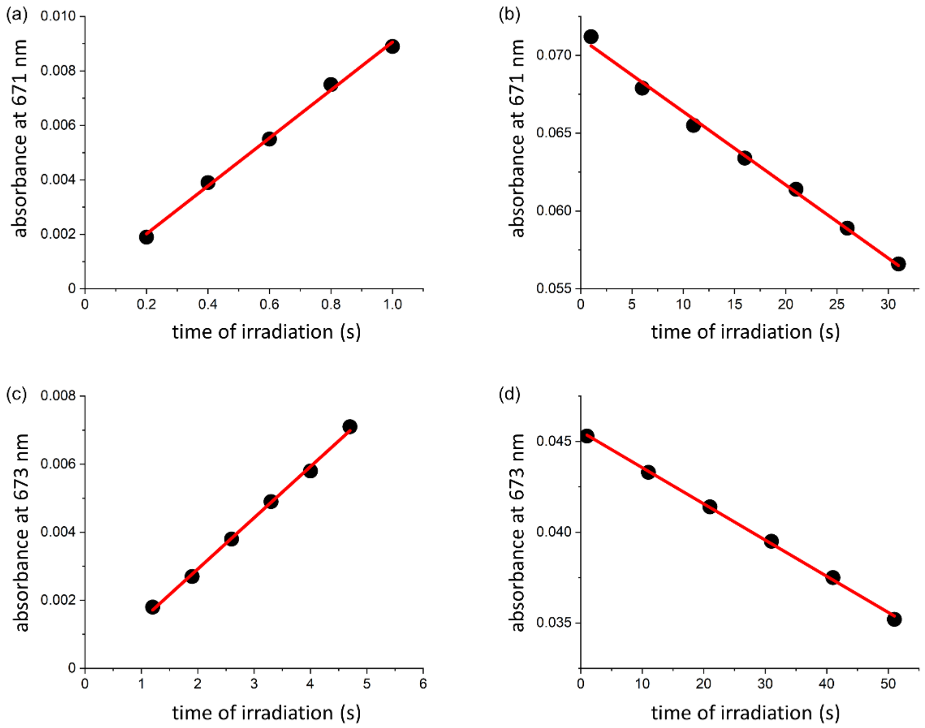

Figure S12 The initial region of the kinetic trace of (a) photocyclization and (b) photoreversion in THF, and (c) photocyclization and (d) photoreversion in MeCN of **Dy-yD** and their linear fits. The gradient m of (a) 0.0090 ± 0.0002 s^{-1}, (b) 0.00047 ± 0.00002 s^{-1}, (c) 0.00142 ± 0.00003 s^{-1} and (d) 0.00019 ± 0.00001 s^{-1} were used in the calculation of photochemical quantum yields.

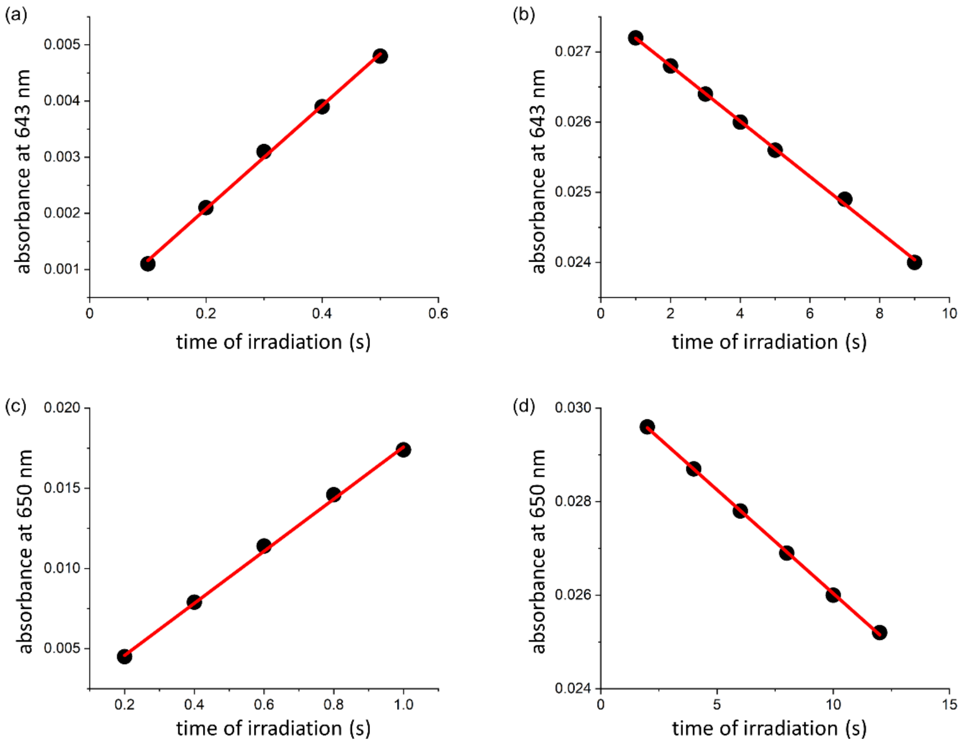

Figure S13 The initial region of the kinetic trace of (a) photocyclization and (b) photoreversion in MeCN of **Ay-yA** as hexafluorophosphate salt, and (c) photocyclization and (d) photoreversion in water of **Ay-yA** as chloride salt and their linear fits. The gradient *m* of (a) 0.0095 ± 0.0002 s^{-1}, (b) 0.00040 ± 0.00001 s^{-1}, (c) 0.0172 ± 0.0007 s^{-1} and (d) 0.00045 ± 0.00001 s^{-1} were used in the calculation of photochemical quantum yields.

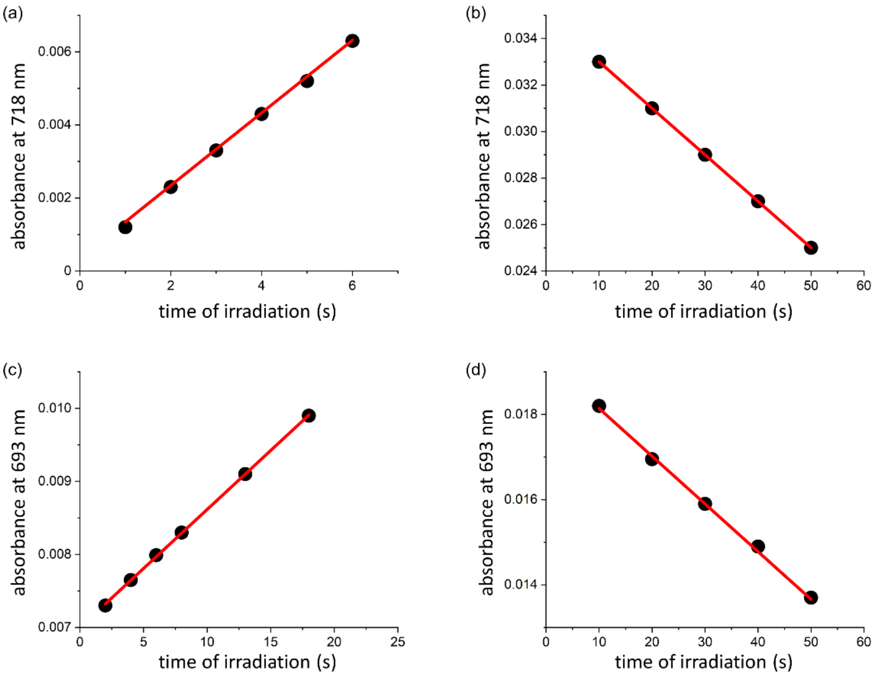

Figure S14 The initial region of the kinetic trace of (a) photocyclization and (b) photoreversion in THF, and (c) photocyclization and (d) photoreversion in MeCN of **Dy-yA** and their linear fits. The gradient *m* of (a) 0.0010 ± 0.0001 s^{-1}, (b) 0.00017 ± 0.00001 s^{-1}, (c) 0.00018 ± 0.00002 s^{-1} and (d) 0.00013 ± 0.00001 s^{-1} were used in the calculation of photochemical quantum yields.

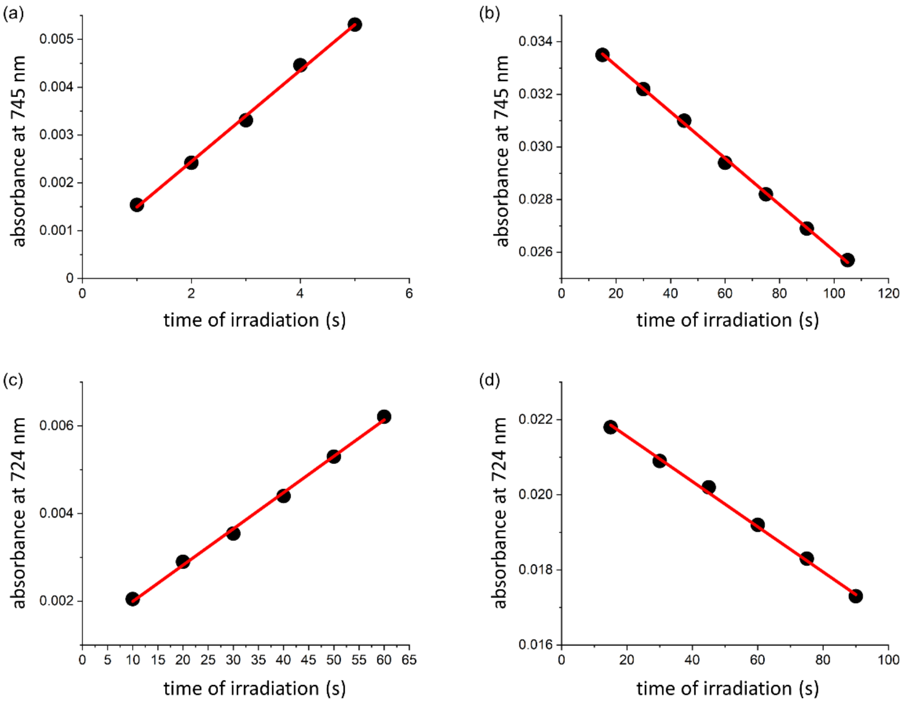

Figure S15 The initial region of the kinetic trace of (a) photocyclization and (b) photoreversion in THF, and (c) photocyclization and (d) photoreversion in MeCN of **Dy-A** and their linear fits. The gradient *m* of (a) 0.00077 ± 0.00007 s^{-1}, (b) 0.000094 ± 0.000005 s^{-1}, (c) 0.000086 ± 0.000007 s^{-1} and (d) 0.000059 ± 0.000003 s^{-1} were used in the calculation of photochemical quantum yields.

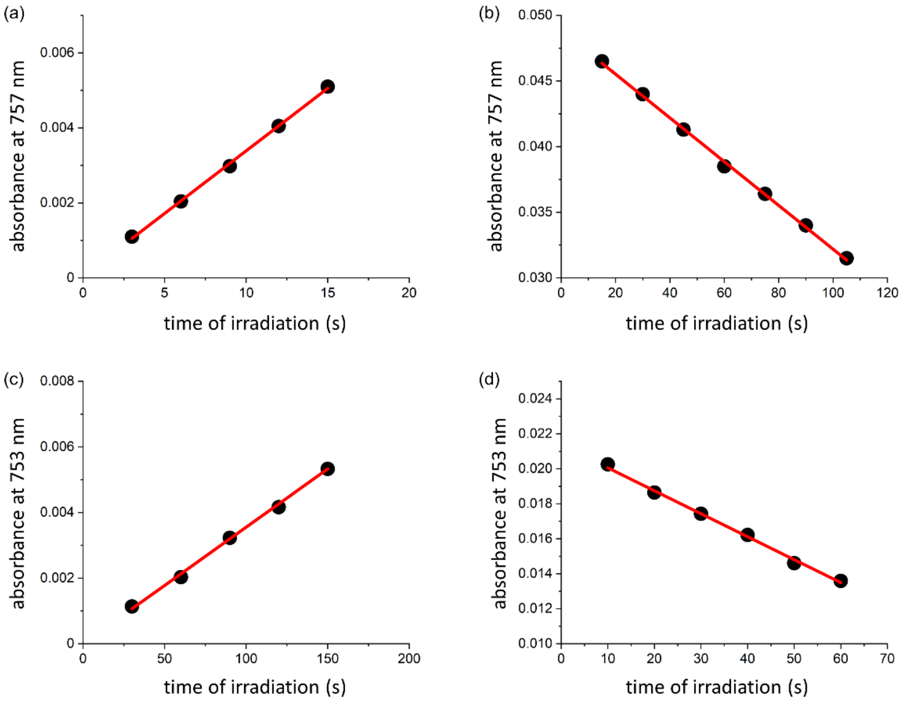

Figure S16 The initial region of the kinetic trace of (a) photocyclization and (b) photoreversion in THF, and (c) photocyclization and (d) photoreversion in MeCN of **D-yA** and their linear fits. The gradient *m* of (a) 0.00025 ± 0.00002 s^{-1} and (b) 0.00017 ± 0.00001 s^{-1}, (c) 0.000036 ± 0.000002 s^{-1} and (d) 0.00013 ± 0.00001 s^{-1} were used in the calculation of photochemical quantum yields.

Table S4 Absorbance at excitation wavelength (365 nm and 630 nm), power of LED, wavelength of irradiation (365 nm and 630 nm), and molar absorption coefficient at monitoring wavelength used for calculating the photochemical quantum yield of each compound in different solvents.

DTE	Solvent	Process	A	P /W	exci. λ /nm	moni. λ /nm	ε_{prod} /M^{-1} cm^{-1}
**D-D**	THF	O-C	0.0749	0.0189	365	654	42700
		C-O	0.0754	0.1120	630		
	MeCN	O-C	0.0134	0.1591	365	667	43100
		C-O	0.0504	0.1120	630		
**A-A**	MeCN	O-C	0.0743	0.0189	365	658	19100
		C-O	0.0374	0.1120	630		
	water	O-C	0.1068	0.0189	365	659	19000
		C-O	0.0319	0.1120	630		
**D-A**	THF	O-C	0.0803	0.1591	365	805	38200
		C-O	0.0098	0.1120	630		
	MeCN	O-C	0.1004	0.1591	365	792	38100
		C-O	0.0102	0.1120	630		
**Dy-yD**	THF	O-C	0.0887	0.0189	365	671	47300
		C-O	0.0930	0.1120	630		
	MeCN	O-C	0.0878	0.1591	365	673	41900
		C-O	0.0401	0.1120	630		
**Ay-yA**	MeCN	O-C	0.0978	0.0189	365	643	22300
		C-O	0.0323	0.1120	630		
	water	O-C	0.1089	0.0189	365	650	23100
		C-O	0.0372	0.1120	630		
**Dy-yA**	THF	O-C	0.1024	0.1591	365	718	37800
		C-O	0.0259	0.1120	630		
	MeCN	O-C	0.1209	0.1591	365	693	37600
		C-O	0.0199	0.1120	630		
**Dy-A**	THF	O-C	0.1393	0.1591	365	745	29400
		C-O	0.0176	0.1120	630		
	MeCN	O-C	0.0632	0.1591	365	724	26500
		C-O	0.0115	0.1120	630		
**D-yA**	THF	O-C	0.8092	0.1591	365	757	32300
		C-O	0.0254	0.1120	630		
	MeCN	O-C	0.05836	0.1591	365	753	35100
		C-O	0.02147	0.1120	630		

Table S5 Summary of quantum yields of photocyclization and photoreversion measured by the initial slope method.

Compound	Solvent	m_{o-c}	φ_{o-c} / %	m_{c-o}	φ_{c-o} / %
**D-D**	THF	100 ± 4 ×10^{-4} s^{-1}	7.7 ± 0.4	5.7 ± 0.2 ×10^{-4} s^{-1}	0.043 ± 0.002
	MeCN	22.2 ± 4 ×10^{-4} s^{-1}	0.18 ± 0.01	3.6 ± 0.2 ×10^{-4} s^{-1}	0.039 ± 0.001
**A-A**[a]	MeCN	183 ± 4 ×10^{-4} s^{-1}	31 ± 2	5.0 ± 0.2 ×10^{-4} s^{-1}	0.16 ± 0.01
	water	300 ± 20 ×10^{-4} s^{-1}	38 ± 3	4.3 ± 0.1 ×10^{-4} s^{-1}	0.16 ± 0.01
**D-A**	THF	2.1 ± 0.1 ×10^{-4} s^{-1}	0.020 ± 0.001	2.6 ± 0.4 ×10^{-5} s^{-1}	0.015 ± 0.002
	MeCN	1.5 ± 0.1 ×10^{-5} s^{-1}	1.2 ± 0.2 × 10^{-3}	2.3 ± 0.1 ×10^{-5} s^{-1}	0.014 ± 0.001
**Dy-yD**	THF	90 ± 2 ×10^{-4} s^{-1}	5.5 ± 0.2	4.7 ± 0.2 ×10^{-4} s^{-1}	0.026 ± 0.002
	MeCN	14.2 ± 0.3 ×10^{-4} s^{-1}	0.11 ± 0.01	1.9 ± 0.1 ×10^{-4} s^{-1}	0.026 ± 0.001
**Ay-yA**[a]	MeCN	95 ± 2 ×10^{-4} s^{-1}	9.2 ± 0.5	4.0 ± 0.1 ×10^{-4} s^{-1}	0.13 ± 0.01
	water	172 ± 7 ×10^{-4} s^{-1}	17 ± 1	4.5 ± 0.1 ×10^{-4} s^{-1}	0.12 ± 0.01
**Dy-yA**	THF	10 ± 1 ×10^{-4} s^{-1}	0.077 ± 0.004	1.7 ± 0.1 ×10^{-4} s^{-1}	0.039 ± 0.003
	MeCN	1.8 ± 0.2 ×10^{-4} s^{-1}	0.012 ± 0.001	1.3 ± 0.1 ×10^{-4} s^{-1}	0.039 ± 0.004
**Dy-A**	THF	7.7 ± 0.7 ×10^{-4} s^{-1}	0.059 ± 0.004	9.4 ± 0.5 ×10^{-5} s^{-1}	0.041 ± 0.002
	MeCN	8.6 ± 0.7 ×10^{-5} s^{-1}	0.015 ± 0.001	5.9 ± 0.3 ×10^{-5} s^{-1}	0.043 ± 0.003
**D-yA**	THF	2.5 ± 0.2 ×10^{-4} s^{-1}	0.028 ± 0.002	1.7 ± 0.1 ×10^{-4} s^{-1}	0.047 ± 0.003
	MeCN	3.6 ± 0.2 ×10^{-5} s^{-1}	5.0 ± 0.3 × 10^{-3}	1.3 ± 0.1 ×10^{-4} s^{-1}	0.040 ± 0.003

[a] Measured in MeCN as the hexafluorophosphate salt and in water as the chloride salt

S6. Determination of Percentage Conversion at Photostationary State by ^{1}H NMR

The percentage conversion at the PSS, or the photostationary state distribution (PSD), of each compound was measured by comparing the ^{1}H NMR spectrum of the open-form DTE with the final ^{1}H NMR spectrum of the DTE after irradiating with 365 nm light at 159.1 mW until no further change in the spectrum was observed. All NMR spectra here were recorded with extended (10 s) relaxation time. The aromatic proton signals were integrated and used to determine the relative percentage of the two isomers. A typical procedure for determining the percentage conversion at PSS is as follows: a sample of **D-D** in THF-d_8 was irradiated with 630 nm light at 112.0 mW for 3 minutes and then the NMR spectrum of the open-form was recorded. The sample was then irradiated with 365 nm light at 159 mW for 5 minutes and then the NMR spectrum was recorded. Irradiation with 365 nm light for 5 minutes followed by taking NMR spectrum was repeated for 7 times until no further change in the NMR spectrum was observed. The integration ratios of the peaks H(a):H(a') and H(b):H(b') at the PSD were measured to give a PSD of >99% conversion to the closed form, i.e. no detectable trace of the open isomer. The uncertainty associated with NMR integration is evaluated by setting the integration of H(a) peak to 2.00 and randomly integrate on the baseline where no peak occurs (with the width of 0.05 ppm) for ten times, giving average uncertainty of ± 0.02, which corresponds to 1% error in the integration value.

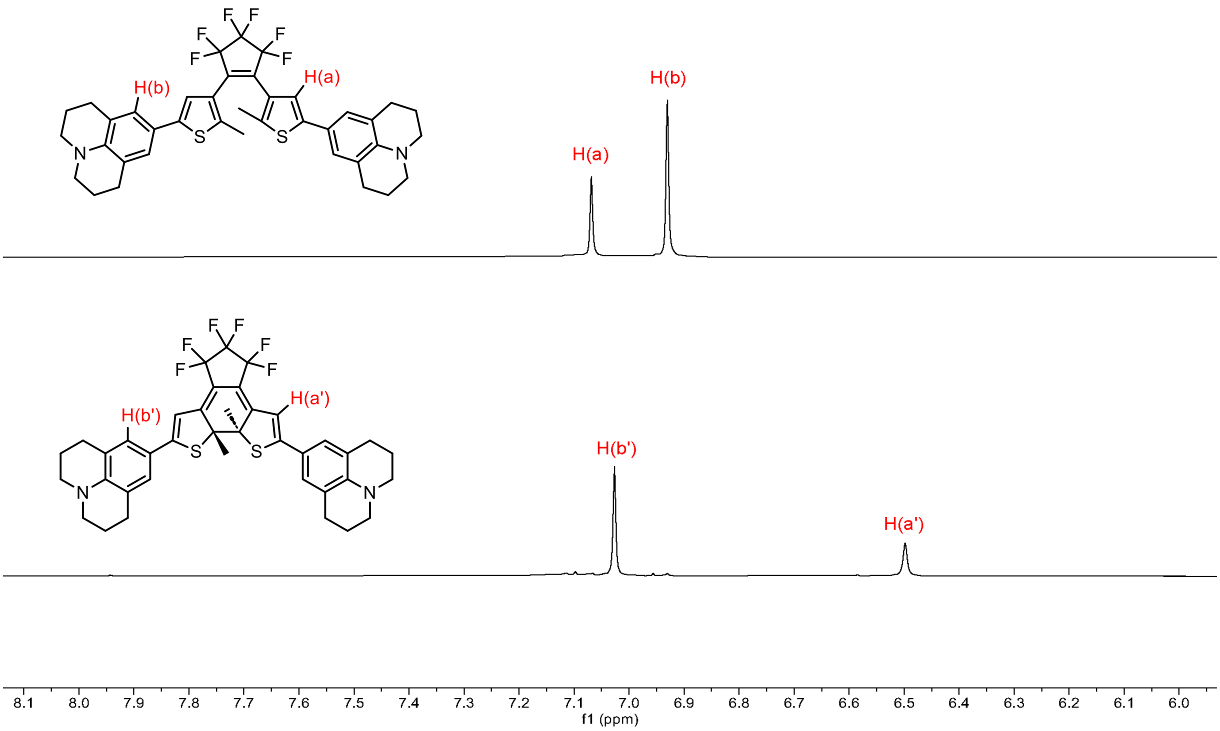

Figure S17 ^{1}H NMR spectra (500 MHz) of open-form **D-D** (top) and >99% conversion reached at PSS by 365 nm irradiation (bottom) in THF-d_8.

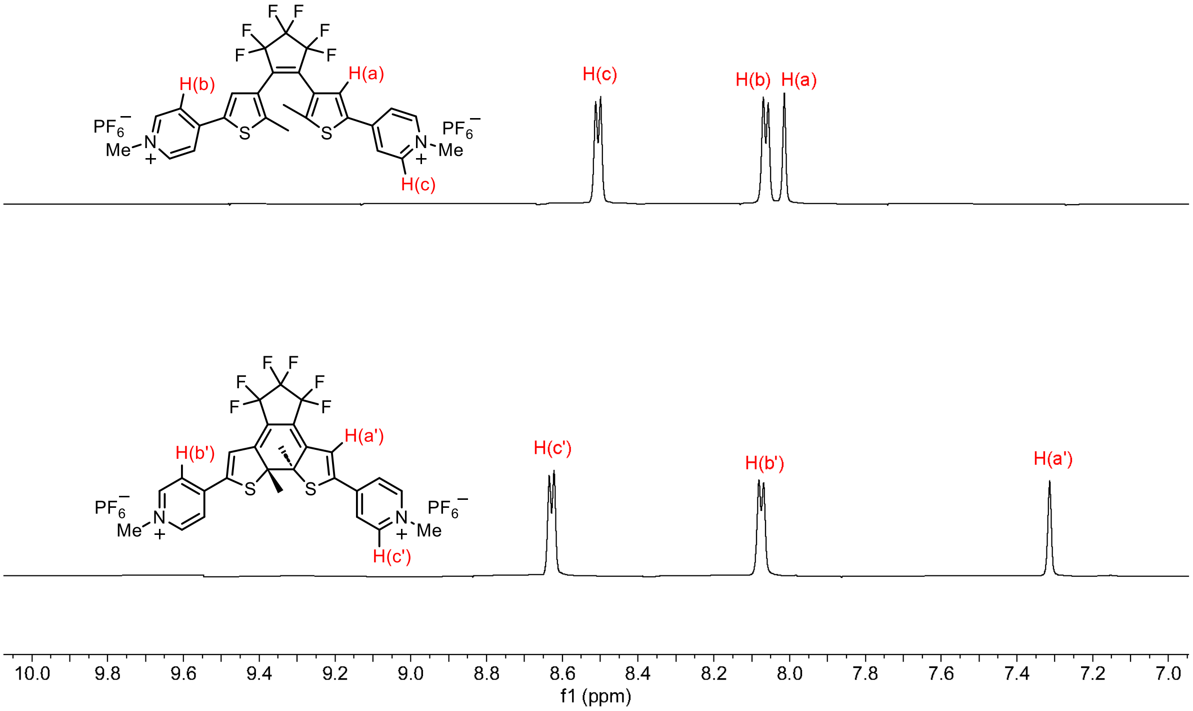

Figure S18 ^{1}H NMR spectra (500 MHz) of open-form **A-A** (top) and >99% conversion reached at PSS by 365 nm irradiation (bottom) in MeCN-d_3.

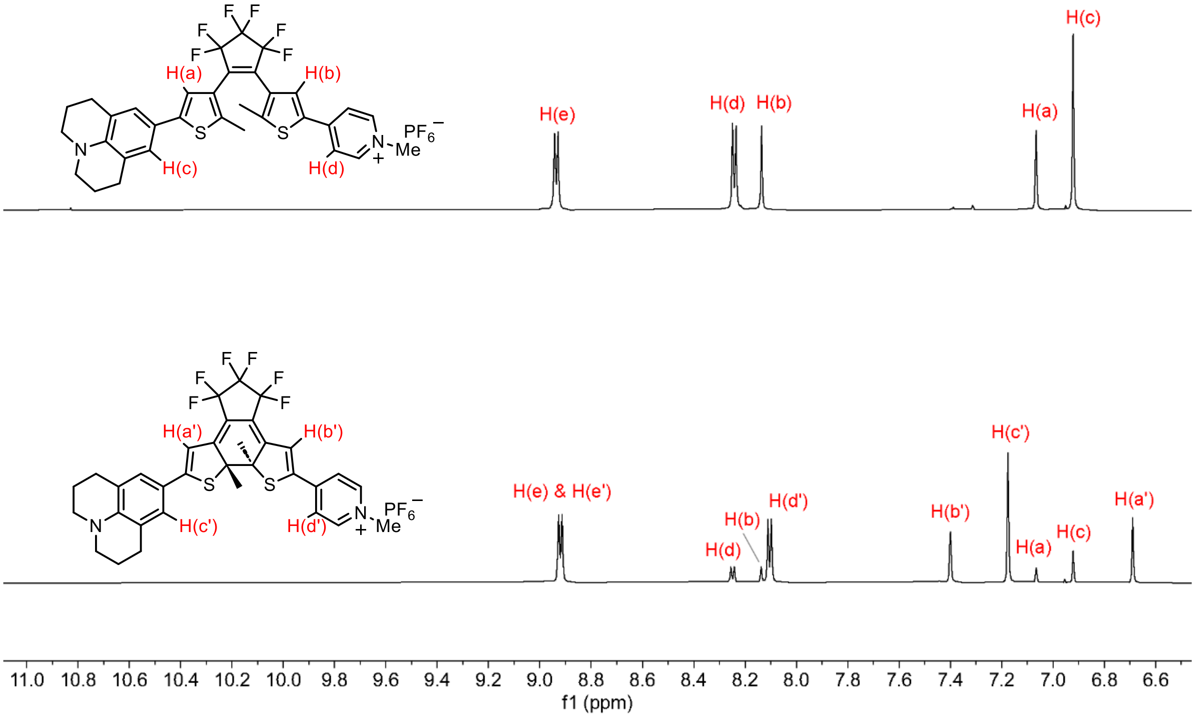

Figure S19 ^{1}H NMR spectra (500 MHz) of open-form **D-A** (top) and 71% ± 1% conversion reached at PSS by 365 nm irradiation (bottom) in THF-d_8.

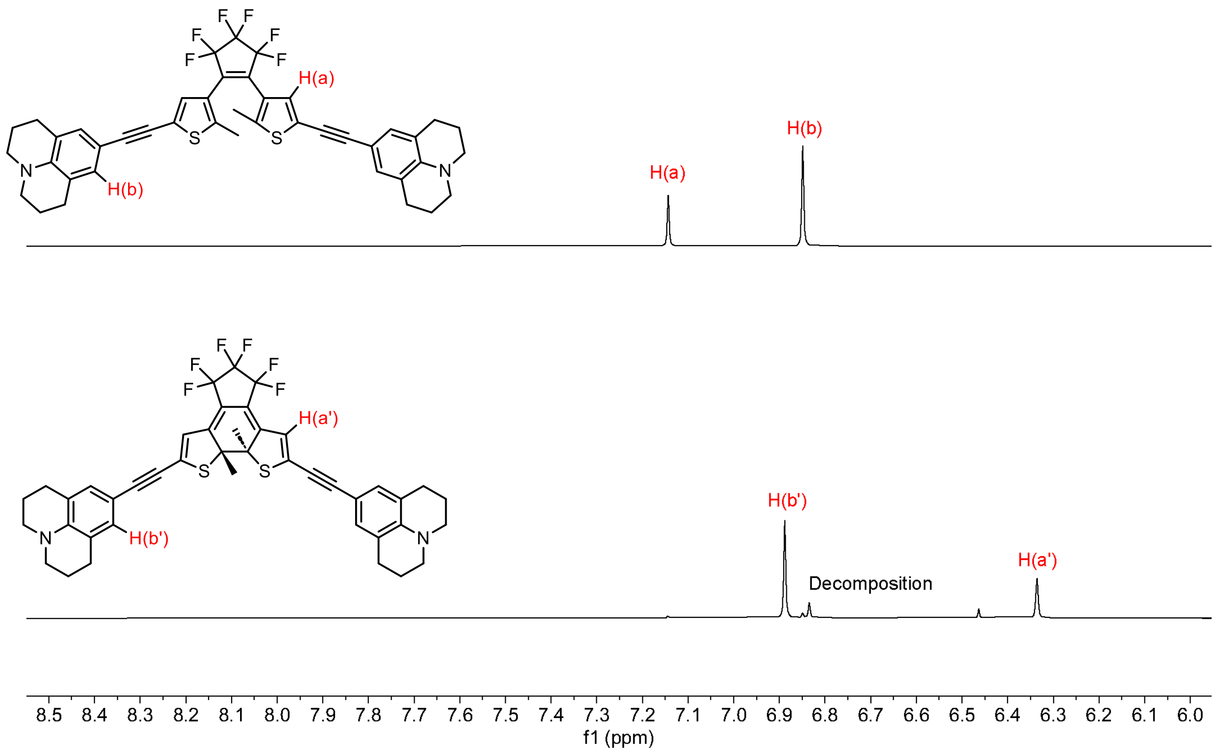

Figure S20 ^{1}H NMR spectra (500 MHz) of open-form **Dy-yD** (top) and >99% conversion reached at PSS by 365 nm irradiation (bottom) in THF-d_8.

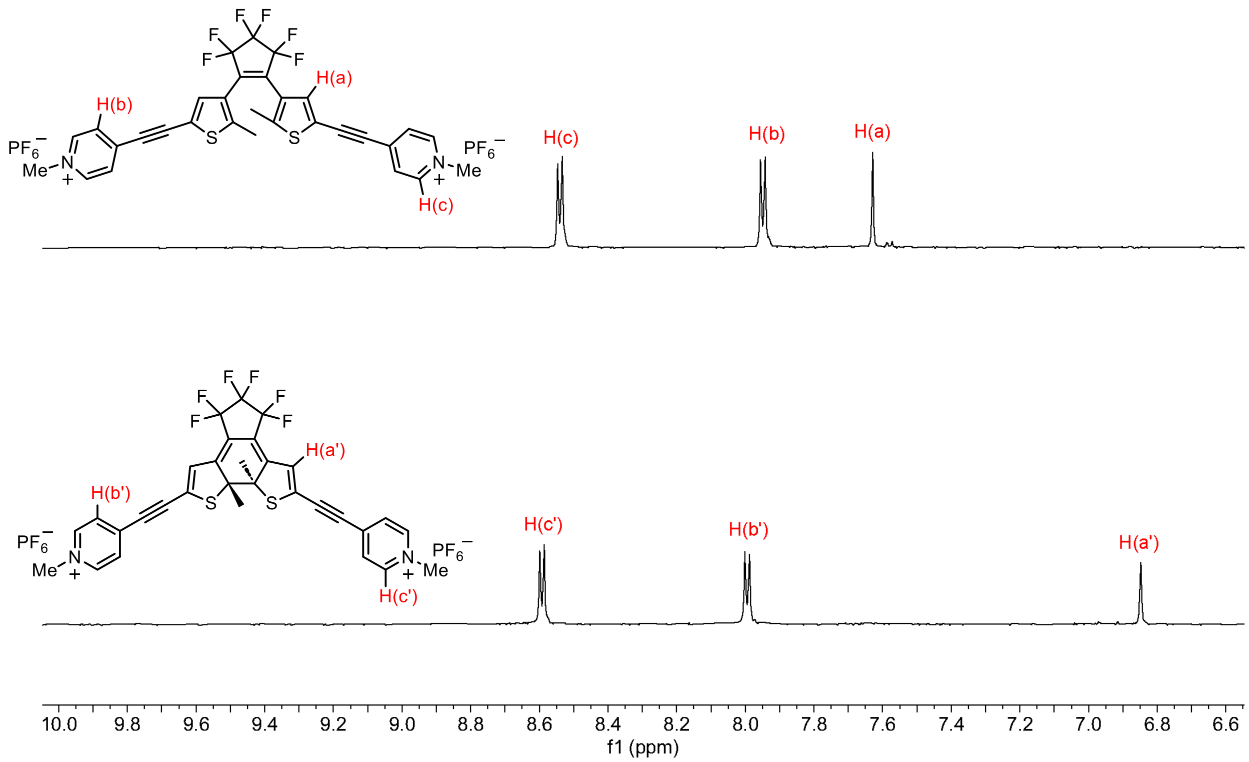

Figure S21 ^{1}H NMR spectra (500 MHz) of open-form **Ay-yA** (top) and >99% conversion reached at PSS by 365 nm irradiation (bottom) in MeCN-d_3.

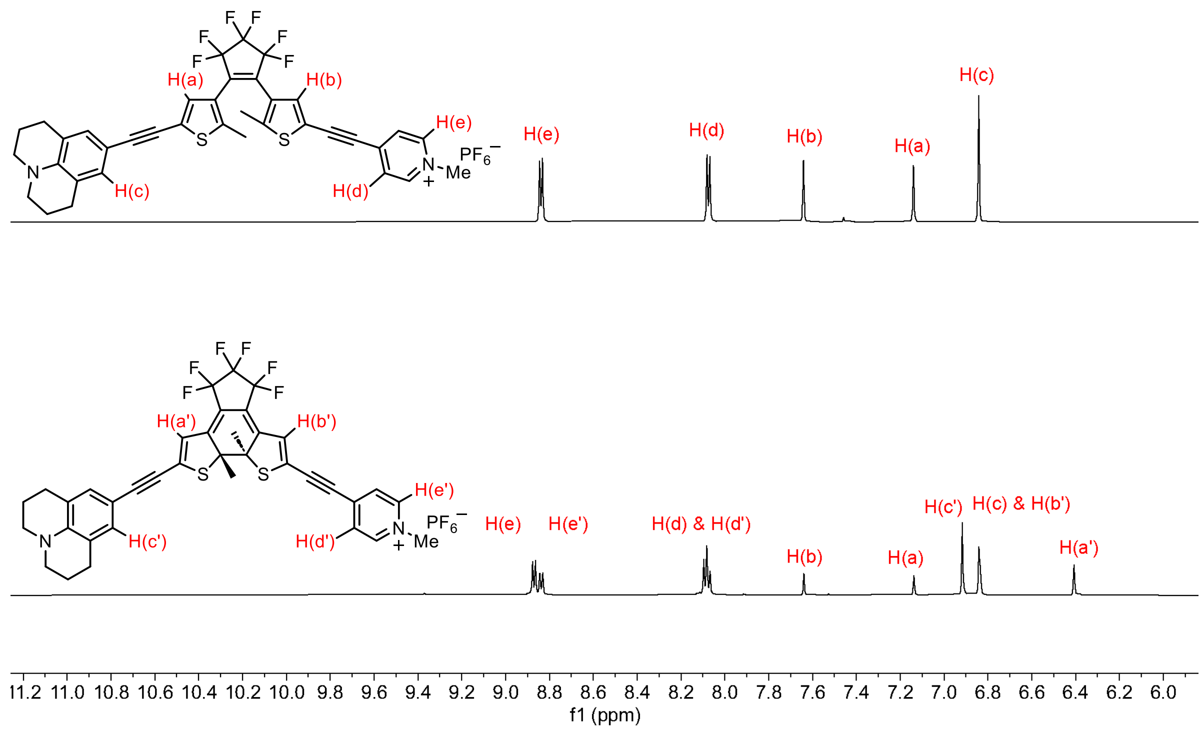

Figure S22 ^{1}H NMR spectra (500 MHz) of open-form **Dy-yA** (top) and 80% ± 1% conversion reached at PSS by 365 nm irradiation (bottom) in THF-d$_8$.

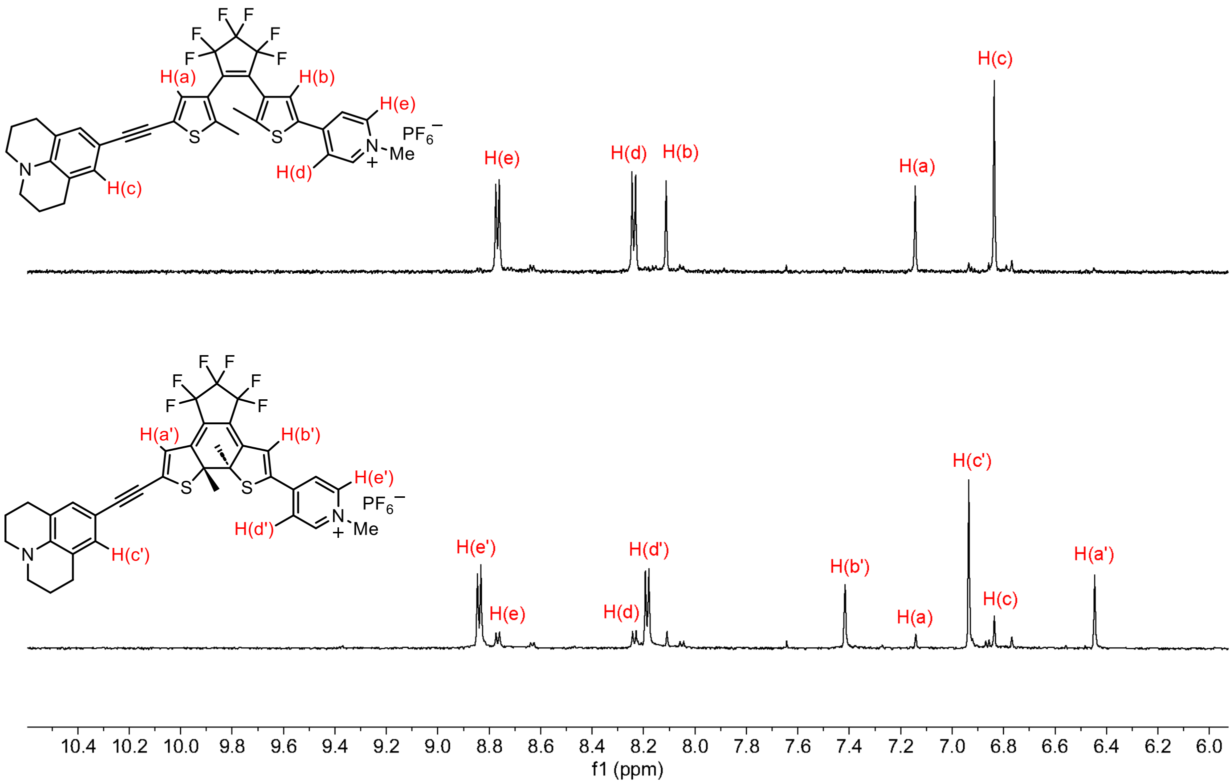

Figure S23 ^{1}H NMR spectra (500 MHz) of open-form **Dy-A** (top) and 85% ± 1% conversion reached at PSS by 365 nm irradiation (bottom) in THF-d$_8$.

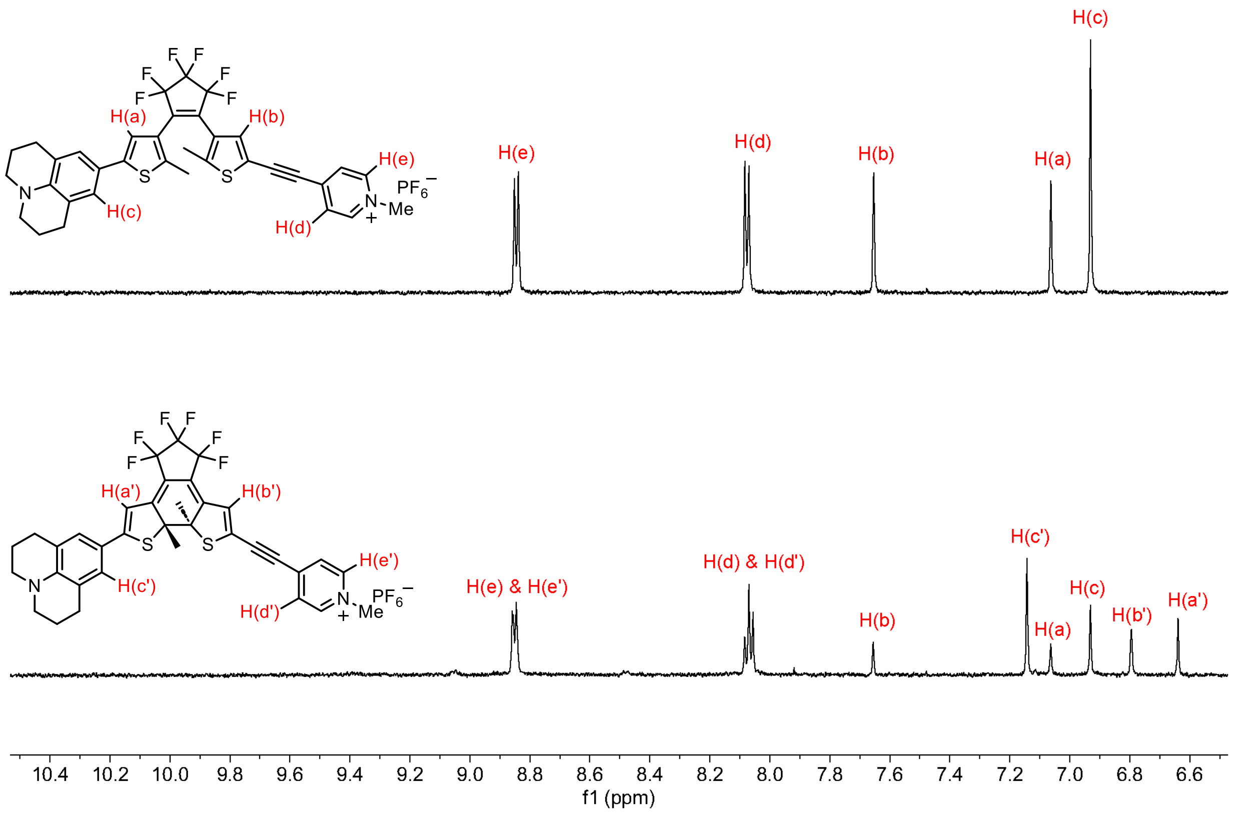

Figure S24 ^{1}H NMR spectra (500 MHz) of open-form **D-yA** (top) and 66% ± 1% conversion reached at PSS by 365 nm irradiation (bottom) in THF-d_8.

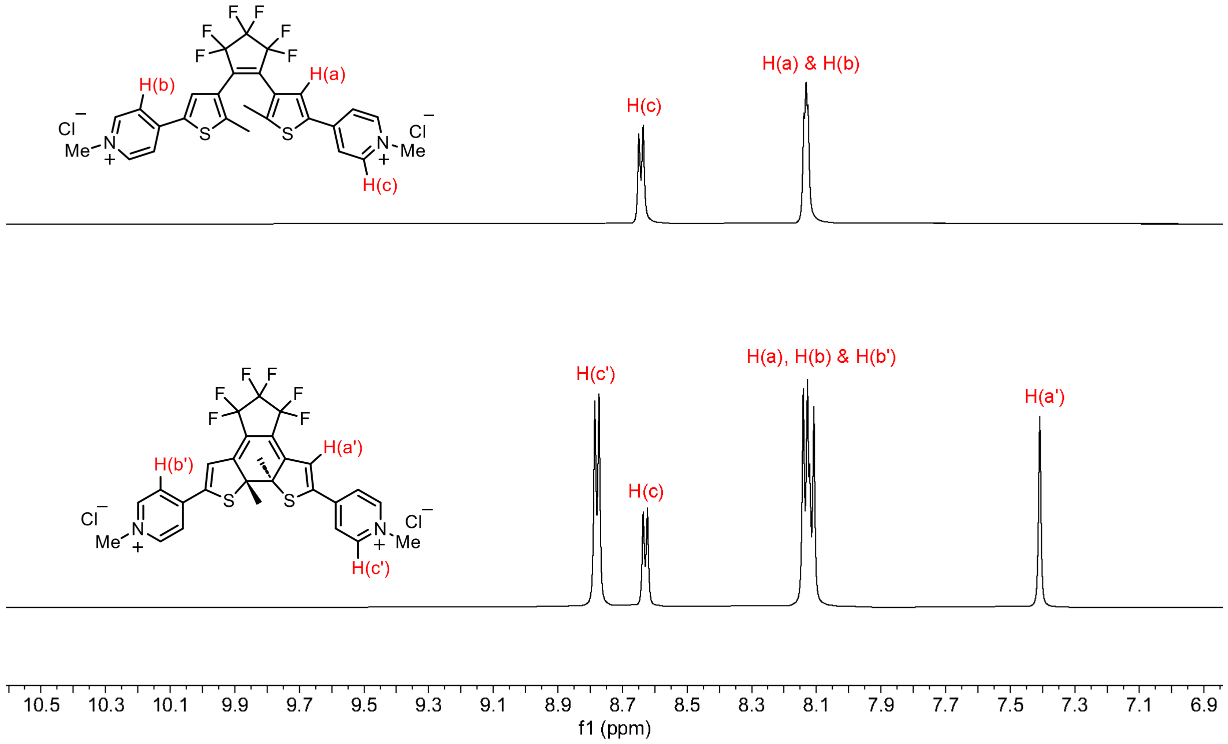

Figure S25 ^{1}H NMR spectra (500 MHz) of open-form **A-A** (chloride) (top) and 71% ± 1% conversion reached at PSS by 405 nm light irradiation (bottom) in D_2O.

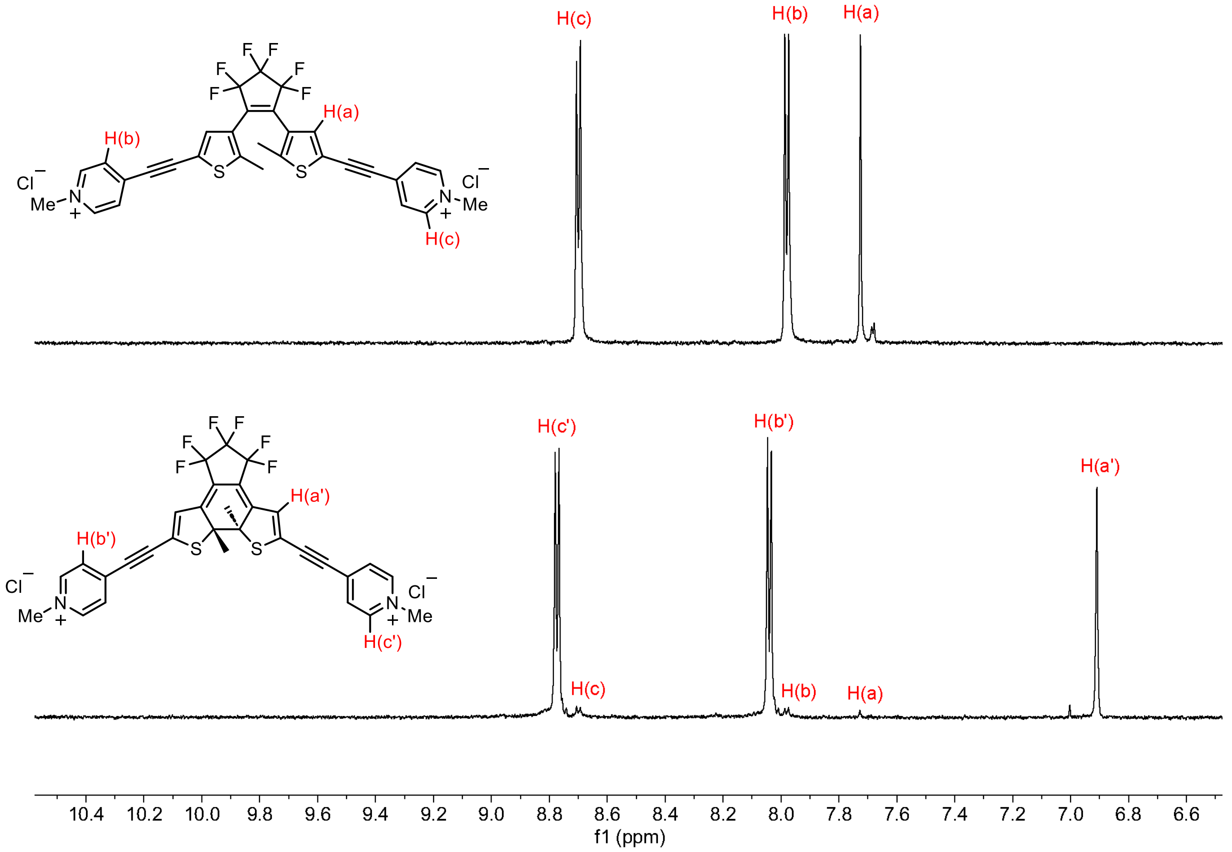

Figure S26 ^{1}H NMR spectra (500 MHz) of open-form **Ay-yA** (chloride) (top) and 96% ± 1% conversion reached at PSS by 405 nm irradiation (bottom) in D_2O.

S7. Determination of Percentage Conversion at Photostationary State by UV-vis

The percentage conversion at PSS of **D-D**, **D-A**, **Dy-yD**, **Dy-yA**, **Dy-A** and **D-yA** in acetonitrile could not be characterized by NMR because of slow switching resulting from low photochemical quantum yields. It was not possible reach the PSD after several hours of irradiation at NMR concentration. Therefore, their percentage conversion at PSD was characterized by UV-vis spectroscopy. A typical procedure for obtaining the percentage conversion at PSD in acetonitrile is as follows: the UV-vis spectra of the open-form and the PSD reached in THF of **Dy-yA** were measured in acetonitrile. An open-form **Dy-yA** sample in acetonitrile was then repeatedly irradiated with 365 nm light and the UV-vis absorption was monitored after each cycle of irradiation until no further change in the UV-vis spectrum was observed, representing the UV-vis absorption of the PSS reached in acetonitrile. The percentage conversion at PSS in acetonitrile was then calculated taking the closed-form absorption maximum (693 nm) as the reference point based on the fact that the PSS absorption spectrum is a linear combination of open-form absorption and closed-form absorption, yielding the result of 33% conversion at PSS in acetonitrile with 365 nm light irradiation **(Figure S27)**.

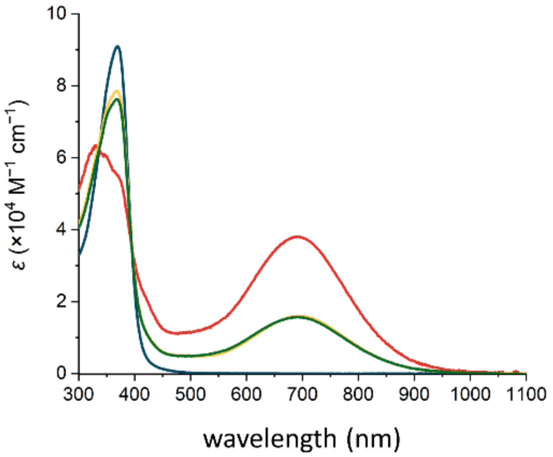

Figure S27 Determining percentage conversion of **Dy-yA** at PSS by UV-vis method. The blue line represents the absorption of open-form **Dy-yA** in acetonitrile. The red line represents the absorption of a **Dy-yA** sample (80% conversion at PSS in THF) in acetonitrile. The yellow line represents the experimental PSS reached by 365 nm light irradiation in acetonitrile. The green line represents the calculated PSD containing 33% closed-form in acetonitrile.

The conversions from open-form to closed-form of all DTEs at photostationary state in different solvents under light irradiation measured by either NMR method or UV-vis method are summarized in **Table S6**. The PSS reached with 405 nm light irradiation was examined for **A-A** (chloride) and **Ay-yA** (chloride) in water using the NMR method, demonstrating the incorporation of additional alkyne can lead to 25% increase in the PSD due to red-shifted open-form absorption spectrum.

Table S6 Summary of photostationary state distribution measured by NMR and UV-vis spectroscopy. Open-to-closed photostationary states were achieved by irradiation with 365 nm or 405 nm light. Closed-to-open photostationary states were achieved by irradiation with 630 nm light.

Compound	Solvent	$PSD_{O\text{–}C}$ (365 nm) (%)	$PSD_{O\text{–}C}$ (405 nm) (%)	$PSD_{C\text{–}O}$ (630 nm) (%)
**D-D**	THF	>99	/	100
	MeCN	69	/	100
**A-A**[a]	MeCN	>99	/	100
	water	>99	71	100
**D-A**	THF	71	/	100
	MeCN	21	/	100
**Dy-yD**	THF	>99	/	100
	MeCN	86	/	100
**Ay-yA**[a]	MeCN	>99	/	100
	water	>99	96	100
**Dy-yA**	THF	80	/	100
	MeCN	33	/	100
**Dy-A**	THF	85	/	100
	MeCN	64	/	100
**D-yA**	THF	66	/	100
	MeCN	26	/	100

[a] Measured in MeCN as the hexafluorophosphate salt and water as the chloride salt.

S8. Calculation of PSD Based on Experimental Molar Absorption Coefficient and Quantum Yield

After verifying the reproducibility of the measurements of photochemical switching quantum yields, we further analyzed the reliability of the result by calculating the PSD based on the experimental molar absorption coefficient and quantum yield and comparing the calculated PSD with the experimental PSD. All the experimental and calculated PSD values are listed in **Table S7**. The PSD of forward reaction (PSD_λ) is expressed as the fraction of closed-form DTE and is calculated using **Equations 1** and **3**,

$$\mathrm{PSD}_\lambda = \frac{[\mathrm{closed}]}{[\mathrm{open}] + [\mathrm{closed}]} \tag{1}$$

$$\mathrm{PSD}_\lambda = \frac{\varepsilon_{\mathrm{open},\lambda} \cdot \Phi_{\mathrm{o-c}}}{\varepsilon_{\mathrm{closed},\lambda} \cdot \Phi_{\mathrm{c-o}} + \varepsilon_{\mathrm{open},\lambda} \cdot \Phi_{\mathrm{o-c}}} \tag{3}$$

where $\varepsilon_{\mathrm{open},\lambda}$ and $\varepsilon_{\mathrm{closed},\lambda}$ are the molar absorption coefficients of the open and closed isomers, respectively, at the excitation wavelength; $\Phi_{\mathrm{o-c}}$ and $\Phi_{\mathrm{c-o}}$ are the quantum yields for isomerization in each direction.

Table S7 Experimental and calculated PSD in different solvents.

Compound	Solvent	Exp. PSD_{o-c} (365 nm) /%	Exp. PSD_{o-c} (405 nm) /%	Calc. PSD_{o-c} (365 nm) /%	Calc. PSD_{o-c} (405 nm) /%
**D-D**	THF	>99	/	99.8	/
	MeCN	69	/	79.1	/
**A-A**[a]	MeCN	>99	71	99.9	79.2
	water	>99	/	99.9	/
**D-A**	THF	71	/	82.1	/
	MeCN	21	/	28.6	/
**Dy-yD**	THF	>99	/	99.8	/
	MeCN	86	/	93.2	/
**Ay-yA**[a]	MeCN	>99	/	99.6	/
	water	>99	96	99.8	99.2
**Dy-yA**	THF	80	/	78.9	/
	MeCN	33	/	37.1	/
**Dy-A**	THF	85	/	80.5	/
	MeCN	64	/	49.5	/
**D-yA**	THF	66	/	72.4	/
	MeCN	26	/	36.2	/

[a] Measured in MeCN for the hexafluorophosphate salt and water for the chloride salt.

S9. Determination of Fatigue Resistance by UV-vis Spectroscopy

The fatigue resistance of a DTE is characterized by repeatedly switching the DTE between the open-form and closed-form and monitoring the change of the UV-vis signal maximum corresponding to the closed-form, as shown in **Figure S28**. The change in absorbance after a certain number of cycles of switching gives an indication of whether decomposition due to light irradiation occurs as well as the possible pathway of decomposition.

Given that each DTE has different photochemical quantum yields, we did not use a uniform sample concentration in the cuvette, irradiation time and irradiation power to measure the fatigue resistance. Instead, the optimal combination of parameters was measured so that the PSS of photocyclization and photoreversion are just reached. The optimal combination of parameters is used to avoid decomposition due to over-irradiation.

A typical procedure for measuring the fatigue resistance is as follows: a THF solution of **D-D** (2.8 µM, 3.0 mL) was placed in a cuvette in the sample holder of the UV-vis spectrometer and stirred at 25 °C. The absorbance of the sample at 654 nm was recorded. The solution was then irradiated with a pulse of 365 nm light (18.9 mW, 10 s) and the absorption at 654 nm was recorded. Afterwards, the sample was irradiated with a pulse of 630 nm (112 mW, 3 min) and the absorbance at 653 nm was recorded. This procedure of irradiation with alternative light sources was repeated for 100 cycles and the absorption at 654 nm was recorded after each pulse of irradiation. The UV-vis absorption at 654 nm was plotted against the number of switching cycles.

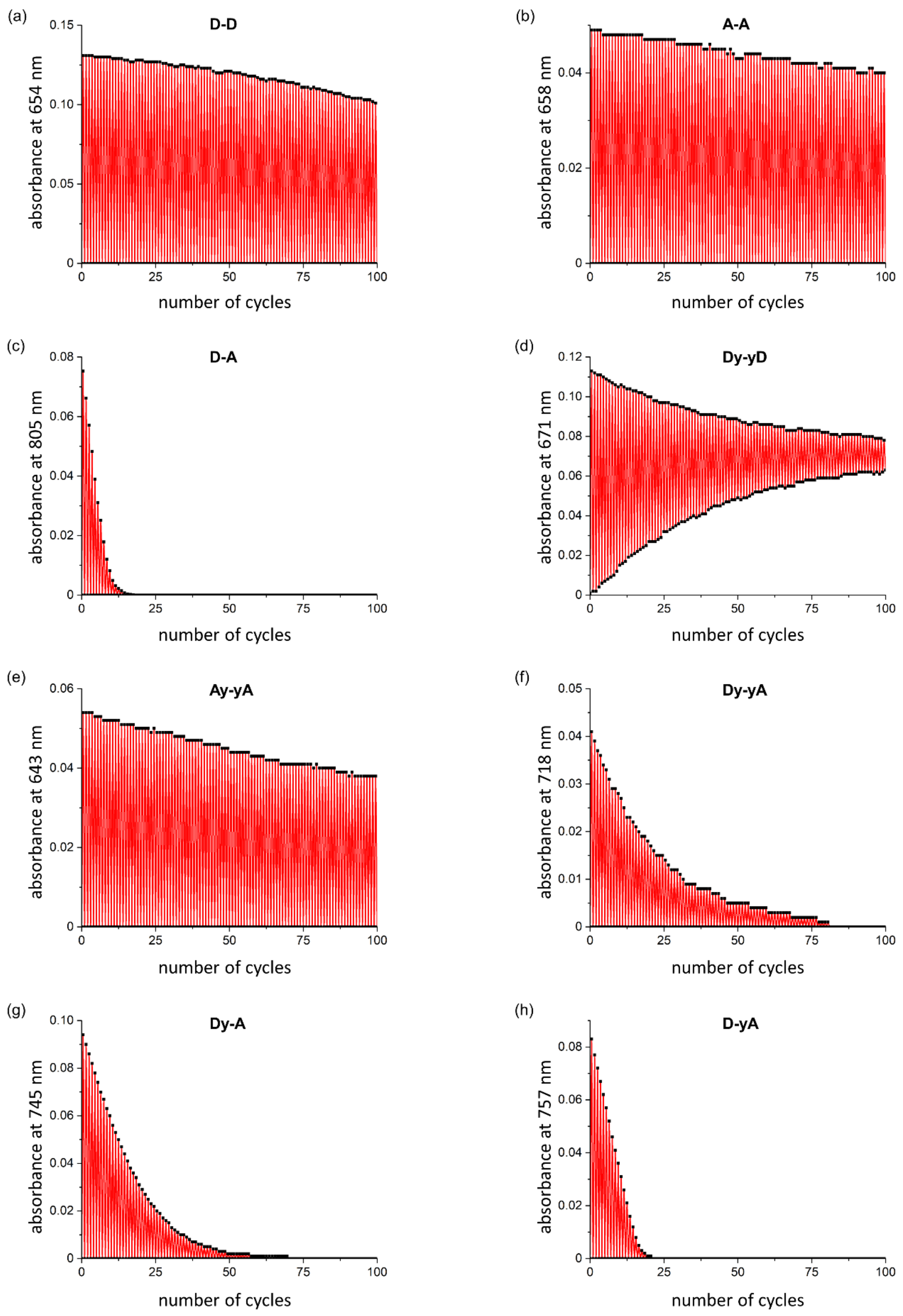

Figure S28 Fatigue resistance of **D-D** (THF), **A-A** (MeCN), **D-A** (THF), **Dy-yD** (THF), **Ay-yA** (MeCN), **Dy-yA** (THF), **Dy-A** (THF) and **D-yA** (THF).

Although almost all previous work on photoswitches report the fatigue resistance in the number of switching cycles, such method has an intrinsic drawback. It is not possible to determine the exact time required to reach the PSS, and therefore, the sample is usually over-irradiated in each cycle. The over-irradiation time could have a significant impact on the number of cycles that the photoswitch can be switched. To better understand the kinetics of decomposition, herein we assume that **A-A** (chloride) and **Ay-yA** (chloride) decompose from their closed forms and monitored the decomposition under extended time of 365 nm light irradiation. A typical procedure for monitoring the decomposition is as follows: a stock solution of **A-A** (chloride) in MeCN was diluted to 2.4 μM in water in a cuvette. The sample was irradiated with 365 nm light (159.1 mW, 2 minutes) to convert all open-form DTE to the closed form. The sample was irradiated with 365 nm light (159.1 mW, 3 minutes) and the absorbance at 659 nm was measured. The process was repeated until the absorbance at 659 nm decayed to zero. The data showing compound decomposition were fitted with a first-order decay curve in Origin. The initial gradient of the two curves were obtained by taking the first derivative of the fitting function and setting the time to zero. The initial gradients were used to calculate the photochemical quantum yield of decomposition of **A-A** (chloride) and **Ay-yA** (chloride), yielding results of 0.0020% ± 0.0003% and 0.0026% ± 0.0003%, respectively.

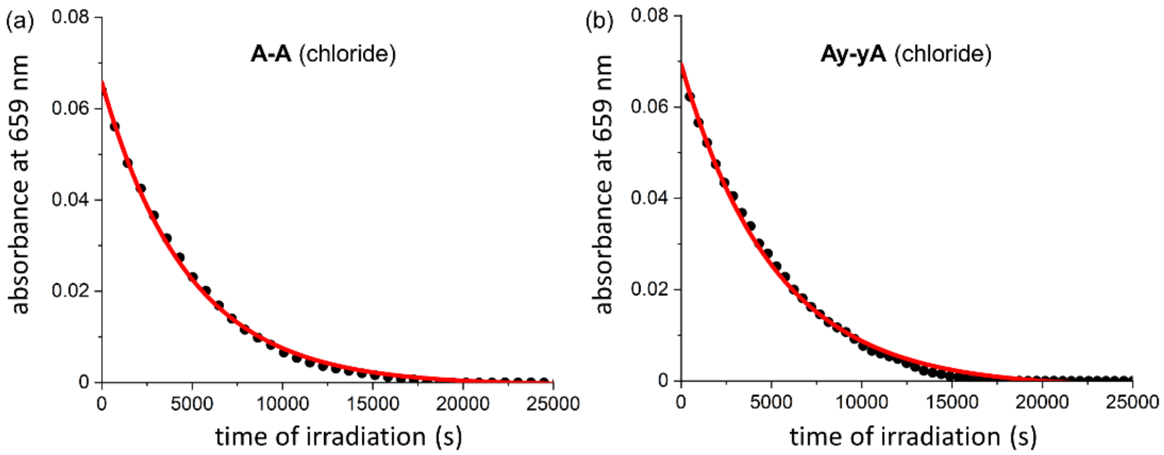

Figure S29 Photo-induced decomposition of the closed forms of (a) **A-A** (chloride) and (b) **Ay-yA** (chloride). The fitted equations are (a) $\boldsymbol{y = 0.0713 \cdot e^{\left(-\frac{x}{7323}\right)} - 0.0022}$ and (b) $\boldsymbol{y = 0.0566 \cdot e^{\left(-\frac{x}{5141}\right)} - 0.0003}$. The initial gradients of (a) and (b) were used to calculate the quantum yield of photochemical decomposition, yielding results of 0.0020% ± 0.0003% and 0.0026% ± 0.0006%, respectively.

S10. Verification of Intramolecular Charge Transfer in D-A by Protonation

The ~130 nm red-shift of the closed-form UV-vis absorption band of **D-A** compared to **D-D** and **A-A** suggests that an intramolecular charge transfer state occurs in the closed-form **D-A** as the only structural difference of **D-A** compared to **D-D** and **A-A** is the donor-acceptor substitution pattern. Additionally, julolidine with the Hammett σ value of –2.03,[9] is a stronger donor than aniline (σ = –1.30) or dimethylaniline (σ = –1.70), while *N*-methylpyridinium group is a strong electron acceptor, with the σ value of 2.57.[10] To verify if intramolecular charge transfer is responsible for the red-shift of the absorption band, we performed a protonation experiment as shown in **Scheme S8**. In the protonation experiment, a stock solution of **D-A** (0.46 mM) in THF was irradiated with 365 nm light until PSS was reached. The stock solution was diluted into MeCN (2.2 μM, 3.0 mL) in a cuvette and the UV-vis absorption spectrum was recorded. Trifluoroacetic acid (TFA, 15.0 μL) was then added to the cuvette under fast stirring and the sample was stirred for 2 min. The UV-vis absorption spectrum of the sample containing TFA was then recorded. Triethylamine (0.1 mL) was then added to the cuvette under fast stirring and the sample was stirred for 2 min. The UV-vis absorption spectrum of the neutralized sample was recorded. The closed-form spectra of **D-A** before and after protonation, and after deprotonation were calculated by taking the 71% conversion at PSS into account and assuming the open-form **D-A** does not absorb between 500 nm and 1100 nm regardless of the state of protonation. The UV-vis absorption spectra of **D-A** (closed), **D-A** (closed) after protonation with TFA, and final **D-A** (closed) deprotonated with triethylamine are shown in **Figure S30**. A clear blue-shift of the absorption band is observed after protonation, changing the compound into an acceptor-acceptor substitution pattern. The intramolecular charge transfer state is recovered upon deprotonation with triethylamine.

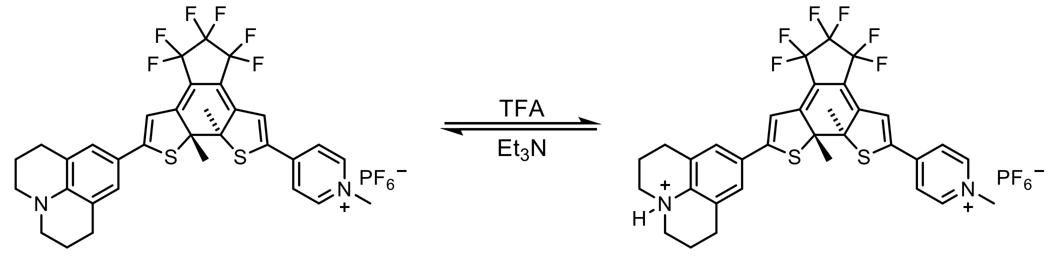

Scheme S8. Protonation of the closed-form **D-A** with TFA and the reverse deprotonation with Et_3N.

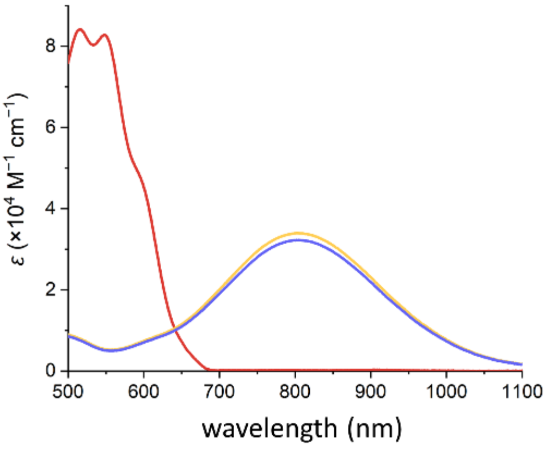

Figure S30. UV-vis-NIR spectra of **D-A** (closed, yellow), **D-A** (closed, red) after protonation, and final deprotonated **D-A** (closed, blue) in MeCN.

S11. Characterization of the Decomposition of Dy-yD

While all other DTEs decompose into non-characterizable species over extended periods of 365 nm light irradiation, **Dy-yD** decomposes into a single species in which some extent of conjugation is retained due to increased absorbance at 671 nm, shown in **Figure S28(d)**.

To measure the UV-vis absorption spectrum of decomposed **Dy-yD**, a solution of **Dy-yD** in THF (2.0 μM) in a cuvette was irradiated with 365 nm light (0.159 W, 1 hour) and the UV-vis spectrum was measured from 280 nm to 1100 nm. The sample was further irradiated and measured for 4 cycles until no further change in the absorption spectrum was observed. Given the clean decomposition pathway, the final absorption spectrum represents the absorption of the decomposed product **(Figure S31)**.

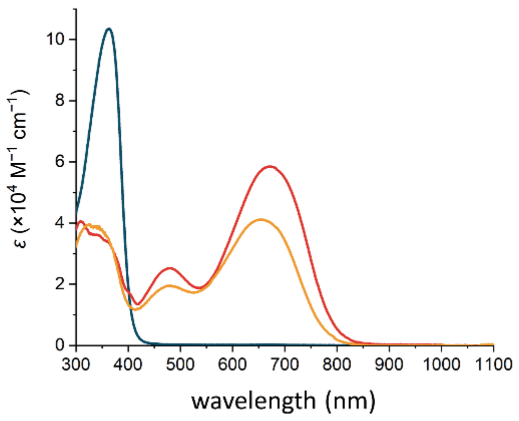

Figure S31 UV-vis absorption spectra of open-form **Dy-yD** (blue), closed-form **Dy-yD** (red) and the decomposed product of **Dy-yD** after extended 365 nm light irradiation in THF (orange).

To characterize the decomposition of **Dy-yD** by NMR, 10 mg of **Dy-yD** was dissolved in THF-d_8 and ^{1}H, ^{13}C, COSY, HSQC and HMBC NMR spectra were recorded. The NMR sample was then irradiated with 365 nm light at 159 mW for 20 min and ^{1}H NMR was recorded. The NMR sample was further irradiated with 365 nm light at 159 mW for 3 hours to form the decomposed **Dy-yD** and ^{1}H, ^{13}C, COSY, HSQC and HMBC NMR spectra were recorded. No purification was performed after each irradiation.

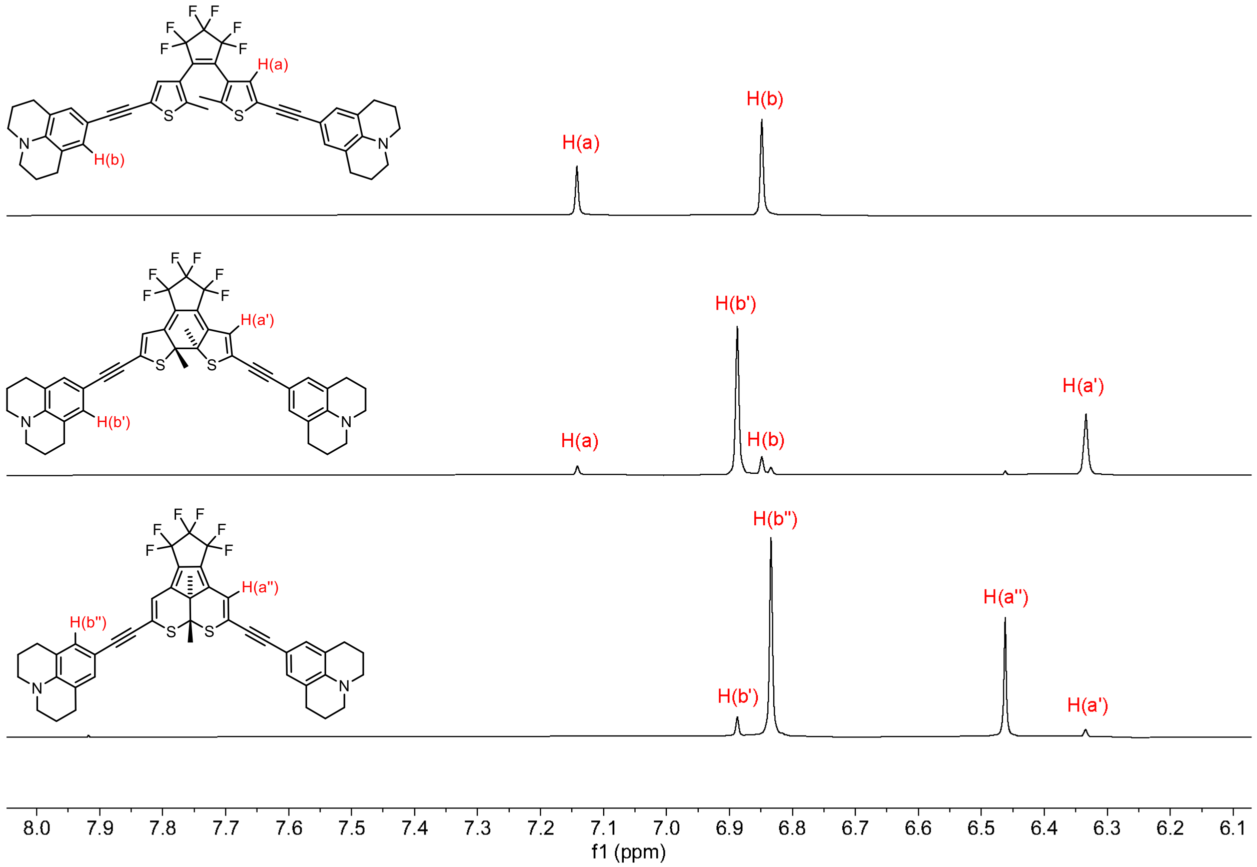

Figure S32 ^{1}H NMR (500 MHz) spectra of open-form **Dy-yD** (top), before reaching PSS by 365 nm irradiation (middle), and decomposed product (bottom) in THF-d_8.

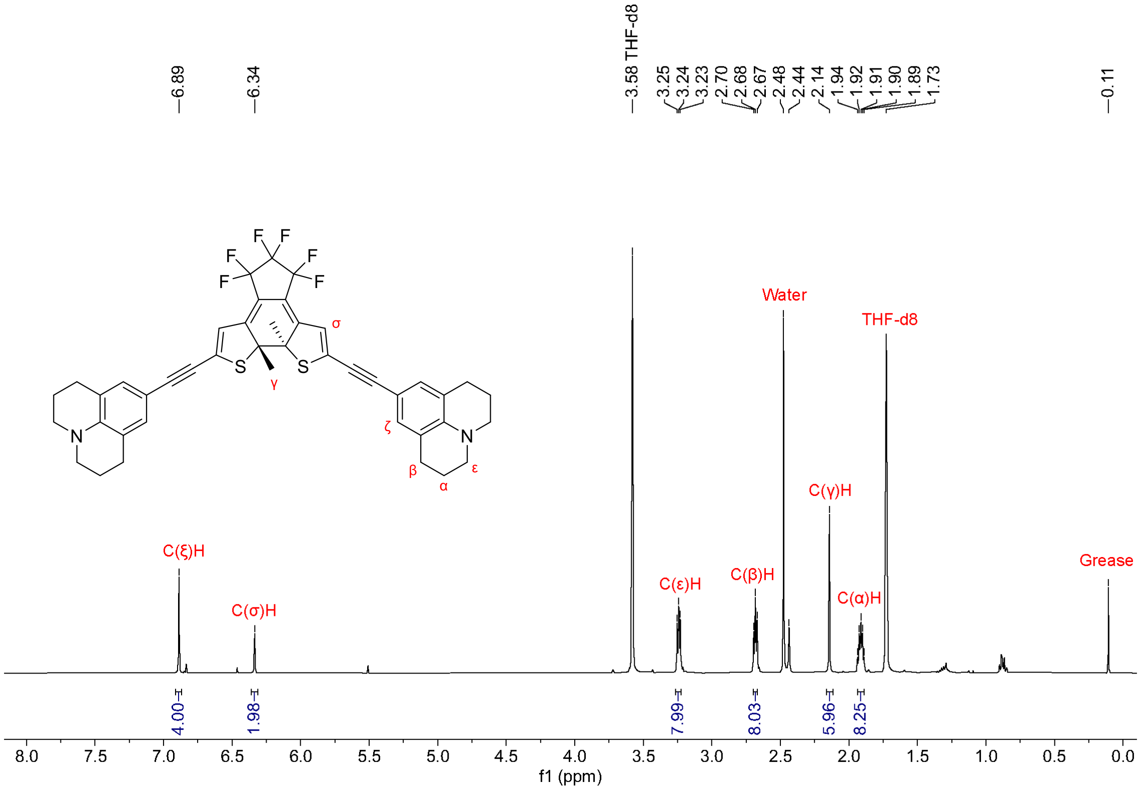

Figure S33 ^{1}H NMR spectrum (500 MHz, THF-d8) of closed-form **Dy-yD.**

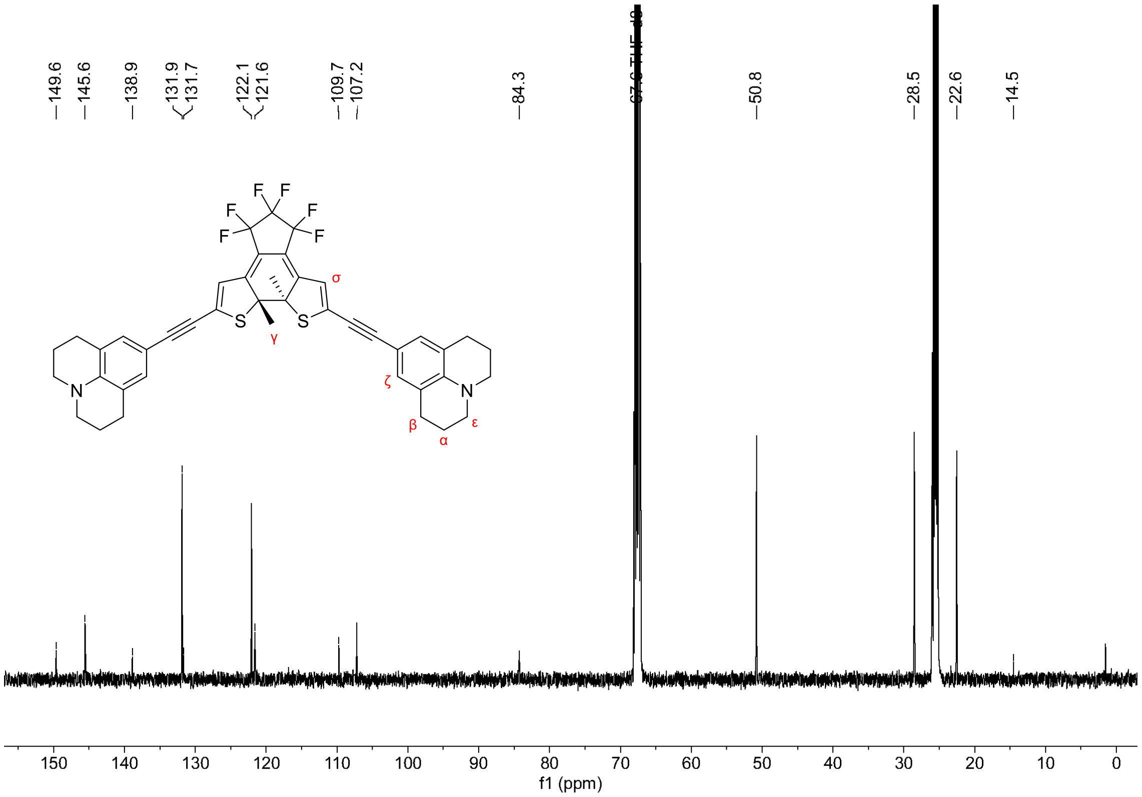

Figure S34 ^{13}C NMR spectrum (101 MHz, THF-d8) of closed-form **Dy-yD**.

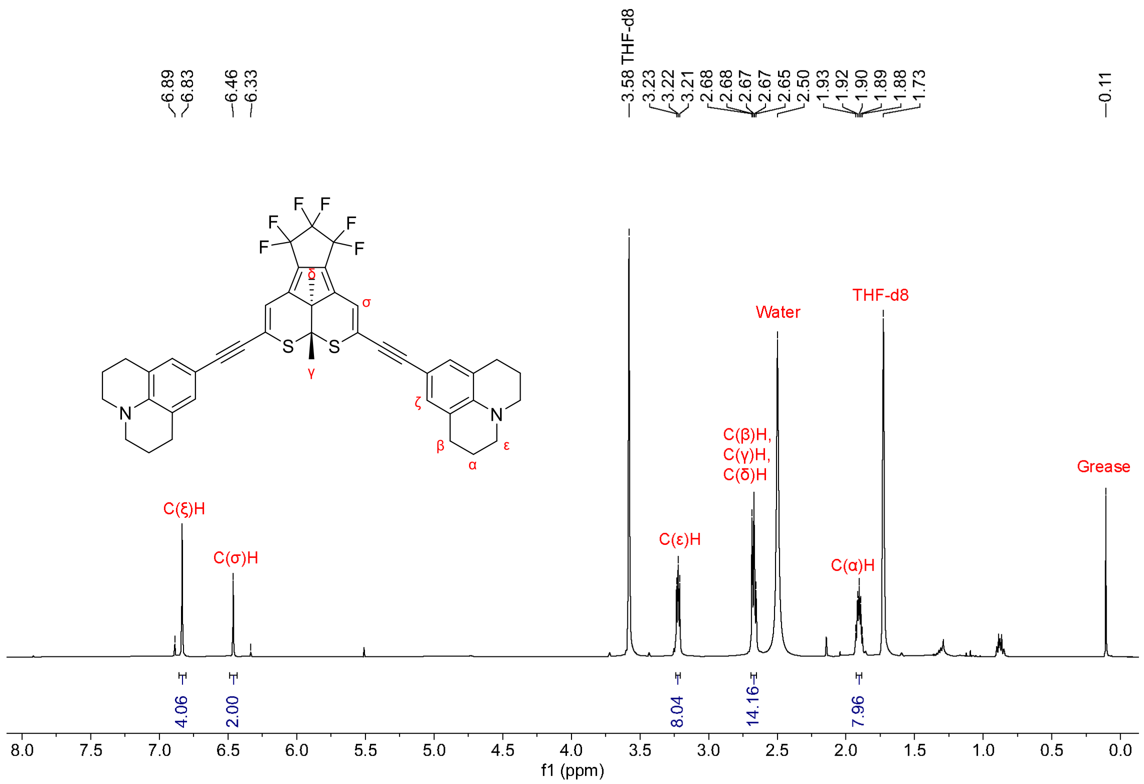

Figure S35 ^{1}H NMR spectrum (500 MHz, THF-d$_8$) of decomposed **Dy-yD** containing 6% closed-form **Dy-yD**.

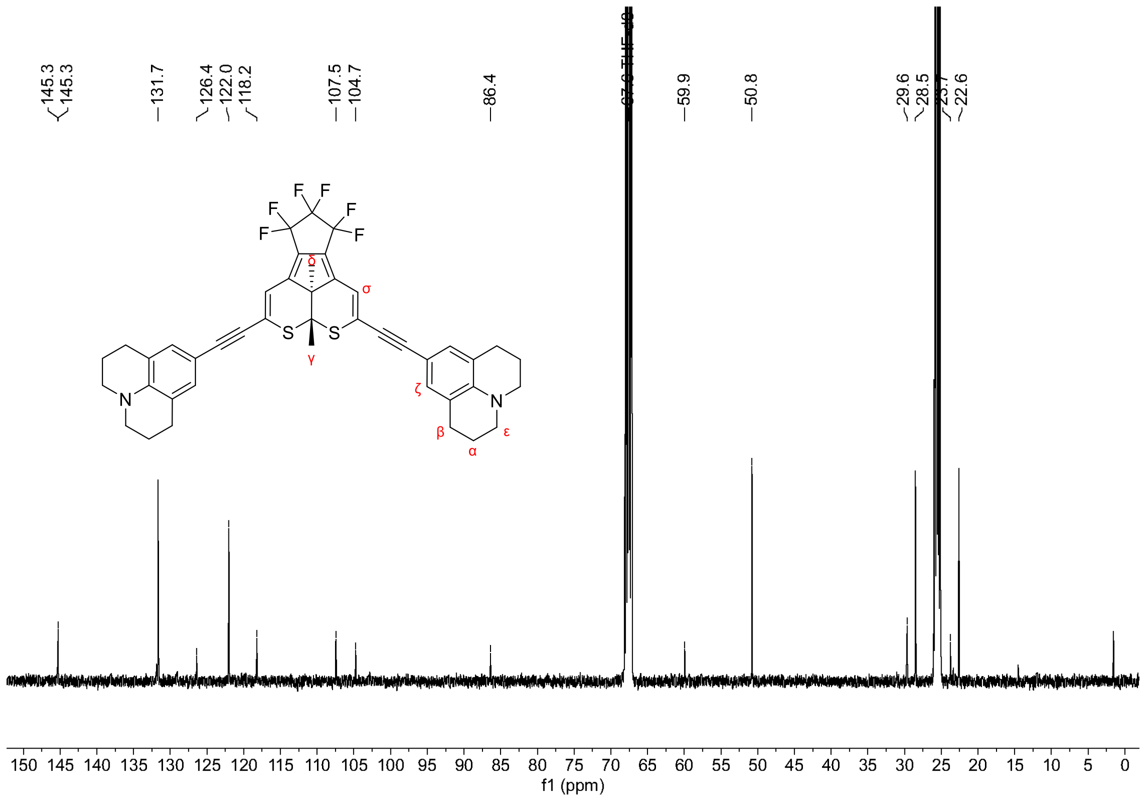

Figure S36 ^{1}H NMR spectrum (500 MHz, THF-d$_8$) of decomposed **Dy-yD.**

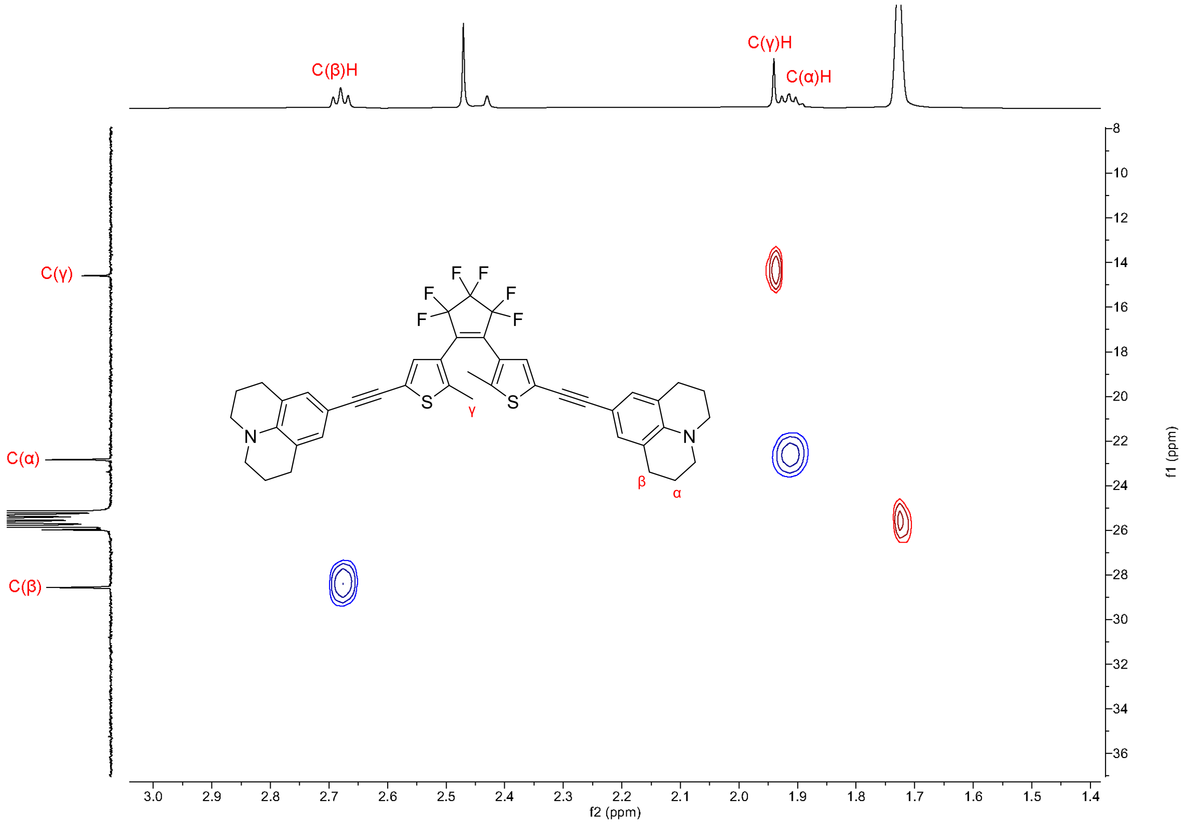

Figure S37 HSQC assignment of open-form **Dy-yD**.

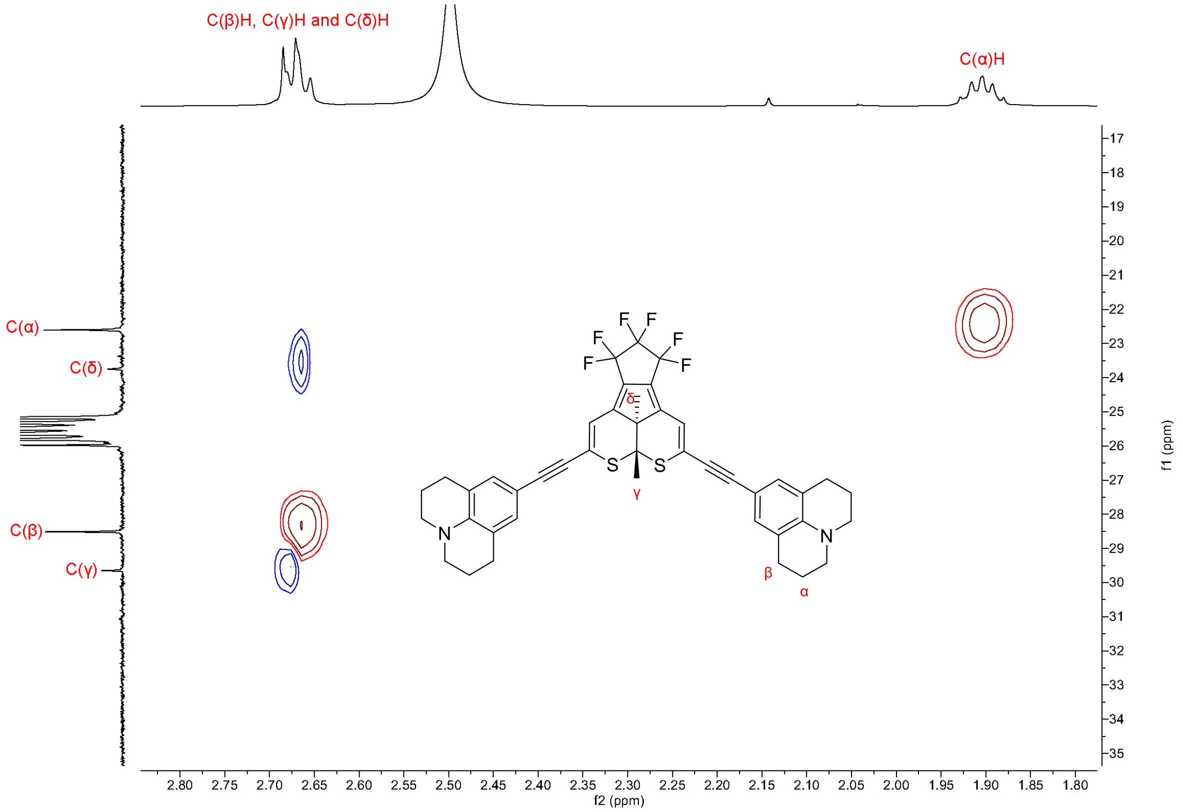

Figure S38 HSQC assignment of decomposed **Dy-yD** showing that C(γ) and C(δ) are no longer equivalent.

Based on the fact that C(γ) and C(δ) are no longer equivalent after decomposition **(Figure S37)**, the following decomposition pathway **(Scheme S9)** is proposed, taking previous reports on the well-established DTE decomposition pathway into account work.[11-14]

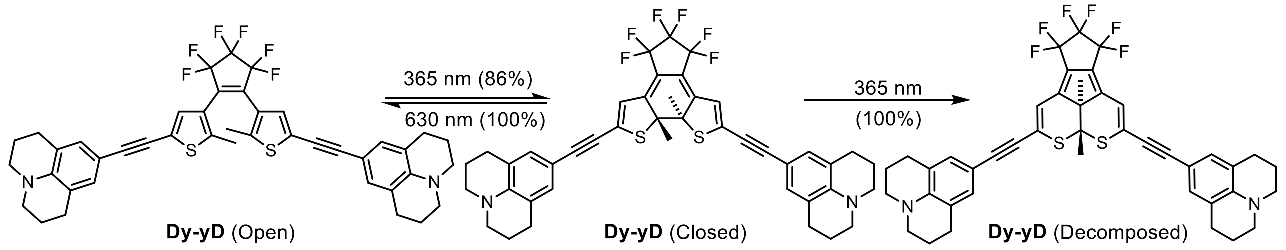

Scheme S9 Decomposition pathway of **Dy-yD**.

S12. Absorption Solvatochromism of Closed-Form D-A

The solvatochromic behavior of the UV-vis absorption spectra of closed-form **D-D**, **A-A**, **D-A** and **Dy-yA** was examined in several different solvents. The typical procedure for measuring solvatochromic behavior is described as follows: A stock solution of **D-A** at PSS (365 nm) in THF (see Section S10 for details) was diluted into a range of different solvents (final concentration 2.2 μM) and the UV-vis absorption spectrum was recorded (Figure S38).

No significant solvatochromism was observed for **D-D (Figure S39(a))**, while negative solvatochromism (bathochromic shift of the absorption band with decreased solvent polarity) was observed for **D-A** and **Dy-yA (Figure S39)**. **D-A** also shows an abnormal red-shift in halogenated solvents, including CH_2Cl_2, chloroform, 1,2-dichloroethane (1,2-DCE), dibromomethane (DBM), bromoform, iodomethane, and chlorobenzene. **A-A** also does not show significant solvatochromic behavior, but abnormal behavior in halogenated solvents is also observed.

The energies of the absorption maxima of **D-A** in different solvents were plotted against the solvent dielectric constants **(Figure S39(f))**. A linear dependence of absorption maximum on dielectric constant was observed but the gradient in halogenated solvents is different from the gradient in non-halogenated solvents. The intercept dielectric constant of the two linear fits is 1.08. These data indicate that the halogenation of the solvent is the cause of the unusual solvatochromic behavior. These results suggest that specific interaction between the DTE and the halogenated solvents is responsible for the abnormal solvatochromic behavior.

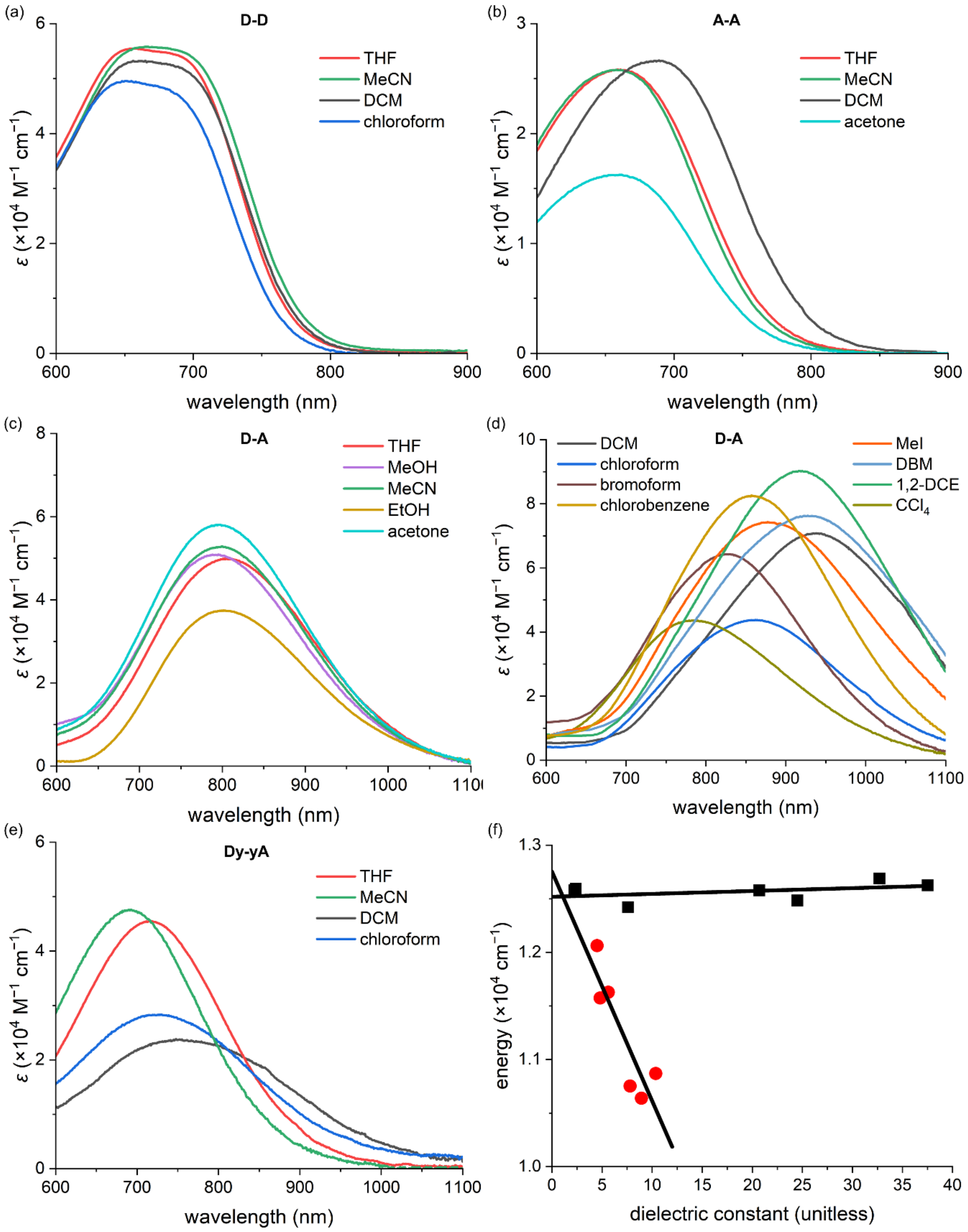

Figure S39 UV-vis absorption solvatochromism of (a) **D-D**, (b) **A-A**, (c) **D-A** in non-halogenated solvents, (d) **D-A** in halogenated solvents and (e) linear fits of solvatochromic behavior of **D-A** in non-halogenated solvents (black squares) and halogenated solvents (red dots). The intercept dielectric constant of the two linear fit is 1.08.

To further characterize the abnormal solvatochromic behavior, we fitted the energies of the maxima of the closed-form absorption in different solvents to a simplified version of the Kamlet-Abboud-Taft (KAT) solvatochromic model, a linear solvation energy relationship (LSER).[15-17] The KAT model is a multi-regression analytic model with the general **Equation S5**, where v is the observed energy of the absorption maximum in different solvents, $v_{max,0}$ is the energy of the absorption maximum in vacuum obtained from linear regression in different solvents, α is a measure of hydrogen bond donor acidity and describes the ability of the solvent to donate a proton in a solvent-to-solute hydrogen bond. The β value is a measure of hydrogen bond acceptor basicity and describes a solvent's ability to accept a proton in a solute-to-solvent hydrogen bond. The π^* value is an index of dipolarity and polarizability of the solvent and measures the ability of the solvent to stabilize a charge or a dipole. The δ term is the discontinuous polarizability correction term. The correction term is incorporated because the polarizability of different classes of solvents varies significantly. The discontinuous polarizability correction term is 1 for aromatic solvents, 0.5 for chlorinated aliphatic solvents and 0 for other solvents. Despite assigning δ values to different classes of solvents for correction, there is currently no clear physical interpretation why aromatic and halogenated solvents deviate from others. The δ_H (Hildebrand solubility) and ξ (coordinate valency) terms in the original KAT model are neglected because the δ_H term is a measure of the solvent/solvent interactions that are interrupted in creating a cavity for the solute and is most relevant for the solvation of polymers while the ξ term provide a measure of basicity in presence of C=O, S=O, P=O, pyridine, or amine groups.[15] The α, β, π^* and δ parameters are published for THF, MeOH, MeCN, EtOH, acetone, CH_2Cl_2 and chloroform **(Table S8)** and are used directly in our analysis.[15]

$$v_{max} = v_{max,0} + a\alpha + b\beta + s(\pi^* + d\delta) \qquad \textbf{(S5)}$$

Table S8 α, β, π* and δ values of selected solvents for the KAT model analysis.[15]

Solvent	α	β	π^*	δ
THF	0.00	0.55	0.58	0
MeOH	0.93	0.62	0.60	0
MeCN	0.19	0.31	0.75	0
EtOH	0.83	0.77	0.54	0
acetone	0.08	0.48	0.71	0
CH_2Cl_2	0.30	0.00	0.82	0.5
chloroform	0.44	0.00	0.58	0.5

Table S9 Multi regression results of the KAT model.

Term	$v_{max,0}$ (cm^{-1})	a (cm^{-1})	b (cm^{-1})	s (cm^{-1})	d
Value	12600±300	14±500	−300±800	−300±1400	13±4

The multi-regression analysis yields the results as shown in **Table S9**. Small *a*, *b* and *s* values close to zero with significantly greater error ranges indicate that the abnormal solvatochromism is irrelevant to hydrogen-bond donation, hydrogen-bond acceptance, dipolarity or polarizability of the solvent. The value of *d* is expected to be zero when only one class of solvents is considered and small ($|d| < 0.5$) and negative when different classes of solvents are considered together. The significantly greater value of *d* than what has commonly been reported in the literature suggests that it is not the polarizability but a specific interaction between the solvent and the molecules which leads to the abnormal solvatochromism. After ruling out hydrogen bonding, solvent polarity and polarizability, the most likely explanation to the abnormal solvatochromism is a specific halogen-bonding interaction between the solvent and the molecule. Halogen bonding can potentially be formed between the hexafluorophosphate anion and the α-hole of the halogenated solvents. Such an interaction would weaken the ion pairing between the hexafluorophosphate and the *N*-methylpyridinium, leading to stronger intramolecular charge transfer in **D-A**. Similarly, a stronger charge transfer from the central thiophene to the *N*-methylpyridinium is established in **A-A**. Increasing the extent of intramolecular charge transfer between the donor and the acceptor will lower the energy of HOMO and leads to the bathochromic shift of the absorption spectrum.

S13. Functional and Basis Set Benchmarking for DFT and TD-DFT Calculations

To rationalize the observed trend in the experimentally-measured UV-vis absorption spectra of the DTEs, we performed density functional theory (DFT) calculations geometry optimization and time-dependent density functional theory (TD-DFT) calculations for calculating the vertical transition energies of all compounds. Note that these vertical transition energies do not account for vibronic coupling, which may not be insignificant. All calculations were carried out using the Gaussian 16 software package.

Ground state geometry benchmarking was carried out with **D-D**, **A-A**, and **D-A** (both open- and closed-forms) as the prototype DTEs. Calculations were done in the gas phase, and numerical frequency calculations were used to confirm stationary points as minima. Benchmarked geometries were calculated at B3LYP/Def2SVP, PBE0/Def2SVP, ωB97X-D/Def2SVP, and tuned CAM-B3LYP/Def2SVP levels of theory. B3LYP and PBE0 are two hybrid functionals without long-range correction. Tuned CAM-B3LYP ($\mu = 0.150$, $\alpha = 0.0799$ and $\beta = 0.9201$) with long-range correction was adopted from a report published by Okuno and co-workers,[18] which demonstrated that CAM-B3LYP with the tuned parameters could semi-quantitatively

reproduce several excitation energies obtained from the experimental UV-vis spectra of 15 closed-form DTE derivatives. The ωB97X-D functional includes both long-range correction and empirical dispersion. The GD3BJ empirical dispersion was applied to the functionals without empirical dispersion, i.e. B3LYP, PBE0 and tuned CAM-B3LYP, during both DFT and TD-DFT calculations.

The output geometries were aligned and RMSD values calculated between each pair of optimized geometries of **D-D**, **A-A**, and **D-A** were calculated and are summarized in **Table S10**, **Table S11**, and **Table S12**. An example of aligned structures is shown in **Figure S40**.

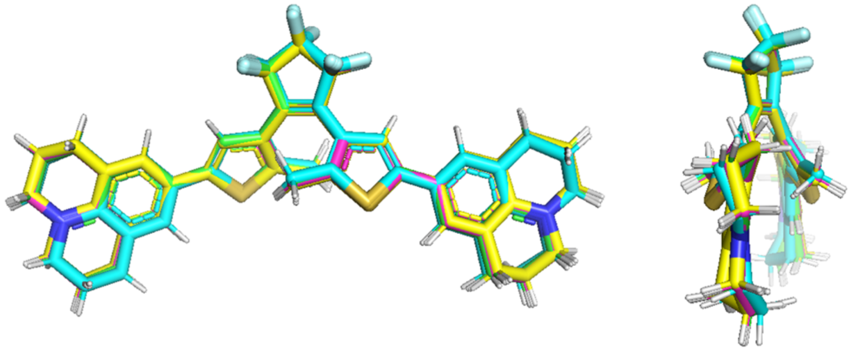

Figure S40 Front (left) and side view (right) of superposed geometries of open-form **D-D** optimized at B3LYP/Def2SVP (pink), PBE0/Def2SVP (green), tuned CAM-B3LYP/Def2SVP (yellow), and ωB97X-D/Def2SVP (blue) levels of theory. Hydrogen, fluorine, sulfur, and nitrogen atoms are shown in white, light blue, dark yellow and dark blue, respectively.

Table S10 Summary of RMSD values between each pair of optimized geometries of **D-D** (open) and **D-D** (closed).

**D-D** (open)					**D-D** (closed)				
	B3LYP	PBE0	CAM	ωB97X-D		B3LYP	PBE0	CAM	ωB97X-D
B3LYP	/	/	/	/	B3LYP	/	/	/	/
PBE0	0.059	/	/	/	PBE0	0.080	/	/	/
CAM	0.079	0.131	/	/	CAM	0.078	0.053	/	/
ωB97X-D	0.280	0.235	0.340	/	ωB97X-D	0.452	0.397	0.420	/

Table S11 Summary of RMSD values between each pair of optimized geometries of **A-A** (open) and **A-A** (closed).

**A-A** (open)					**A-A** (closed)				
	B3LYP	PBE0	CAM	ωB97X-D		B3LYP	PBE0	CAM	ωB97X-D
B3LYP	/	/	/	/	B3LYP	/	/	/	/
PBE0	0.046	/	/	/	PBE0	0.057	/	/	/
CAM	0.104	0.140	/	/	CAM	0.045	0.050	/	/
ωB97X-D	0.091	0.063	0.191	/	ωB97X-D	0.196	0.177	0.197	/

Table S12 Summary of RMSD values between each pair of optimized geometries of **D-A** (open) and **D-A** (closed).

**D-A** (open)					**D-A** (closed)				
	B3LYP	PBE0	CAM	ωB97X-D		B3LYP	PBE0	CAM	ωB97X-D
B3LYP	/	/	/	/	B3LYP	/	/	/	/
PBE0	0.091	/	/	/	PBE0	0.057	/	/	/
CAM	0.214	0.251	/	/	CAM	0.036	0.045	/	/
ωB97X-D	0.305	0.230	0.346	/	ωB97X-D	0.040	0.054	0.061	/

The RMSD values demonstrate that the geometries are fairly similar between functionals, except for those calculated with ωB97X-D, which tend to have larger deviations. Analysis of the structures showed the dihedral angle between the central perfluorinated five-membered ring and the thiophene ring (C-T dihedral), as well as the dihedral angle between the thiophene ring and the aromatic ring attached to thiophene (T-A dihedral) as the main sources of the geometry deviation (**Figure S41)**. The average C-T and T-A dihedral angles of each optimized geometry was measured and summarized in **Table S13**, **Table S14**, and **Table S15**. Geometries optimized with ωB97X-D functional have a larger T-A dihedral angle than the geometries optimized with other functionals, which better resembles the angles in X-ray crystallographic data of several reported DTEs.[19-21]

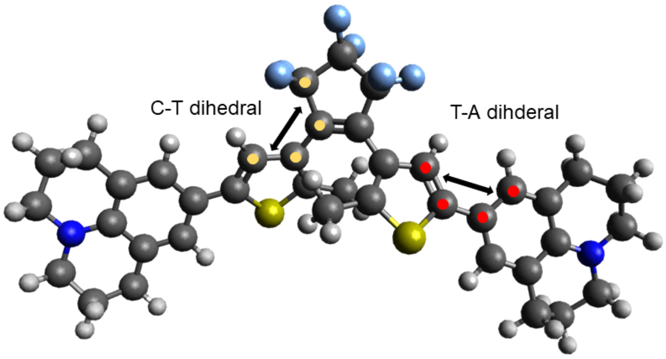

Figure S41 Optimized geometry of **D-D** at B3LYP/Def2SVP level of theory in Avogadro software for measuring the dihedral angles.

Table S13 Summary of average C-T and T-A dihedral angles of optimized **D-D** geometry with different functionals.

**D-D** (open)					**D-D** (closed)				
	B3LYP	PBE0	CAM	ωB97X-D		B3LYP	PBE0	CAM	ωB97X-D
Avg. C-T /°	44.0	44.3	41.8	47.5	Avg. C-T /°	7.6	7.0	7.2	6.3
Avg. T-A /°	18.8	16.1	20.2	27.6	Avg. T-A /°	1.2	1.8	2.8	15.9

Table S14 Summary of average C-T and T-A dihedral angles of optimized **A-A** geometry with different functionals.

**A-A** (open)					**A-A** (closed)				
	B3LYP	PBE0	CAM	ωB97X-D		B3LYP	PBE0	CAM	ωB97X-D
Avg. C-T /°	40.5	41.0	37.6	43.3	Avg. C-T /°	8.4	7.8	7.8	7.0
Avg. T-A /°	1.2	1.2	0.5	2.1	Avg. T-A /°	4.9	6.4	5.7	17.1

Table S15 Summary of average C-T and T-A dihedral angles of optimized **D-A** geometry with different functionals.

**D-A** (open)					**D-A** (closed)				
	B3LYP	PBE0	CAM	ωB97X-D		B3LYP	PBE0	CAM	ωB97X-D
Avg. C-T /°	45.0	45.2	40.9	46.9	Avg. C-T /°	11.1	10.7	11.0	10.8
Avg. T-A /°	6.1	8.4	5.4	13.8	Avg. T-A /°	1.0	0.8	1.0	1.4

We further analyzed the impact of different optimized ground-state geometries on the predicted transitions with TD-DFT calculations. For all optimized ground-state geometries, TD-DFT was performed at tuned CAM-B3LYP/Def2SVP level of theory with acetonitrile Solvation Model based on Density (SMD), using GD3BJ empirical dispersion. The resulting transition data were analyzed with Gaussian broadening (35 nm, Chemcraft software) to simulate the full width at half maximum (FWHM) of experimental absorption spectra, and the peak maximum was compared between the different geometries. The experimental and calculated absorption maxima are summarized in **Table S16**, **Table S17**, and **Table S18**. We found that the different ground-state geometries of open-form DTEs have little impact on the TD-DFT calculations while significant impact is observed in the closed-forms. Different ground-state geometries led to > 60 nm difference for closed-form **D-D** and >100 nm difference for closed-form **A-A,** moreover, TD-DFT at tuned CAM-B3LYP/Def2SVP level of theory yields very poor prediction of absorption spectra for closed-form **A-A** and **D-A**. As ωB97X-D-optimized ground-state geometry most closely resemble literature crystal data,[19-21] and gave TD-DFT results closer to literature values, we chose the ωB97X-D functional for the geometry optimization of all other DTEs.

Table S16 Calculated absorption maxima (tuned CAM-B3LYP/Def2SVP) of ground-state geometries obtained from geometry optimization with different functionals, and the experimental absorption maxima of **D-D**.

**D-D** (open)					**D-D** (closed)				
	B3LYP	PBE0	CAM	ωB97X-D		B3LYP	PBE0	CAM	ωB97X-D
Calc. /nm	358.1	354.6	359.6	342.9	Calc. /nm	720.2	712.9	696.3	653.6
Exp. /nm	346				Exp. /nm	667			

Table S17 Calculated absorption maxima (tuned CAM-B3LYP/Def2SVP) of ground-state geometries obtained from geometry optimization with different functionals, and the experimental absorption maxima of **A-A**.

**A-A** (open)					**A-A** (closed)				
	B3LYP	PBE0	CAM	ωB97X-D		B3LYP	PBE0	CAM	ωB97X-D
Calc. /nm	366.6	364.1	365.8	350.0	Calc. /nm	847.7	837.4	797.0	723.6
Exp. /nm	353				Exp. /nm	658			

Table S18 Calculated absorption maxima (tuned CAM-B3LYP/Def2SVP) of ground-state geometries obtained from geometry optimization with different functionals, and the experimental absorption maxima of **D-A**.

**D-A** (open)					**D-A** (closed)				
	B3LYP	PBE0	CAM	ωB97X-D		B3LYP	PBE0	CAM	ωB97X-D
Calc. /nm	369.2	364.5	369.9	353.2	Calc. /nm	1077	1071	1063	1045
Exp. /nm	350				Exp. /nm	792			

S14. Comparison of ωB97X-D and CAM-B3LYP Functionals for Spectral Calculation of DTEs

After benchmarking the ground-state geometry, we decided to employ the tuned CAM-B3LYP and ωB97X-D functionals to calculate the spectroscopic features of all eight compounds in both isomeric forms (open-form and closed-form) because the long-range correction of these two functionals are important for properties influenced by charge transfer. The Def2SVP basis set was used throughout the calculation. The transitions were calculated at three combinations of functionals, including ωB97X-D/ωB97X-D (where both geometry optimization and TD-DFT calculation were performed with ωB97X-D), ωB97X-D/CAM-B3LYP (where geometry optimization was performed with ωB97X-D and TD-DFT was performed with tuned CAM-B3LYP), and CAM-B3LYP/CAM-B3LYP (where both geometry optimization and TD-DFT calculation were performed with tuned CAM-B3LYP). The calculated vertical transition energies are summarized in **Table S19**, where CAM represents the tuned CAM-B3LYP functional. NOTE: the calculated vertical transition energies do not take into account vibronic effects, and therefore are strictly equivalent to the (0,0) transition, rather than λ_{max}. **Figure S42** shows the comparison between calculated vertical transition energies and experimental excitation wavelengths of all DTEs in both isomers.

Table S19 Calculated wavelengths (nm) of absorption maxima of DTEs at three different combinations of functionals.

DTE	isomer	CAM/CAM	ωB97X-D/ωB97X-D	ωB97X-D/CAM
**D-D**	open	360	312	343
	closed	696	580	654
**A-A**	open	369	332	359
	closed	797	616	724
**D-A**	open	370	328	353
	closed	1063	975	1045
**Dy-yD**	open	389	347	379
	closed	745	613	697
**Ay-yA**	open	406	360	395
	closed	825	640	751
**Dy-yA**	open	406	360	393
	closed	1125	827	957
**Dy-A**	open	390	348	379
	closed	1109	926	1035
**D-yA**	open	410	368	402
	closed	1111	911	1023

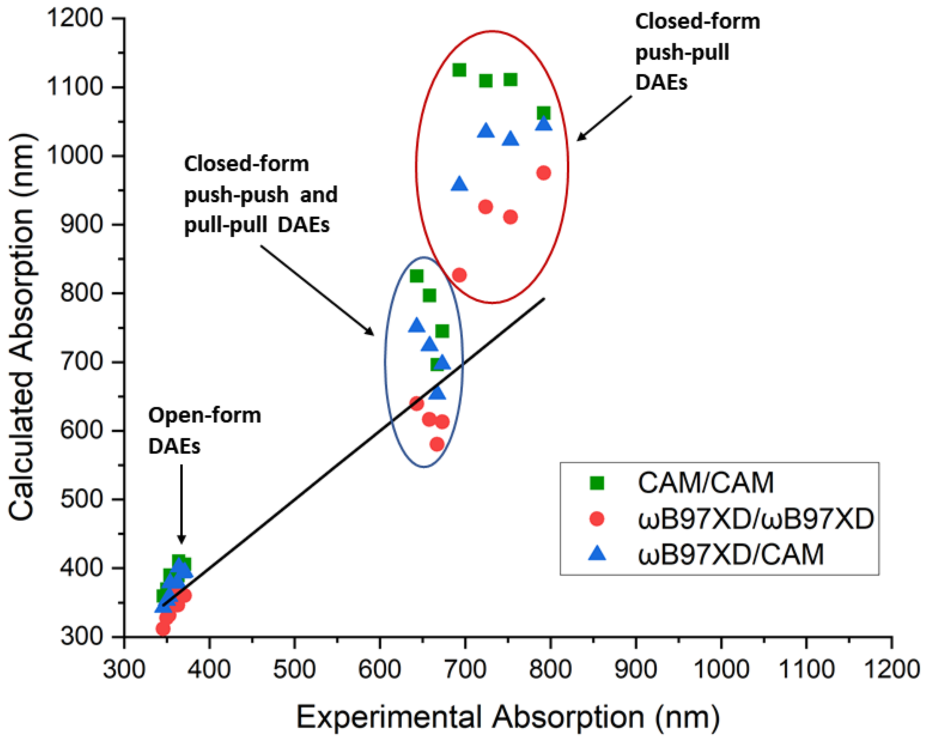

Figure S42. Comparison between the experimental and theoretical absorption maxima wavelengths of the DTEs in their open-form and closed-form isomers calculated by tuned CAM-B3LYP/tuned CAM-B3LYP, ωB97X-D/ωB97X-D, and ωB97X-D/tuned CAM-B3LYP functionals with Def2SVP basis set.

Overall, according to **Figure S42**, we demonstrate that all functional combinations for geometry optimization and TD-DFT give good estimation of the absorption maxima of open-form DTEs. For the closed-form DTEs, the theoretical prediction on push-push and pull-pull DTEs show moderate agreement with the

experimental data but the stabilization of intramolecular charge transfer is significantly overestimated. Comparing the three combinations of functionals, we find that geometry optimization at ωB97X-D/Def2SVP level of theory followed by TD-DFT calculation at the same level of theory yields the theoretical absorption maxima wavelengths that best match the experimental data.

S15. Electron Density Difference Plot between Ground and Excited State

To visualize the intramolecular charge transfer state of the closed-form DTEs that leads to the significant bathochromic shift of UV-vis absorption spectrum and rationalize the insulating property of alkynes in the backbone, we calculated SCF (ground-state) and CI (excited-state) density of **D-D**, **A-A**, **D-A** and **Dy-yA**. The electron density difference plot (EDD) was generated by subtracting the SCF density from the CI density. The resulting density difference was then mapped onto the isosurface of SCF density to generate the EDD plot (with a contour value of 0.0008) shown in **Figure S43**.

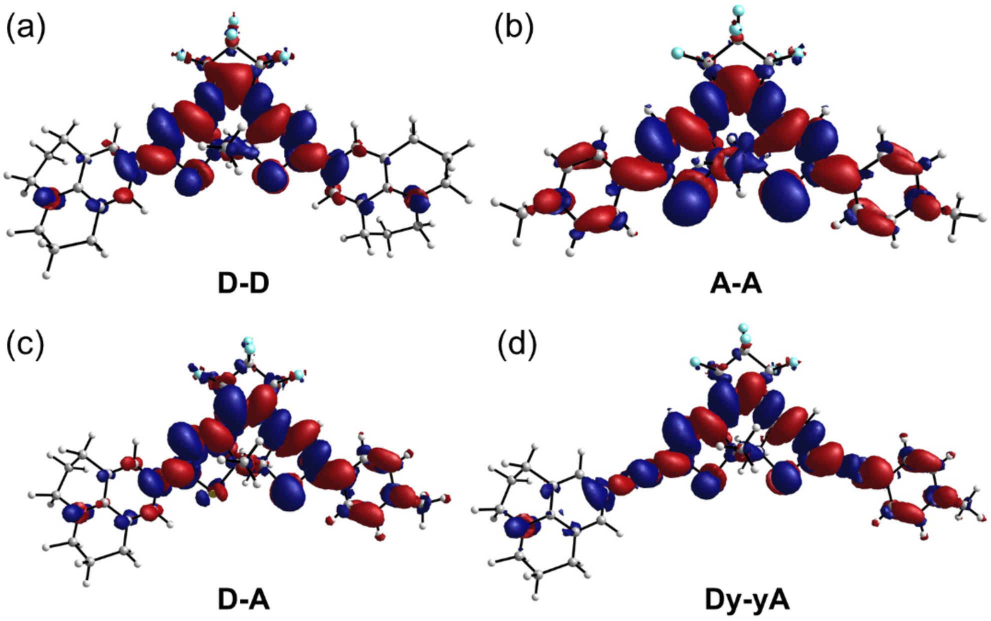

Figure S43. Electron density difference plot between the ground and the excited state of **D-D**, **A-A**, **D-A**, and **Dy-yA**. The red and blue colors indicate decreased and increased electron density during the transition, respectively.

S16. Conformational Analysis on the DTEs

Conformational searches of molecules **A-A**, **D-A**, **D-D**, and **Dy-yA** were performed using CREST, starting from a parallel-1 geometry input.[28] Six different conformers were generated for **A-A** and they were manually grouped into the three main types (anti-parallel, parallel 1 and parallel 2 - **Figure S44**, two conformers for each category). Significantly more conformers were generated with the incorporation of julolidine (e.g. 40 for **D-A** and 74 for **D-D**) due to the flexible julolidine aliphatic side chain. Since the fluctuation in julolidine is small (3 kJ/mol for an inversion of the CH_2 in julolidine) and equally common across all three categories of conformation, we neglected the impact of such displacements in julolidine groups. Among the three main conformation groups **(Figure S44)**, anti-parallel conformers and parallel 2 conformers exist as mirror-imaged pairs. Parallel 1 conformer consists of two mirror-imaged pairs if the two arms are different. Geometry optimizations were performed on the representative structures (anti-parallel, parallel 1 and parallel 2) at the ωB97X-D/def2-SVP level of theory with SMD solvent model (acetonitrile). The Boltzmann distributions of each molecule at different conformations **(Table S20)** were calculated with the sum of electronic and zero-point energies as well as the sum of electronic and thermal free energies (accounting for entropic contributions, **Table S21**) by the equation shown as **Equation S6**, where p_i is the probability of state i, g_i is the number of degenerate states (mirror-image), ε_i is the energy of state i, k is the Boltzmann constant, T is the absolute temperature, M is the number of all states accessible to the system of interest, and Q is the normalization denominator.

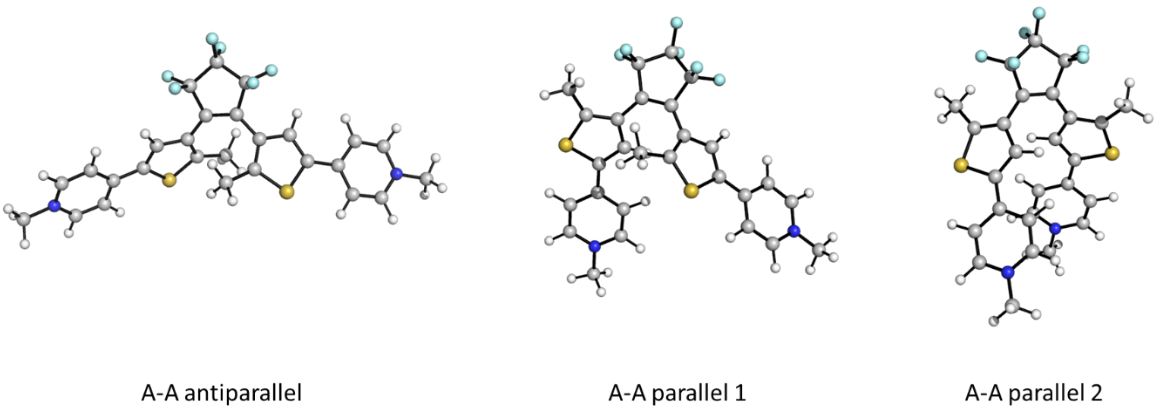

Figure S44. Representative **A-A** structures for the three main conformers of the studied DTEs.

$$p_i = \frac{1}{Q} \cdot e^{-\frac{\varepsilon_i}{kT}} = \frac{g_i e^{-\frac{\varepsilon_i}{kT}}}{\sum_{j=1}^{M} g_j e^{-\frac{\varepsilon_j}{kT}}} \quad \textbf{(S6)}$$

Table S20 Relative energy (calculated from the sum of electronic and zero-point energies) and Boltzmann distribution (%) of different conformers of **D-D**, **A-A**, **D-A** and **Dy-yA**. The values in the bracket denote the number of degenerate structures, *M*, used in Boltzmann distribution calculation.

	**D-D**		**A-A**		**D-A**		**Dy-yA**	
	energy (kJ/mol) (*M*)	distribution (%)	energy (kJ/mol) (*M*)	distribution (%)	energy (kJ/mol) (*M*)	distribution (%)	energy (kJ/mol) (*M*)	distribution (%)
anti-parallel	5.3 (2)	10.3%	0 (2)	>99.9%	12.4 (2)	0.5%	8.2 (2)	3.4%
parallel 1	16.8 (2)	0.2%	25.4 (2)	<0.1%	16.0 (4)	0.4%	16.2 (4)	0.2%
parallel 2	0 (2)	89.5%	/[a]	0%	0 (2)	99.1%	0 (2)	96.4%

[a] local energy minimum was not found for **A-A** in parallel 2 conformation.

Table S21 Relative energy (calculated from the sum of electronic and thermal free energies) and Boltzmann distribution (%) of different conformers of **D-D**, **A-A**, **D-A** and **Dy-yA**. The value in the bracket denote the number of degenerate structures being accounted for Boltzmann distribution calculation.

	**D-D**		**A-A**		**D-A**		**Dy-yA**	
	energy (kJ/mol) (*M*)	distribution (%)	energy (kJ/mol) (*M*)	distribution (%)	energy (kJ/mol) (*M*)	distribution (%)	energy (kJ/mol) (*M*)	distribution (%)
anti-parallel	4.9 (2)	12.1%	0 (2)	>99.9%	11.2 (2)	0.8%	7.1 (2)	3.9%
parallel 1	14.2 (2)	0.6%	20.8 (2)	<0.1%	12.2 (4)	1.4%	9.9 (4)	3.1%
parallel 2	0 (2)	87.3%	/[a]	0%	0 (2)	97.8%	0 (2)	93.0%

[a] local energy minimum was not found for **A-A** in parallel 2 conformation.

S17. Synthetic Procedures

3-Bromo-5-chloro-2-methylthiophene (1b)

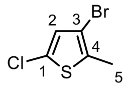

This compound was prepared from **1a** using a procedure published by Lucas and co-workers,[22] yielding a pale-yellow oil (10.1 g, 63%). δ_H (400 MHz, $CDCl_3$): 6.73 (1H, s, C(2)H), 2.32 (3H, s, C(5)H); δ_C (101 MHz, $CDCl_3$): 133.3, 128.6, 127.0, 107.7, 14.9.

3,3'-(Perfluorocyclopent-1-ene-1,2-diyl)bis(5-chloro-2-methylthiophene) (1)

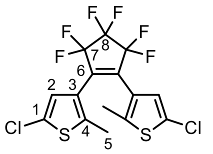

This compound was prepared using a procedure published by Hermes and co-workers,[2] yielding a brown solid (5.97 g, 64%). δ_H (400 MHz, $CDCl_3$): 6.88 (2H, s, C(2)H), 1.89 (6H, s, C(5)H); δ_C (101 MHz, $CDCl_3$): 140.7, 128.2, 125.7, 124.2, 14.5, C(6), C(7) and C(8) were not observed because the carbon signals are weak as a consequence of C-F coupling; δ_F (376 MHz, $CDCl_3$): −110.3 (4F, t, $^3J_{FF}$ 5.4, C(7)F), −131.9 (2F, p, $^3J_{FF}$ 5.4, C(8)F).

4-Bromojulolidine (3b)

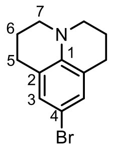

The compound **3b** was prepared following a published procedure[3] with modifications in the purification method. To a solution of julolidine (2.24 g, 12.9 mmol) in MeCN (40 mL) under inert atmosphere at 0 °C was slowly added a solution of NBS (2.53 g, 14.2 mmol, 1.1 equiv.) in MeCN (15 mL). The reaction mixture was warmed to room temperature and stirred for 3 h. After confirming the total consumption of julolidine by TLC (CH_2Cl_2), the mixture was poured into water, basified with a saturated solution of sodium bicarbonate, and extracted with CH_2Cl_2. The organic extracts were combined, washed with brine, dried with $MgSO_4$, and concentrated *in vacuo* to give the crude product as a red-wine-colored oil. The crude compound was purified by flash column chromatography (CH_2Cl_2) to yield the product as a red-wine-colored oil (1.99 g, 61%). δ_H (400 MHz, $CDCl_3$): 6.88 (2H, s, C(3)H), 3.11 (4H, t, $^3J_{HH}$ 5.8, C(7)H), 2.71 (4H, t, $^3J_{HH}$ 6.6, C(5)H), 1.94 (4H, tt, $^3J_{HH}$ 5.8, $^3J_{HH}$ 6.6, C(6)H); δ_C (101 MHz, $CDCl_3$): 142.0, 129.3, 123.7, 107.3, 49.9, 27.6, 21.9.

9-(4,4,5,5-Tetramethyl-1,3,2-dioxaborolan-2-yl)-2,3,6,7-tetrahydro-1*H*,5*H*-pyrido[3,2,1-*ij*]quinolone (3)

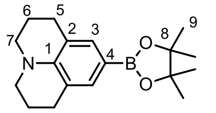

Compound **3** was prepared following a published procedure[4] with modification in the catalytic system. Dry dioxane and triethylamine were degassed by three cycles of freeze-pump-thaw. A solution of **3b** (6.00 g, 23.8 mmol), $Pd(OAc)_2$ (267 mg, 1.19 mmol, 5 mol%), XPhos (1.13 mg, 2.38 mmol, 10 mol%) in dioxane (200 mL) and triethylamine 40 mL) was stirred at 80 °C for 10 min before adding pinacolborane (13.8 mL, 95.2 mmol, 4 equiv.). The reaction mixture was further stirred at 80 °C for 20

h. After confirming the full consumption of **5** by TLC (CH_2Cl_2), the reaction mixture was cooled to room temperature, concentrated *in vacuo*, dispersed in water (20 mL) and extracted with hexane/EtOAc (1:1). The crude product was purified by flash column chromatography (EtOAc/hexane 1:20) to yield the product as a white solid (5.81 g, 82%). δ_H (400 MHz, $CDCl_3$): 7.25 (2H, s, C(3)H), 3.17 (4H, t, $^3J_{HH}$ 5.4, C(7)H), 2.74 (4H, t, $^3J_{HH}$ 6.3, C(5)H), 1.94 (4H, tt, $^3J_{HH}$ 5.4, $^3J_{HH}$ 6.3, C(6)H), 1.31 (12H, s, C(9)H); δ_C (101 MHz, $CDCl_3$): 145.5, 133.8, 120.4, 83.1, 50.1, 27.7, 24.9, 22.0, C(4) was not observed due to coupling with ^{10}B and ^{11}B; δ_B (128 MHz, $CDCl_3$): 31.3 (C(4)B).

9,9'-((Perfluorocyclopent-1-ene-1,2-diyl)bis(5-methylthiophene-4,2-diyl))bis(2,3,6,7-tetrahydro-1*H*,5*H*-pyrido[3,2,1-*ij*]quinoline) (D-D)

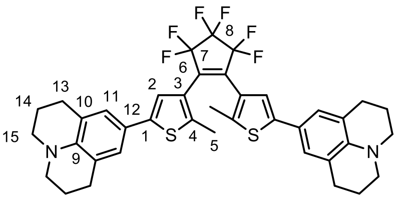

1 (200 mg, 0.457 mmol), **3** (300 mg, 1.00 mmol), $Pd(PPh_3)_4$ (53.0 mg, 0.0458 mmol, 10 mol%), XPhos (50.0 mg, 0.105 mmol), and Cs_2CO_3 (1.0 g, 3.1 mmol) were added to the reaction vessel. Dioxane and water were degassed by three cycles of freeze-pump-thaw. To the mixture of solids under an argon atmosphere was added dioxane (10 mL) and water (2.5 mL). The reaction mixture was heated at reflux for 48 h before being allowed to cool to room temperature. The reaction mixture was poured into water (50 mL) and extracted with Et_2O. The organic extracts were combined, washed with brine, dried ($MgSO_4$), and concentrated *in vacuo* to yield the crude product as a brown oil. The crude product was purified by flash column chromatography (PE/EtOAc 19:1) and precipitated in a mixture of CH_2Cl_2 and PE to yield the final product as a dark green solid (97 mg, 37%). δ_H (400 MHz, $CDCl_3$): 7.05 (2H, s, C(2)H), 6.98 (4H, s, C(11)H), 3.17 (8H, t, $^3J_{HH}$ 5.8, C(15)H), 2.78 (8H, t, $^3J_{HH}$ 6.5, C(13)H), 1.99 (8H, tt, $^3J_{HH}$ 5.8, $^3J_{HH}$ 6.5, C(14)H), 1.88 (6H, s, C(5)H); δ_C (101 MHz, $CDCl_3$): 143.4, 142.9, 138.9, 125.8, 124.5, 121.8, 121.0, 119.7, 50.1, 27.8, 22.1, 14.5, C(6), C(7) and C(8) are not observed because the carbon signals are very weak resulting from the C-F coupling; δ_F (376 MHz, $CDCl_3$): −110.0 (4F, t, $^3J_{FF}$ 5.7, C(7)F), −131.9 (2F, p, $^3J_{FF}$ 5.7, C(8)F); HRMS (ESI$^+$) *m/z*: $[M+H]^+$ calcd. for $C_{39}H_{37}N_2F_6S_2^+$ 711.2273; found 711.2272. UV-vis (MeCN): λ_{max} 346 nm (ε = 53000 M^{-1} cm^{-1}).

4,4'-((Perfluorocyclopent-1-ene-1,2-diyl)bis(5-methylthiophene-4,2-diyl))dipyridine (A-A(p))[6, 19, 23-26]

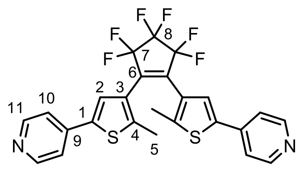

The compound **A-A(p)** was prepared following published procedure with slight modifications in the catalytic system. **1** (300 mg, 0.686 mmol), 4-(4,4,5,5-tetramethyl-1,3,2-dioxaborolan-2-yl)pyridine (563 mg, 2.74 mmol), $Pd(PPh_3)_4$

(79.3 mg, 0.0686 mmol, 10 mol%), XPhos (56.3 mg, 0.137 mmol, 20 mol%), and Cs_2CO_3 (1.12 g, 3.43 mmol) were added to the reaction vessel. Dioxane and water were degassed by three cycles of freeze-pump-thaw. To the dried mixture of solids under an argon atmosphere was added dioxane (20 mL) and water (5 mL). The reaction mixture was heated at reflux for 72 h before being allowed to cool to room temperature. The reaction mixture was poured into water (70 mL) and then extracted with CH_2Cl_2. The organic extracts were combined, washed with brine, dried ($MgSO_4$), and concentrated *in vacuo* to yield the crude product as a brown oil. The crude product was purified by flash column chromatography (PE/EtOAc 1:5, 1% Et_3N) to yield the product as a white solid (141 mg, 39%). With contamination of triphenylphosphine oxide (18% by ^{1}H NMR), the crude product was not further purified. δ_H (400 MHz, $CDCl_3$): 8.61 (4H, d, $^3J_{HH}$ 4.3, C(11)H), 7.47 (2H, s, C(2)H), 7.40 (4H, d, $^3J_{HH}$ 4.3, C(10)H), 1.99 (6H, s, C(5)H); δ_C (101 MHz, $CDCl_3$): 150.7, 143.8, 140.2, 139.5, 126.3, 124.8, 119.7, 14.9, C(6), C(7) and C(8) are not observed because the carbon signals are very weak resulting from the C-F coupling; δ_F (376 MHz, $CDCl_3$): −110.1 (4F, t, $^3J_{FF}$ 5.3, C(7)F), −131.8 (2F, p, $^3J_{FF}$ 5.3, C(8)F).

4,4'-((Perfluorocyclopent-1-ene-1,2-diyl)bis(5-methylthiophene-4,2-diyl))bis(1-methylpyridin-1-ium) bishexafluorophosphate (A-A)[6, 24-26]

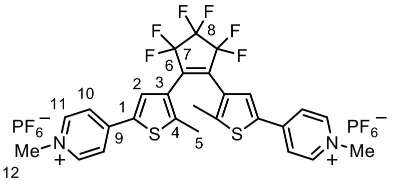

To a solution of **A-A(p)** (260 mg, 0.498 mmol) in CH_2Cl_2 (15 mL) was added iodomethane (1.50 mL, 24.1 mmol). The reaction mixture was stirred at room temperature for 28 h and the precipitate in the reaction mixture was filtered off. The precipitate was dissolved in methanol (100 mg, 0.129 mmol) and then added to a solution of potassium hexafluorophosphate (8.0 g, 43.46 mmol) in water (100 mL). The solution was stirred at room temperature for 1 h for ion exchange and the precipitate was filtered off to yield the final product as a pale green solid (110 mg, 95%). δ_H (400 MHz, CD_3CN): 8.52 (4H, d, $^3J_{HH}$ 7.1, C(11)H), 8.06 (4H, d, $^3J_{HH}$ 7.1, C(10)H), 8.01 (2H, s, C(2)H), 4.22 (6H, s, C(12)H), 2.11 (6H, s, C(5)H); δ_C (101 MHz, $CDCl_3$): 151.3, 148.6, 146.2, 136.4, 132.0, 127.6, 123.5, 48.4, 15.4, C(6), C(7) and C(8) are not observed because the carbon signals are very weak resulting from the C-F coupling; δ_F (376 MHz, CD_3CN): −72.9 (6F, d, $^1J_{FP}$ 705.7, hexafluorophosphate), −110.9 (4F, t, $^3J_{FF}$ 4.9, C(7)F), −132.4 (2F, p, $^3J_{FF}$ 4.9, C(8)F). HRMS (ESI$^+$) *m/z*: $[M]^{2+}$ calcd. for $C_{27}H_{22}F_6N_2S_2^{2+}$ 276.0559; found 276.0558. UV-vis (MeCN): λ_{max} 353 nm (ε = 661200 M^{-1} cm^{-1}).

9-(4-(3,3,4,4,5,5-Hexafluoro-2-(2-methyl-5-(pyridin-4-yl)thiophen-3-yl)cyclopent-1-en-1-yl)-5-methylthiophen-2-yl)-2,3,6,7-tetrahydro-1*H*,5*H*-pyrido[3,2,1-*ij*]quinoline (D-A(p))

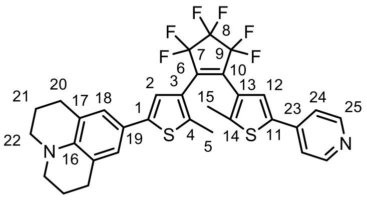

1 (1.53 g, 3.50 mmol), **3** (1.10 g, 3.67 mmol, 1.05 equiv.), 4-(4,4,5,5-tetramethyl-1,3,2-dioxaborolan-2-yl)pyridine (753 mg, 3.67 mmol, 1.05 equiv.), $Pd(PPh_3)_4$ (202 mg, 175 μmol, 5 mol%), XPhos (167 mg, 350 μmol, 10 mol%), and Cs_2CO_3 (4.40 g, 13.5 mmol) were dried under high vacuum for 2 h. Dioxane and water were degassed by three cycles of freeze-pump-thaw. To the dried mixture of solids under an argon atmosphere was added dioxane (100 mL) and water (20 mL). The reaction mixture was heated at reflux for 72 h before being allowed to cool to room temperature. The reaction mixture was concentrated *in vacuo* to remove dioxane and then poured into water and extracted with CH_2Cl_2. The organic extracts were combined, washed with brine, dried ($MgSO_4$), and concentrated *in vacuo* to yield the crude product as a brown oil. The crude product was purified by flash column chromatography (PE/EtOAc 4:1, 1% Et_3N) to yield the product as a green solid (210 mg, 28%). δ_H (400 MHz, $CDCl_3$): 8.60 (2H, d, $^3J_{HH}$ 4.4, C(25)H), 7.47 (1H, s, C(12)H), 7.40 (2H, d, $^3J_{HH}$ 4.4, C(24)H), 7.02 (1H, s, C(2)H), 6.96 (2H, s, C(18)H), 3.17 (4H, t, $^3J_{HH}$ 5.8, C(22)H), 2.76 (4H, t, $^3J_{HH}$ 6.4, C(20)H), 2.00–1.94 (overlapping s and tt, 7H, $^3J_{HH}$ 5.8, $^3J_{HH}$ 6.4, C(15)H and C(21)H), 1.90 (s, 3H, C(5)H); δ_C (101 MHz, $CDCl_3$): 150.7, 144.0, 143.9, 143.0, 140.5, 138.9, 138.8, 126.7, 125.4, 125.1, 124.5, 121.8, 120.6, 119.7, 119.4, 50.1, 27.8, 22.0, 14.9, 14.6, are not observed because the carbon signals are very weak resulting from the C-F coupling; δ_F (376 MHz, $CDCl_3$): −109.8 (2F, t, $^3J_{FF}$ 8.1, C(7)F), −110.2 (2F, t, $^3J_{FF}$ 8.6, C(9)F), −131.9 (4F, tt, $^3J_{FF}$ 8.1, $^3J_{FF}$ 8.6, C(8)F). HRMS (ESI$^+$) *m/z*: $[M+H]^+$ calcd. for $C_{32}H_{27}F_6N_2S_2^+$ 617.1514; found 617.1513.

4-(4-(3,3,4,4,5,5-Hexafluoro-2-(2-methyl-5-(2,3,6,7-tetrahydro-1*H*,5*H*-pyrido[3,2,1-*ij*]quinolin-9-yl)thiophen-3-yl)cyclopent-1-en-1-yl)-5-methylthiophen-2-yl)-1-methylpyridin-1-ium hexafluorophsphate (D-A)

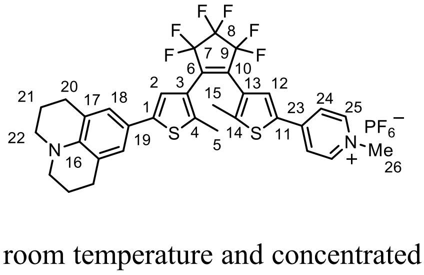

To a solution of **D-A(p)** (100 mg, 162 μmol) in CH_2Cl_2 (10 mL) was added iodomethane (1.00 mL, 16.2 mmol, 100 equiv.). The reaction mixture was stirred at 40 °C for 15 h until the starting material was fully consumed (monitored by MS). The reaction mixture was then cooled to room temperature and concentrated *in vacuo* to yield a green oil. The green oil was dissolved in methanol (2 mL) and then added to a solution of potassium hexafluorophosphate (1.5 g, 8.1 mmol) in water (20 mL). The reaction mixture for ion exchange was stirred at room temperature for 1 h and the precipitation was filtered off

to yield the crude product as a yellow solid. The crude product was further purified by flash column chromatography (CH_2Cl_2/MeOH 95:5) to yield the product as a pale green solid (113 mg, 90%). δ_H (400 MHz, DMSO): 8.89 (2H, d, $^3J_{HH}$ 7.5, C(25)H), 8.36 (2H, d, $^3J_{HH}$ 7.5, C(24)H), 8.30 (1H, s, C(12)H), 7.14 (1H, s, C(2)H), 6.95 (2H, s, C(18)H), 4.26 (3H, s, C(26)H), 3.14 (4H, t, $^3J_{HH}$ 5.7, C(22)H), 2.68 (4H, t, $^3J_{HH}$ 6.4, C(20)H), 2.08 (3H, s, C(15)H), 1.91 (3H, s, C(5)H), 1.86 (4H, tt, $^3J_{HH}$ 5.7, $^3J_{HH}$ 6.4, C(21)H); δ_C (101 MHz, DMSO): 149.0, 146.4, 145.7, 143.7, 142.7, 138.3, 135.2, 131.1, 126.7, 124.2, 123.8, 122.1, 121.1, 119.2, 118.5, 49.2, 47.0, 27.0, 21.3, 14.5, 14.0, C(6), C(7), C(8), C(9) and C(10) are not observed because the carbon signals are very weak resulting from the C-F coupling; δ_F (376 MHz, DMSO): −70.2 (6F, d, $^1J_{FP}$ 710.1, hexafluorophosphate), −109.2 (2F, t, $^3J_{FF}$ 8.9, C(7)F), −109.6 (2F, t, $^3J_{FF}$ 8.1, C(9)F), −131.1 (2F, tt, $^3J_{FF}$ 8.9, $^3J_{FF}$ 8.1, C(8)F). HRMS (ESI$^+$) *m/z*: $[M]^+$ calcd. for $C_{33}H_{29}F_6N_2S_2^+$ 631.1671; found 631.1660. UV-vis (MeCN): λ_{max} 350 nm (ε = 57200 M^{-1} cm^{-1}).

((4-Bromo-5-methylthiophen-2-yl)ethynyl)trimethylsilane (11a).

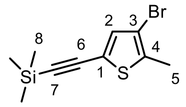

Compound **11a** was synthesized following a published procedure,[5] yielding the product as a white solid (4.69 g, 88%, including by-product) with around 12% (estimated by ^{1}H NMR) contamination by the by-product 1,4-bis(trimethylsilyl)butadiyne from trimethylsilylacetylene homocoupling. The product was not further purified as the by-product does not react in the next step. δ_H (400 MHz, $CDCl_3$): 7.02 (1H, s, C(2)H), 2.36 (3H, s, C(5)H), 0.23 (9H, s, C(8)H); δ_C (101 MHz, $CDCl_3$): 136.5, 135.0, 120.8, 108.9, 99.7, 96.6, 15.0, −0.1.

(((Perfluorocyclopent-1-ene-1,2-diyl)bis(5-methylthiophene-4,2-diyl))bis(ethyne-2,1-diyl))bis(trimethylsilane) (11b)

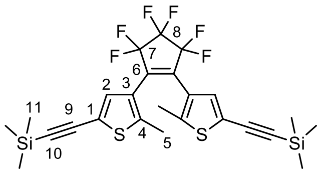

The compound **11** was prepared from a published procedure,[5] yielding a white solid (2.40 g, 45%). δ_H (400 MHz, $CDCl_3$): 7.19 (2H, s, C(2)H), 1.88 (6H, s, C(5)H), 0.25 (18H, s, C(11)H); δ_C (101 MHz, $CDCl_3$): 143.5, 132.2, 124.7, 121.9, 100.2, 96.3, 14.6, −0.1, C(6), C(7) and C(8) are not observed because the carbon signals are very weak resulting from the C-F coupling; δ_F (376 MHz, ($CDCl_3$): −110.4 (4F, t, $^3J_{FF}$ 5.3, C(7)F), −131.9 (2F, p, $^3J_{FF}$ 5.3, C(8)F).

3,3'-(Perfluorocyclopent-1-ene-1,2-diyl)bis(5-ethynyl-2-methylthiophene) (11).[5]

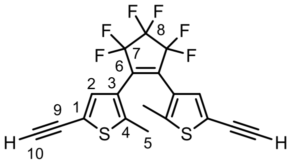

The TMS-deprotection was carried out following a published procedure, yielding a white solid (747 mg, 89%). δ_H (400 MHz, $CDCl_3$): 7.23 (2H, s, C(2)H), 3.35 (2H, s, C(10)H), 1.91 (6H, s, C(5)H); δ_C (101 MHz, $CDCl_3$): 143.8, 132.7, 124.7, 120.8, 82.4, 75.9, 14.5, C(6), C(7) and C(8) are not observed because the carbon signals are very weak resulting from the C-F coupling; δ_F (376 MHz, ($CDCl_3$): −110.4 (4F, t, $^3J_{FF}$ 5.4, C(7)F), −131.9 (2F, p, $^3J_{FF}$ 5.4, C(8)F).

4-Iodojulolidine (5).

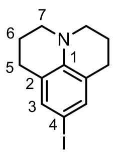

To a solution of julolidine **5a** (2.00 g, 11.5 mmol) in a mixture of 1,4-dioxane (25 mL) and pyridine (25 mL) under an argon atmosphere at 0 °C was slowly added a solution of iodine (5.30 g, 21.0 mmol, 1.8 equiv.) in 1,4-dioxane (10 mL). The reaction mixture was stirred at 0 °C for 2 h before being allowed to warm to room temperature. Iodine (2.93 g, 11.5 mmol, 1 equiv.) was further added and the reaction was stirred at room temperature for 1 h. The reaction mixture was then poured into a saturated aqueous solution of sodium thiosulfate and extracted with CH_2Cl_2. The organic extracts were combined, dried with $MgSO_4$, and concentrated *in vacuo* to give the crude product as a brown oil. The crude compound was purified by flash column chromatography (CH_2Cl_2) to yield the product as a brown oil (2.50 g, 72%). δ_H (400 MHz, $CDCl_3$): 7.06 (2H, s, C(3)H), 3.13 (4H, t, $^3J_{HH}$ 6.1, C(7)H), 2.70 (4H, t, $^3J_{HH}$ 6.8, C(5)H), 1.94 (4H, tt, $^3J_{HH}$ 6.1, $^3J_{HH}$ 6.8, C(6)H); δ_C (101 MHz, $CDCl_3$): 142.5, 135.1, 124.1, 76.2, 49.8, 27.4, 21.8. HRMS (ESI$^+$) *m/z*: $[M+H]^+$ calcd. for $C_{12}H_{15}IN$ 300.0559; found 300.0559.

3,3'-(Perfluorocyclopent-1-ene-1,2-diyl)bis(5-((2,3,7,8,9,9a-hexahydro-1*H*-phenalen-5-yl)ethynyl)-2-methylthiophene) (Dy-yD).

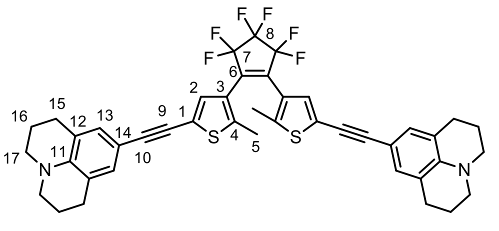

11 (150 mg, 360 µmol), **5** (269 mg, 900 µmol, 2.5 equiv.), $Pd(PPh_3)_4$ (41.6 mg, 36.0 µmol, 10 mol%), and CuI (13.6 mg, 71.3 µmol, 20 mol%) were dried under high vacuum for 30 min. Dry triethylamine was degassed by three cycles of freeze-pump-thaw. To the dried mixture of solids under an argon atmosphere was added triethylamine. The reaction mixture was stirred at 45 °C for 20 h before being allowed to cool to room temperature. Upon completion of the reaction verified by TLC (PE/EtOAc 9:1 with 1% triethylamine), the reaction mixture was concentration *in vacuo* to yield the crude product as a green oil. The crude product was then purified by flash column chromatography

(PE/EtOAc 9:1 with 1% triethylamine) to yield the product as a green solid (244 mg, 56%). δ_H (400 MHz, $CDCl_3$): 7.13 (2H, s, C(2)H), 6.94 (4H, s, C(13)H), 3.19 (8H, t, $^3J_{HH}$ 6.0, C(17)H), 2.71 (8H, t, $^3J_{HH}$ 6.2, C(15)H), 1.95 (8H, tt, $^3J_{HH}$ 6.2, $^3J_{HH}$ 6.0, C(16)H), 1.89 (6H, s, C(5)H); δ_C (101 MHz, $CDCl_3$): 143.4, 142.2, 130.3, 130.0, 124.8, 123.2, 121.1, 107.8, 96.0, 79.1, 50.0, 27.7, 21.8, 14.6, C(6), C(7) and C(8) are not observed because the carbon signals are very weak resulting from the C-F coupling; δ_F (376 MHz, ($CDCl_3$): −110.2 (4F, t, $^3J_{FF}$ 5.2, C(7)F), −131.9 (2F, p, $^3J_{FF}$ 5.2, C(8)F). HRMS (ESI$^+$) *m/z*: $[M+H]^+$ calcd. for $C_{43}H_{36}F_6N_2S_2^+$ 758.2219; found 758.2212. UV-vis (MeCN): λ_{max} 363 nm (ε = 84800 M^{-1} cm^{-1}).

4,4'-(((Perfluorocyclopent-1-ene-1,2-diyl)bis(5-methylthiophene-4,2-diyl))bis(ethyne-2,1-diyl))dipyridine (Ay-yA(p)).

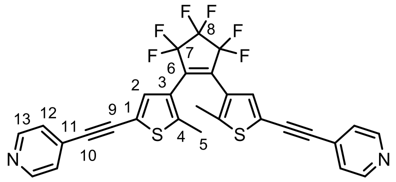

11 (223 mg, 535 μmol), 4-iodopyridine (329 mg, 1.60 mmol, 3 equiv.), $Pd(PPh_3)_4$ (30.9 mg, 26.7 μmol, 5 mol%), and copper(I) iodide (10.2 mg, 53.5 μmol, 10 mol%) were dried under high vacuum for 30 min. Anhydrous triethylamine was degassed by three cycles of freeze-pump-thaw. To the dried mixture of solids under an argon atmosphere was added degassed triethylamine (20 mL). The reaction mixture was stirred at 45 °C for 20 h before being allowed to cool to room temperature. Upon completion of the reaction verified by TLC (PE/EtOAc 4:1 with 1% triethylamine), the reaction mixture was concentration *in vacuo*. The crude product was then purified by flash column chromatography (PE/EtOAc 4:1 with 1% triethylamine) to yield the product as an off-white solid (150 mg, 49%). δ_H (400 MHz, $CDCl_3$): 8.61 (4H, d, $^3J_{HH}$ 4.5, C(13)H), 7.35 (4H, d, $^3J_{HH}$ 4.5, C(12)H), 7.33 (2H, s, C(2)H), 1.97 (6H, s, C(5)H); δ_C (101 MHz, $CDCl_3$): 150.0, 144.9, 132.8, 130.7, 125.2(4), 125.1(5), 120.8, 91.5, 86.0, 14.7, C(6), C(7) and C(8) are not observed because the carbon signals are very weak resulting from the C-F coupling; δ_F (376 MHz, ($CDCl_3$): −110.3 (4F, t, $^3J_{FF}$ 5.0, C(7)F), −131.8 (2F, p, $^3J_{FF}$ 5.0, C(8)F); HRMS (ESI$^+$) *m/z*: $[M+H]^+$ calcd. for $C_{29}H_{17}F_6N_2S_2^+$ 571.0732; found 571.0721.

4,4'-(((Perfluorocyclopent-1-ene-1,2-diyl)bis(5-methylthiophene-4,2-diyl))bis(ethyne-2,1-diyl))bis(1-methylpyridin-1-ium) bishexafluorophosphate (Ay-yA).

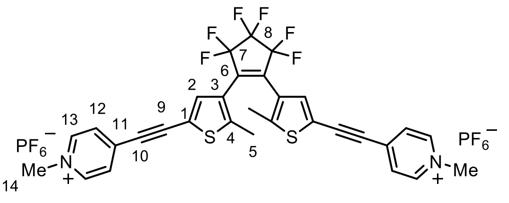

To a solution of **Ay-yA(p)** (55.0 mg, 96.4 μmol) in CH_2Cl_2 (5 mL) was added iodomethane (2.0 mL, 34 mmol). The reaction mixture was stirred at room temperature for 24 h and the precipitation was filtered off. The precipitate was dissolved in

methanol (2 mL) and added to a solution of potassium hexafluorophosphate (4.00 g, 21.7 mmol) in water (50 mL). The solution was stirred at room temperature for 1 h for ion exchange and the precipitate was filtered off to yield the product as a dark green solid (95 mg, 83%). δ_H (400 MHz, CD_3CN): 8.56 (4H, d, $^3J_{HH}$ 6.7, C(13)H), 7.95 (4H, d, $^3J_{HH}$ 6.7, C(12)H), 7.61 (2H, s, C(2)H), 4.26 (6H, s, C(14)H), 2.19 (6H, s, C(5)H); δ_C (101 MHz, CD_3CN): 150.0, 146.2, 140.3, 136.8, 129.7, 126.1, 119.8, 96.3, 90.7, 49.1, 15.1, C(6), C(7) and C(8) are not observed because the carbon signals are very weak resulting from the C-F coupling; δ_F (376 MHz, (CD_3CN): −72.8 (12F, d, $^1J_{FP}$ 707.6, hexafluorophosphate), −110.8 (4F, t, J_{FF} 5.0, C(7)F), −132.3 (2F, p, $^3J_{FF}$ 5.0, C(8)F). HRMS (ESI$^+$) *m/z*: [M^{2+}] calcd. for $C_{31}H_{22}F_6N_2S_2^{2+}$ 300.0559; found 300.0559. UV-vis (MeCN): λ_{max} 371 nm (ε = 67100 M^{-1} cm^{-1}).

9-((4-(3,3,4,4,5,5-Hexafluoro-2-(2-methyl-5-(pyridin-4-ylethynyl)thiophen-3-yl)cyclopent-1-en-1-yl)-5-methylthiophen-2-yl)ethynyl)-2,3,6,7-tetrahydro-1*H*,5*H*-pyrido[3,2,1-*ij*]quinoline (Dy-yA(p)).

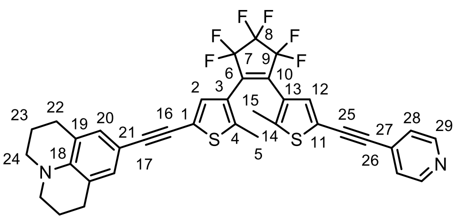

11 (0.395 g, 0.704 mmol, 1 equiv.), 4-iodopyridine (0.159 g, 0.775 mmol, 1.1 equiv.), **5** (0.232 g, 0.775 mmol, 1.1 equiv.), $Pd(PPh_3)_4$ (81.4 mg, 70.4 μmol, 10 mol%), and CuI (26.8 mg, 141 μmol, 20 mol%) were dried under high vacuum for 30 min. Dry triethylamine was degassed by three cycles of freeze-pump-thaw. To the dried mixture of solids under an argon atmosphere was added degassed triethylamine (20 mL). The reaction mixture was stirred at 45 °C for 20 h before being allowed to cool to room temperature. Upon completion of the reaction verified by TLC (PE/EtOAc 4:1 with 1% triethylamine), the reaction mixture was concentrated *in vacuo*. The crude product was then purified by flash column chromatography (PE/EtOAc 4:1 with 1% triethylamine) to yield the product as a dark green solid (150 mg, 32%). δ_H (400 MHz, $CDCl_3$): 8.61 (2H, d, $^3J_{HH.}$ 4.7, C(29)H), 7.35 (2H, d, $^3J_{HH.}$ 4.7, C(28)H), 7.33 (1H, s, C(12)H), 7.12 (1H, s, C(2)H), 6.94 (2H, s, C(20)H), 3.19 (4H, t, $^3J_{HH}$ 5.6, C(24)H), 2.71 (4H, t, $^3J_{HH}$ 6.3, C(22)H), 1.98-1.91 (7H, overlapping s and tt, $^3J_{HH}$ 5.6, $^3J_{HH}$ 6.3, C(15)H and C(23)H), 1.91 (3H, s, C(5)H); δ_C (101 MHz, $CDCl_3$): 150.0, 145.0, 143.5, 142.0, 133.0, 130.8, 130.3, 129.8, 125.4, 125.3, 124.6, 123.6, 121.1, 120.5, 107.6, 96.3, 91.3, 86.2, 78.9, 50.0, 27.7, 21.8, 14.8, 14.6, C(6), C(7), C(8), C(9) and C(10) are not observed because the carbon signals are very weak resulting from the C-F coupling; δ_F (376 MHz, ($CDCl_3$): −110.1 (2F, t, $^3J_{FF}$ 9.8, C(9)F), −110.3 (2F, t, $^3J_{FF}$ 10.3, C(7)F), −131.8 (2F, tt, $^3J_{FF}$ 9.8, $^3J_{FF}$ 10.3, C(8)F). HRMS (ESI$^+$) *m/z*: $[M+H]^+$ calcd. for $C_{36}H_{27}F_6N_2S_2^+$ 665.1514; found 665.1506.

4-((4-(3,3,4,4,5,5-Hexafluoro-2-(2-methyl-5-((2,3,6,7-tetrahydro-1*H*,5*H*-pyrido[3,2,1-*ij*]quinolin-9-yl)ethynyl)thiophen-3-yl)cyclopent-1-en-1-yl)-5-methylthiophen-2-yl)ethynyl)-1-methylpyridin-1-ium hexafluorophosphate (Dy-yA).

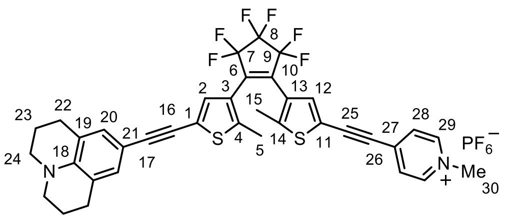

To a solution of **Dy-yA(p)** (200 mg, 312 μmol) in CH_2Cl_2 (20 mL) was added iodomethane (2.0 mL, 32 mmol). The reaction mixture was stirred at room temperature for 20 h and at 45 °C for 4 h before being concentrated *in vacuo*. The crude product was dissolved in methanol and added to a solution of potassium hexafluorophosphate (4.00 g, 21.7 mmol) in water (50 mL). The solution was stirred at room temperature for 1 h for ion exchange and the precipitate was filtered off to yield the crude product as a brown solid. The crude product was further purified by flash column chromatography (CH_2Cl_2/MeOH 95:5) to yield the product as a pale green solid (206 mg, 82%). δ_H (400 MHz, CD_3CN): 8.53 (2H, d, $^3J_{HH}$ 6.8, C(29)H), 7.95 (2H, d, $^3J_{HH}$ 6.8, C(28)H), 7.60 (1H, s, C(12)H), 7.13 (1H, s, C(2)H), 6.86 (2H, s, C(20)H), 4.24 (3H, s, C(30)H), 3.20 (4H, t, $^3J_{HH}$ 5.7, C(24)H), 2.68 (4H, t, $^3J_{HH}$ 6.3, C(22)H), 2.05 (3H, s, C(15)H), 1.96 (3H, s, C(5)H), 1.89 (4H, tt, $^3J_{HH}$ 5.7, $^3J_{HH}$ 6.3, C(23)H); δ_C (101 MHz, CD_3CN): 149.9, 146.0, 144.5, 143.9, 140.3, 136.9, 130.8, 130.6, 129.6, 126.4, 125.1, 124.2, 122.1, 119.4, 107.5, 97.2, 96.2, 90.5, 79.1, 50.4, 49.0, 28.1, 22.2, 15.0, 14.6, C(6), C(7), C(8), C(9) and C(10) are not observed because the carbon signals are very weak resulting from the C-F coupling; δ_F (376 MHz, (CD_3CN): −73.0 (6F, d, $^1J_{FP}$ 708.2, hexafluorophosphate), −110.7 (2F, t, $^3J_{FF}$ 5.1, C(9)F), −110.9 (2F, t, $^3J_{FF}$ 6.3, C(7)F), −132.4 (2F, tt, $^3J_{FF}$ 5.1, $^3J_{FF}$ 6.3, C(8)F). HRMS (ESI$^+$) *m/z*: [M$^+$] calcd. for $C_{37}H_{29}F_6N_2S_2^+$ 679.1642; found 679.1661. UV-vis (MeCN): λ_{max} 369 nm (ε = 72400 M^{-1} cm^{-1}).

3-Bromo-5-ethynyl-2-methylthiophene (4).

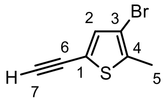

To a solution of **11a** (170 mg, 622 μmol) in a mixture of THF (5 mL) and methanol (5 mL) was added solid potassium carbonate (1.00 g, 7.20 mmol) and the reaction mixture was stirred at room temperature for 30 min. Upon the completion of the reaction verified by TLC (PE), the reaction mixture was poured into water (30 mL) and extracted with CH_2Cl_2. The organic extracts were combined, washed with water, dried ($MgSO_4$), and concentrated *in vacuo*. The crude product was purified by flash column chromatography (PE) to yield the final product as a colorless oil (106 mg, 85%). δ_H (400 MHz, $CDCl_3$): 7.07 (1H, s, C(2)H), 3.33 (1H, s, C(7)H), 2.38 (3H, s, C(5)H); δ_C (101 MHz, $CDCl_3$): 136.9, 135.5, 119.6, 108.9, 82.0, 76.2, 15.0.

9-((4-Bromo-5-methylthiophen-2-yl)ethynyl)-2,3,6,7-tetrahydro-1*H*,5*H*-pyrido[3,2,1-*ij*]quinoline (6).

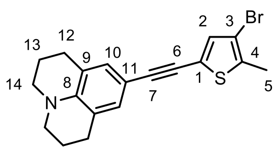

5 (1.00 g, 4.97 mmol), **4** (1.49 g, 4.97 mmol), $Pd(PPh_3)_4$ (575 mg, 497 µmol, 10 mol%), and CuI (189 mg, 995 µmol, 20 mol%) were dried under high vacuum for 30 min. Dry toluene and DIPA were degassed by three cycles of freeze-pump-thaw. To the dried mixture of solids under an argon atmosphere was added degassed toluene (50 mL) and DIPA (50 mL). The reaction mixture was stirred at 75 °C for 23 h before being allowed to cool to room temperature. Upon completion of the reaction verified by TLC (PE/EtOAc 20:1), the reaction mixture was concentration *in vacuo*. The crude product was then purified by flash column chromatography (PE/EtOAc 20:1) to yield the product as an off-white solid (831 mg, 45%). δ_H (400 MHz, $CDCl_3$): 6.94 (1H, s, C(2)H), 6.92 (2H, s, C(10)H), 3.19 (4H, t, $^3J_{HH}$ 5.8, C(14)H), 2.71 (4H, t, $^3J_{HH}$ 6.4, C(12)H), 2.37 (3H, s, C(5)H), 1.94 (4H, tt, $^3J_{HH}$ 5.8, $^3J_{HH}$ 6.4, C(13)H); δ_C (101 MHz, $CDCl_3$): 143.4, 134.9, 132.7, 130.3, 122.4, 121.1, 108.8, 108.0, 95.8, 79.2, 50.0, 27.7, 21.8, 15.0. HRMS (ESI$^+$) *m/z*: $[M+H]^+$ calcd. for $C_{19}H_{13}N_1Br_1S_1$ 372.0416; found 372.0417.

9-((5-Methyl-4-(perfluorocyclopent-1-en-1-yl)thiophen-2-yl)ethynyl)-2,3,6,7-tetrahydro-1*H*,5*H*-pyrido[3,2,1-*ij*]quinoline (8).

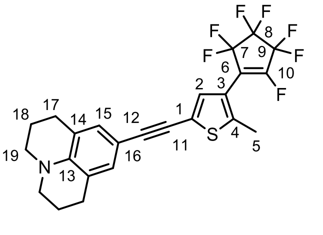

To a solution of **6** (830 mg, 2.23 mmol) in anhydrous THF (70 mL) was added *n*-BuLi (1.6 M in hexane, 1.53 mL, 2.45 mmol, 1.1 equiv.) dropwise at −78 °C. The mixture was stirred for 2 h before adding octafluorocyclopentene (1.00 mL, 7.45 mmol, 3.3 equiv.). The reaction mixture was stirred at −78 °C for 2 h and then at room temperature for 2 h followed by quenching with water and extracting with CH_2Cl_2. The organic extracts were combined and washed with saturated aqueous solution of sodium bicarbonate and water, dried ($MgSO_4$), and concentrated *in vacuo*. The crude product was purified by flash column chromatography (PE/EtOAc 20:1) followed by precipitation in methanol to yield the product as an off-white solid (450 mg, 42%). δ_H (400 MHz, $CDCl_3$): 7.12 (1H, s, C(2)H), 6.94 (2H, s, C(15)H), 3.19 (4H, t, $^3J_{HH}$ 5.9, C(19)H), 2.71 (4H, t, $^3J_{HH}$ 6.9, C(17)H), 2.43 (3H, d, $^6J_{HF}$ 3.3, C(5)H, 1.95 (4H, tt, $^3J_{HH}$ 5.9, $^3J_{HH}$ 6.9, C(18)H); δ_C (101 MHz, $CDCl_3$): 143.8, 143.5, 130.3, 129.8, 123.6, 121.1, 107.7, 96.3, 78.7, 51.0, 50.0, 27.7, 21.8, 14.8; C(6), C(7), C(8), C(9), and C(10) are not observed because the carbon signals are very weak resulting from the C-F coupling; δ_F (376 MHz, $CDCl_3$): −108.5 (2F, m, C(8)F), −118.0 (2F, m, C(9)F), −127.2 (1F, m, C(10)F), −130.0 (2F, m, C(7)F).

4-(4-Bromo-5-methylthiophen-2-yl)pyridine (9)

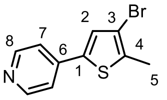

9 (3.00 g, 11.7 mmol, 1 equiv.), 4-(4,4,5,5-tetramethyl-1,3,2-dioxaborolan-2-yl)pyridine (2.52 g, 12.3 mmol, 1.05 equiv.), $Pd(PPh_3)_4$ (135 mg, 117 µmol, 10 mol%), and Cs_2CO_3 (2.00 g, 6.14 mmol) were dried under high vacuum for 2 h. Anhydrous 1,4-dioxane and water were degassed by three cycles of freeze-pump-thaw. To the dried mixture of solids under an argon atmosphere was added 1,4-dioxane (40 mL) and water (5 mL). The reaction mixture was stirred at 80 °C for 48 h before being allowed to cool to room temperature. The reaction was concentrated in vacuo, dispersed in water, and then extracted with CH_2Cl_2. The organic extracts were combined, dried ($MgSO_4$), and concentrated *in vacuo*. The crude product was purified by flash column chromatography (EtOAc) followed by precipitation with pentane at −78 °C to yield the product as an off-white solid (2.38 g, 80%). δ_H (400 MHz, $CDCl_3$): 8.58 (2H, d, $^3J_{HH}$ 5.9, C(8)H), 7.38 (2H, d, $^3J_{HH}$ 5.9, C(7)H), 7.31 (1H, s, C(2)H), 2.44 (3H, s, C(5)H); δ_C (101 MHz, $CDCl_3$): 150.6, 140.5, 138.0, 136.6, 128.1, 119.3, 110.8, 15.2. HRMS (ESI$^+$) *m/z*: $[M+H]^+$ calcd. for $C_{10}H_9NBrS$ 253.9634; found 253.9635.

9-((4-(3,3,4,4,5,5-Hexafluoro-2-(2-methyl-5-(pyridin-4-yl)thiophen-3-yl)cyclopent-1-en-1-yl)-5-methylthiophen-2-yl)ethynyl)-2,3,6,7-tetrahydro-1*H*,5*H*-pyrido[3,2,1-*ij*]quinoline (Dy-A(p))

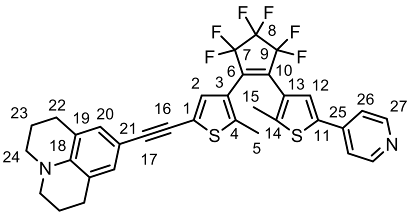

To a solution of **10** (240 mg, 944 µmol, 1 equiv.) in anhydrous THF (50 mL) was added *n*-BuLi (1.6 M in hexane, 0.70 mL, 1.1 mmol, 1.2 equiv.) dropwise at −78 °C. The mixture was stirred for 1.5 h before adding a solution of **8** (413 mg, 850 µmol, 0.9 equiv.) in anhydrous THF (5 mL). The reaction mixture was stirred for 2.5 h at −78 °C and then 2 h at room temperature followed by quenching with water (100 mL) and extraction with CH_2Cl_2. The organic extracts were combined and washed with saturated aqueous sodium bicarbonate solution and water, dried ($MgSO_4$), and concentrated *in vacuo*. The crude product was purified by flash column chromatography (PE/EtOAc 5:1) followed by size exclusion chromatography (with $CHCl_3$ eluent) to yield the product as a brown solid (201 mg, 33%). δ_H (400 MHz, $CDCl_3$): 8.60 (2H, d, $^3J_{HH}$ 4.7, C(27)H), 7.45 (1H, s, C(12)H), 7.41 (2H, d, $^3J_{HH}$ 4.7, C(26)H), 7.13 (1H, s, C(2)H), 6.93 (2H, s, C(20)H), 3.19 (4H, t, $^3J_{HH}$ 5.9, C(24)H), 2.71 (4H, t, $^3J_{HH}$ 6.5, C(22)H), 2.00 (3H, s, C(15)H), 1.95 (4H, tt, $^3J_{HH}$ 5.9, $^3J_{HH}$ 6.5, C(23)H), 1.91 (3H, s, C(5)H); δ_C (101 MHz, $CDCl_3$): 150.7, 143.9, 143.5, 142.0, 140.4, 139.2, 130.3, 129.9, 126.4, 124.9, 124.7, 123.5, 121.1, 119.7, 107.6, 96.3, 78.9, 50.0, 27.7, 21.8, 14.9, 14.6, C(6), C(7), C(8), C(9) and C(10) are not observed because the carbon signals are very weak resulting from the C-F coupling; δ_F (376 MHz, $CDCl_3$): −110.0 (2F, t, $^3J_{FF}$ 6.1, C(9)F), −110.3 (2F, t, $^3J_{FF}$ 5.8,

C(7)F), −131.8 (2F, tt, $^3J_{FF}$ 5.8, $^3J_{FF}$ 6.1, C(8)F). HRMS (ESI$^+$) *m/z*: [M+H]$^+$ calcd. for $C_{34}H_{27}F_6N_2S_2^+$ 641.1514; found 641.1508.

4-(4-(3,3,4,4,5,5-Hexafluoro-2-(2-methyl-5-((2,3,6,7-tetrahydro-1*H*,5*H*-pyrido[3,2,1-*ij*]quinolin-9-yl)ethynyl)thiophen-3-yl)cyclopent-1-en-1-yl)-5-methylthiophen-2-yl)-1-methylpyridin-1-ium hexafluorophosphate (Dy-A)

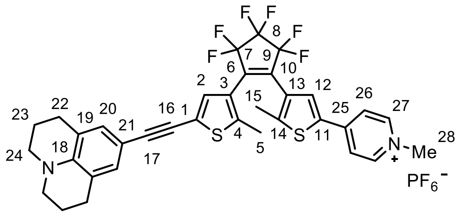

To a solution of **Dy-A(p)** (40.0 mg, 62.4 μmol) in CH_2Cl_2 (20 mL) was added iodomethane (0.50 mL, 8.0 mmol). The reaction mixture was stirred at room temperature for 20 h and then at 45 °C for 2 h before being concentrated *in vacuo*. The dried crude product was dissolved in methanol and then added to a solution of potassium hexafluorophosphate (4.0 g, 22 mmol) in water (50 mL). The solution was stirred at room temperature for 1 h for ion exchange and the precipitate was collected by centrifugation to yield the crude product as a brown solid. The crude product was purified by recrystallisation from CH_2Cl_2/cyclohexane to yield a brown solid. The brown solid was further purified by flash column chromatography (CH_2Cl_2/MeOH 95:5) to yield the product as a pale green solid (40 mg, 98%). δ_H (400 MHz, CD_3CN): 8.55 (2H, d, $^3J_{HH}$ 6.8, C(27)H), 8.05 (2H, d, $^3J_{HH}$ 6.8, C(26)H), 7.98 (1H, s, C(12)H), 7.15 (1H, s, C(2)H), 6.85 (2H, s, C(20)H), 4.22 (3H, s, C(28)H), 3.20 (4H, t, $^3J_{HH}$ 5.8, C(24)H), 2.67 (4H, t, $^3J_{HH}$ 6.5, C(22)H), 2.10 (3H, s, C(15)H), 1.97 (3H, s, C(5)H), 1.89 (4H, tt, $^3J_{HH}$ 6.8, $^3J_{HH}$ 6.5, C(23)H); δ_C (101 MHz, CD_3CN): 151.2, 148.7, 146.2, 144.5, 144.0, 136.1, 132.1, 130.8, 130.5, 128.1, 125.0, 124.2, 123.3, 122.2, 107.5, 97.2, 79.1, 50.3, 48.3, 28.1, 22.2, 15.3, 14.7; C(6), C(7), C(8), C(9) and C(10) are not observed because the carbon signals are very weak resulting from the C-F coupling; δ_F (376 MHz, CD_3CN): −72.9 (6F, d, $^1J_{FP}$ 706.8, hexafluorophosphate), −107.3 (2F, t, $^3J_{FF}$ 5.5, C(7)F), −107.6 (2F, t, $^3J_{FF}$ 6.4, C(9)F), −129.1 (2F, tt, , $^3J_{FF}$ 5.5, $^3J_{FF}$ 6.4, C(8)F). HRMS (ESI$^+$) *m/z*: [M$^+$] calcd. for $C_{35}H_{29}F_6N_2S_2^+$ 655.1671; found 655.1669. UV-vis (MeCN): λ_{max} 354 nm (ε = 74200 M^{-1} cm^{-1}).

Trimethyl((5-methyl-4-(perfluorocyclopent-1-en-1-yl)thiophen-2-yl)ethynyl)silane (13)

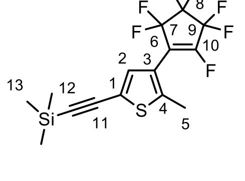

The compound **13** was synthesized following a published procedure,[27] yielding a yellow oil (657 mg, 52%). δ_H (400 MHz, $CDCl_3$): 7.20 (1H, s, C(2)H), 2.43 (3H, d, $^6J_{HF}$ 2.9, C(5)H), 0.25 (9H, s, C(13)H); δ_C (101 MHz, $CDCl_3$): 145.1, 132.1, 122.2, 119.6, 100.4, 96.1, 14.7, −0.2, C(6), C(7), C(8), and C(9) are not observed because the carbon signals

are very weak resulting from the C-F coupling; δ_F (376 MHz, $CDCl_3$): −108.7 (2F, m, C(7)F), −118.3 (2F, m, C(9)F), −126.9 (1F, m, C(10)F), −130.1 (2F, m, C(8)F).

9-(4-Bromo-5-methylthiophen-2-yl)-2,3,6,7-tetrahydro-1*H*,5*H*-pyrido[3,2,1-*ij*]quinoline (14)

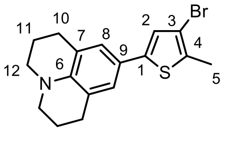

9 (3.00 g, 11.7 mmol), **3** (3.51 g, 11.7 mmol), $Pd(PPh_3)_4$ (667 mg, 586 μmol, 5 mol%), and Cs_2CO_3 (3.00 g, 9.21 mmol) were dried under high vacuum for 2 h. 1,4-Dioxane and water were degassed by three cycles of freeze-pump-thaw. To the dried mixture of solids under an argon atmosphere was added 1,4-dioxane (40 mL) and water (5 mL). The reaction mixture was stirred at 90 °C for 18 h before being allowed to cool to room temperature. The reaction mixture was concentrated *in vacuo*, dissolved in CH_2Cl_2, and washed with water and brine. The organic layer was dried ($MgSO_4$), and concentrated *in vacuo*. The crude product was purified by flash column chromatography (PE/EtOAc 20:1) to yield the product as a yellow solid (3.25 g, 80%). δ_H (400 MHz, $CDCl_3$): 6.95 (2H, s, C(8)H), 6.88 (1H, s, C(2)H), 3.17 (4H, t, $^3J_{HH}$ 5.7, C(12)H), 2.76 (4H, t, $^3J_{HH}$ 6.8, C(10)H), 1.98 (4H, tt, $^3J_{HH}$ 5.7, $^3J_{HH}$ 6.8, C(11)H); δ_C (101 MHz, $CDCl_3$): 142.9, 142.6, 130.9, 124.3, 122.8, 121.7, 121.1, 109.4, 50.1, 27.8, 22.1, 14.9. HRMS (ESI^+) *m/z*: $[M+H]^+$ calcd. for $C_{17}H_{19}BrNS$ 348.0416; found 348.0416.

9-(4-(2-(5-Ethynyl-2-methylthiophen-3-yl)-3,3,4,4,5,5-hexafluorocyclopent-1-en-1-yl)-5-methylthiophen-2-yl)-2,3,6,7-tetrahydro-1*H*,5*H*-pyrido[3,2,1-*ij*]quinoline (15)

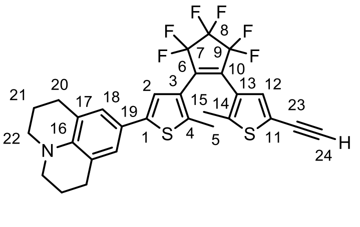

To a solution of **14** (650 mg, 1.87 mmol) in anhydrous THF (15 mL) at −78 °C was added *n*-BuLi (1.6 M in hexane, 1.28 mL, 2.05 mmol, 1.1 equiv.) dropwise. The mixture was stirred for 1 h before being transferred to a separate flask containing a solution of **13** (721 mg, 1.87 mmol) in anhydrous THF (40 mL) at −78 °C through a cannula. The reaction mixture was stirred at −78 °C for 2 h and then at room temperature for 2 h followed by quenching with water (10 mL). To the quenched mixture was added potassium carbonate (3.00 g, 21.7 mmol) and the reaction mixture was stirred for 30 min. The reaction mixture was then poured into water (100 mL) and extracted with CH_2Cl_2. The organic extracts were combined, washed with water, dried ($MgSO_4$), and concentrated *in vacuo*. The crude product was purified by flash column chromatography (PE/EtOAc 20:1) to yield the product as a pale green solid (630 mg, 60%). δ_H (400 MHz, $CDCl_3$): 7.27 (1H, s, C(12)H), 6.99 (1H, s, C(2)H), 6.95 (2H, s, C(18)H), 3.34 (1H, s, C(24)H), 3.17 (4H, t, $^3J_{HH}$ 5.8, C(22)H), 2.77 (4H, t, $^3J_{HH}$ 6.5, C(20)H), 1.98 (4H, tt, $^3J_{HH}$ 5.8, $^3J_{HH}$ 6.5, C(21)H), 1.89 (3H, s, C(15)H), 1.87 (3H, s, C(5)H); δ_C (101 MHz, $CDCl_3$): 143.9, 143.8, 143.0, 138.8, 133.0, 125.3, 125.2, 124.5, 121.8,

120.7, 120.3, 119.3, 82.1, 76.1, 50.1, 27.8, 22.1, 14.5(5), 14.5(2), C(6), C(7), C(8), C(9) and C(10) are not observed because the carbon signals are very weak resulting from the C-F coupling; δ_F (376 MHz, $CDCl_3$): −101.1 (2F, t, $^3J_{FF}$ 6.7, C(7)F), −110.2 (2F, t, $^3J_{FF}$ 7.9, C(9)F), −131.9 (2F, tt, $^3J_{FF}$ 6.7, $^3J_{FF}$ 7.9 C(8)F). HRMS (ESI$^+$) *m/z*: $[M+H]^+$ calcd. for $C_{29}H_{24}F_6NS_2^+$ 564.1249; found 564.1247.

9-(4-(3,3,4,4,5,5-Hexafluoro-2-(2-methyl-5-(pyridin-4-ylethynyl)thiophen-3-yl)cyclopent-1-en-1-yl)-5-methylthiophen-2-yl)-2,3,6,7-tetrahydro-1*H*,5*H*-pyrido[3,2,1-*ij*]quinoline (D-yA(p))

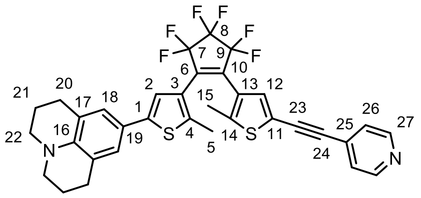

15 (550 mg, 976 µmol), 4-iodopyridine (300 mg, 1.45 mmol, 1.5 equiv.), $Pd(PPh_3)_4$ (113 mg, 97.6 µmol, 10 mol%), and CuI (31.2 mg, 195 µmol, 20 mol%) were dried under high vacuum for 30 min. Anhydrous triethylamine was degassed by three cycles of freeze-pump-thaw. To the dried mixture of solids under an argon atmosphere was added degassed triethylamine (60 mL). The reaction mixture was stirred at 45 °C for 15 h before being allowed to cool to room temperature. Upon completion of the reaction verified by TLC (PE/EtOAc 4:1 with 1% triethylamine), the reaction mixture was concentration *in vacuo*. The crude product was then purified by flash column chromatography (PE/EtOAc 4:1 with 1% triethylamine) to yield the product as a green solid (472 mg, 75%). δ_H (400 MHz, $CDCl_3$): 8.61 (2H, d, $^3J_{HH}$ 5.9, C(27)H), 7.33-7.37 (3H, overlapping s and d, $^3J_{HH}$ 5.9, C(12)H and C(26)H), 7.01 (1H, s, C(2)H), 6.96 (2H, s, C(18)H), 3.17 (4H, t, $^3J_{HH}$ 5.8, C(22)H), 2.77 (4H, t, $^3J_{HH}$ 6.4, C(20)H), 1.98 (4H, tt, $^3J_{HH}$ 5.8, $^3J_{HH}$ 6.4, C(21)H), 1.94 (3H, s, C(15)H), 1.90 (3H, s, C(5)H); δ_C (101 MHz, $CDCl_3$): 150.0, 145.0, 144.0, 143.0, 138.8, 133.2, 130.9, 125.7, 125.3, 125.3, 124.5, 121.8, 120.6, 120.2, 119.3, 91.3, 86.4, 50.0, 27.8, 22.0, 14.6(4), 14.5(7), C(6), C(7), C(8), C(9) and C(10) are not observed because the carbon signals are very weak resulting from the C-F coupling; δ_F (376 MHz, ($CDCl_3$): −110.0 (2F, t, $^3J_{FF}$ 6.7, C(7)F), −110.3 (2F, t, $^3J_{FF}$ 8.1, C(9)F), −131.9 (2F, tt, $^3J_{FF}$ 6.7, $^3J_{FF}$ 8.1, C(8)F). HRMS (ESI$^+$) *m/z*: $[M+H]^+$ calcd. for $C_{29}H_{24}NF_6S_2$ 564.1249; found 564.1247.

4-((4-(3,3,4,4,5,5-Hexafluoro-2-(2-methyl-5-(2,3,6,7-tetrahydro-1*H*,5*H*-pyrido[3,2,1-*ij*]quinolin-9-yl)thiophen-3-yl)cyclopent-1-en-1-yl)-5-methylthiophen-2-yl)ethynyl)-1-methylpyridin-1-ium hexafluorophosphate (D-yA)

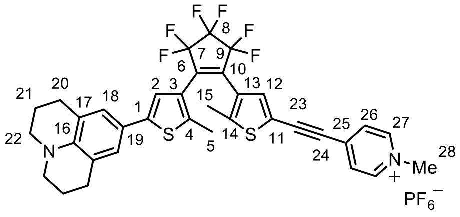

To a solution of **D-yA(p)** (200 mg, 312 μmol) in CH_2Cl_2 (20 mL) was added iodomethane (2.0 mL, 22 mmol). The reaction mixture was stirred at room temperature for 20 h and then at 45 °C for 2 h before being concentrated *in vacuo*. The dried crude product was dissolved in methanol and then added to a solution of potassium hexafluorophosphate (4.0 g, 21.73 mmol) in water (50 mL). The solution was stirred at room temperature for 1 h for ion exchange and the precipitate was collected by centrifugation to yield the crude product as a brown solid. The crude product was purified by recrystallisation from CH_2Cl_2/cyclohexane to yield the product as a brown solid. The brown solid was further purified by flash column chromatography (CH_2Cl_2/MeOH 95:5) to yield the product as a pale brown solid (205 mg, 82%). δ_H (400 MHz, CD_3CN): 8.98 (2H, d, $^3J_{HH}$ 6.5, C(27)H), 8.23 (2H, d, $^3J_{HH}$ 6.5, C(26)H), 7.75 (1H, s, C(12)H), 7.13 (1H, s, C(2)H), 6.95 (2H, s, C(18)H), 4.30 (3H, s, C(28)H), 3.14 (4H, t, $^3J_{HH}$ 5.8, C(24)H), 2.69 (4H, t, $^3J_{HH}$ 6.5, C(22)H), 2.02 (3H, s, C(15)H), 1.90 (3H, s, C(5)H), 1.86 (4H, tt, $^3J_{HH}$ 5.8, $^3J_{HH}$ 6.5, C(23)H); δ_C (101 MHz, CD_3CN): 148.1, 145.5, 143.7, 142.7, 138.4, 137.6, 135.7, 128.5, 125.2, 124.3, 123.8, 121.1, 119.2, 118.5, 118.0, 94.1, 90.1, 49.2, 47.8, 27.1, 21.3, 14.3, 14.0, C(6), C(7), C(8), C(9) and C(10) are not observed because the carbon signals are very weak resulting from the C-F coupling; δ_F (376 MHz, (CD_3CN): −67.9 (6F, d, $^1J_{FP}$ 707.0, hexafluorophosphate), −109.1 (2F, m, C(7)F), −109.4 (2F, m, C(9)F), −130.9 (2F, m, C(8)F). HRMS (ESI$^+$) *m/z*: [M$^+$] calcd. for $C_{35}H_{29}F_6N_2S_2^+$ 655.1671; found 655.1651. UV-vis (MeCN): λ_{max} 364 nm (ε = 55700 M^{-1} cm^{-1}).

4,4'-((Perfluorocyclopent-1-ene-1,2-diyl)bis(5-methylthiophene-4,2-diyl))bis(1-methylpyridin-1-ium) dichloride (A-A (chloride))

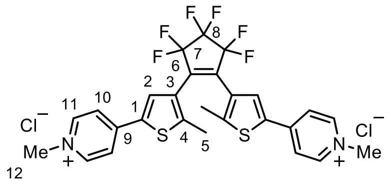

To a solution of tetrabutylammonium chloride (500 mg, 1.80 mmol) in acetone (30 mL) was added a solution of **A-A** (30 mg, 0.035 mmol) in THF (1 mL). The reaction mixture was stirred at room temperature for 1 h for ion exchange and the precipitate was filtered off to yield the crude product as an off-white solid. The crude product was purified by dissolving in MeOH and recrystallizing with a 1:1 mixture of THF and PE (22 mg, 98%). δ_H (400 MHz, CD_3OD): 8.79 (4H, d, $^3J_{HH}$ 7.1, C(11)H), 8.26 (4H, d, $^3J_{HH}$ 7.1 C(10)H), 8.18 (2H, s, C(2)H), 4.34 (6H, s, C(12)H), 2.17 (6H, s, C(5)H); δ_C (101 MHz, CD_3OD):

151.3, 149.2, 146.8, 137.1, 132.3, 128.3, 123.6, 48.0, 15.2, C(6), C(7) and C(8) are not observed because the carbon signals are very weak resulting from the C-F coupling; δ_F (376 MHz, CD_3OD): −118.8 (4F, t, $^3J_{FF}$ 5.3, C(7)F), −133.3 (2F, p, $^3J_{FF}$ 5.3, C(8)F). UV-vis (water): λ_{max} 357 nm (ε = 61500 M^{-1} cm^{-1}).

4,4'-(((Perfluorocyclopent-1-ene-1,2-diyl)bis(5-methylthiophene-4,2-diyl))bis(ethyne-2,1-diyl))bis(1-methylpyridin-1-ium) dichloride (Ay-yA (chloride))

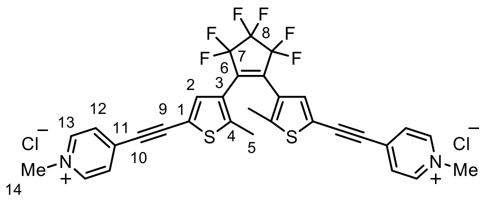

To a solution of tetrabutylammonium chloride (500 mg, 1.80 mmol) in acetone (20 mL) was added a solution of **Ay-yA** (50 mg, 0.056 mmol) in acetone (10 mL). The reaction mixture was stirred at room temperature for 1 h for ion exchange and the precipitate was filtered off to yield the product as an off-white solid (37 mg, 98%). δ_H (400 MHz, CD_3OD): 8.87 (4H, d, $^3J_{HH}$ 6.7, C(13)H), 8.11 (4H, d, $^3J_{HH}$ 6.7, C(12)H), 7.68 (2H, s, C(2)H), 4.38 (6H, s, C(14)H), 2.07 (6H, s, C(5)H); δ_C (101 MHz, CD_3OD): 149.6, 146.6, 147.0, 130.7, 129.9, 126.7, 120.4, 96.1, 90.9, 14.8, C(14) was not resolved due to overlapping with solvent peak, C(6), C(7) and C(8) are not observed because the carbon signals are very weak resulting from the C-F coupling. δ_F (376 MHz, (CD_3OD): −119.9 (4F, t, J_{FF} 5.0, C(7)F), −133.4 (2F, p, $^3J_{FF}$ 5.0, C(8)F). UV-vis (water): λ_{max} 377 nm (ε = 69500 M^{-1} cm^{-1}).

S18. Spectral Data

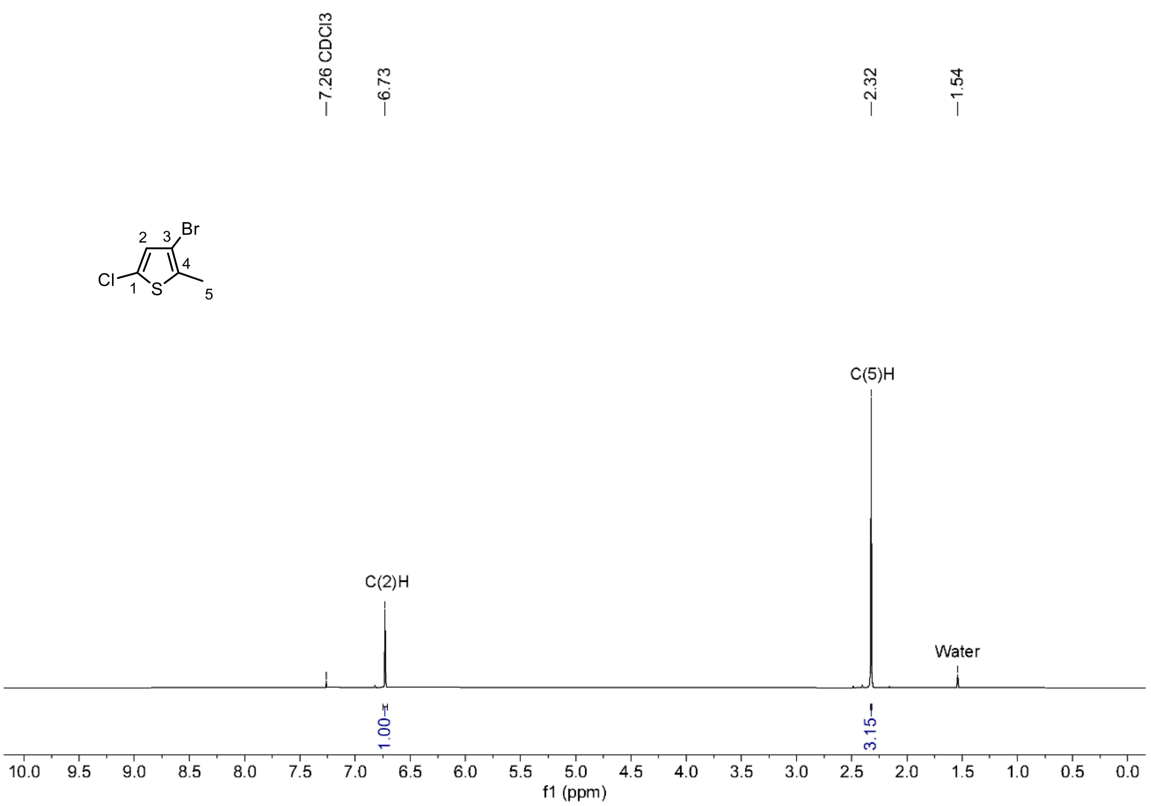

Figure S45 ^{1}H NMR spectrum (400 MHz, $CDCl_3$) of compound **1b**.

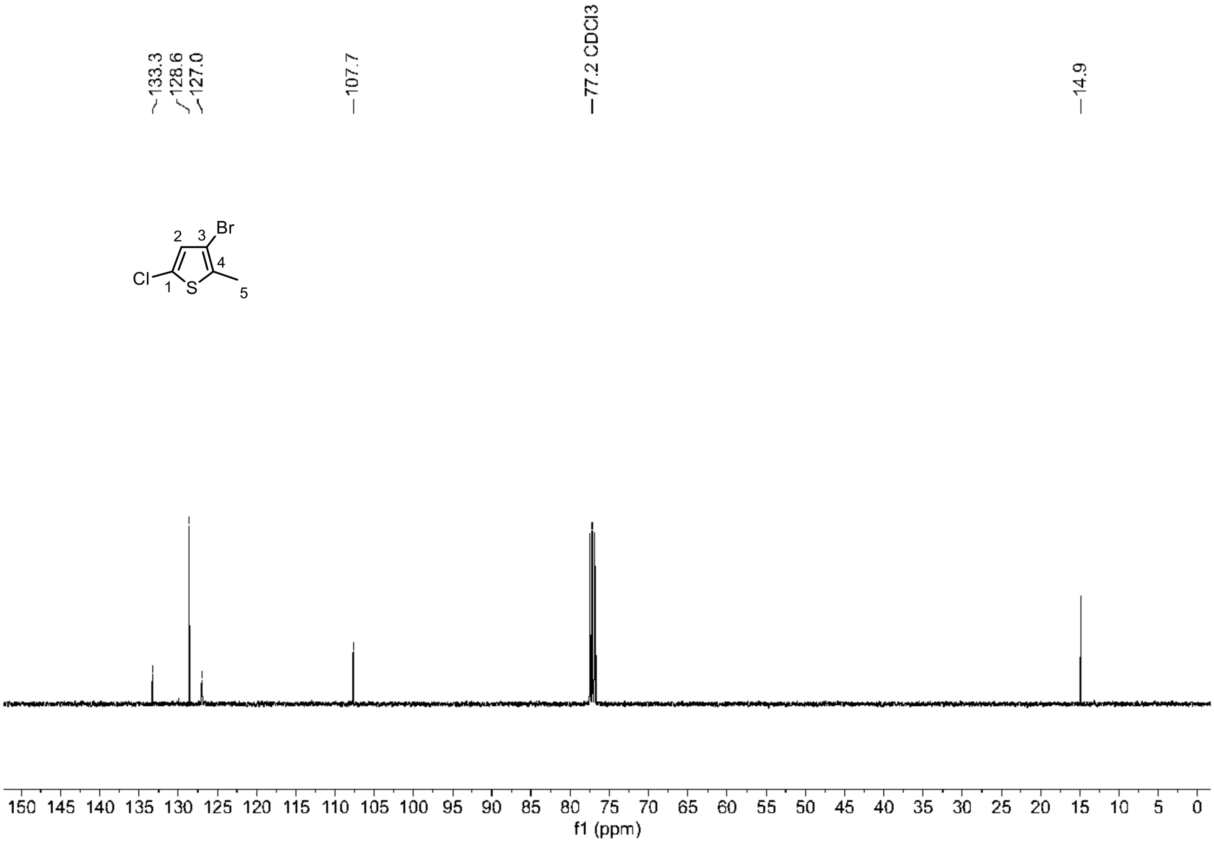

Figure S46 ^{13}C NMR spectrum (101 MHz, $CDCl_3$) of compound **1b**.

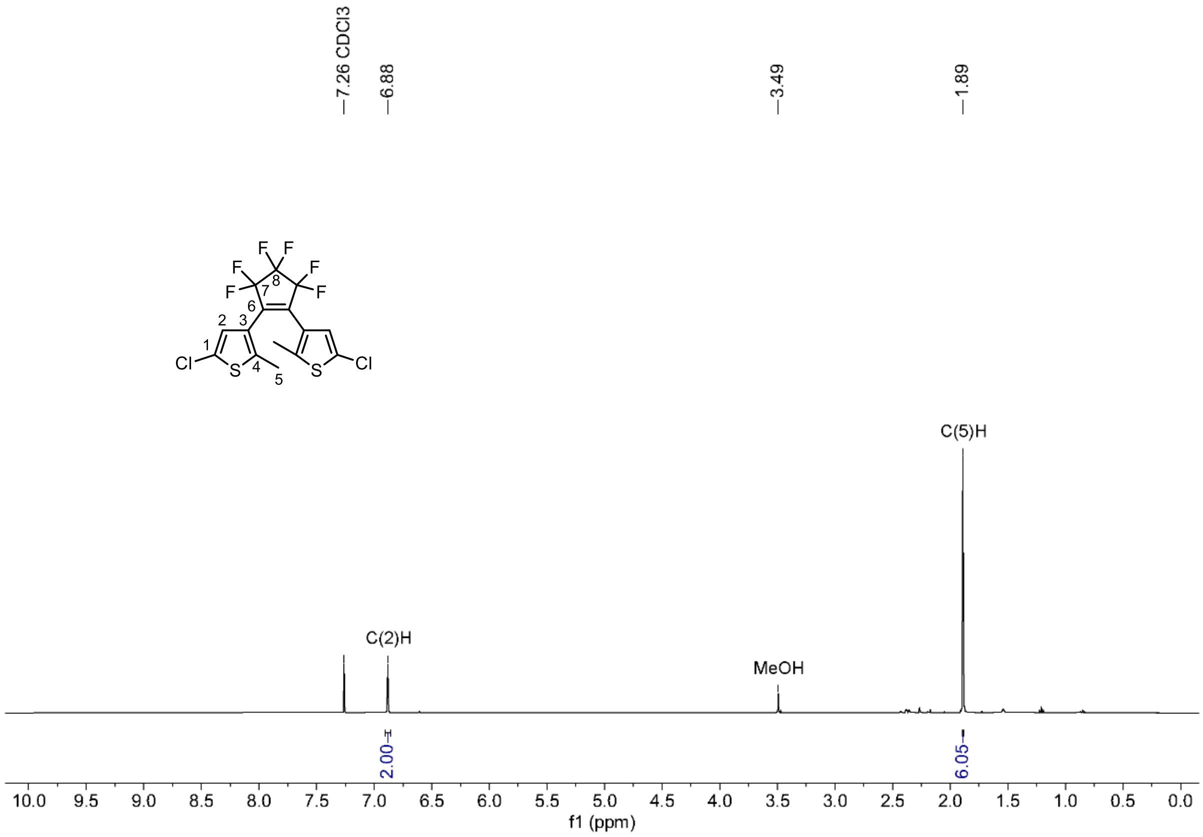

Figure S47 ^{1}H NMR spectrum (400 MHz, $CDCl_3$) of compound **1**.

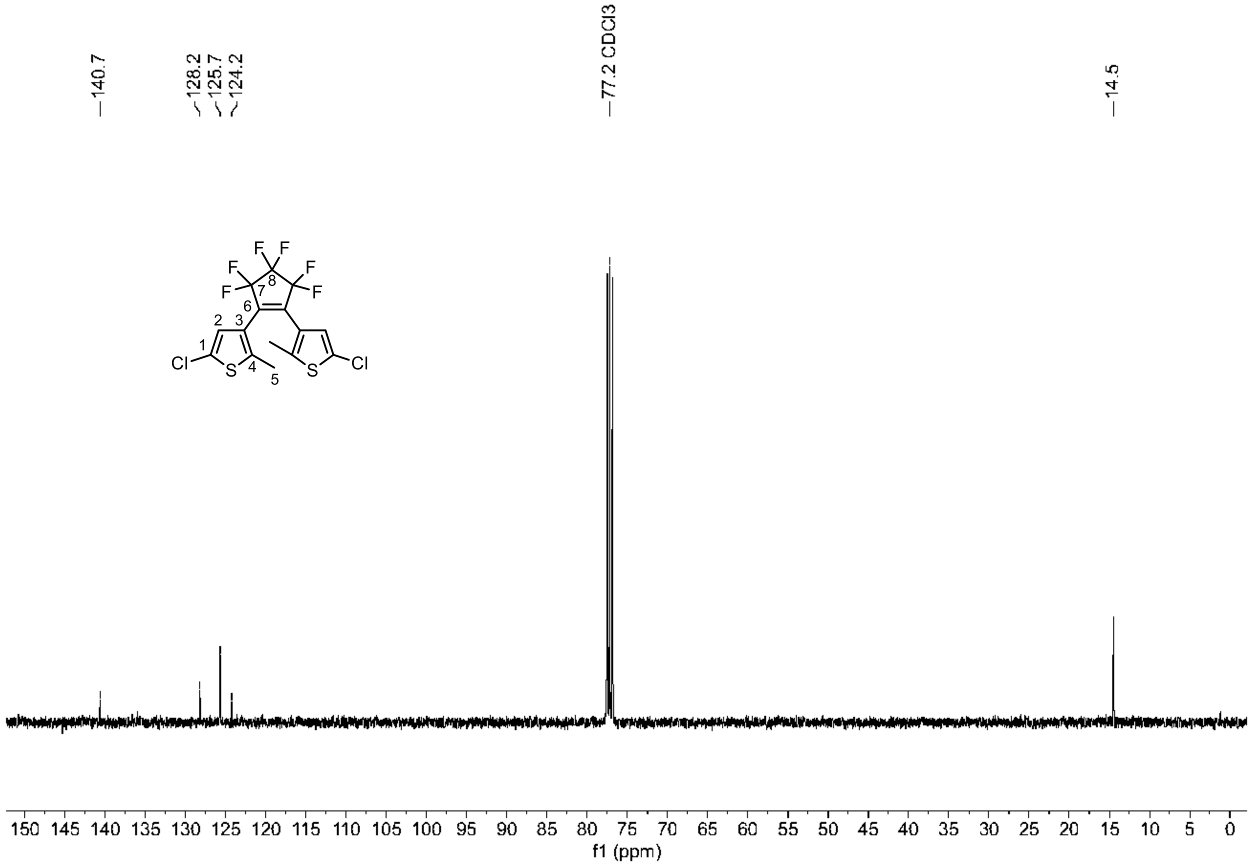

Figure S48 ^{13}C NMR spectrum (101 MHz, $CDCl_3$) of compound **1**.

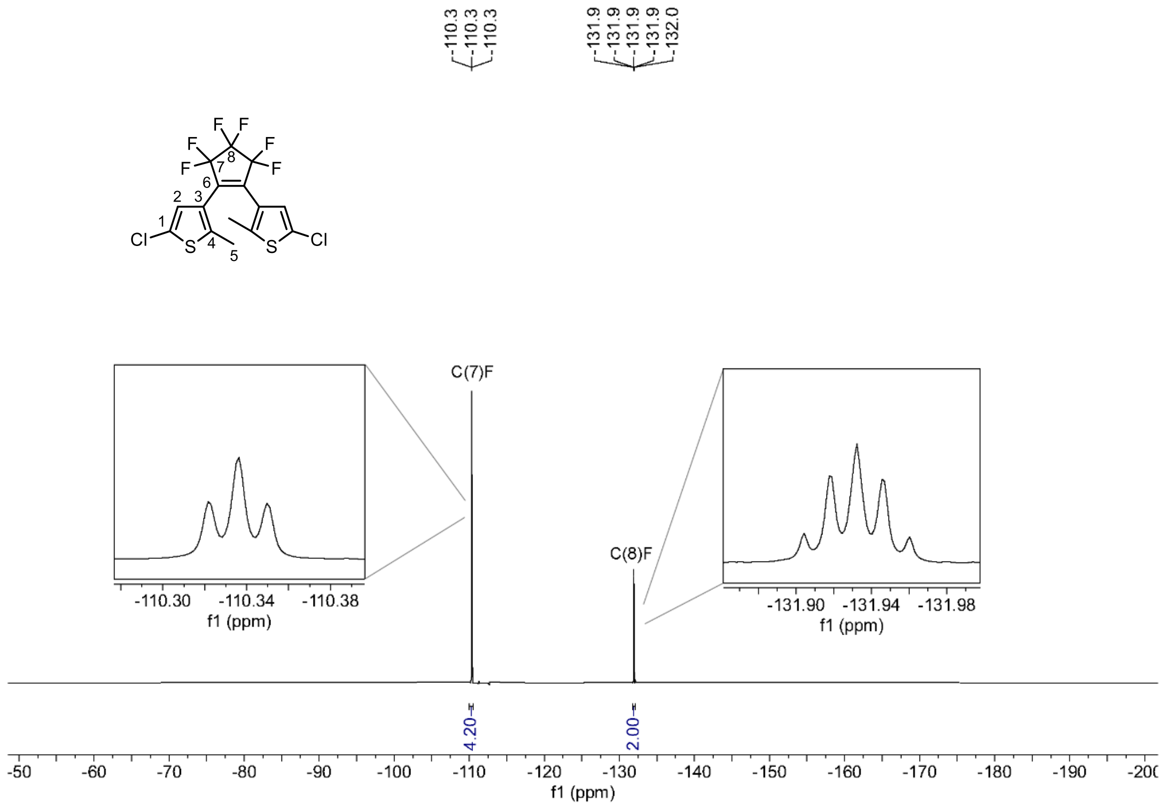

Figure S49 ^{19}F NMR spectrum (376 MHz, $CDCl_3$) of compound **1**.

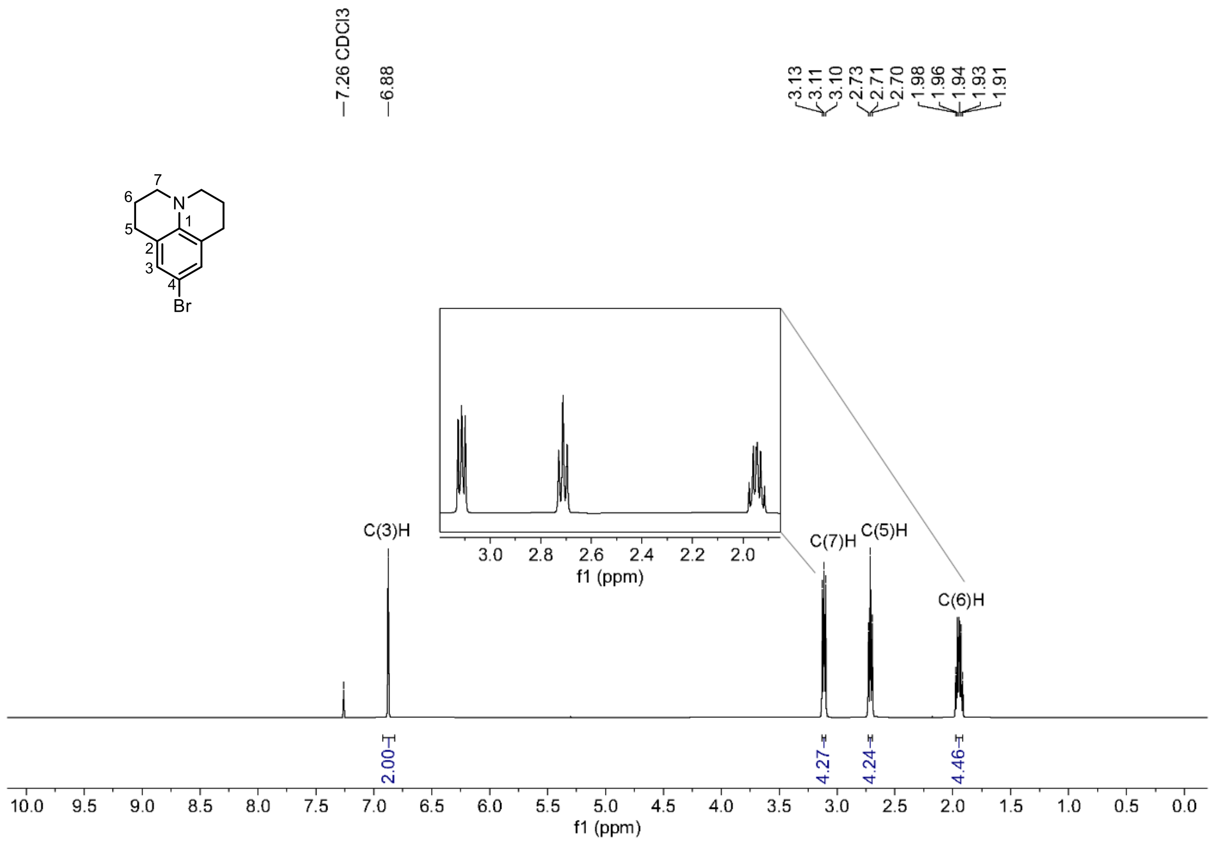

Figure S50 ^{1}H NMR spectrum (400 MHz, $CDCl_3$) of compound **3b**.

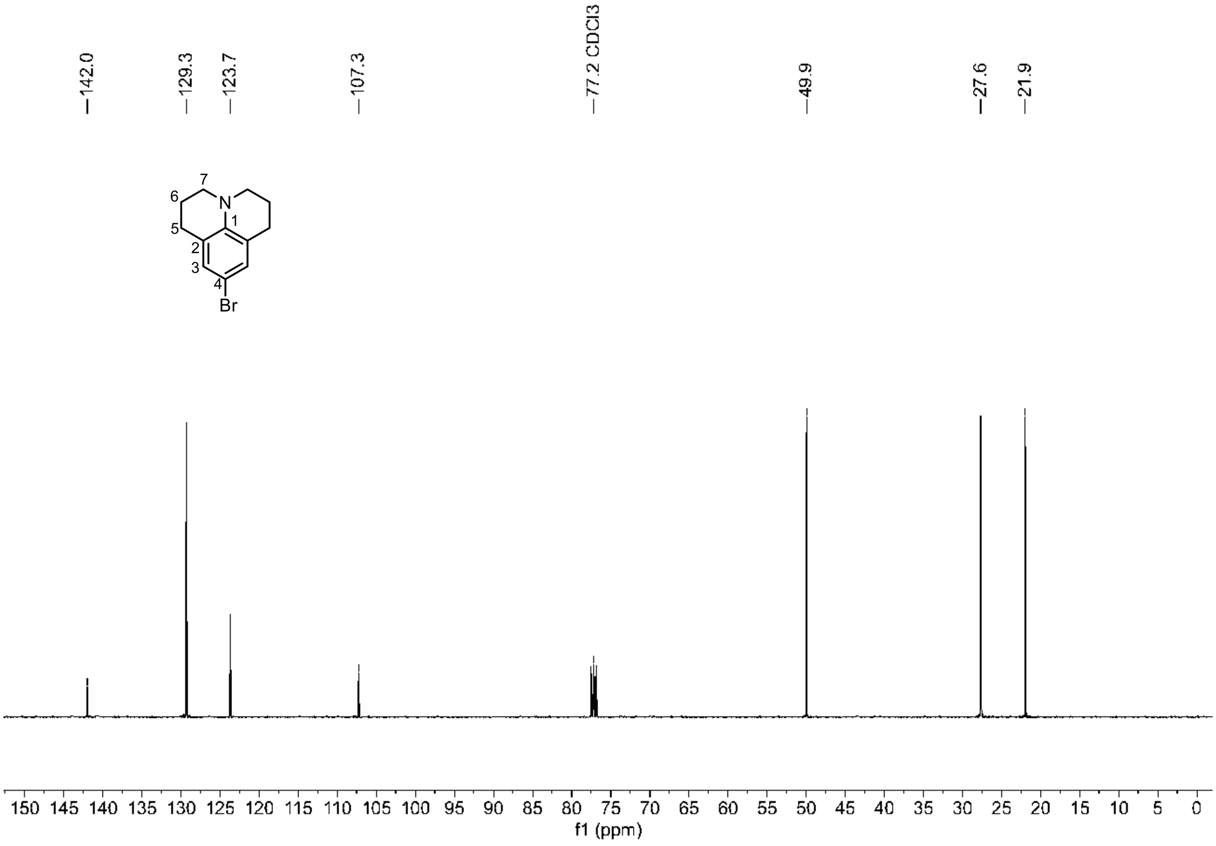

Figure S51 ^{13}C NMR spectrum (101 MHz, $CDCl_3$) of compound **3b**.

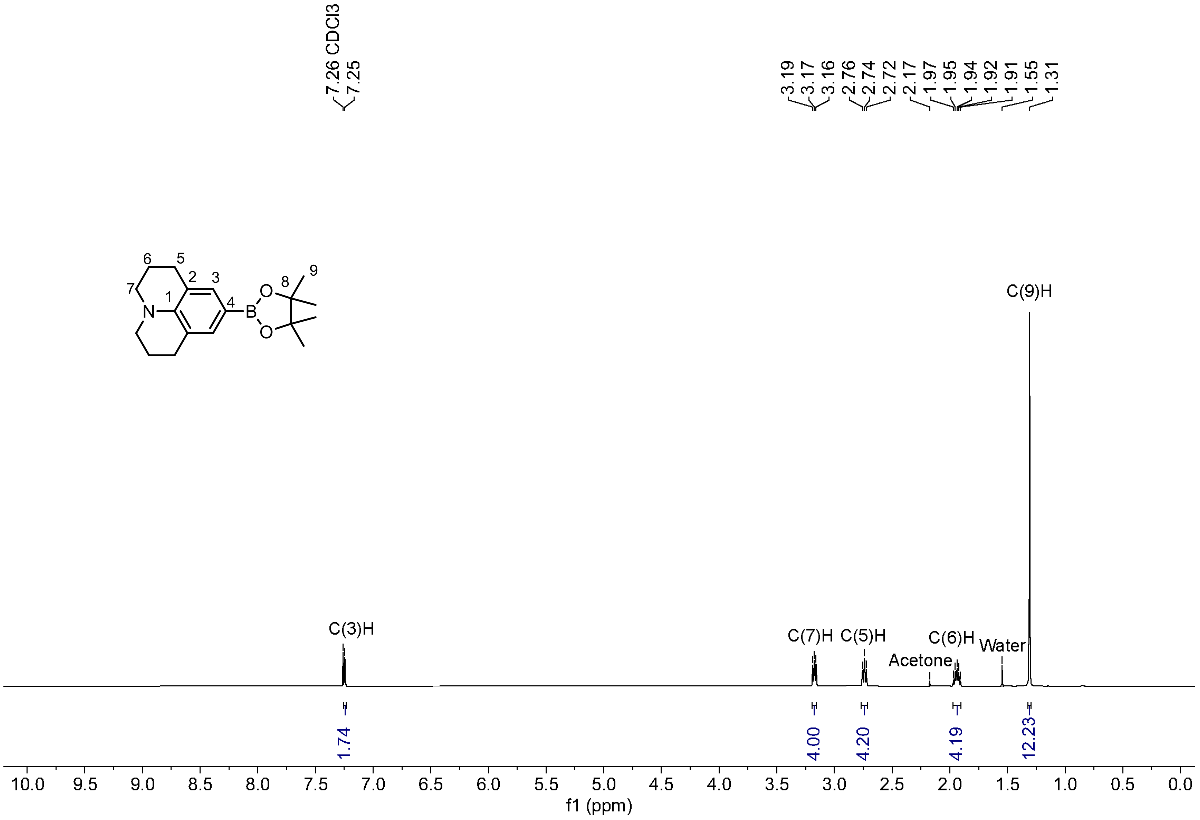

Figure S52 ^{1}H NMR spectrum (400 MHz, $CDCl_3$) of compound **3**.

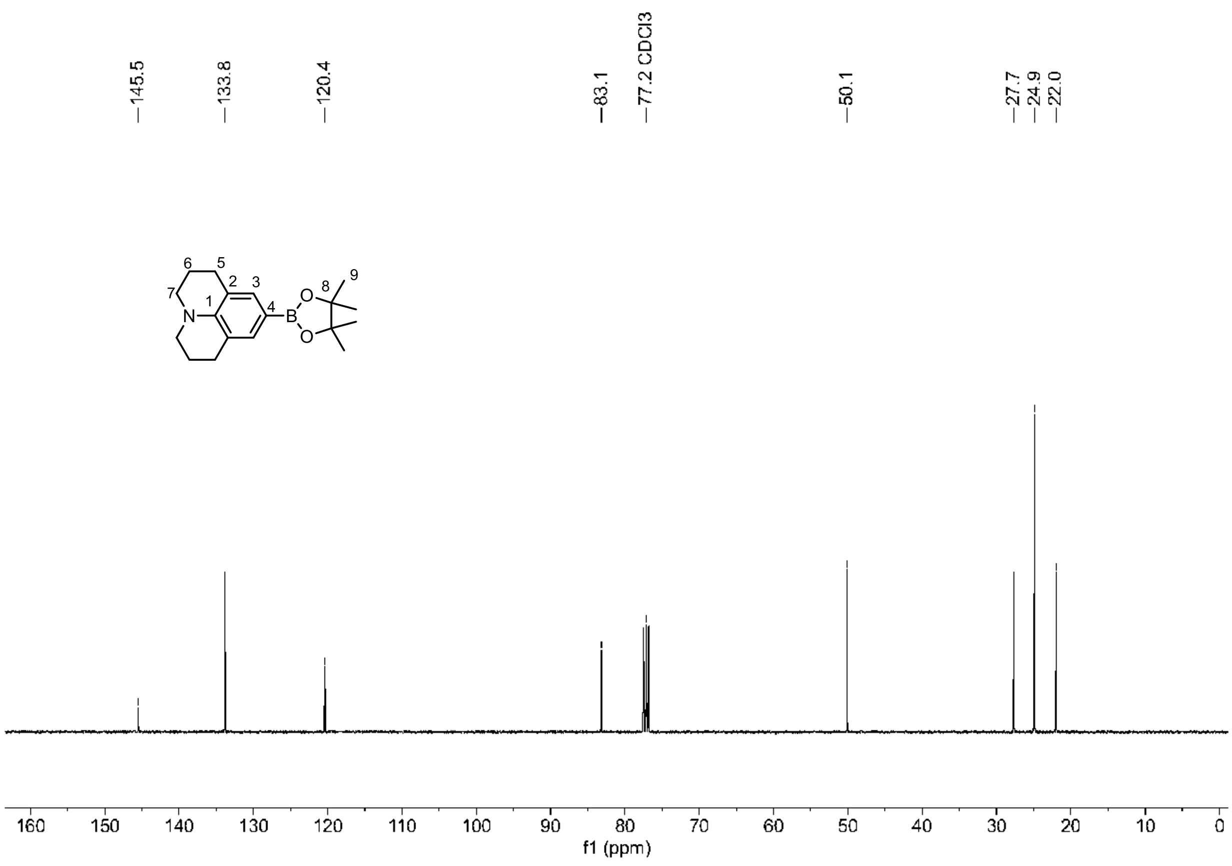

Figure S53 ^{13}C NMR spectrum (101 MHz, $CDCl_3$) of compound **3**.

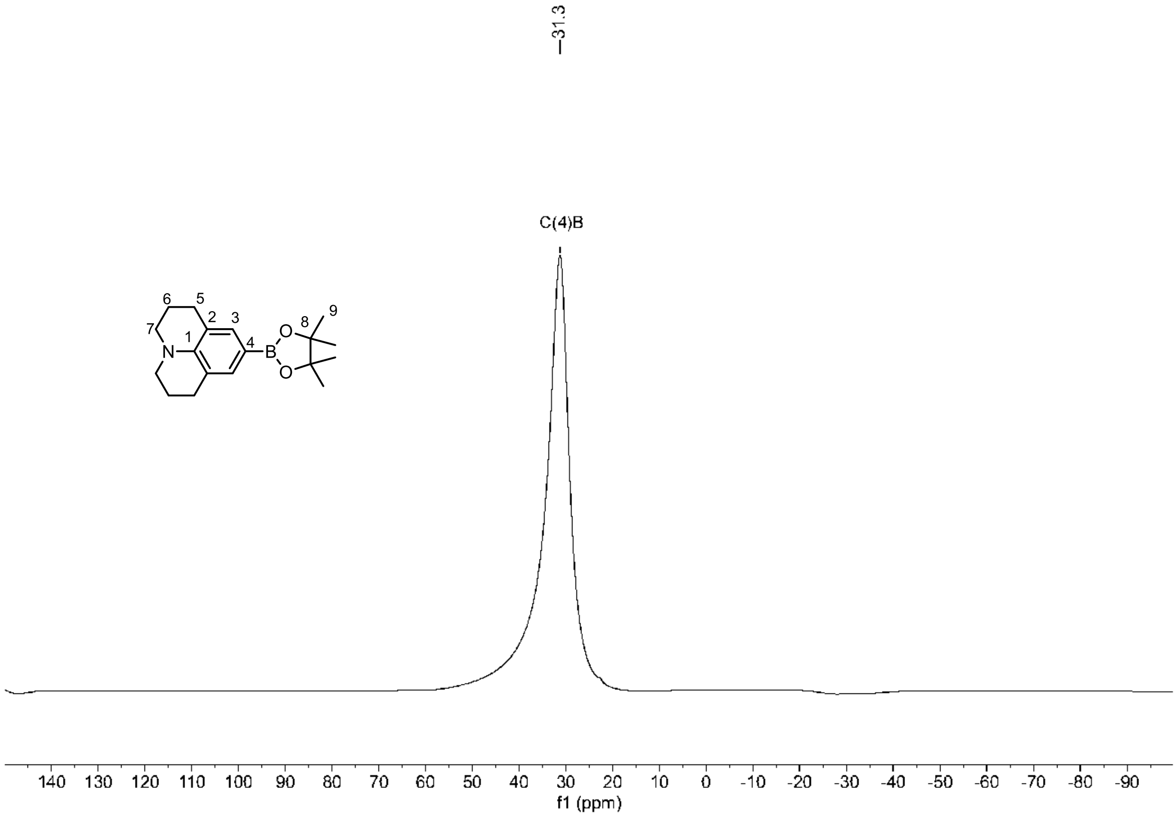

Figure S54 ^{11}B NMR spectrum (128 MHz, $CDCl_3$) of compound **3**.

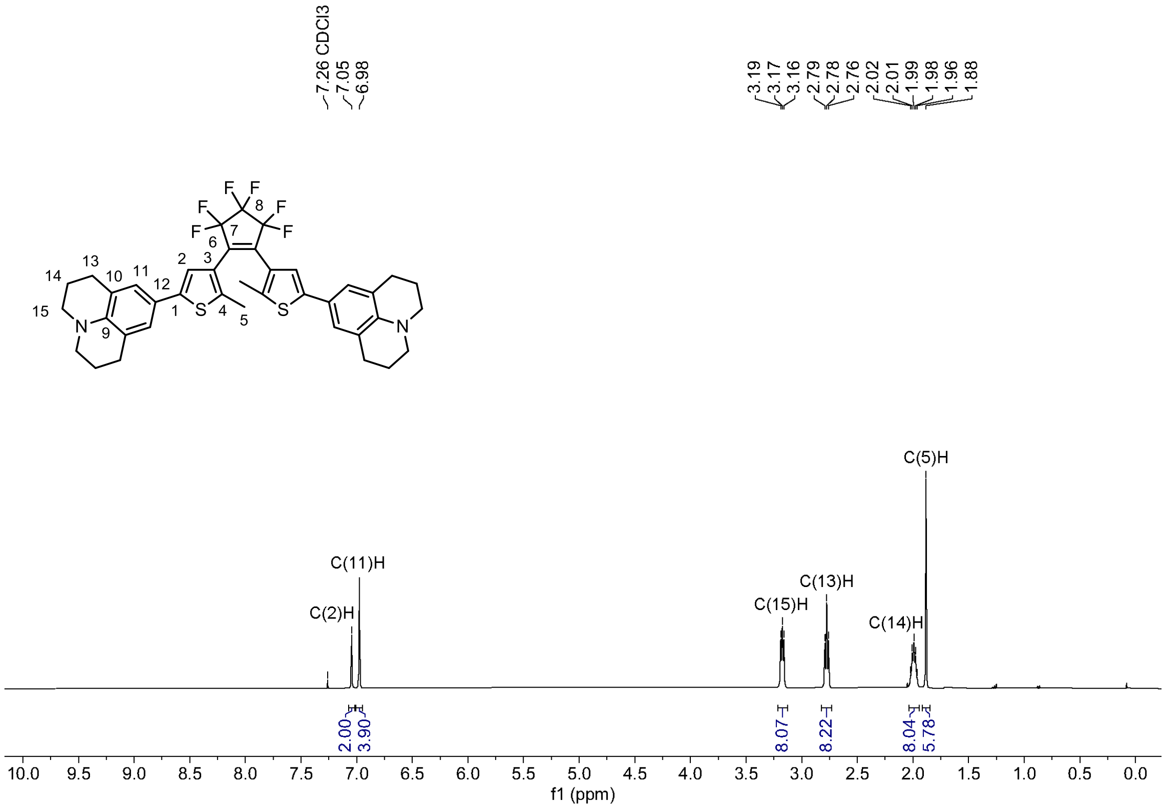

Figure S55 ^{1}H NMR spectrum (400 MHz, $CDCl_3$) of **D-D**.

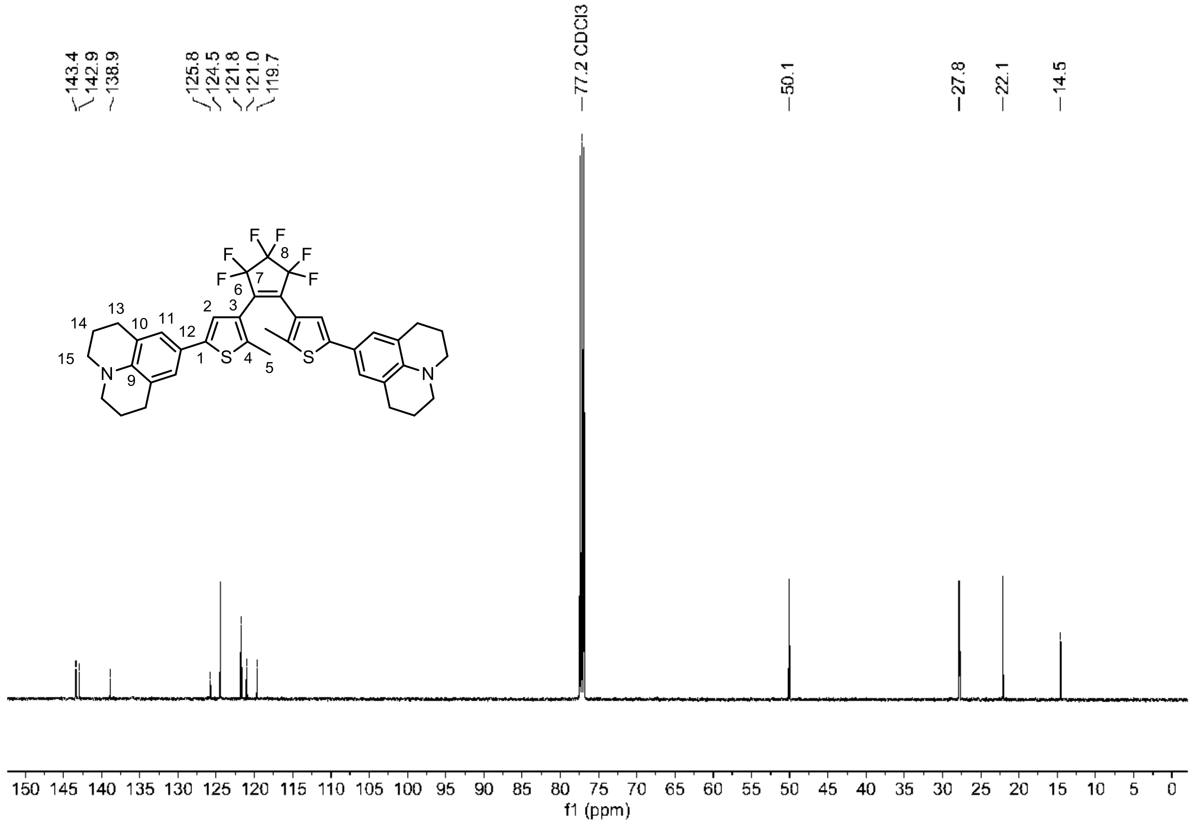

Figure S56 ^{13}C NMR spectrum (101 MHz, $CDCl_3$) of **D-D**.

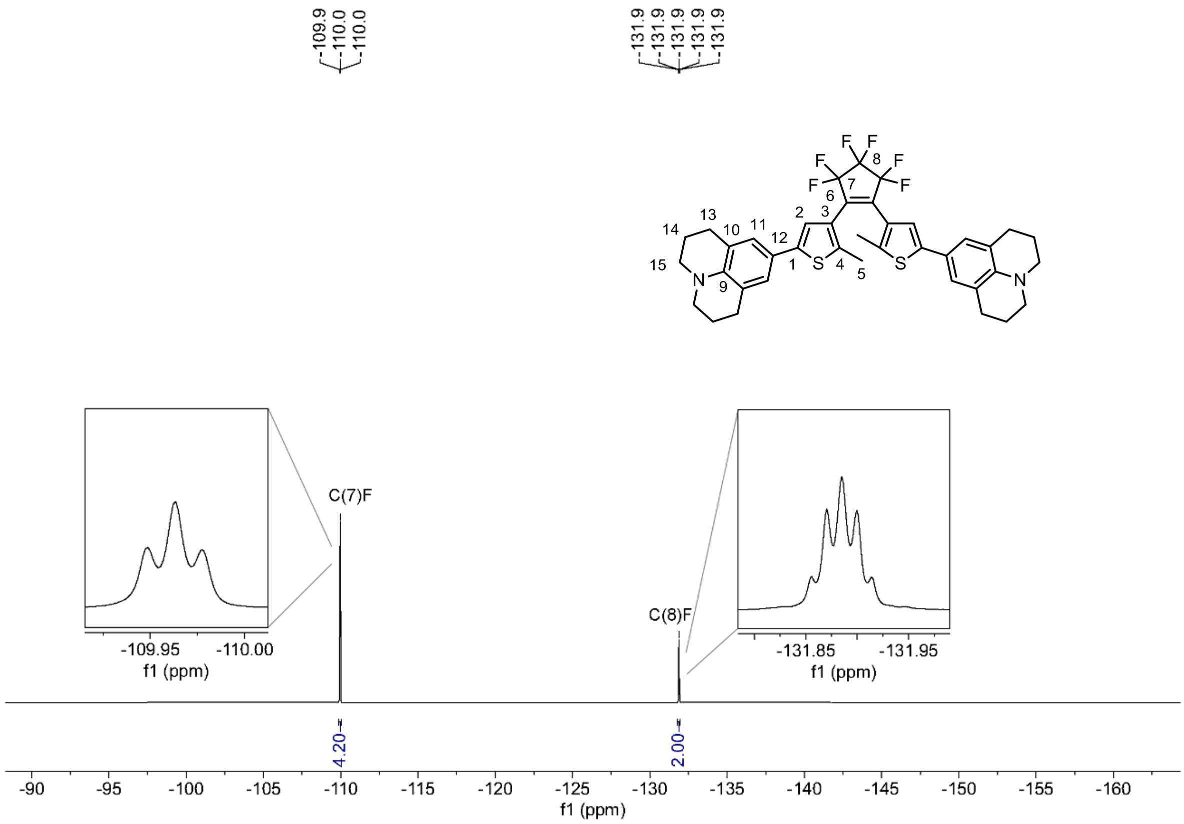

Figure S57 ^{19}F NMR spectrum (376 MHz, CDCl$_3$) of **D-D**.

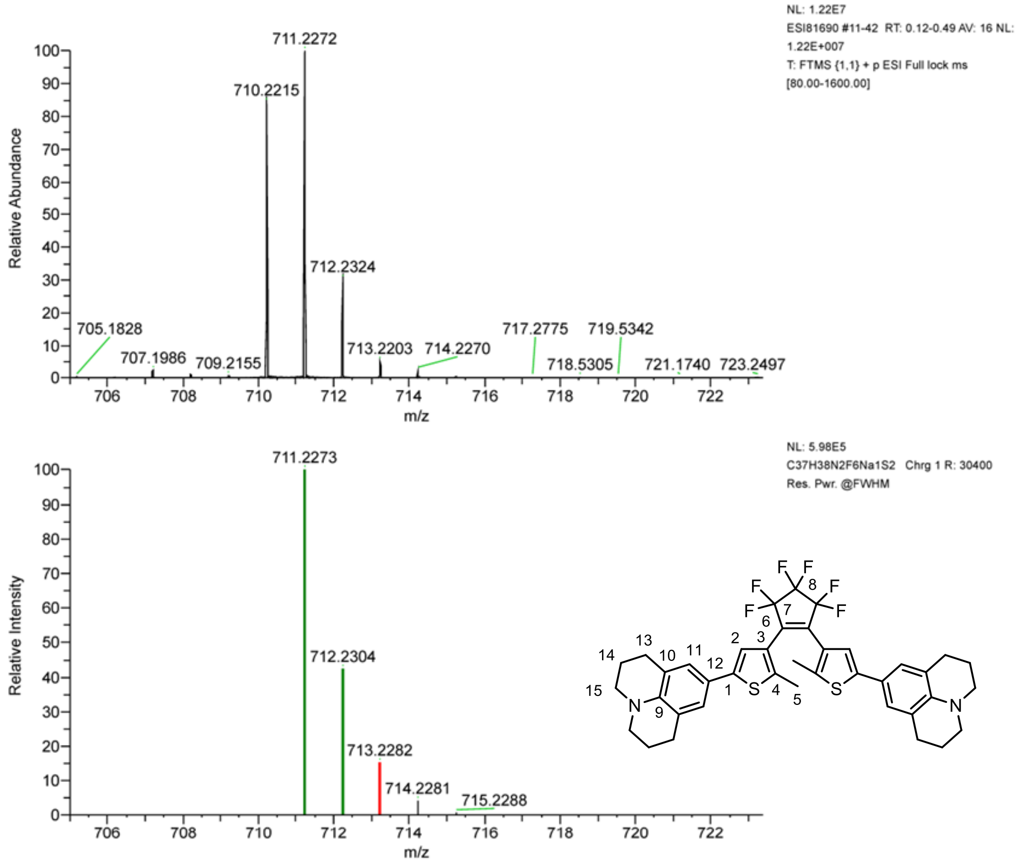

Figure S58 HRMS (ESI$^+$) of **D-D**, *m/z*: [M+H]$^+$ calcd. for $C_{39}H_{37}N_2F_6S_2^+$ 711.2273; found 711.2272.

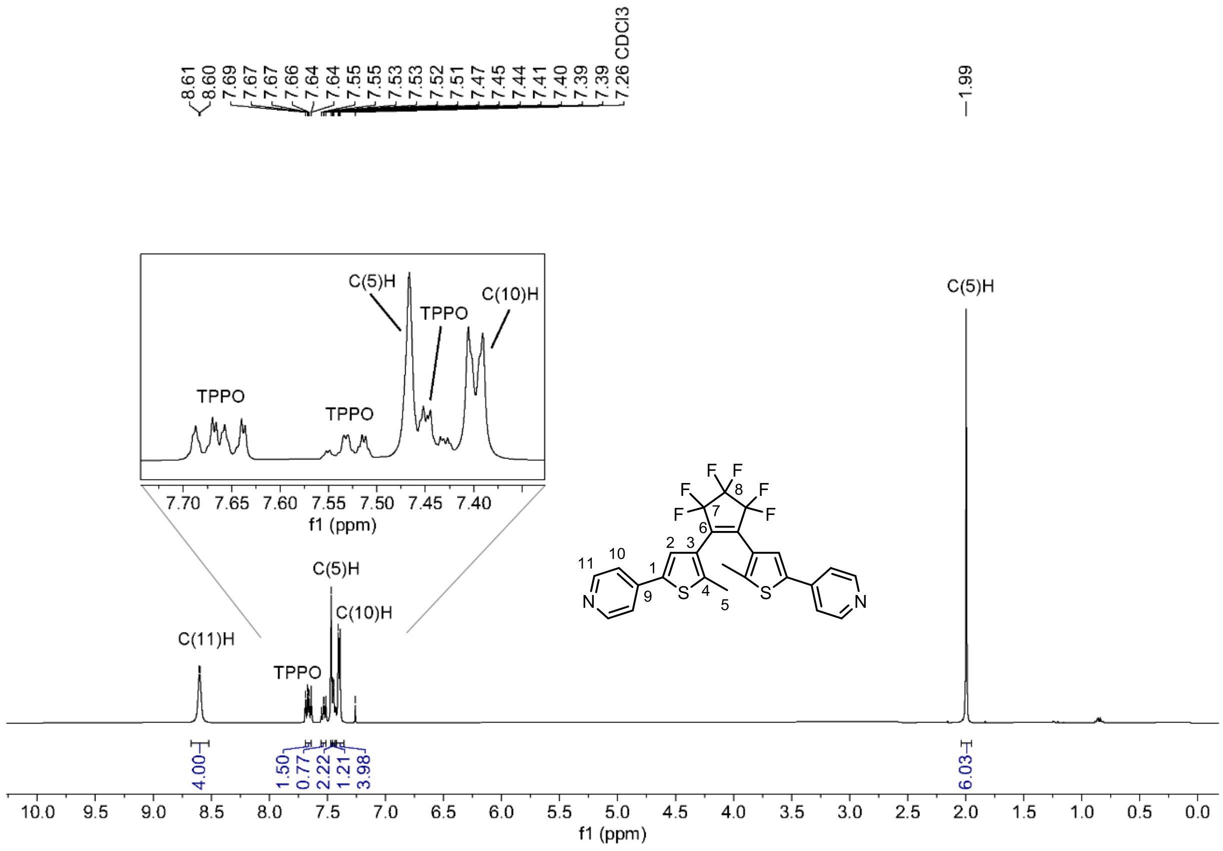

Figure S59 ^{1}H NMR spectrum (400 MHz, $CDCl_3$) of **A-A(p)**.

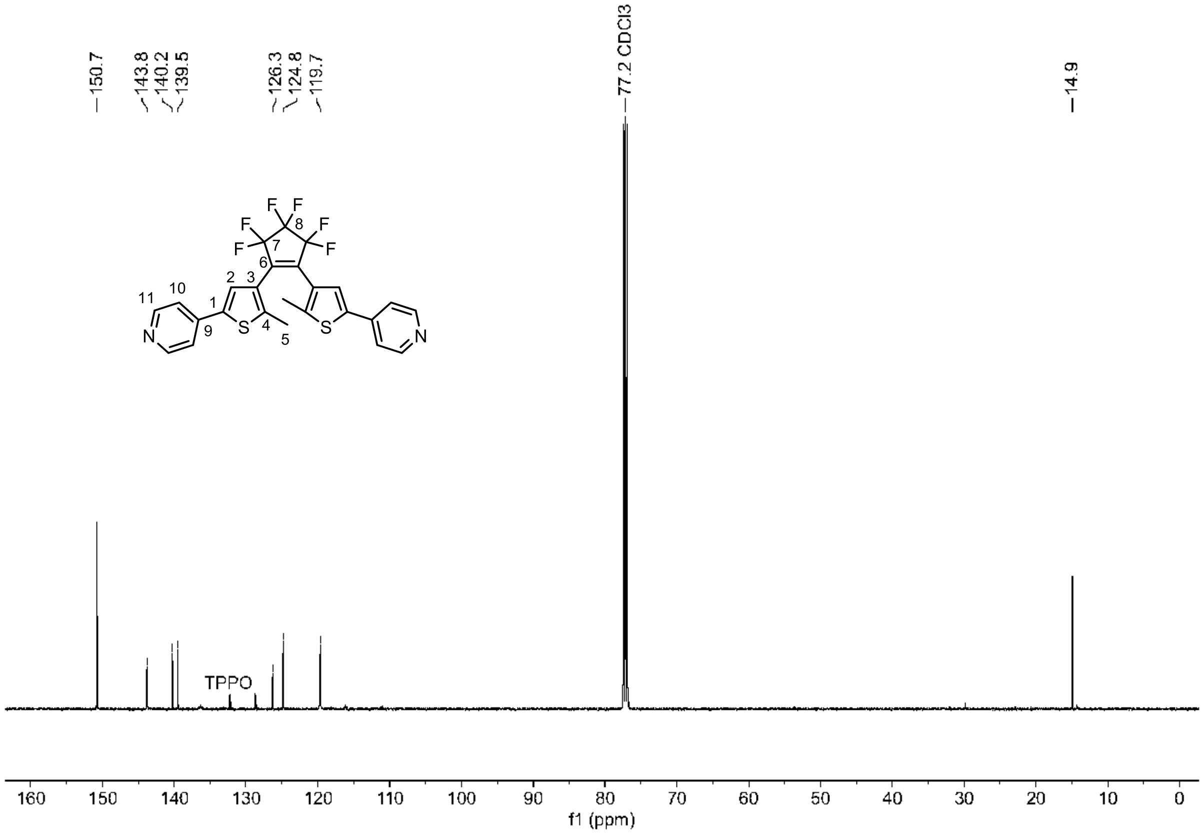

Figure S60 ^{13}C NMR spectrum (101 MHz, $CDCl_3$) of **A-A(p)**.

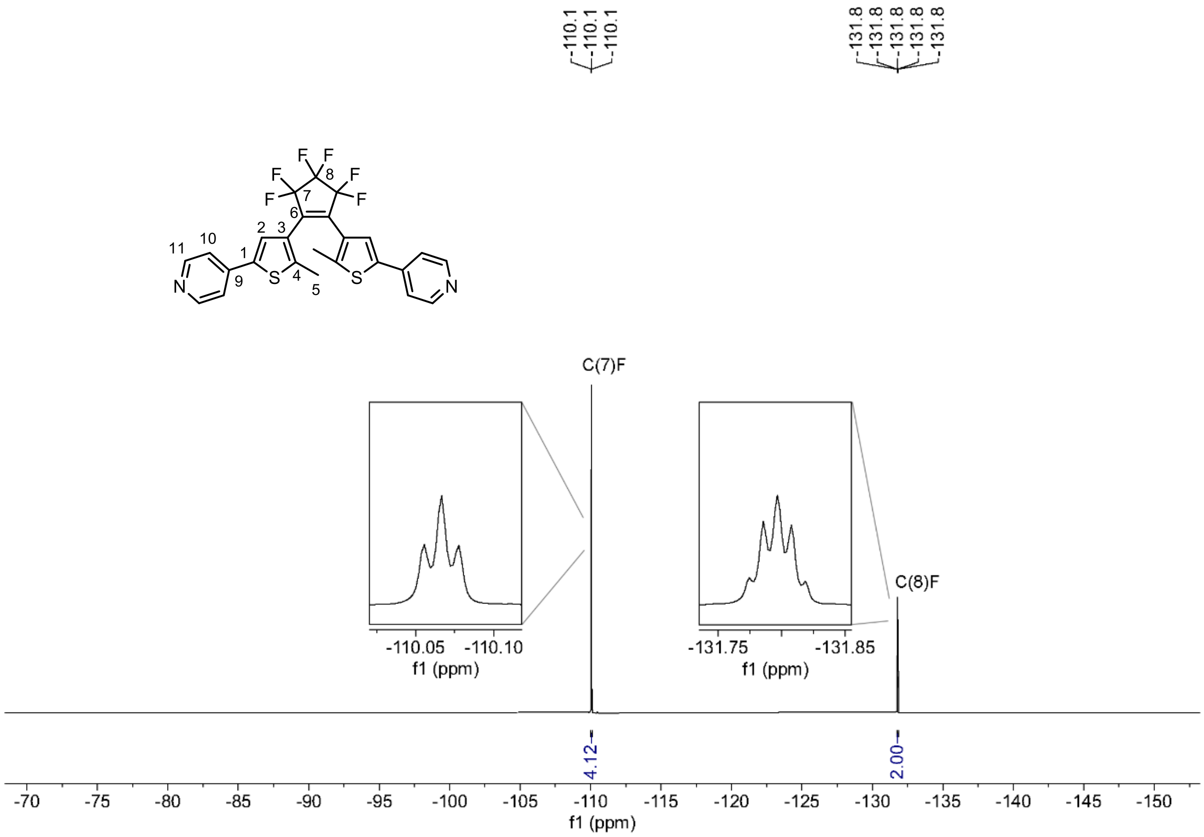

Figure S61 ^{19}F NMR spectrum (376 MHz, $CDCl_3$) of **A-A(p)**.

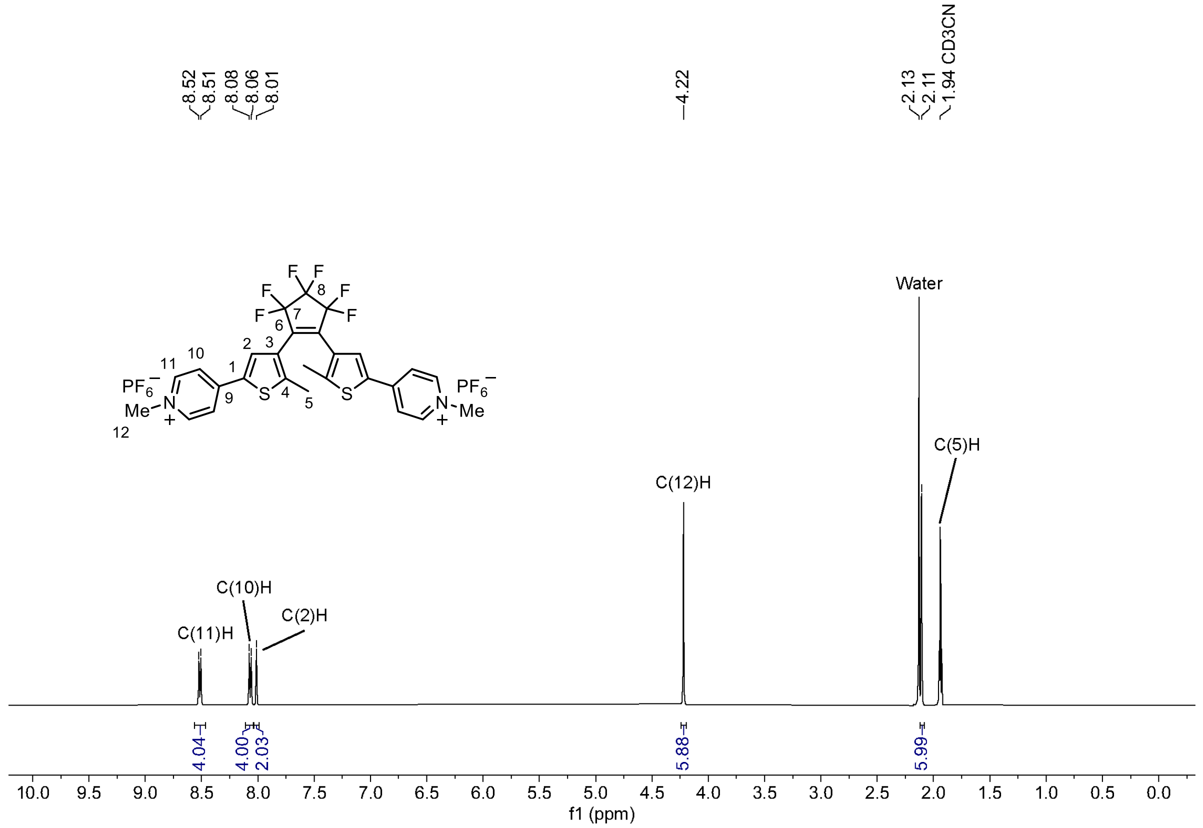

Figure S62 ^{1}H NMR spectrum (400 MHz, CD_3CN) of **A-A**.

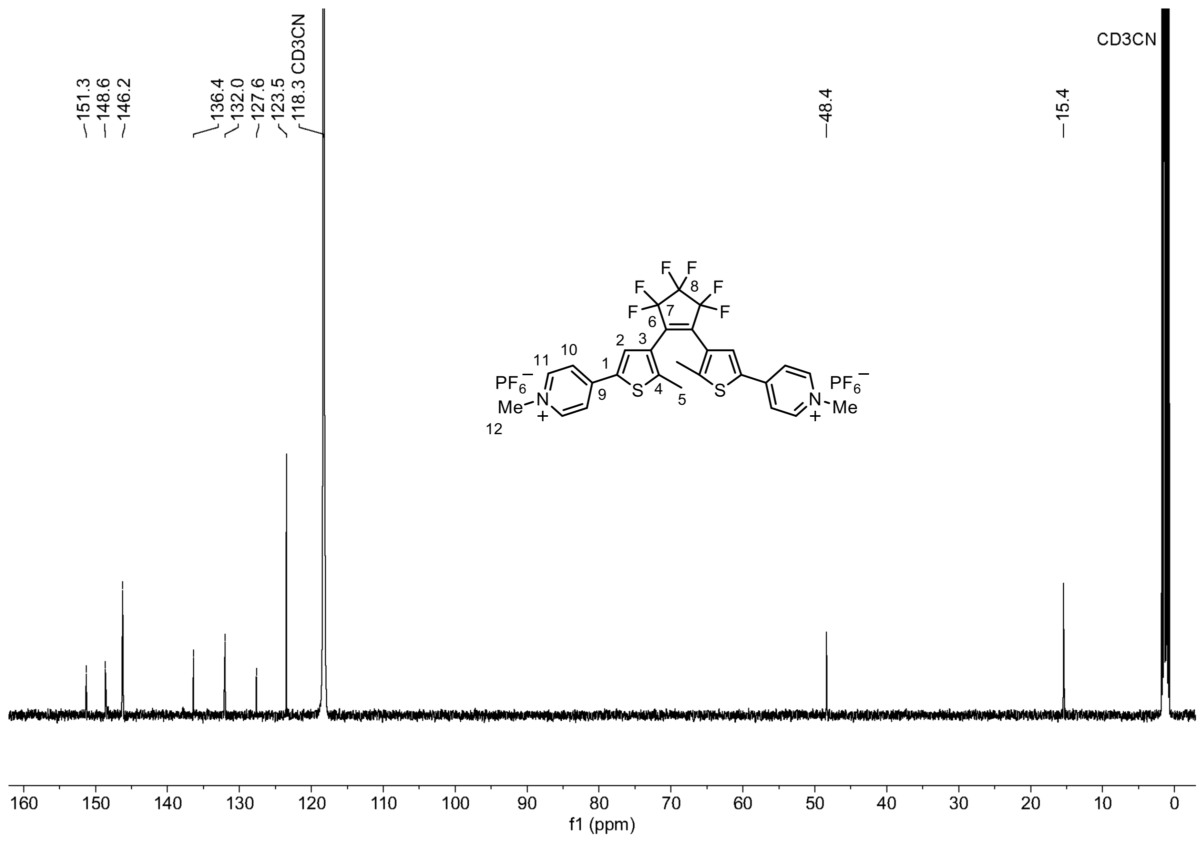

Figure S63 ^{13}C NMR spectrum (101 MHz, CD_3CN) of **A-A**.

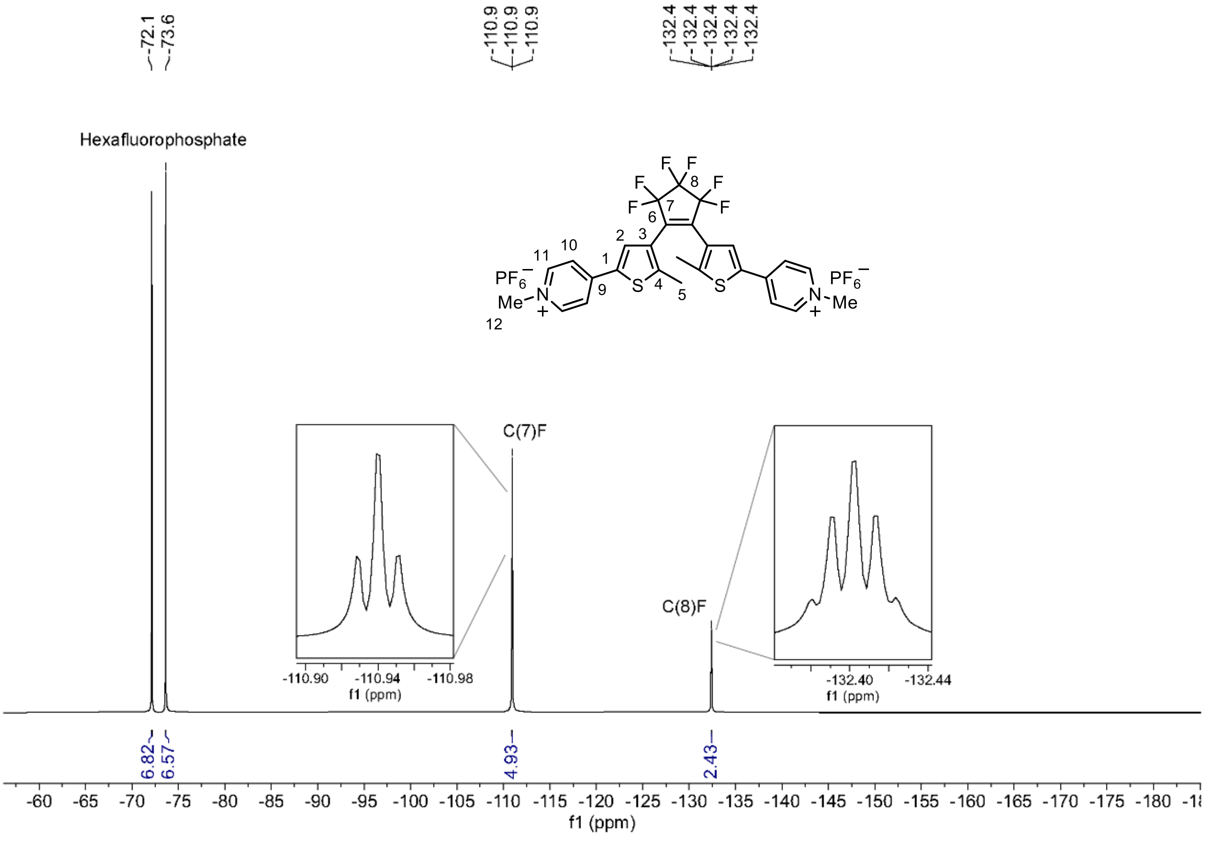

Figure S64 ^{19}F NMR spectrum (376 MHz, CD_3CN) of **A-A**.

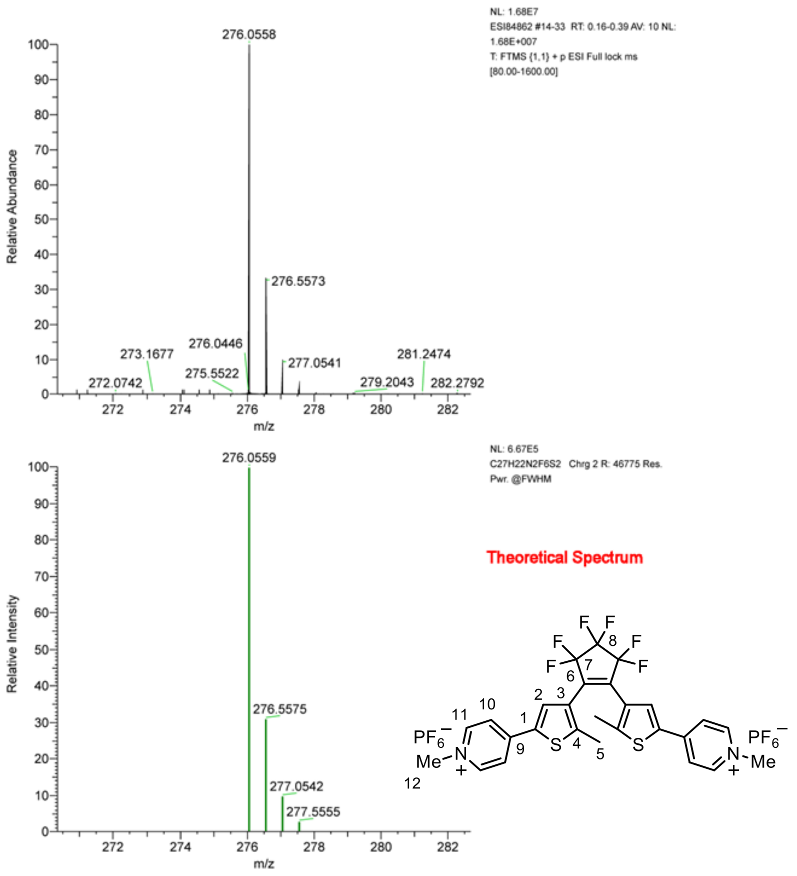

Figure S65 HRMS (ESI^+) of **A-A**, *m/z*: $[M]^{2+}$ calcd. for $C_{27}H_{22}F_6N_2S_2^{2+}$ 276.0559; found 276.0558.

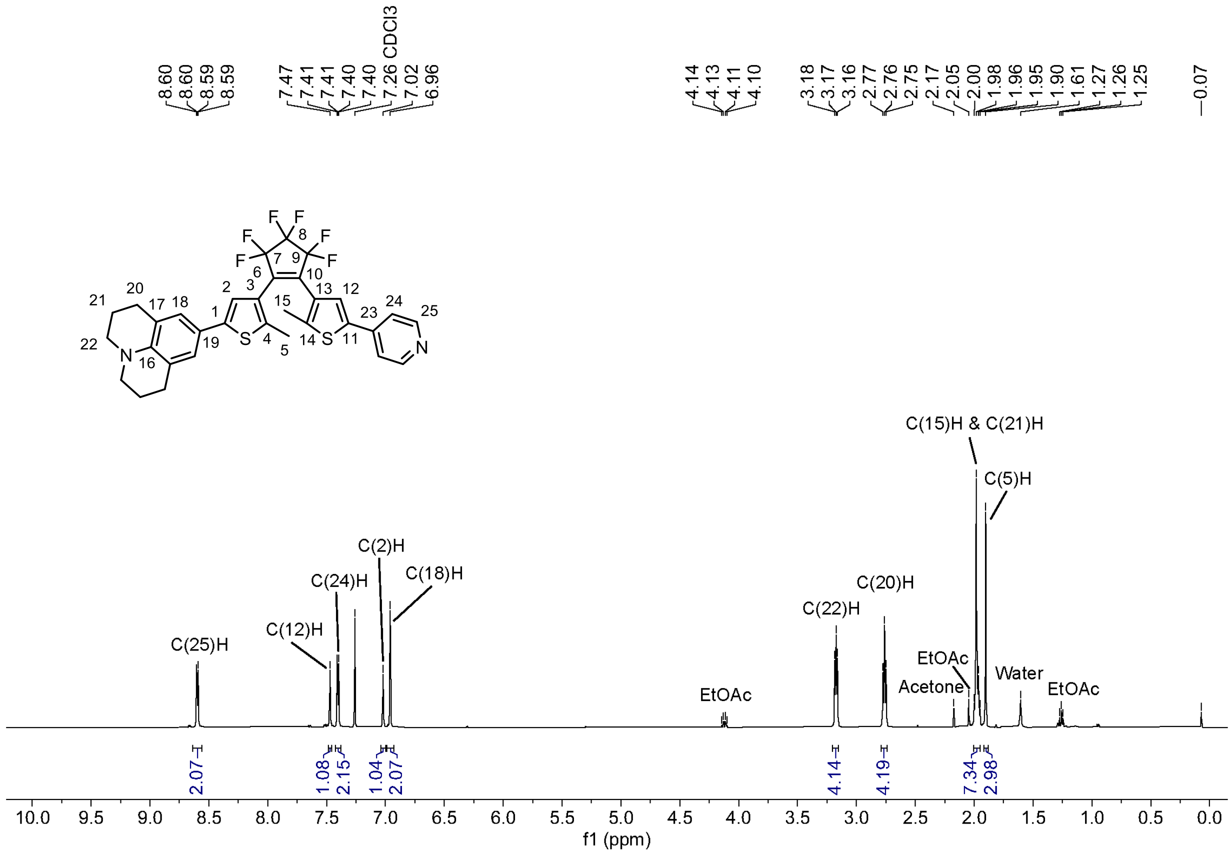

Figure S66 ^{1}H NMR spectrum (400 MHz, $CDCl_3$) of **D-A(p)**.

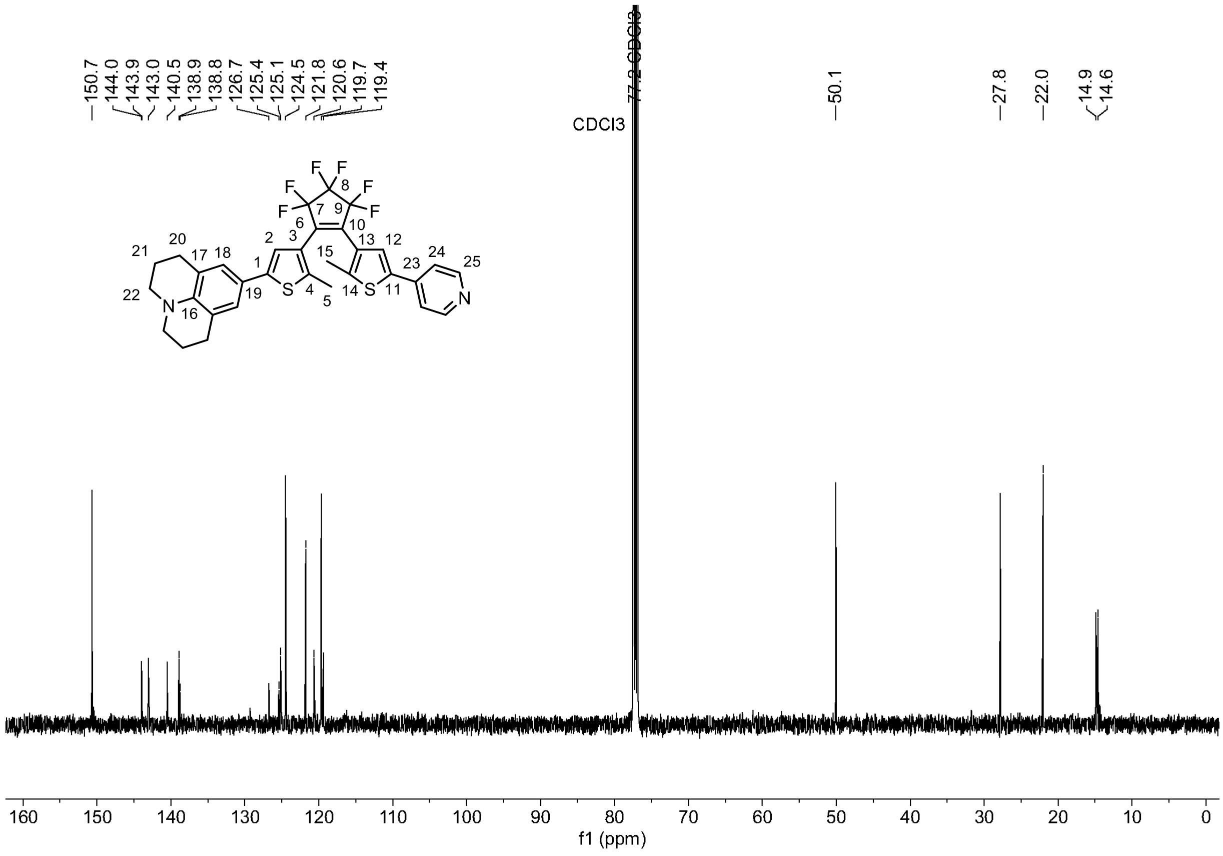

Figure S67 ^{13}C NMR spectrum (101 MHz, $CDCl_3$) of **D-A(p)**.

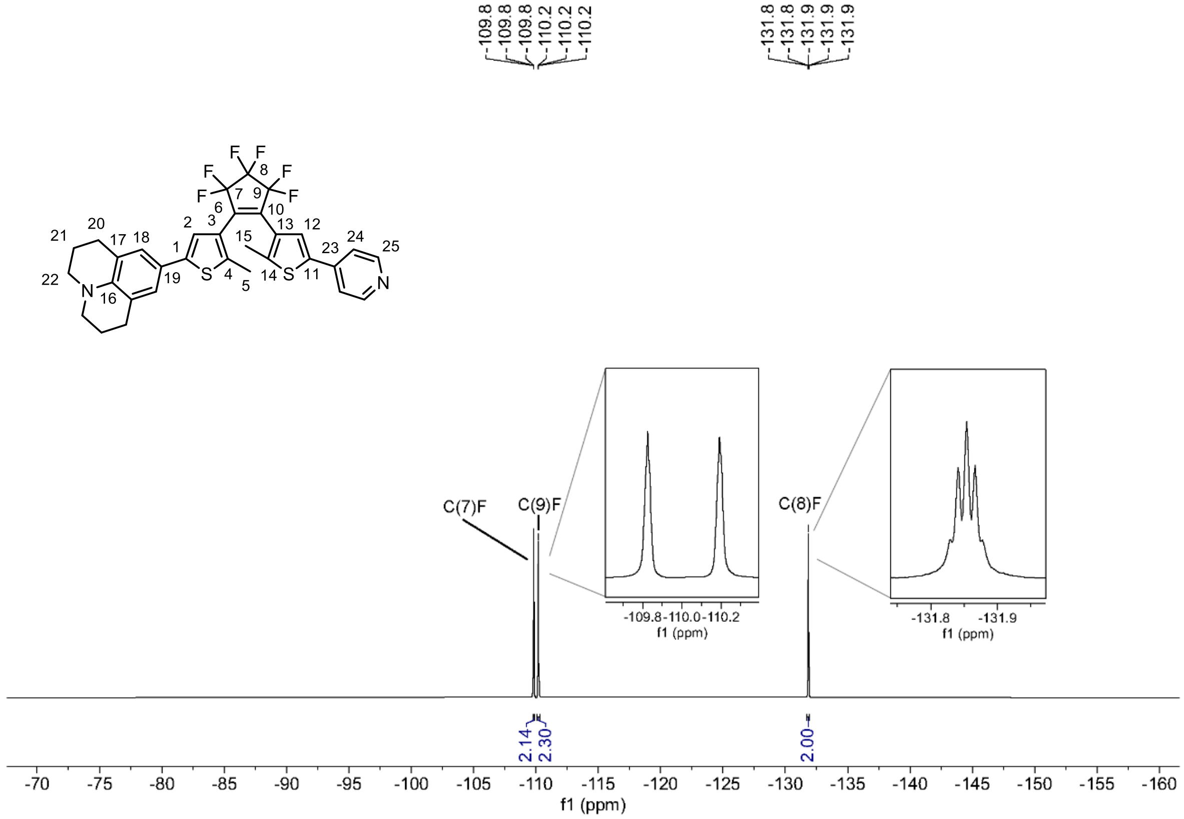

Figure S68 ^{19}F NMR spectrum (376 MHz, $CDCl_3$) of **D-A(p)**.

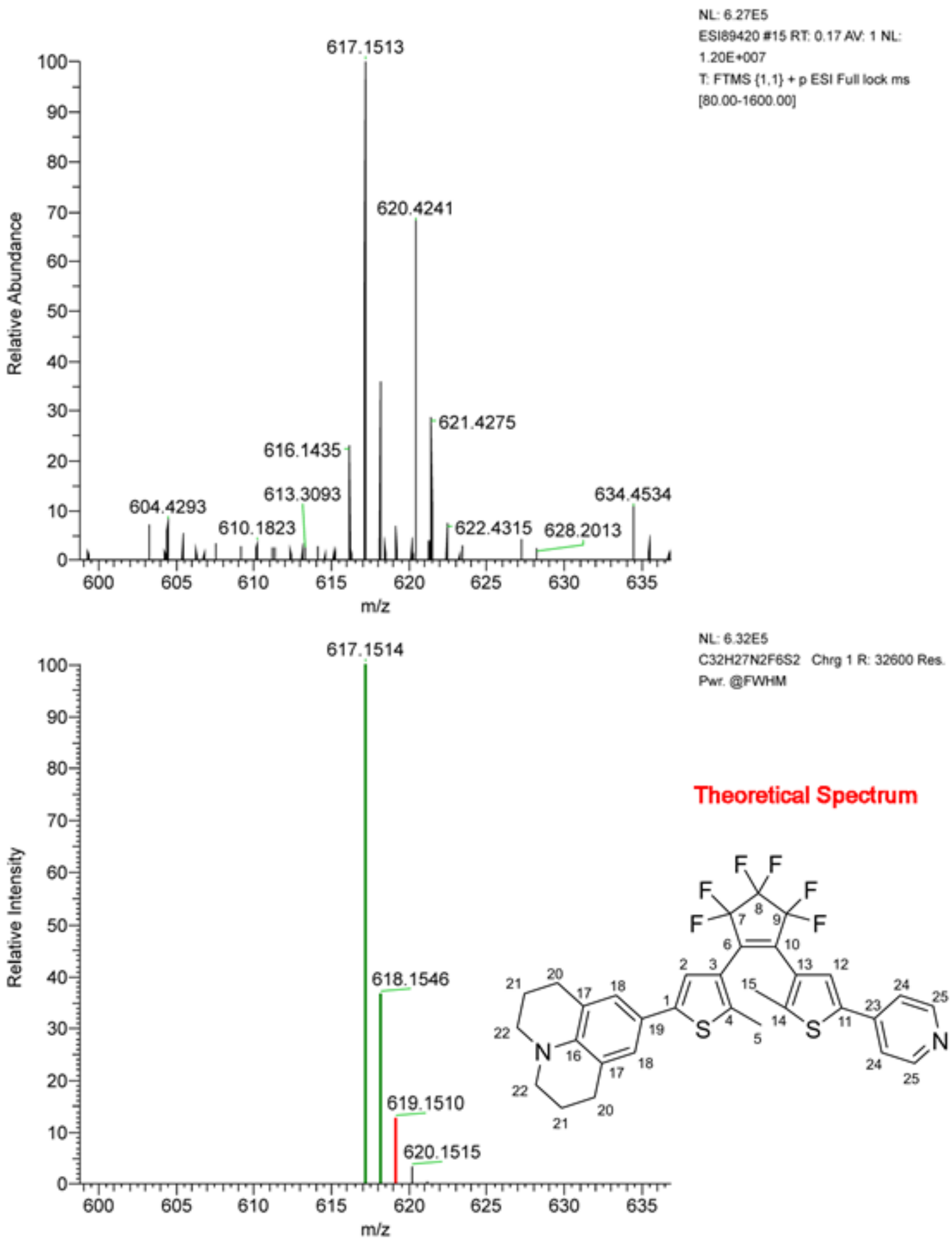

Figure S69 HRMS (ESI$^+$) *m/z*: $[M+H]^+$ calcd. for $C_{32}H_{27}F_6N_2S_2^+$ 617.1514; found 617.1513.

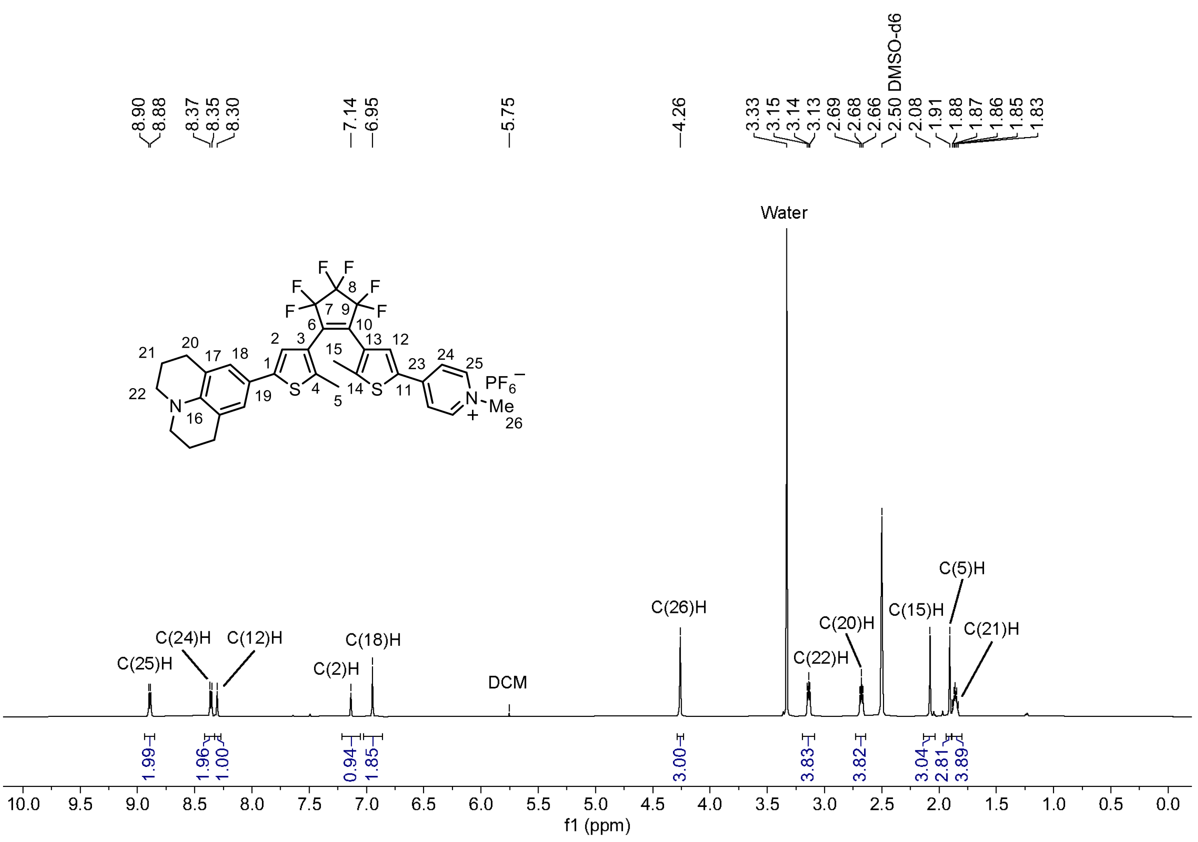

Figure S70 ^{1}H NMR spectrum (400 MHz, DMSO-d_6) of **D-A**.

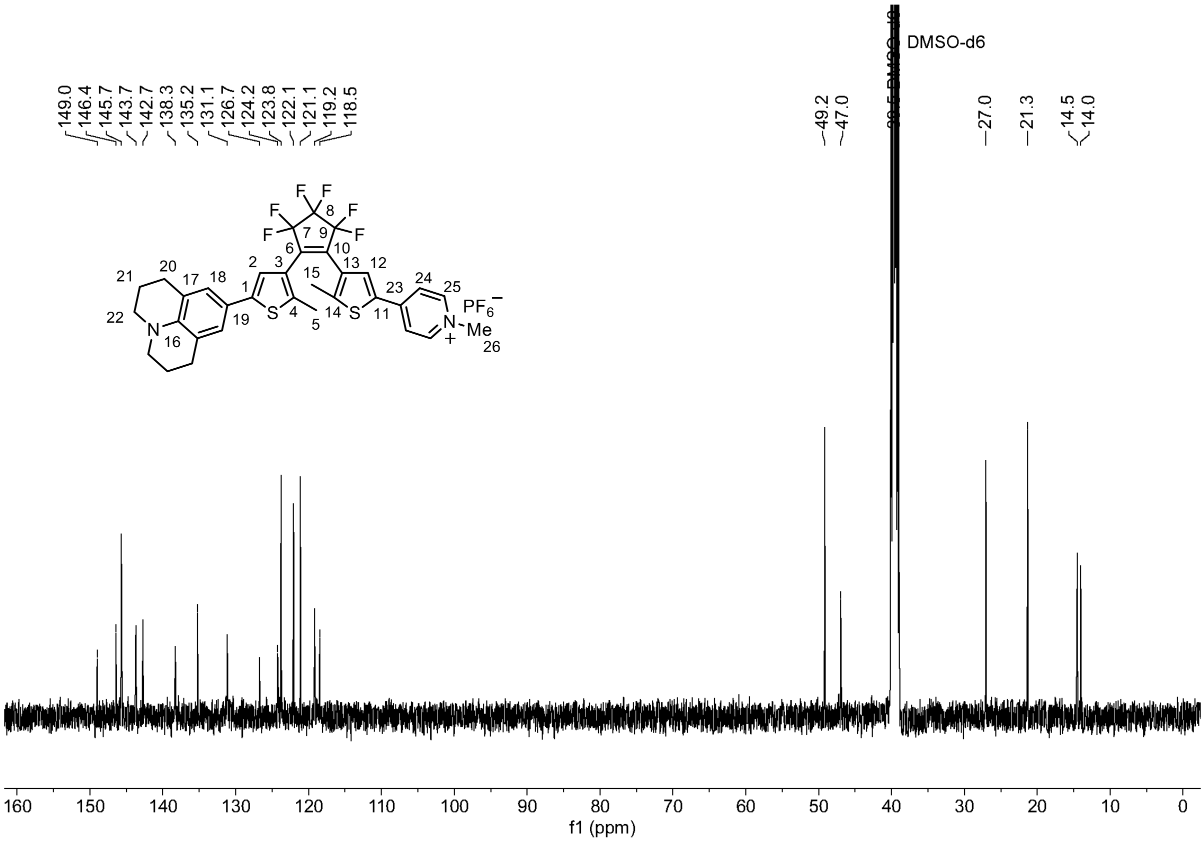

Figure S71 ^{13}C NMR spectrum (101 MHz, DMSO-d_6) of **D-A**.

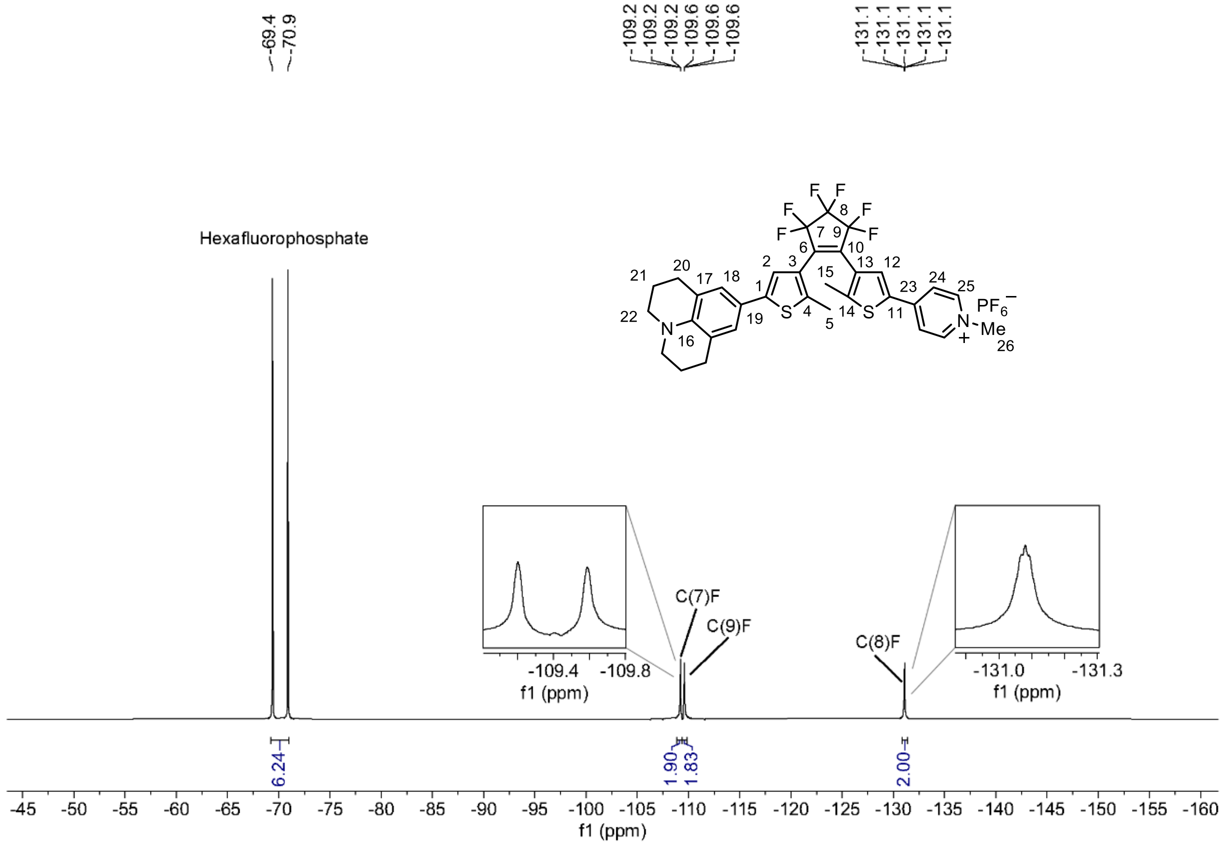

Figure S72 ^{19}F NMR spectrum (376 MHz, DMSO-d_6) of **D-A**.

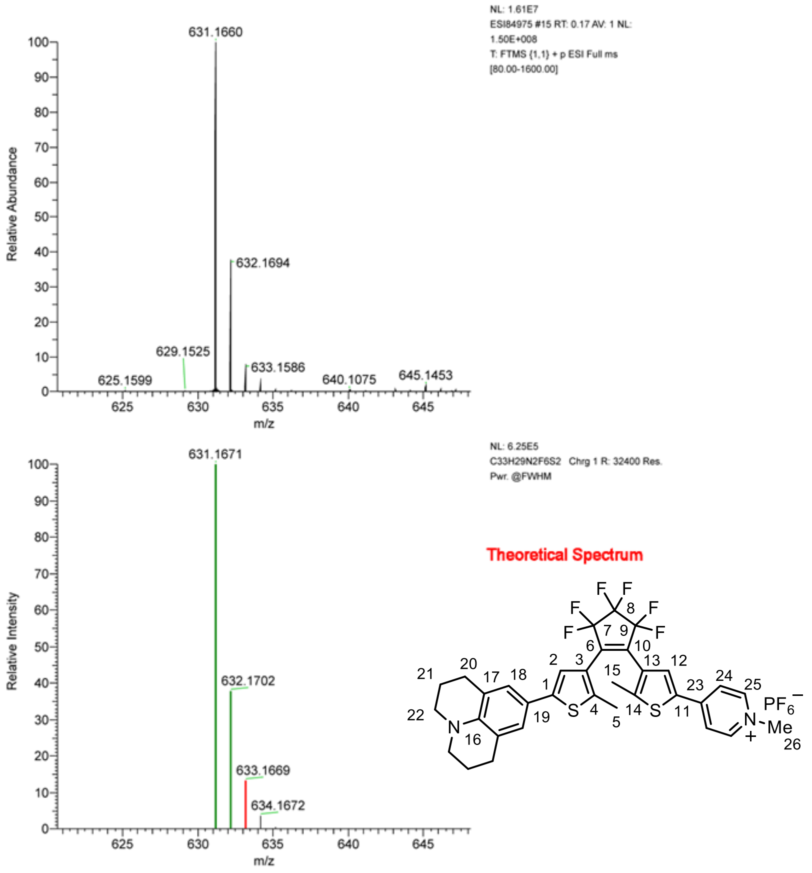

Figure S73 HRMS (ESI$^+$) of **D-A**, *m/z*: $[M]^+$ calcd. for $C_{33}H_{29}F_6N_2S_2^+$ 631.1671; found 631.1660.

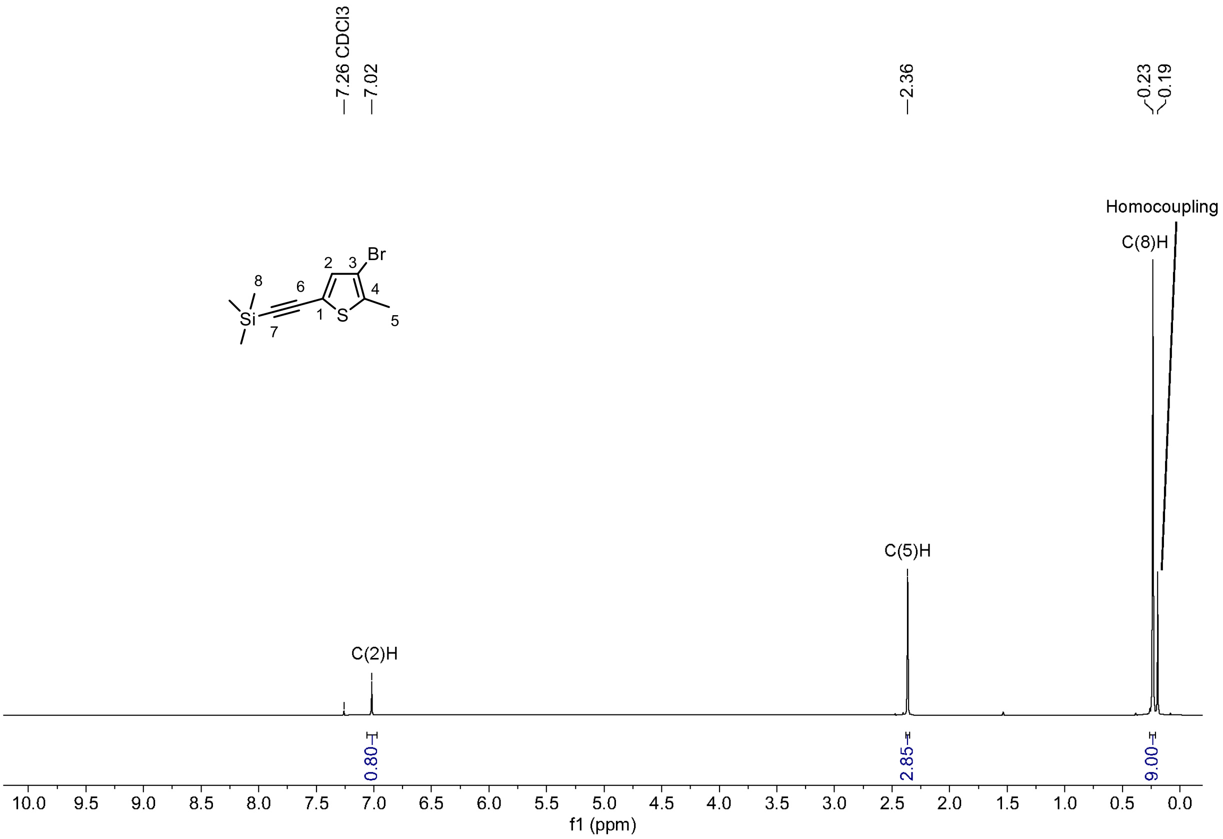

Figure S74 ^{1}H NMR spectrum (400 MHz, $CDCl_3$) of **11a**.

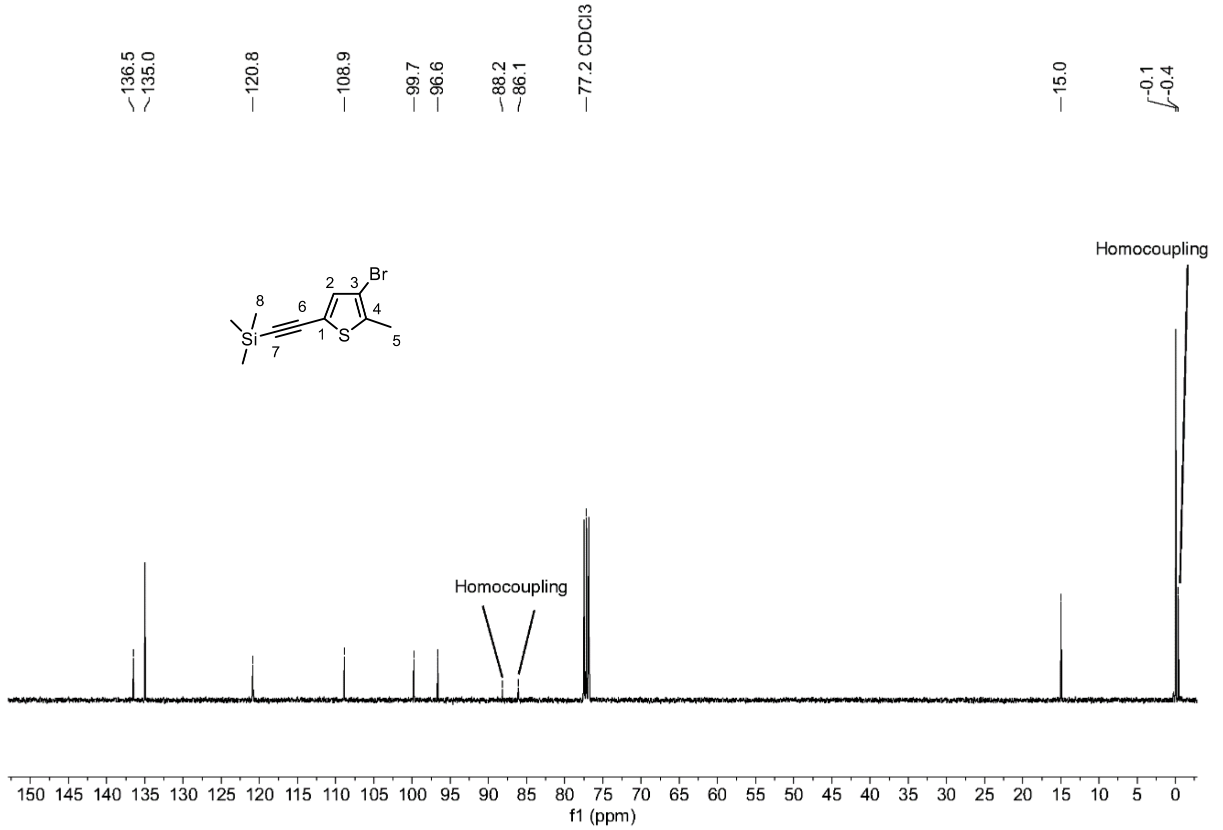

Figure S75 ^{13}C NMR spectrum (101 MHz, $CDCl_3$) of **11a**.

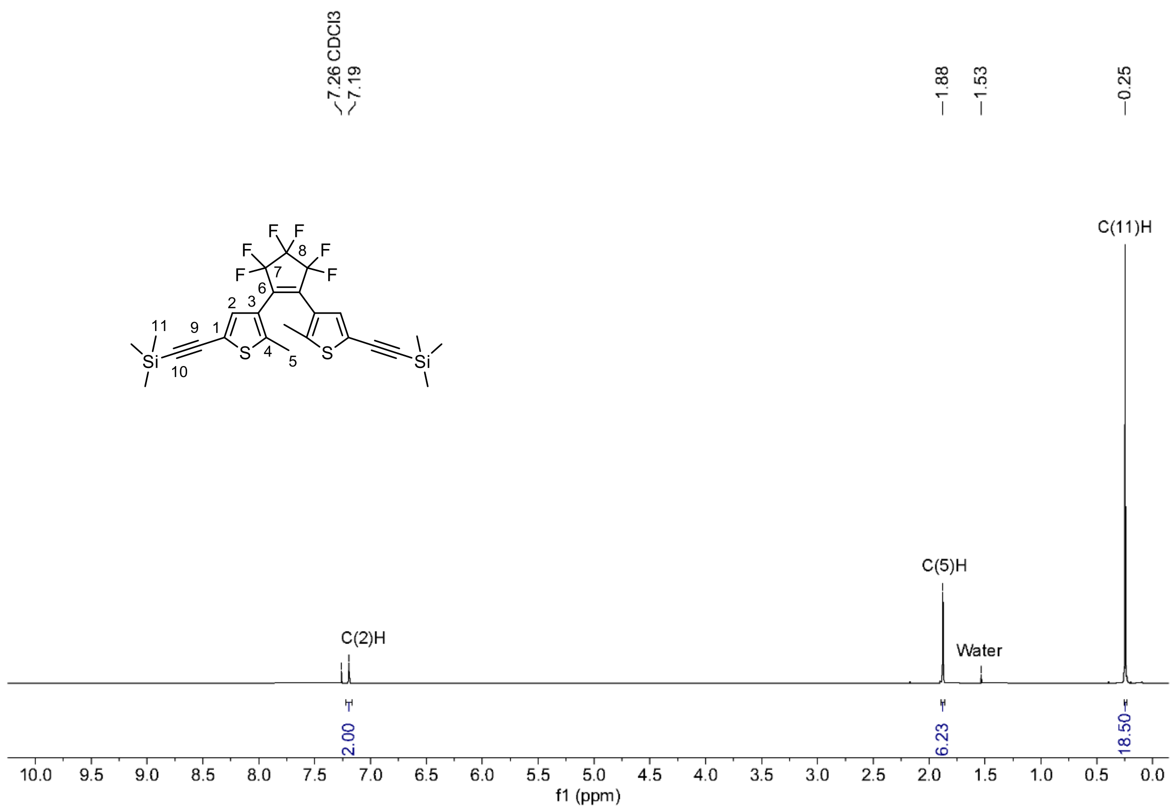

Figure S76 ^{1}H NMR spectrum (400 MHz, $CDCl_3$) of **11b**.

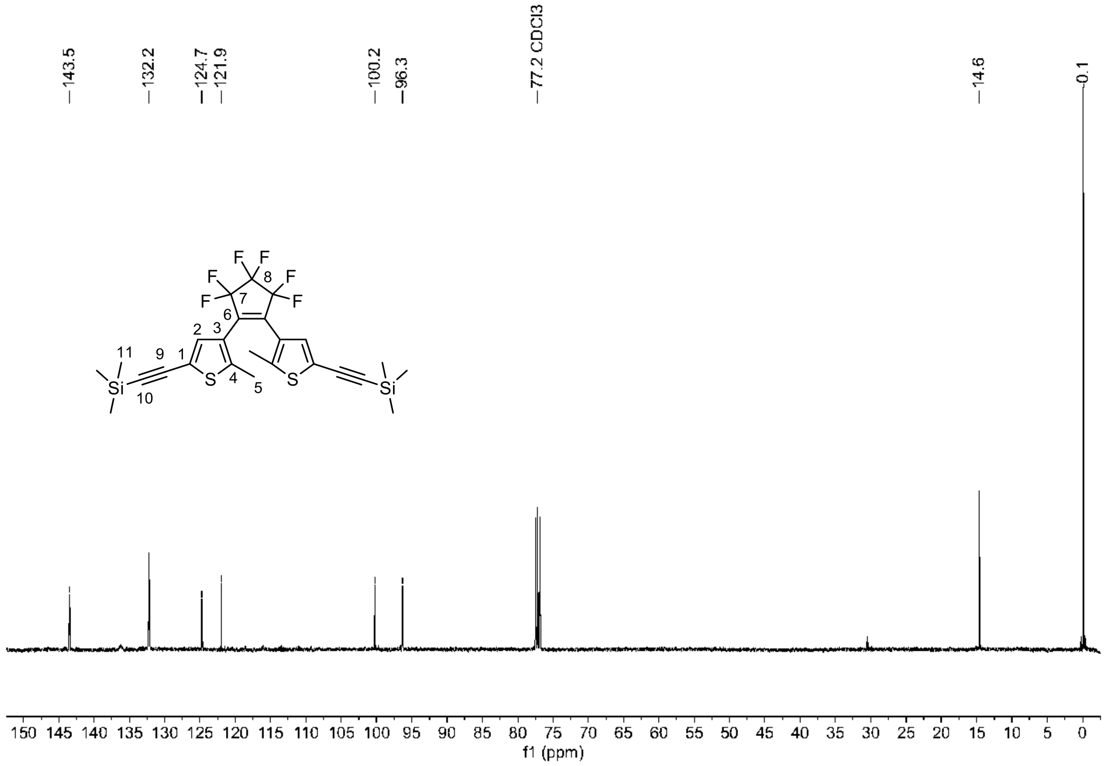

Figure S77 ^{13}C NMR spectrum (101 MHz, $CDCl_3$) of **11b**.

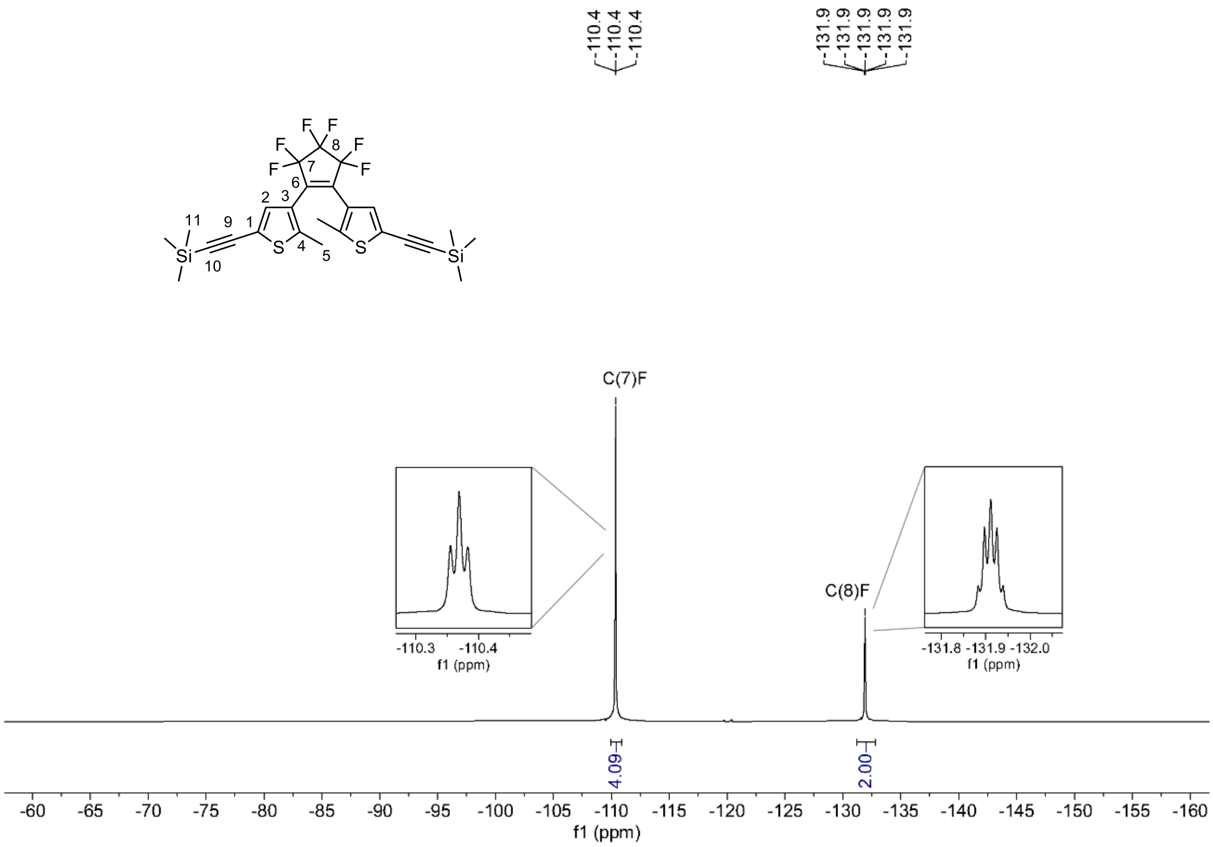

Figure S78 ^{19}F NMR spectrum (376 MHz, $CDCl_3$) of **11b**.

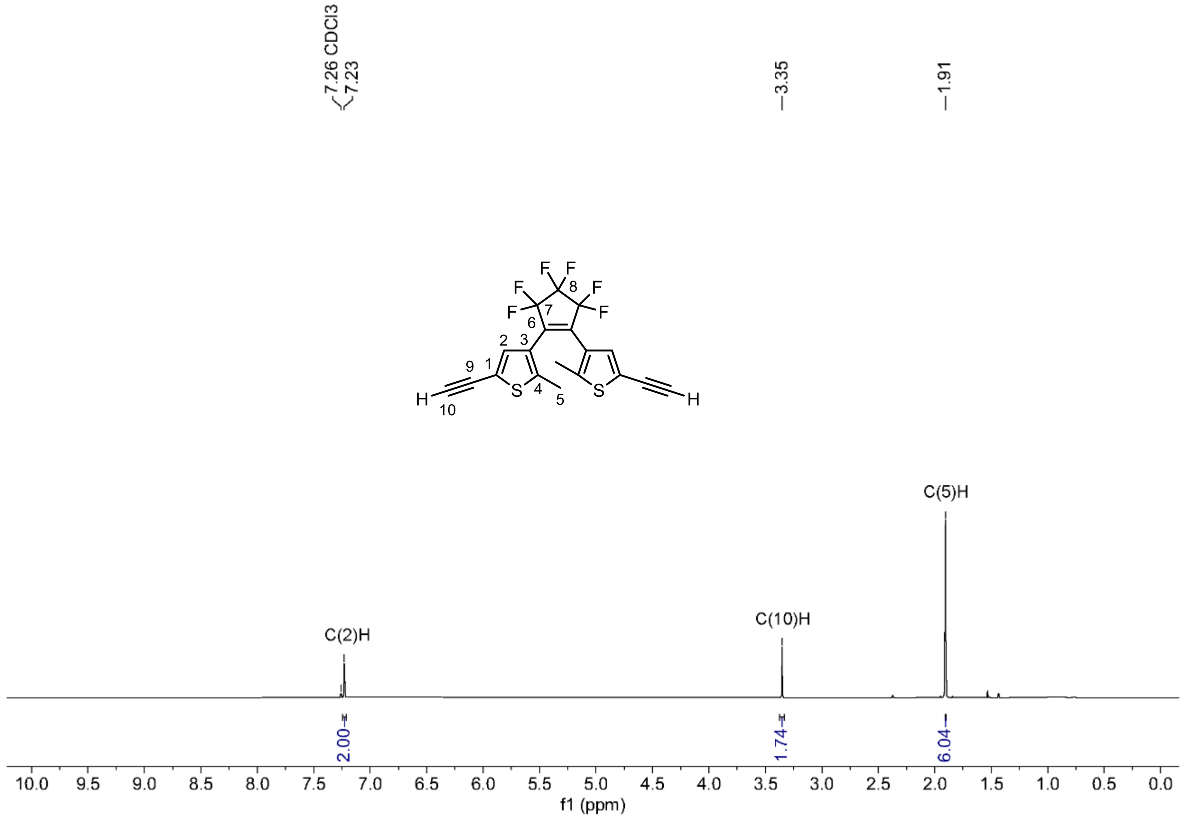

Figure S79 ^{1}H NMR spectrum (400 MHz, $CDCl_3$) of **11**.

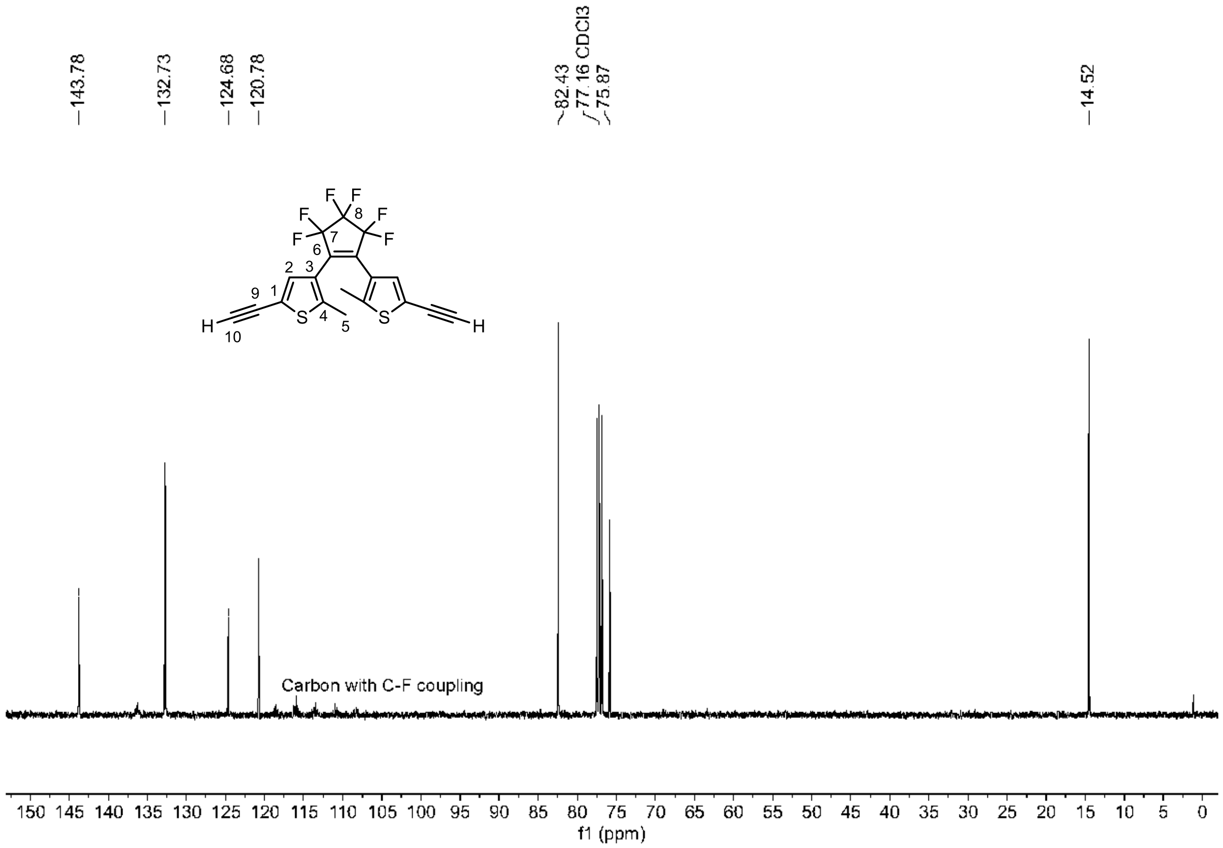

Figure S80 ^{13}C NMR spectrum (101 MHz, $CDCl_3$) of **11**.

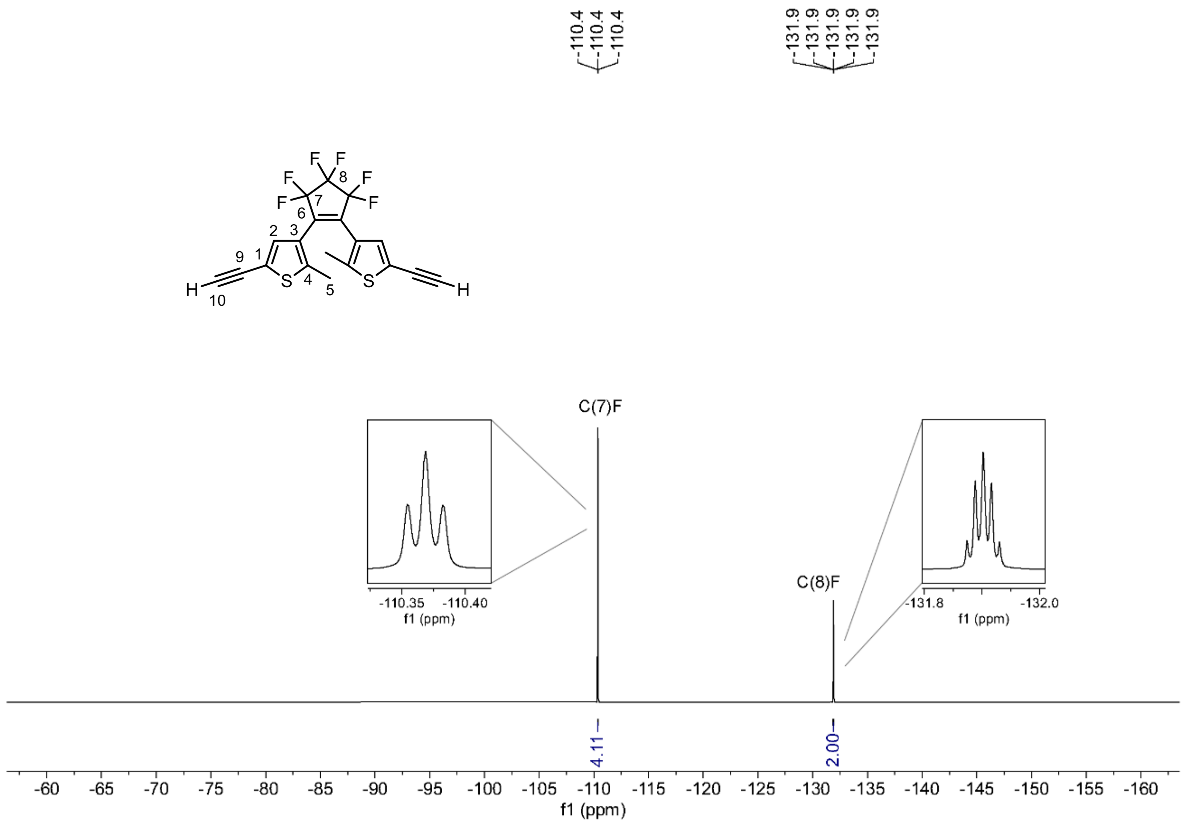

Figure S81 ^{19}F NMR spectrum (376 MHz, $CDCl_3$) of **11**.

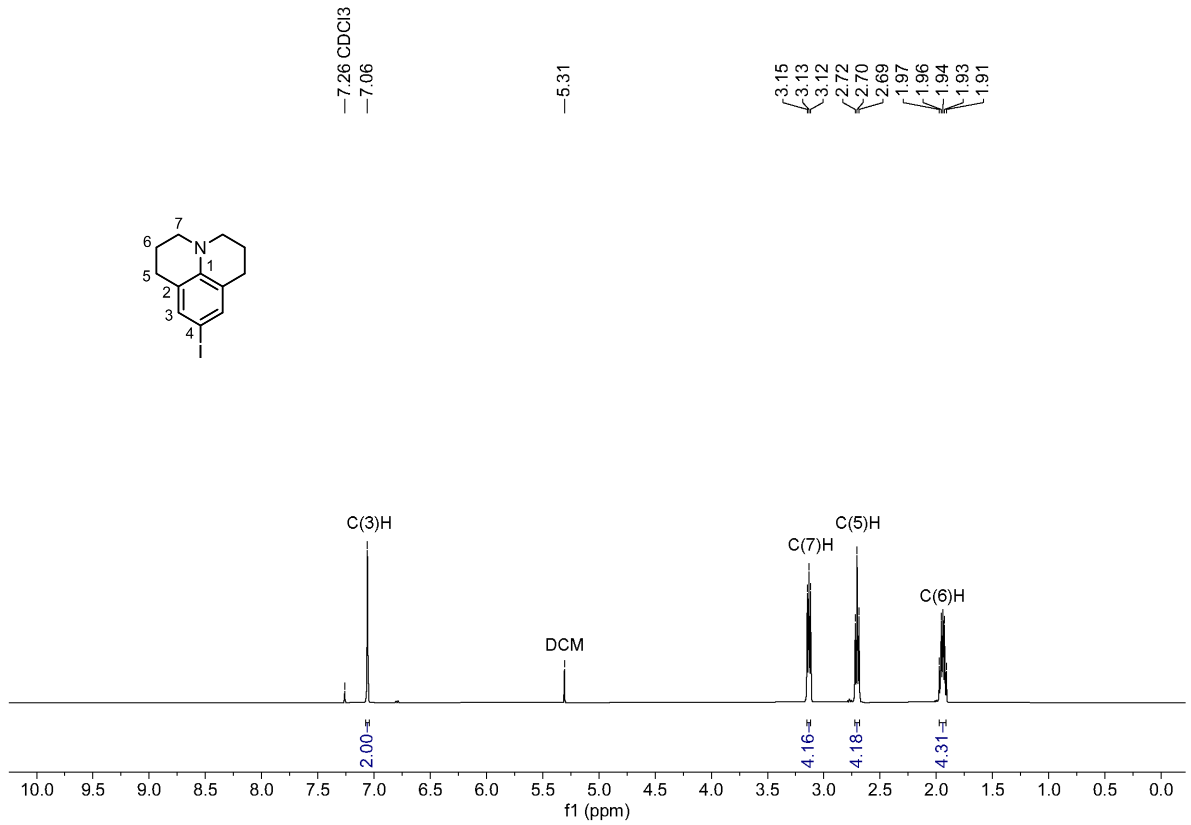

Figure S82 ^{1}H NMR spectrum (400 MHz, $CDCl_3$) of **5**.

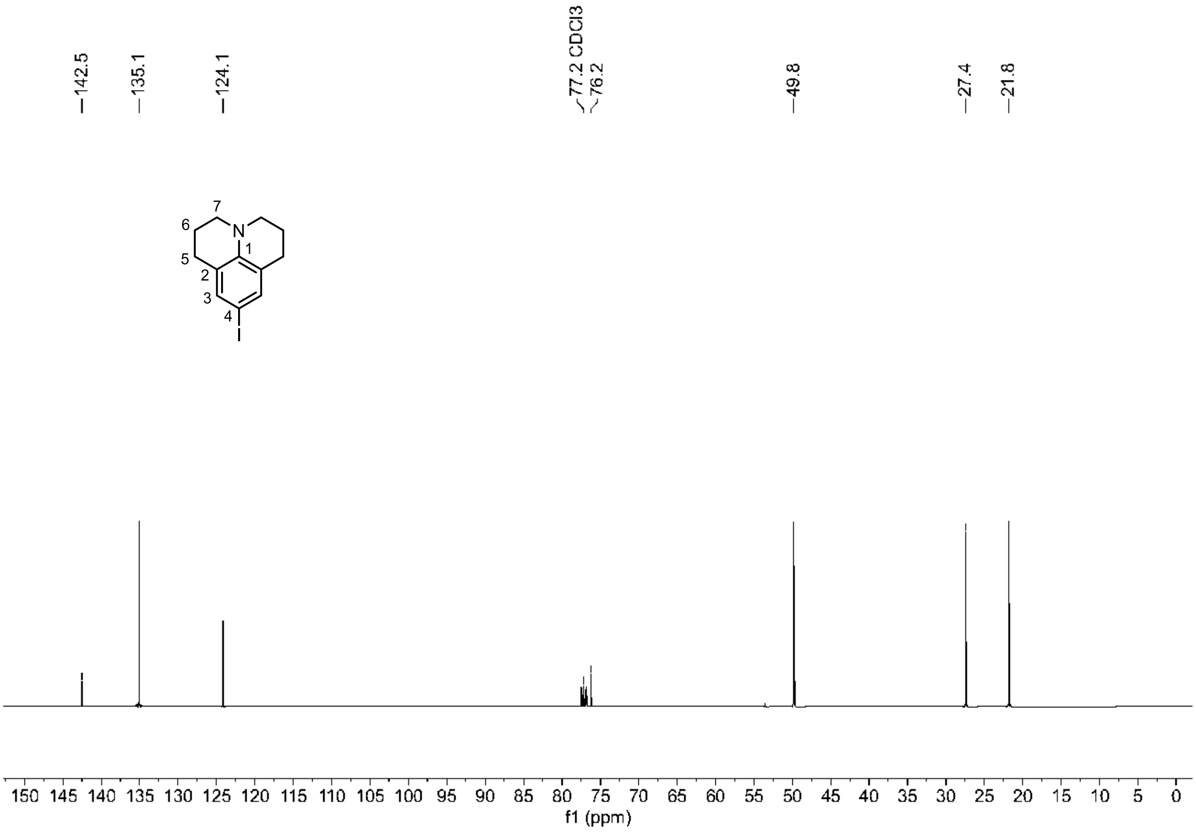

Figure S83 ^{13}C NMR spectrum (101 MHz, $CDCl_3$) of **5**.

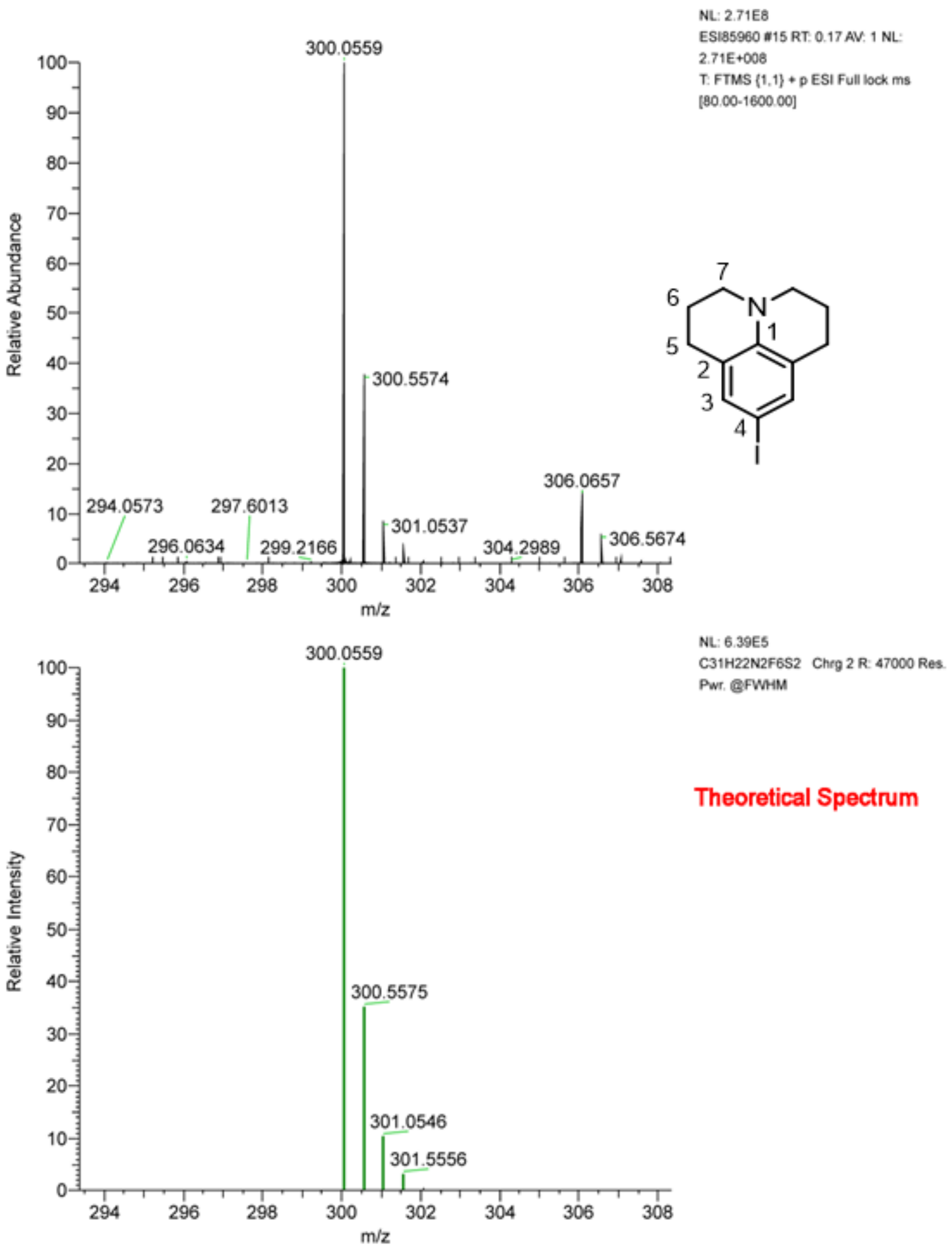

Figure S84 HRMS (ESI$^+$) of **5**, *m/z*: $[M+H]^+$ calcd. for $C_{31}H_{22}F_6N_2S_2$ 300.0559; found 300.0559.

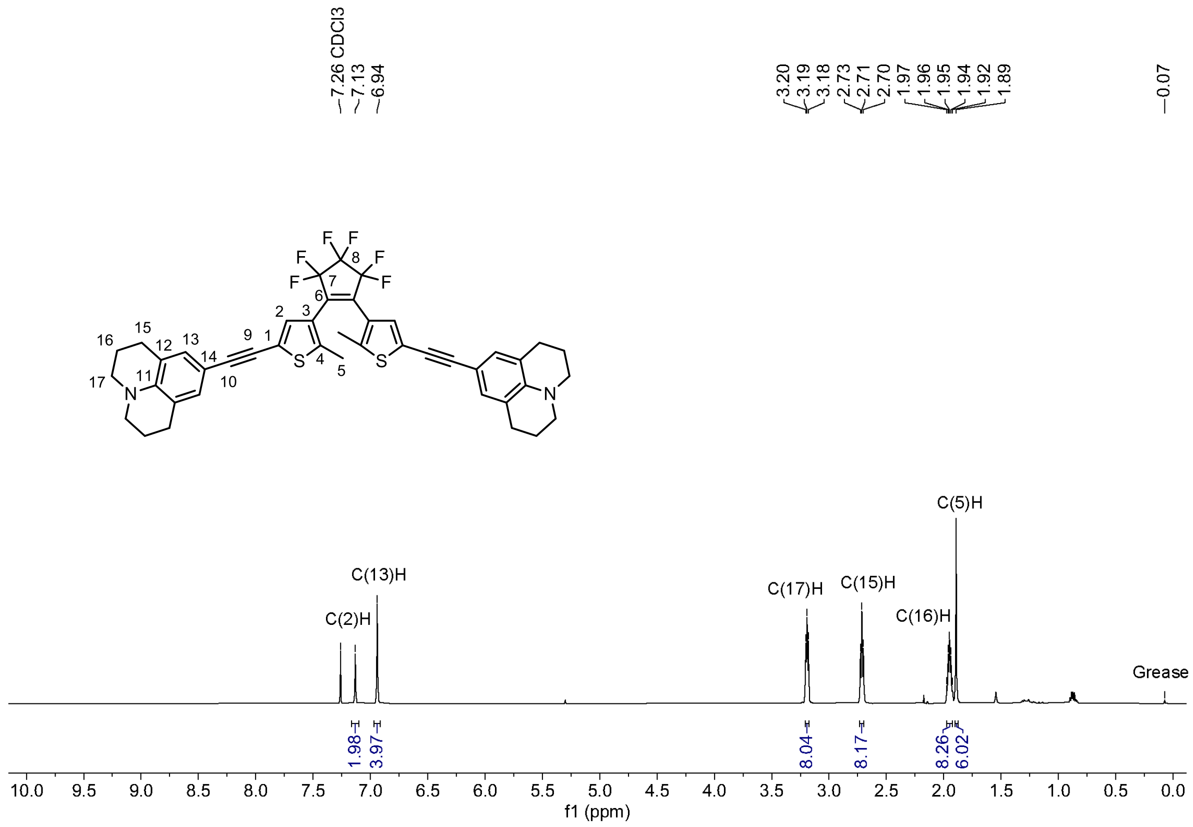

Figure S85 ^{1}H NMR spectrum (400 MHz, $CDCl_3$) of **Dy-yD**.

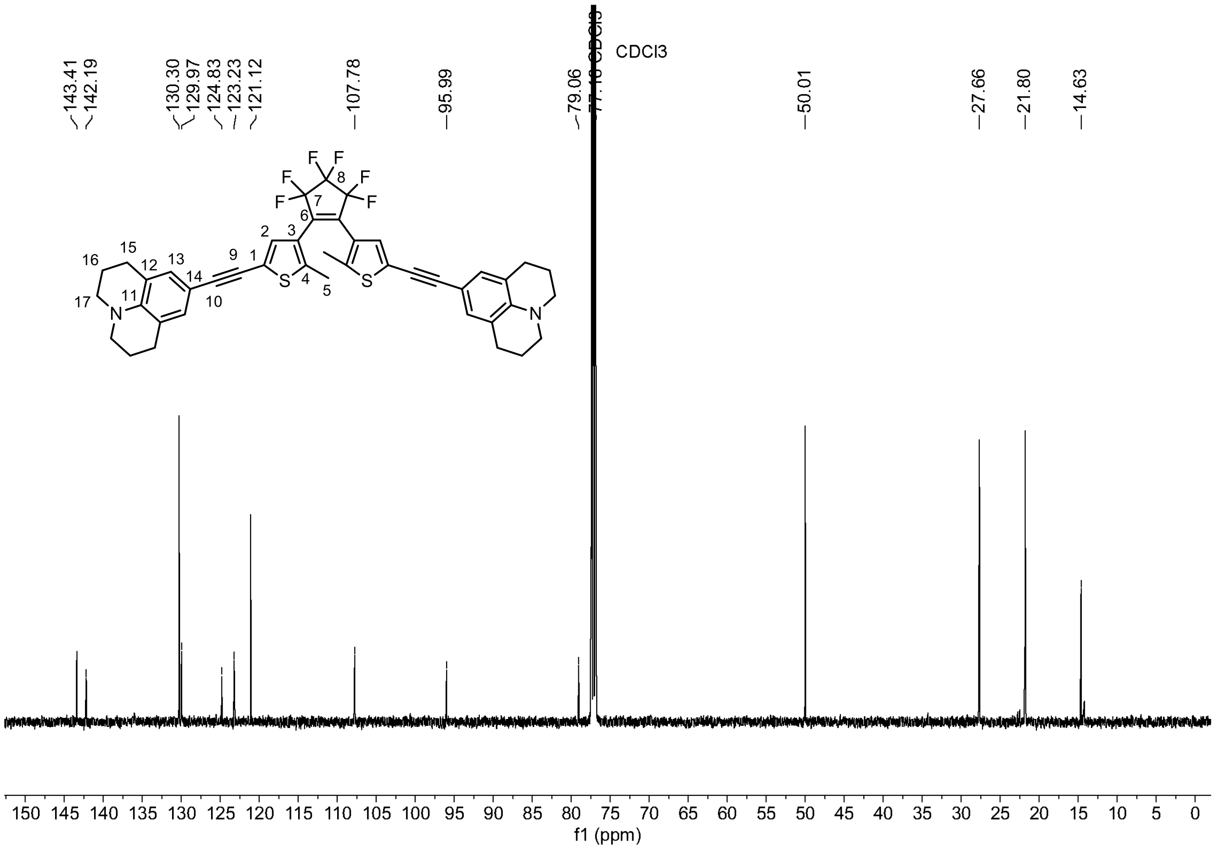

Figure S86 ^{13}C NMR spectrum (101 MHz, $CDCl_3$) of **Dy-yD**.

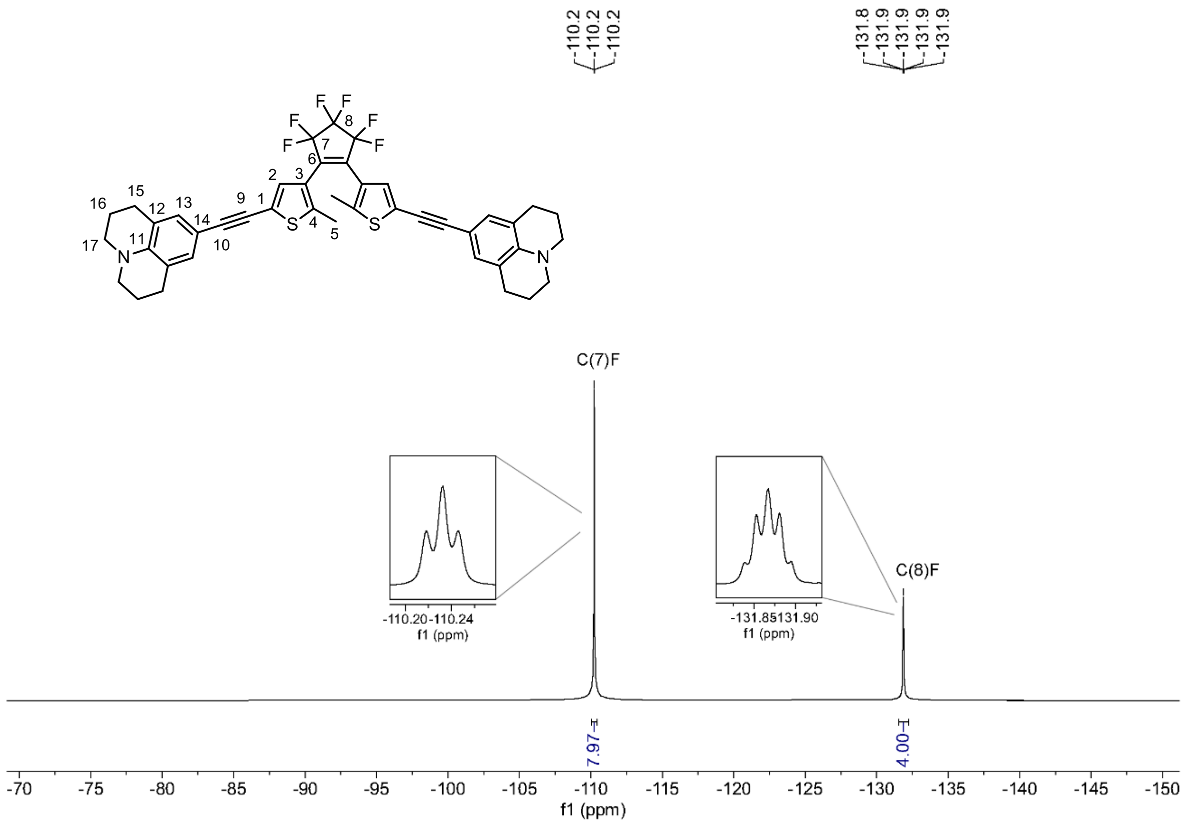

Figure S87 ^{19}F NMR spectrum (376 MHz, $CDCl_3$) of **Dy-yD**.

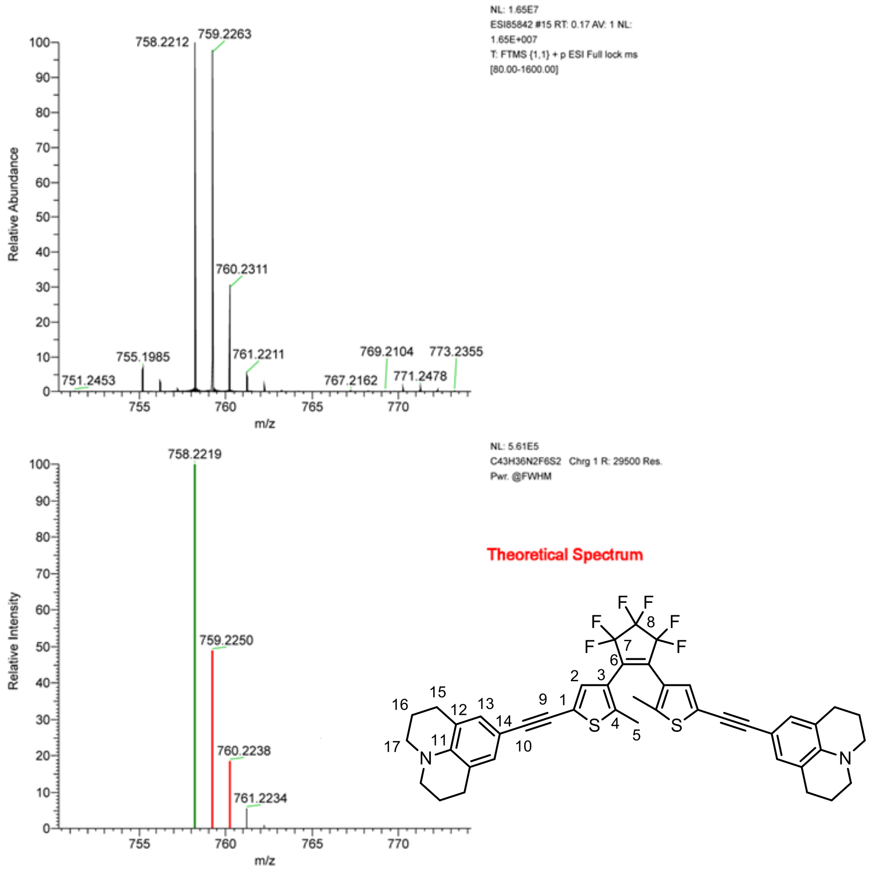

Figure S88 HRMS (ESI$^+$) of **Dy-yD**, *m/z*: $[M+H]^+$ calcd. for $C_{43}H_{36}F_6N_2S_2^+$ 758.2219; found 758.2212.

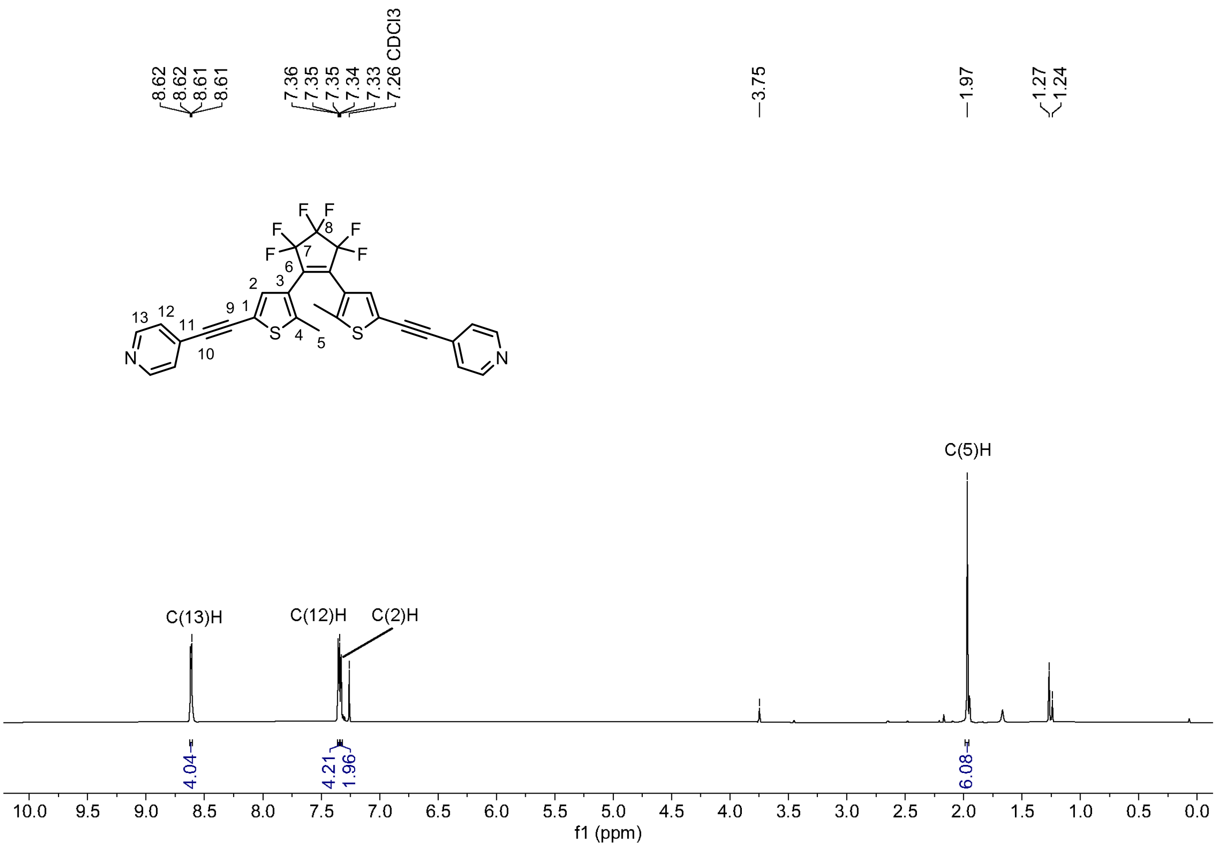

Figure S89 ^{1}H NMR spectrum (400 MHz, $CDCl_3$) of **Ay-yA(p)**.

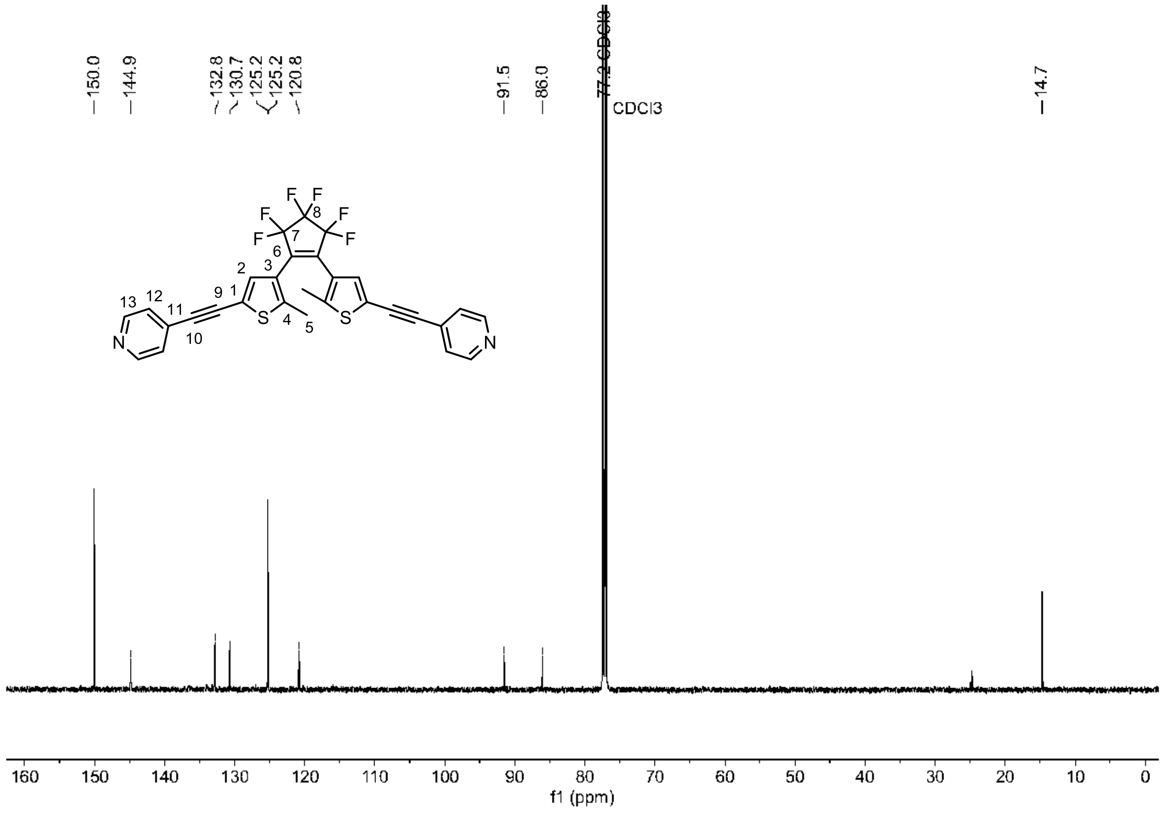

Figure S90 ^{13}C NMR spectrum (101 MHz, $CDCl_3$) of **Ay-yA(p)**.

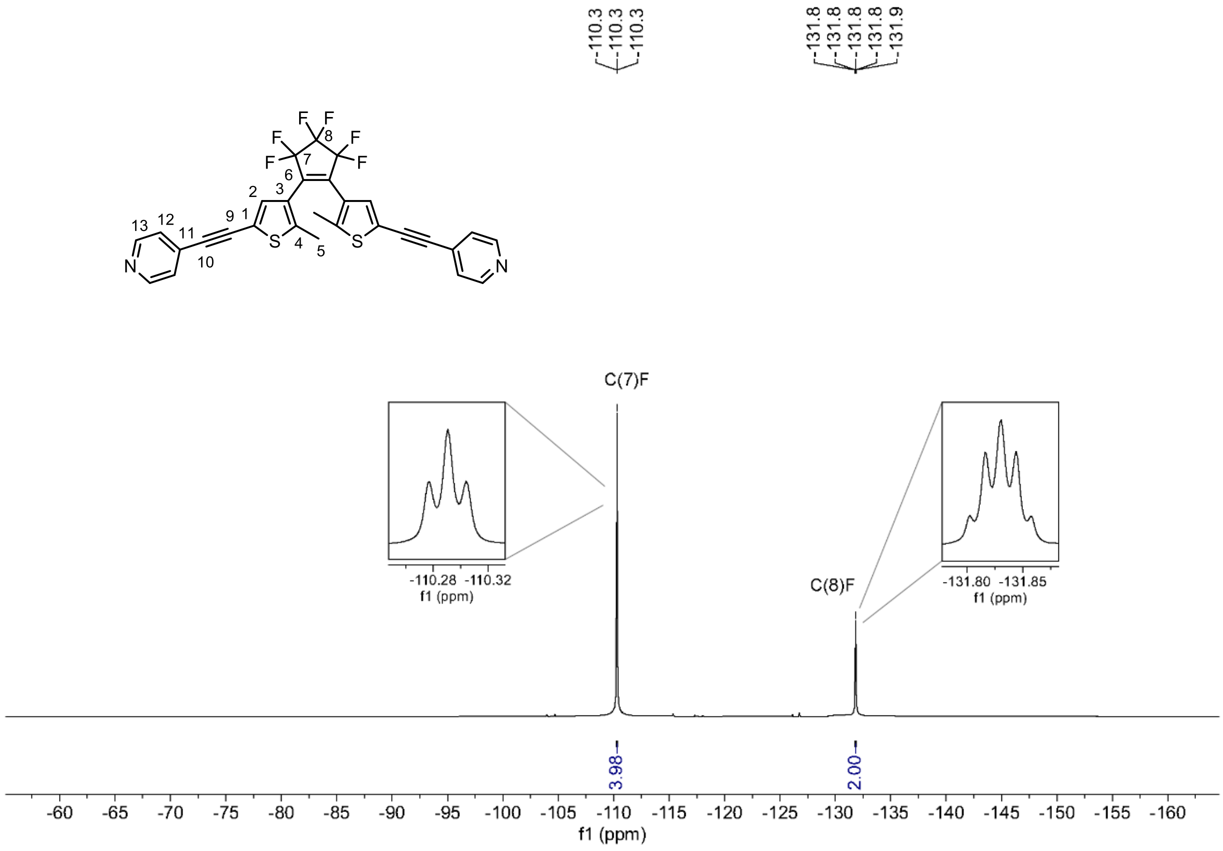

Figure S91 ^{19}F NMR spectrum (376 MHz, $CDCl_3$) of **Ay-yA(p)**.

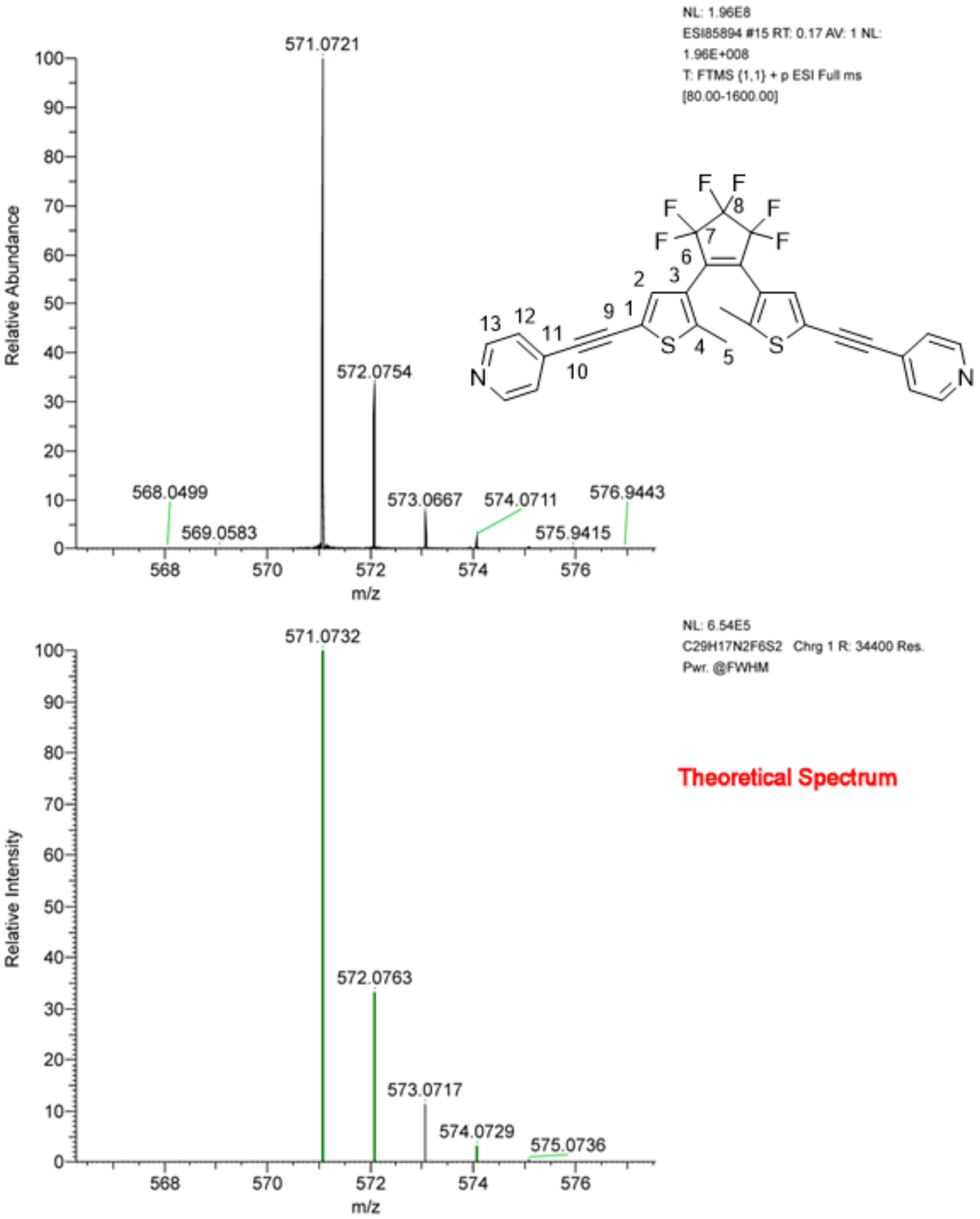

Figure S92 HRMS (ESI$^+$) of **Ay-yA(p)** *m/z*: $[M+H]^+$ calcd. for $C_{29}H_{17}F_6N_2S_2^+$ 571.0732; found 571.0721.

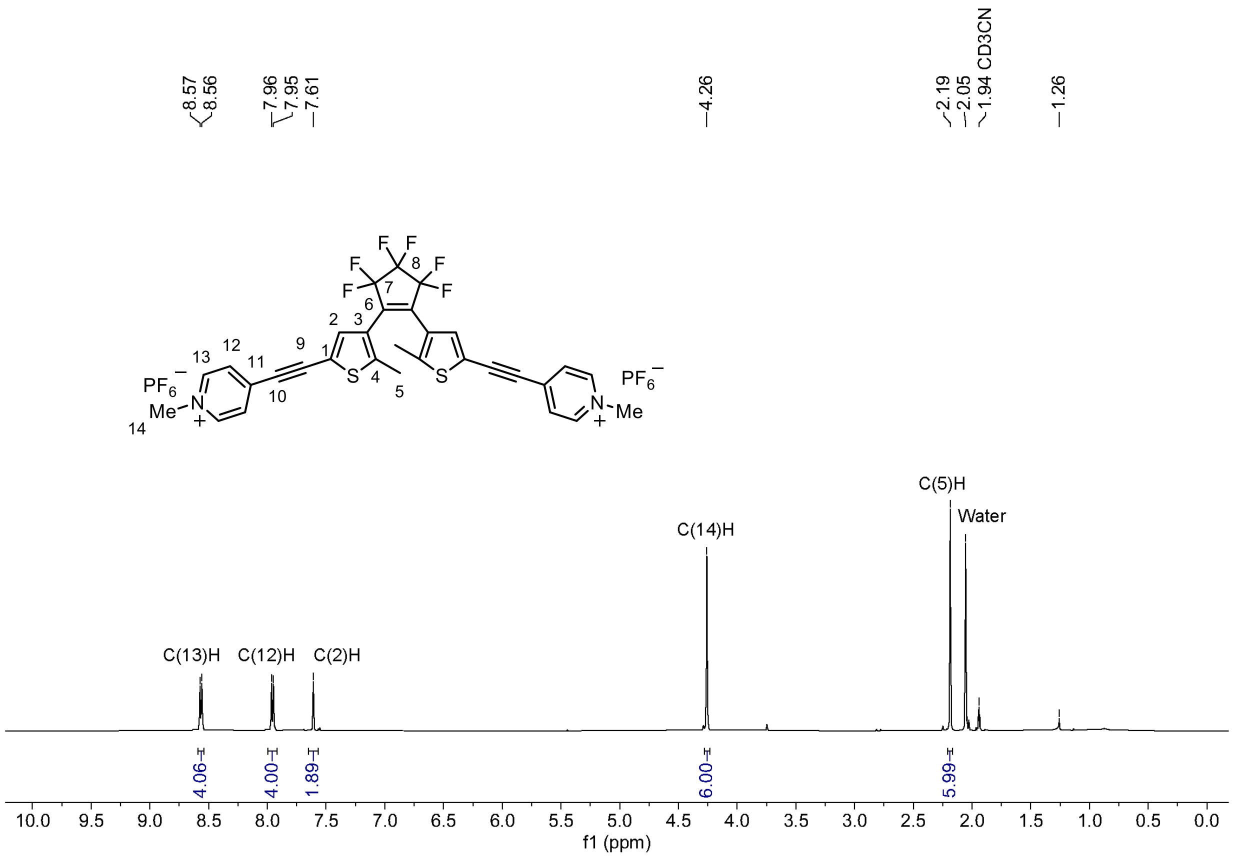

Figure S93 ^{1}H NMR spectrum (400 MHz, CD3CN) of **Ay-yA**.

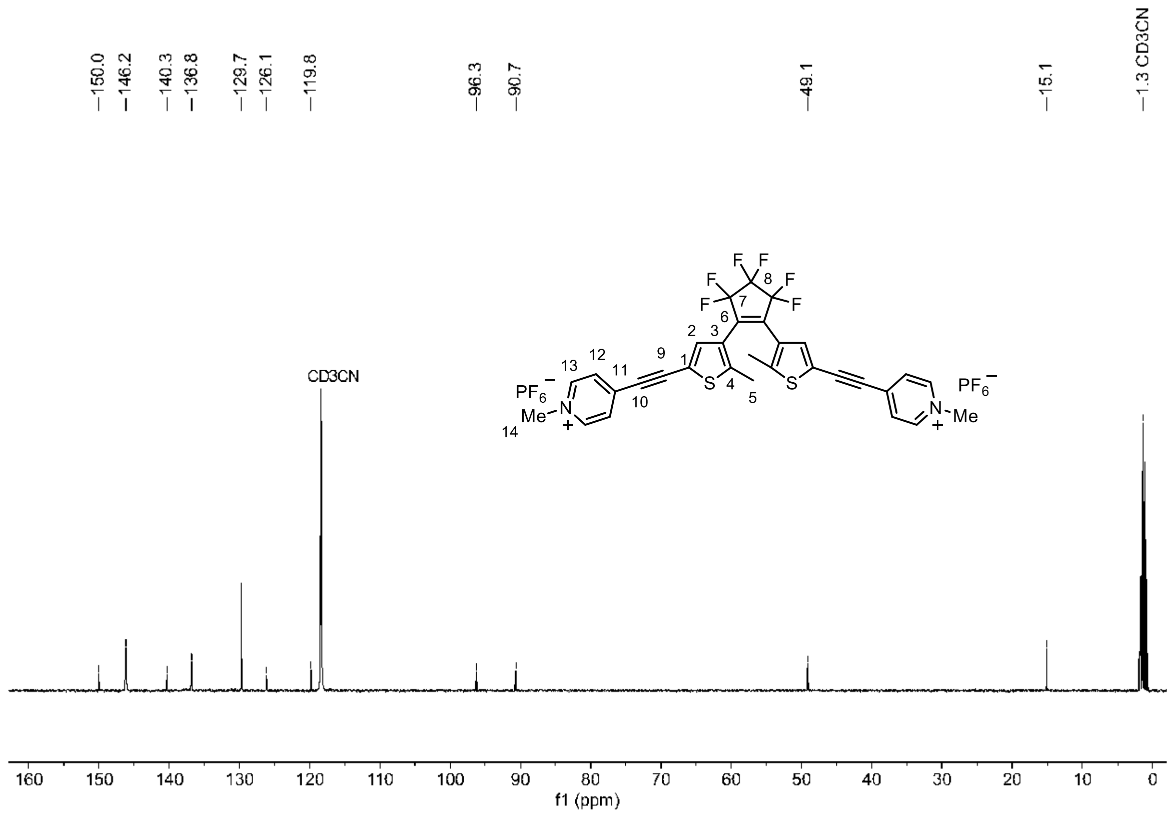

Figure S94 ^{13}C NMR spectrum (101 MHz, CD3CN) of **Ay-yA**.

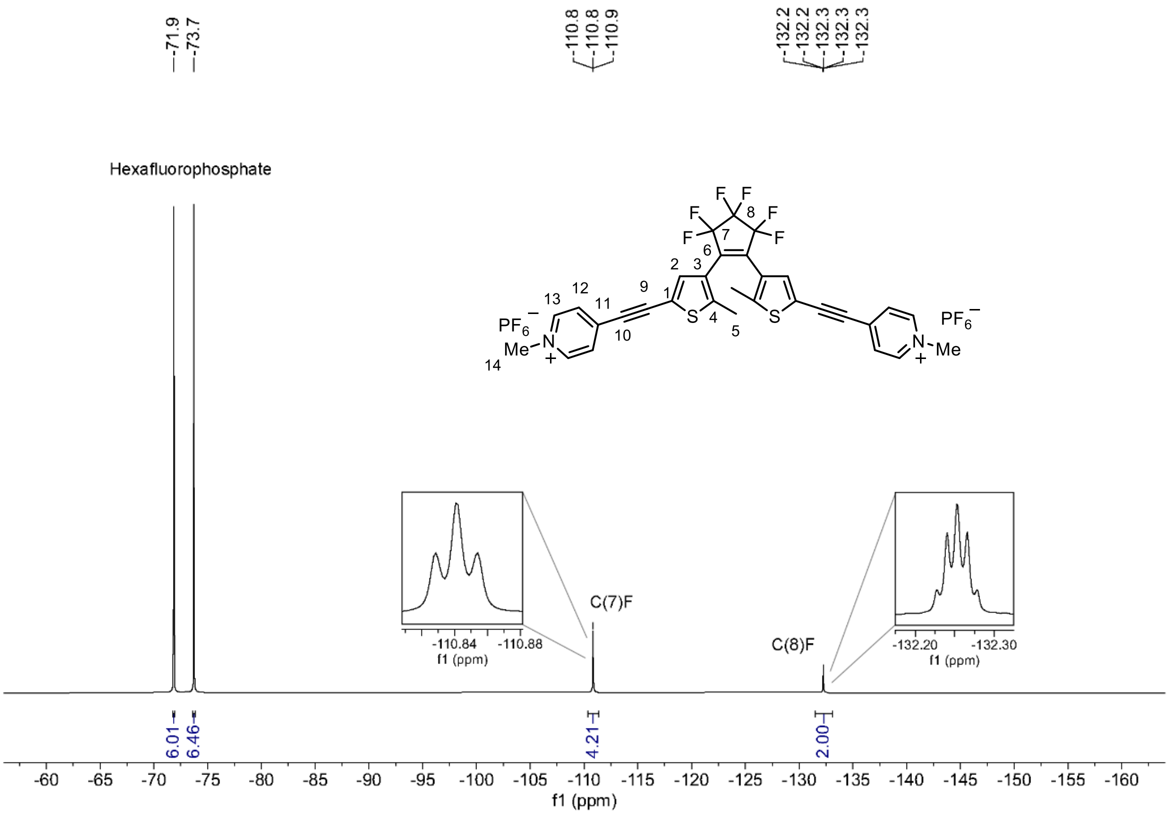

Figure S95 ^{19}F NMR spectrum (376 MHz, CD_3CN) of **Ay-yA**.

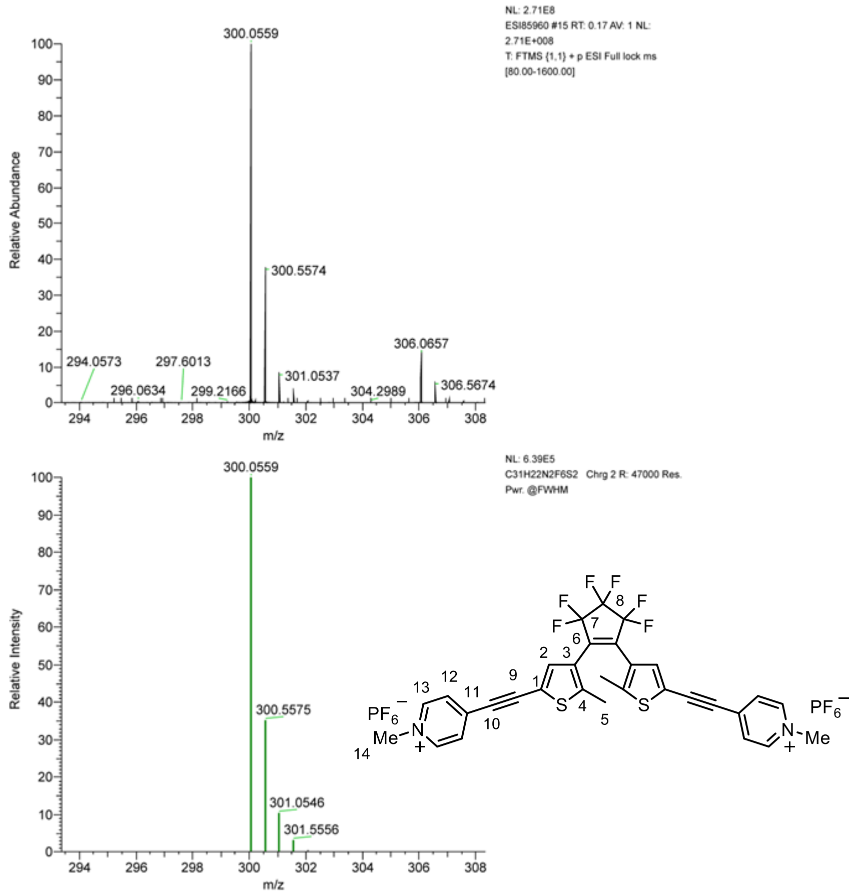

Figure S96 HRMS (ESI+) of **Ay-yA**, *m/z*: $[M]^{2+}$ calcd. for $C_{31}H_{22}F_6N_2S_2^{2+}$ 300.0559; found 300.0559.

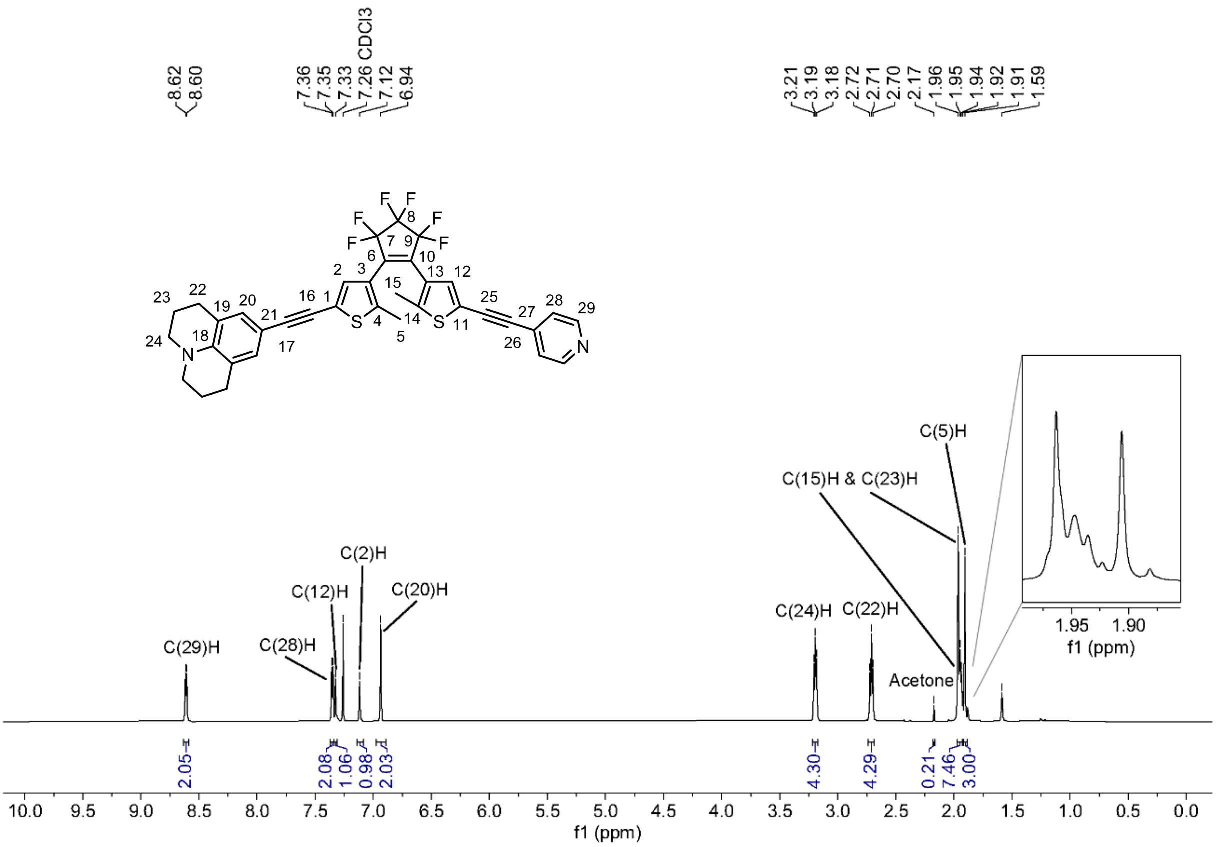

Figure S97 ^{1}H NMR spectrum (400 MHz, $CDCl_3$) of **Dy-yA(p)**.

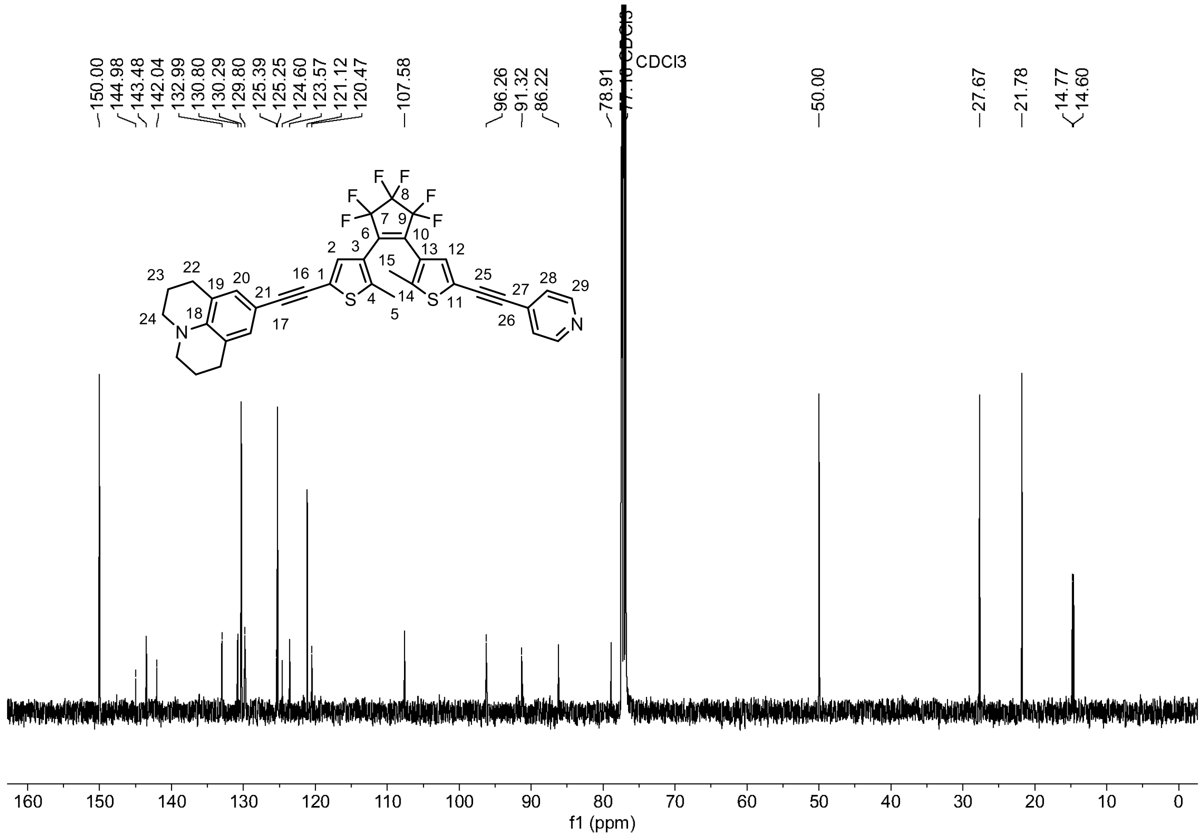

Figure S98 ^{13}C NMR spectrum (101 MHz, $CDCl_3$) of **Dy-yA(p)**.

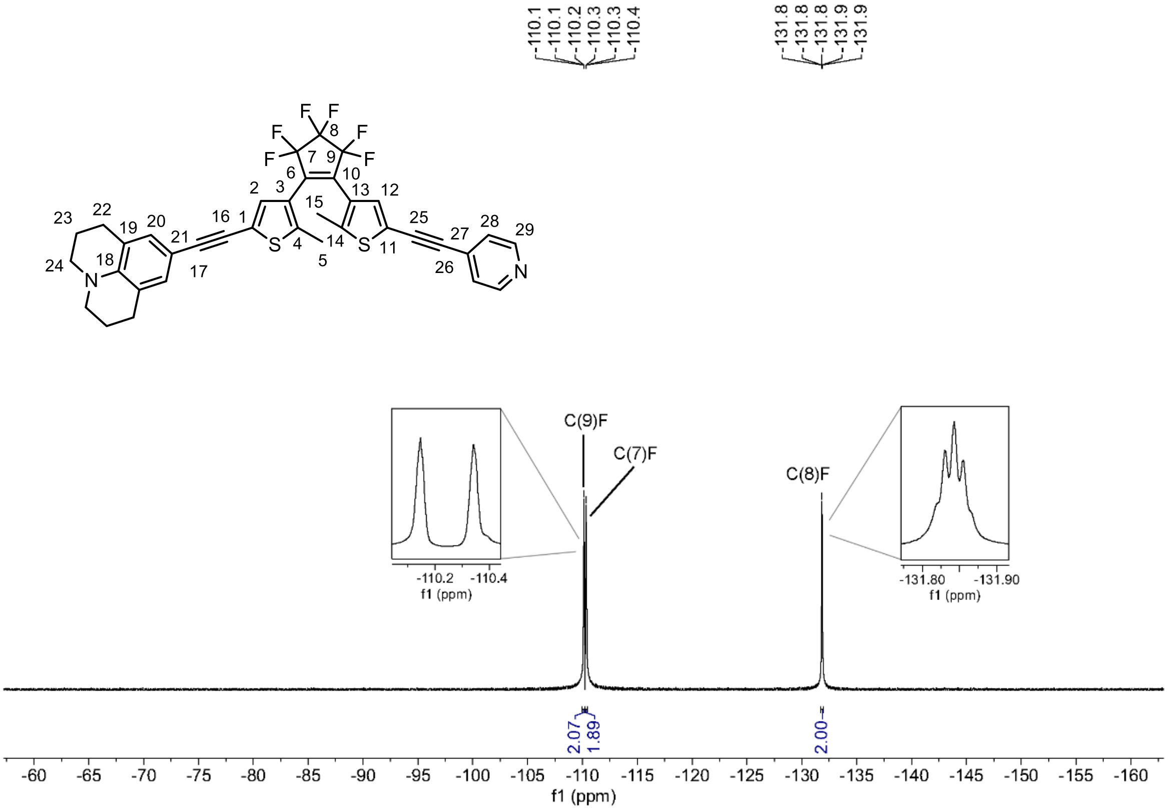

Figure S99 ^{19}F NMR spectrum (376 MHz, $CDCl_3$) of **Dy-yA(p)**.

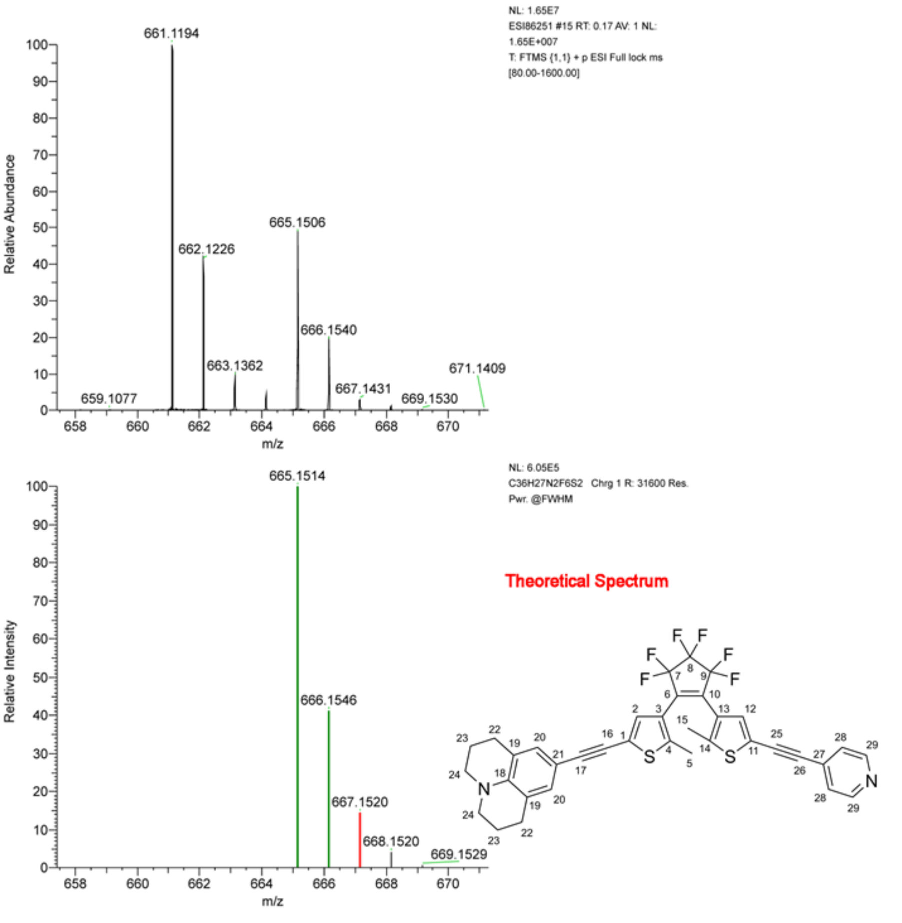

Figure S100 HRMS (ESI$^+$) of Dy-yA(p) *m/z*: $[M+H]^+$ calcd. for $C_{36}H_{27}F_6N_2S_2^+$ 665.1514; found 665.1506.

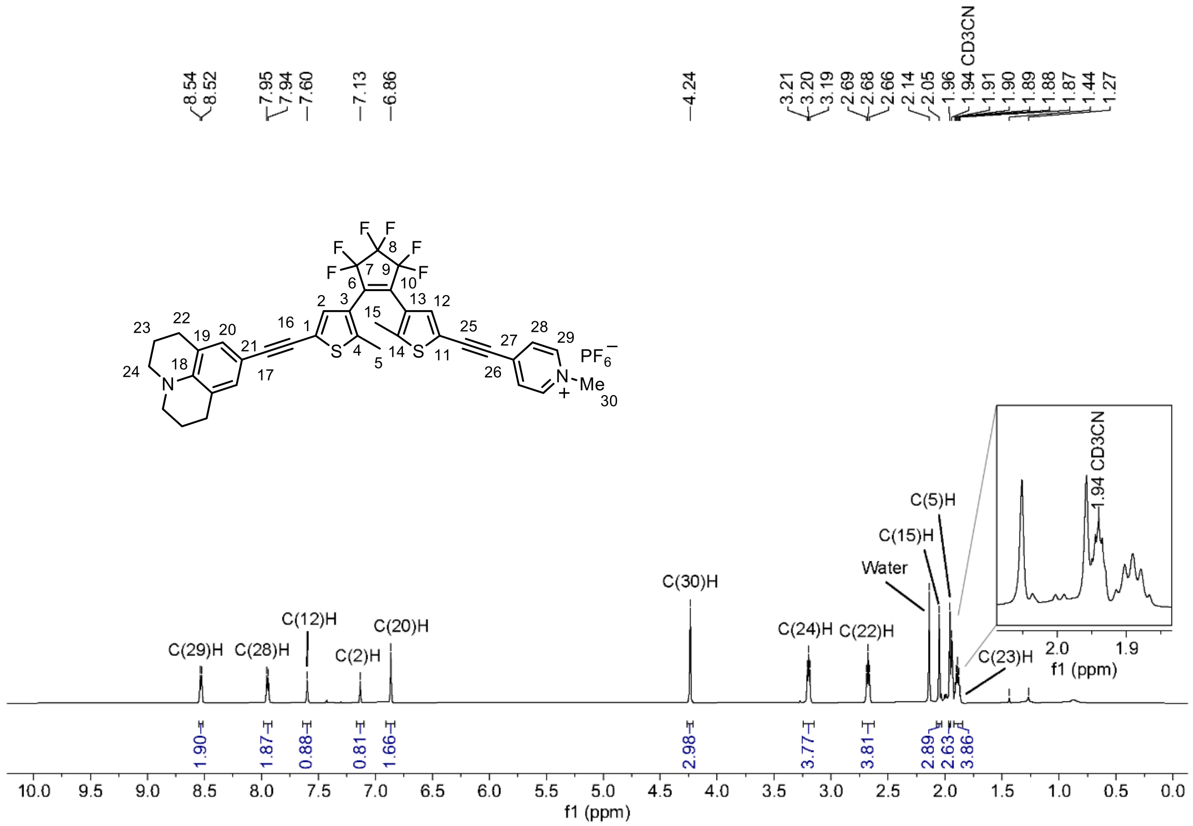

Figure S101 ^{1}H NMR spectrum (400 MHz, CD_3CN) of **Dy-yA**.

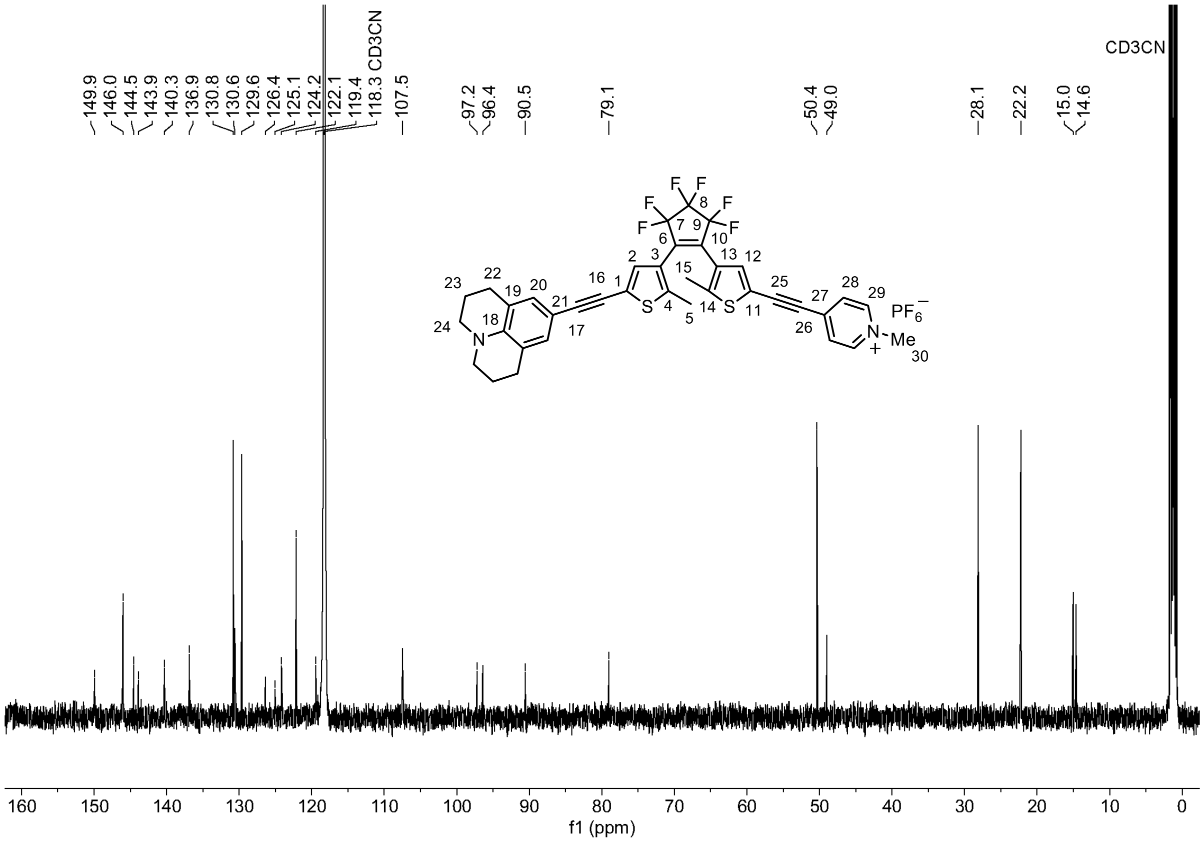

Figure S102 ^{13}C NMR spectrum (101 MHz, CD_3CN) of **Dy-yA**.

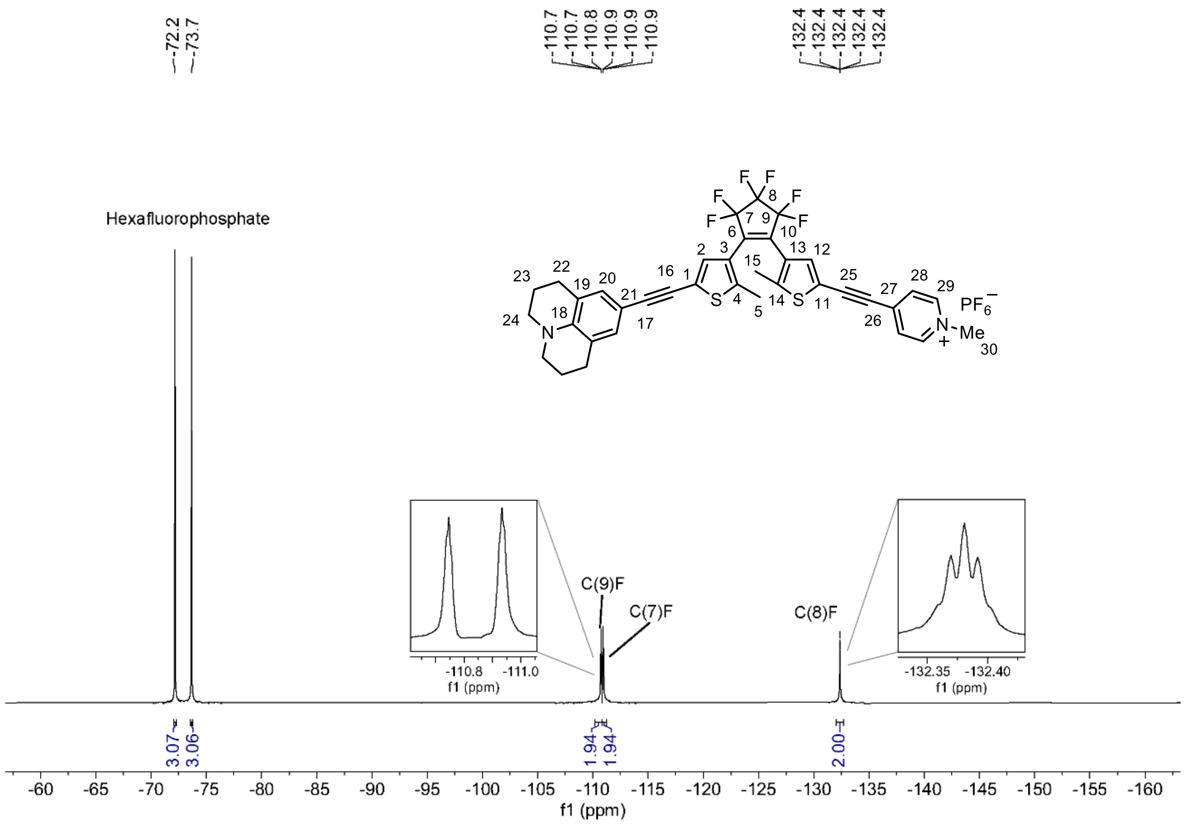

Figure S103 ^{19}F NMR spectrum (376 MHz, CD_3CN) of **Dy-yA**.

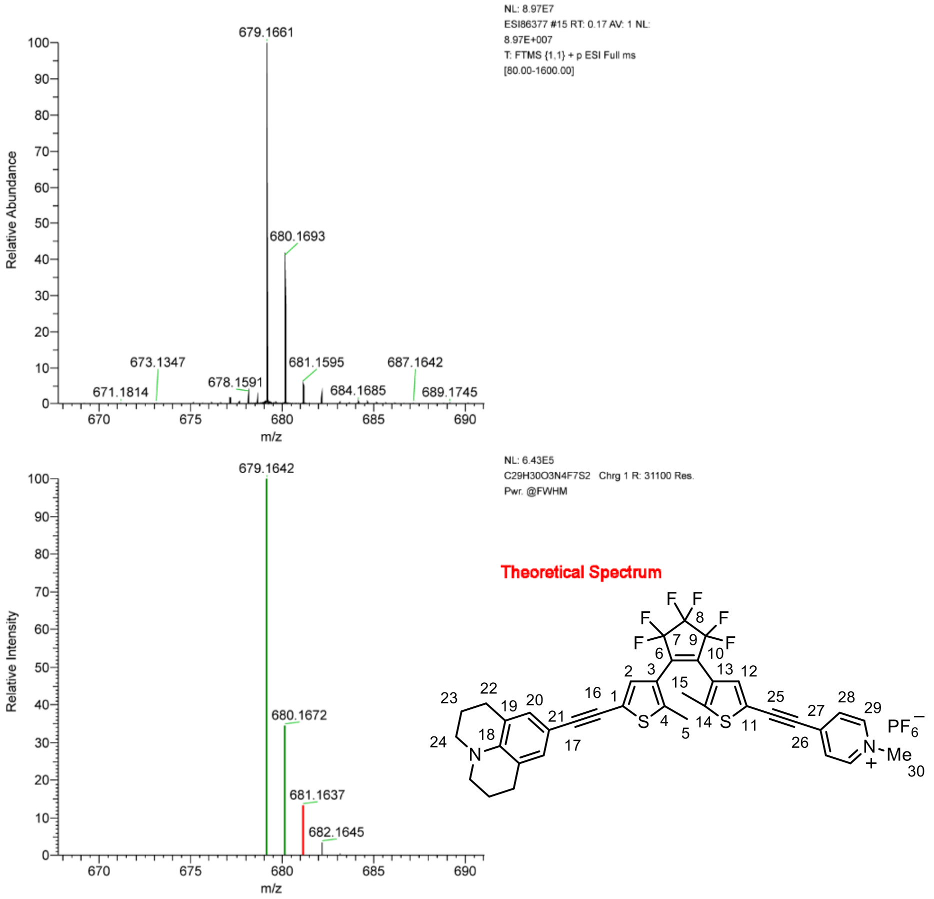

Figure S104 HRMS (ESI[+]) of **Dy-yA**, *m/z*: [M[+]] calcd. for $C_{37}H_{29}F_6N_2S_2^+$ 679.1642; found 679.1661.

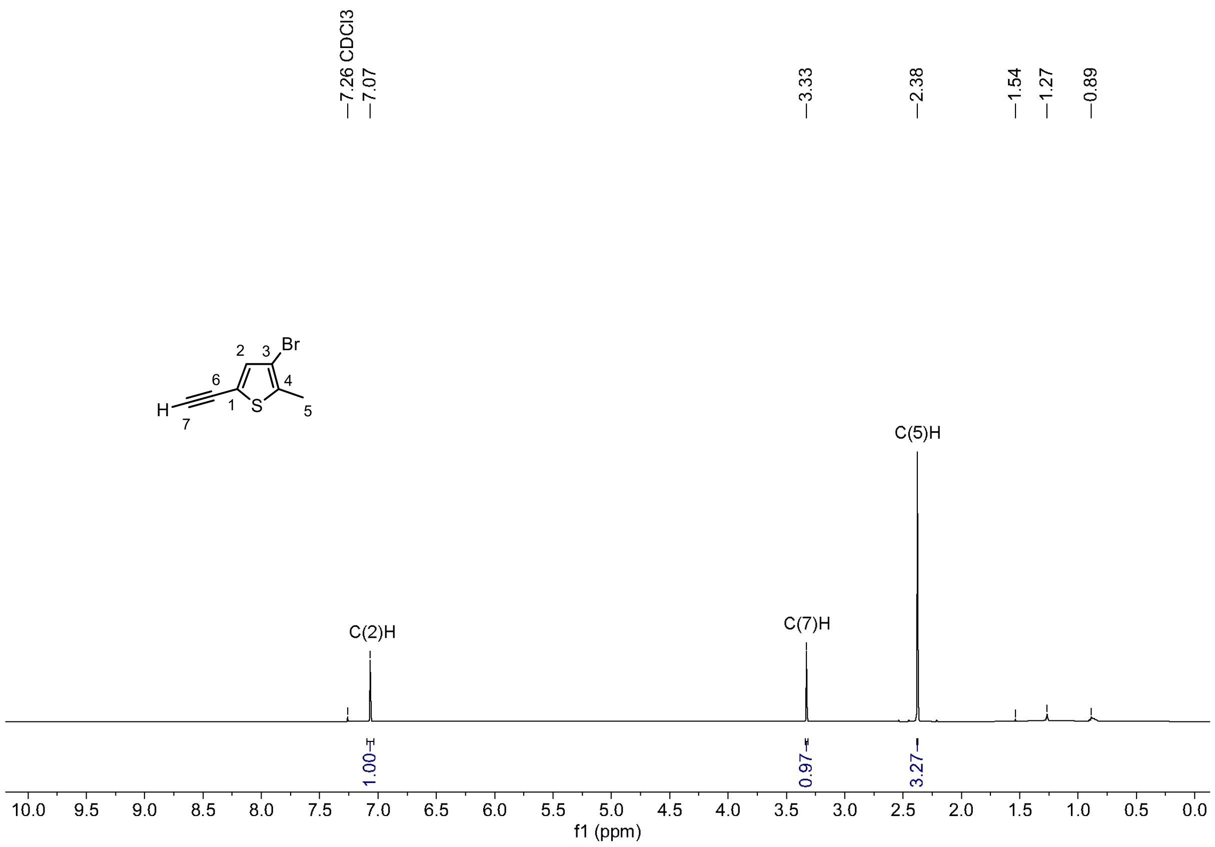

Figure S105 ¹H NMR (400 MHz, $CDCl_3$) of **4**.

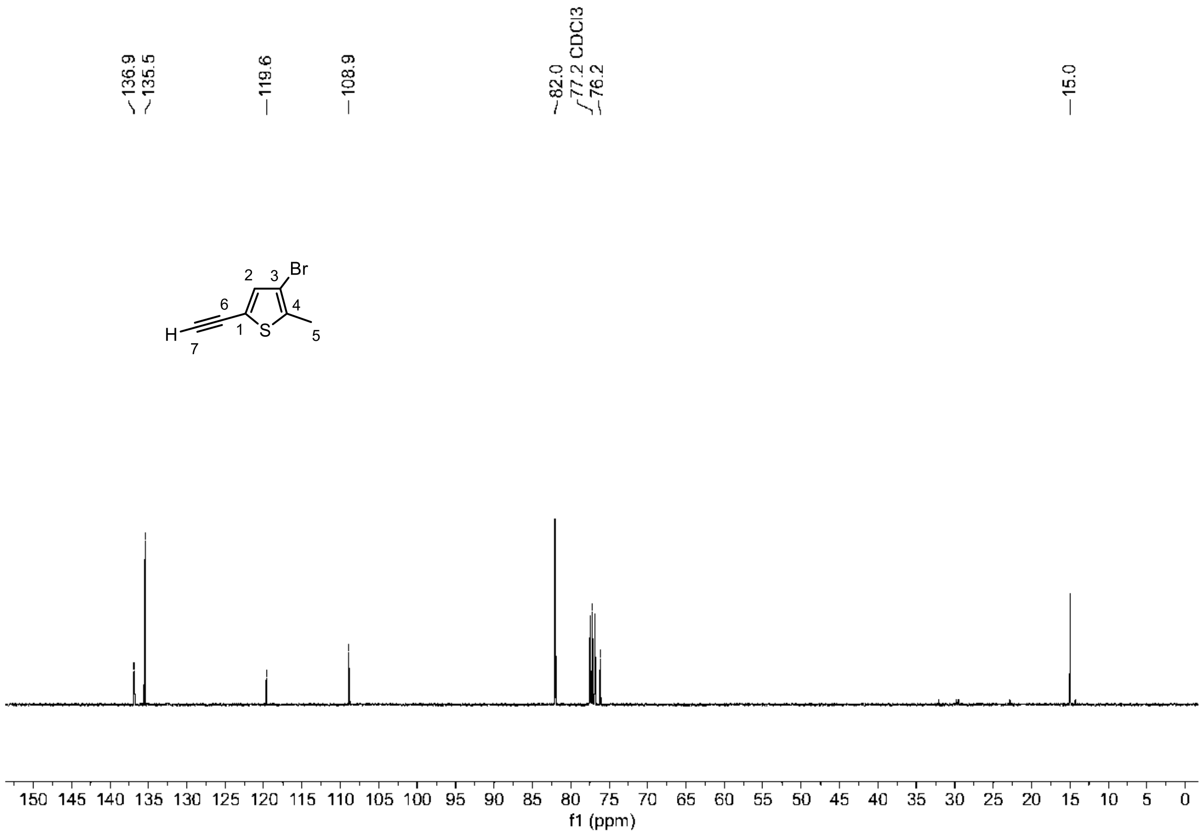

Figure S106 ¹³C NMR spectrum (101 MHz, $CDCl_3$) of **4**.

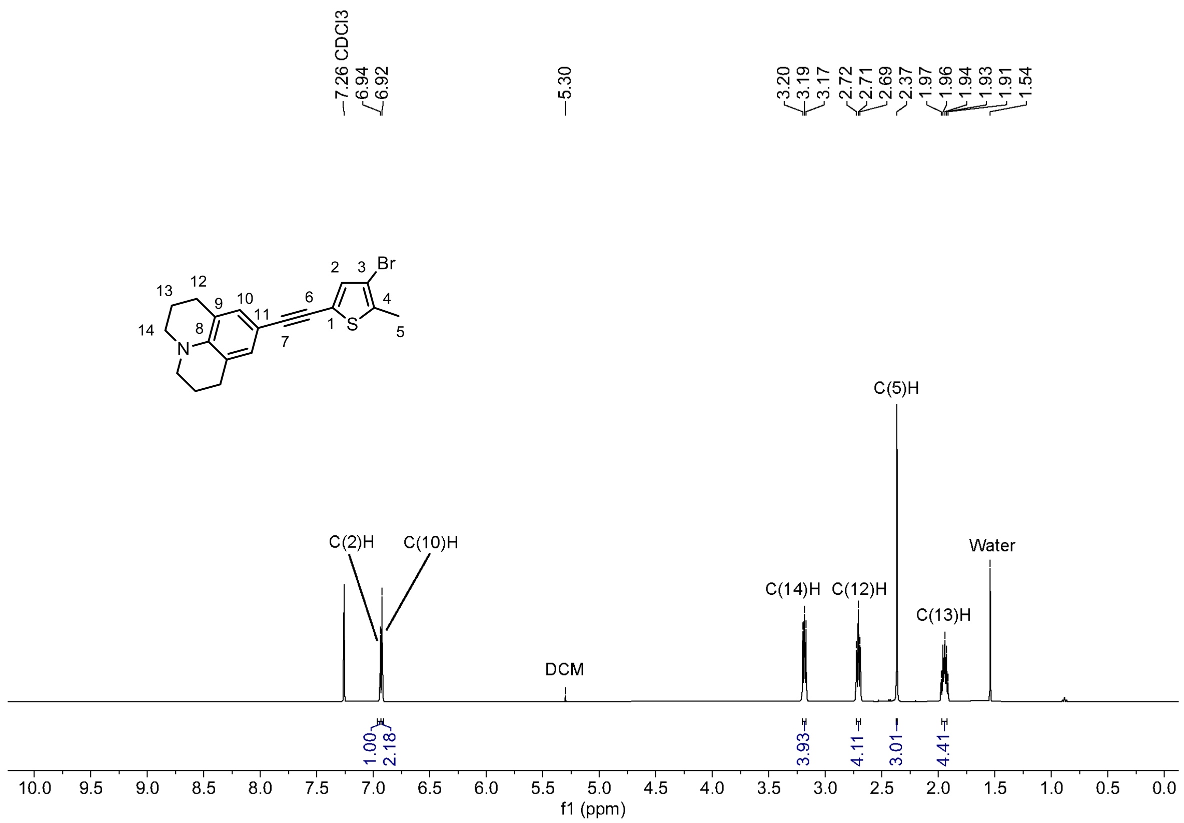

Figure S107 ^{1}H NMR spectrum (400 MHz, $CDCl_3$) of **6**.

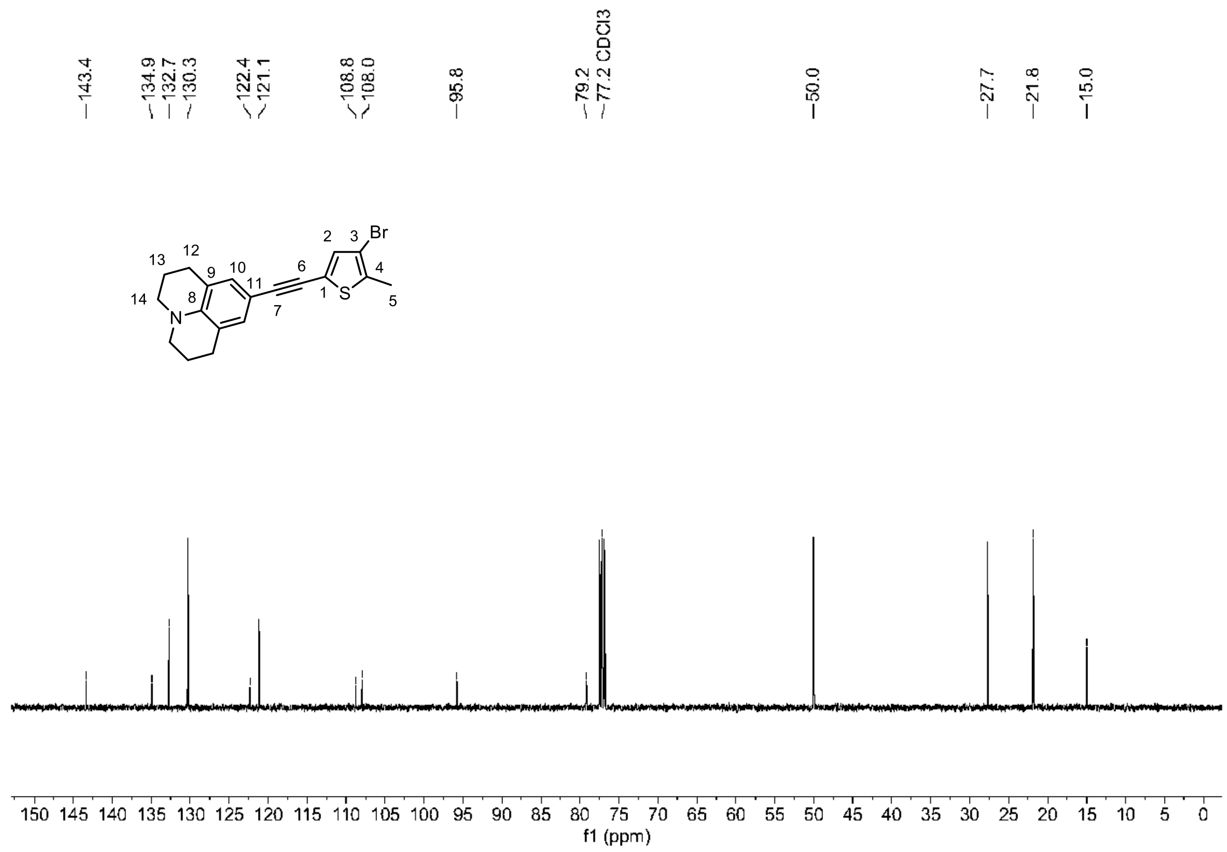

Figure S108 ^{13}C NMR spectrum (101 MHz, $CDCl_3$) of **6**.

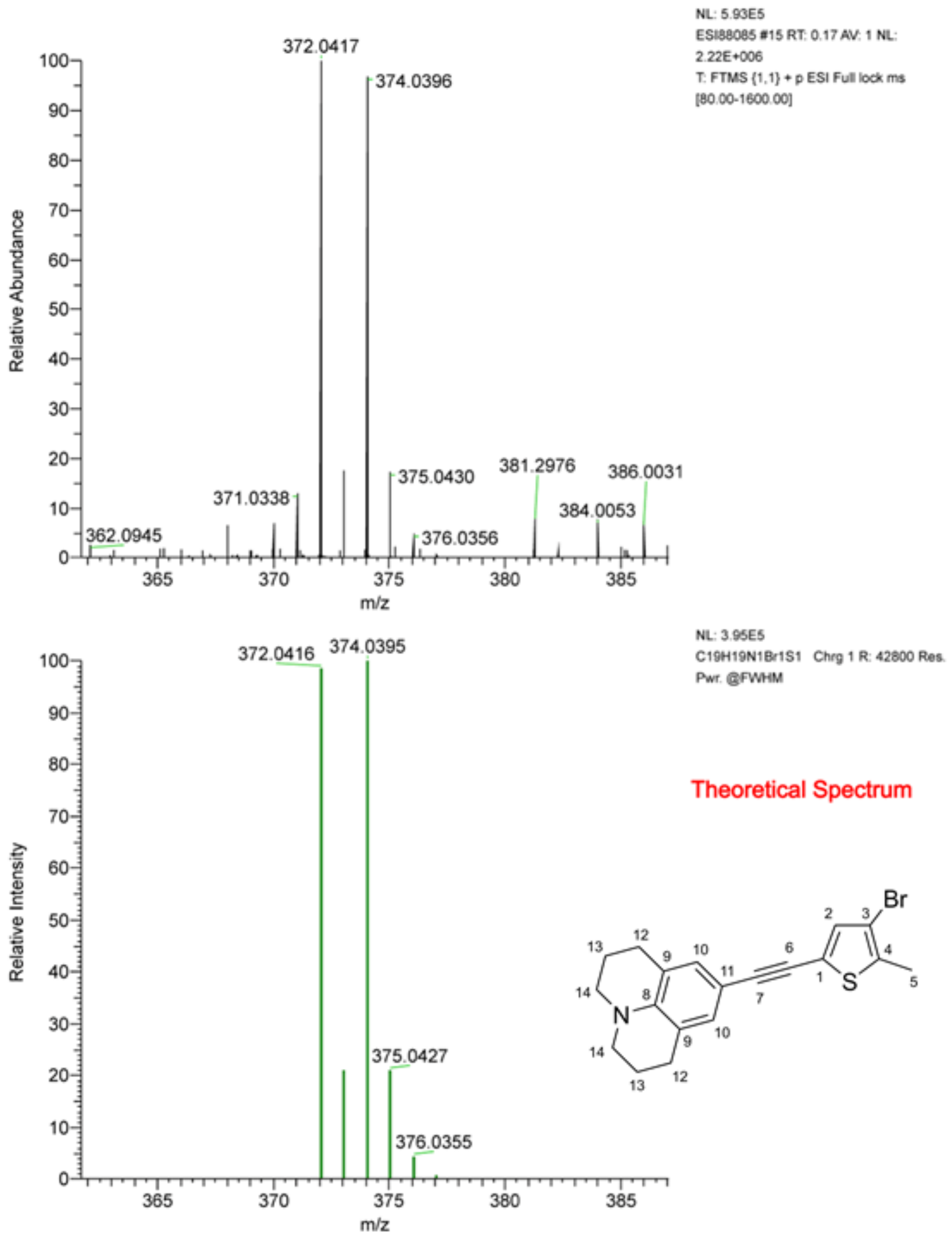

Figure S109 HRMS (ESI+) of **9**, *m/z*: [M]+ calcd. for $C_{19}H_{19}NBrS$ 374.0395; found 374.0396.

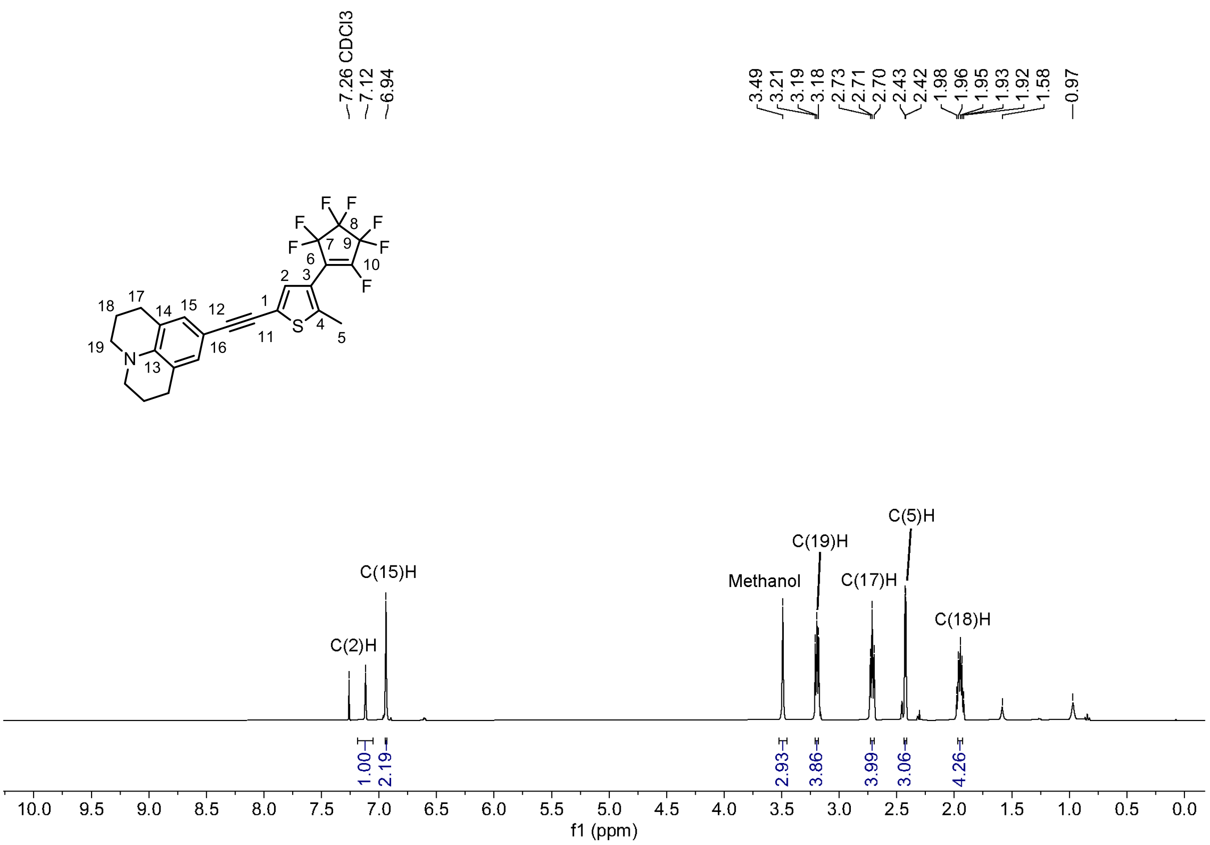

Figure S110 ^{1}H NMR spectrum (400 MHz, $CDCl_3$) of **8**.

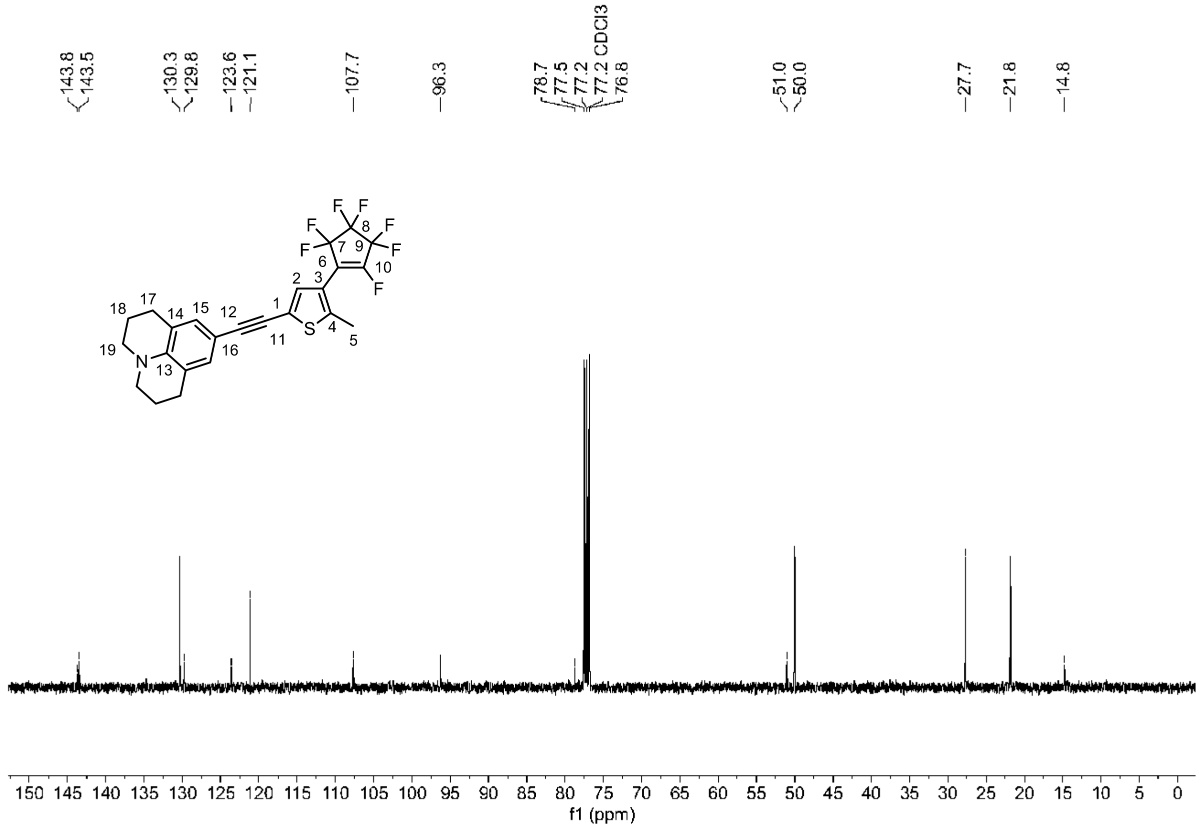

Figure S111 ^{13}C NMR spectrum (101 MHz, $CDCl_3$) of **8**.

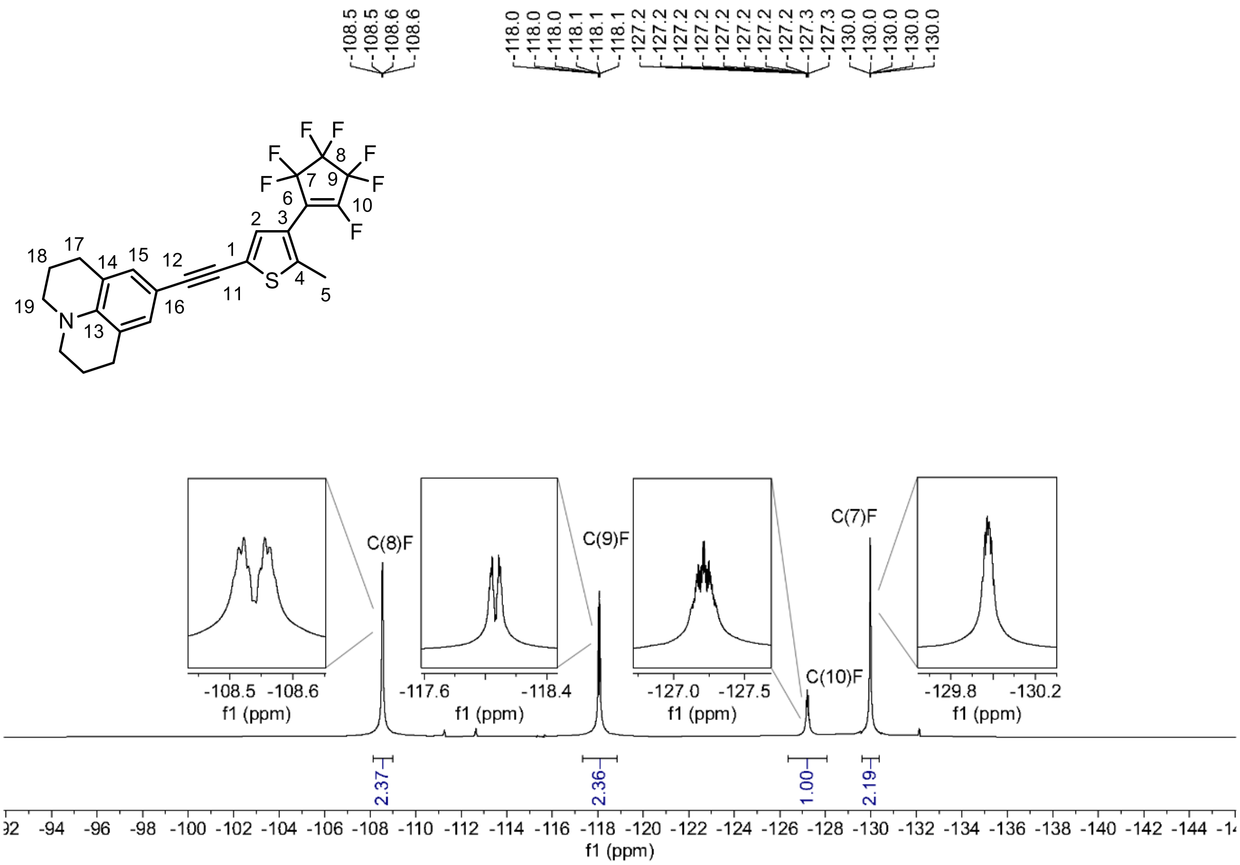

Figure S112 ^{19}F NMR spectrum (376 MHz, $CDCl_3$) of **8**.

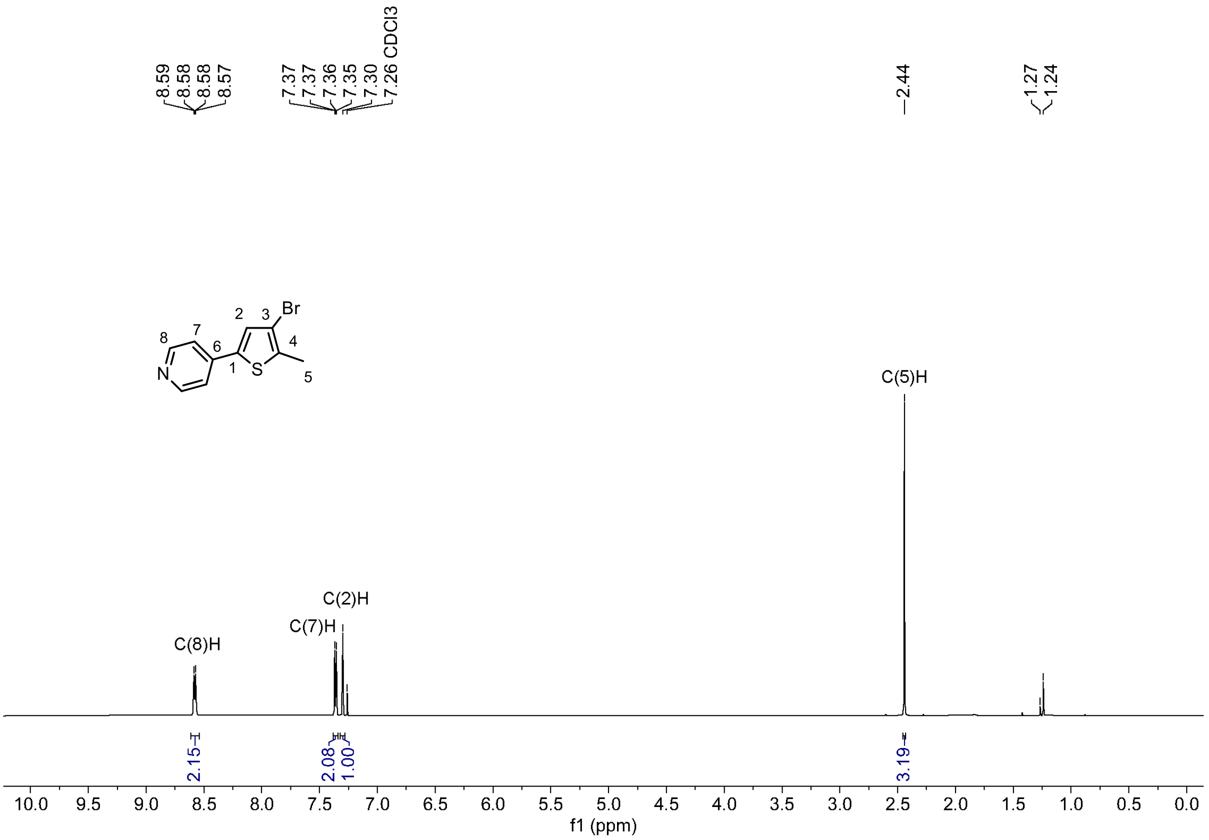

Figure S113 ^{1}H NMR spectrum (400 MHz, $CDCl_3$) of **9**.

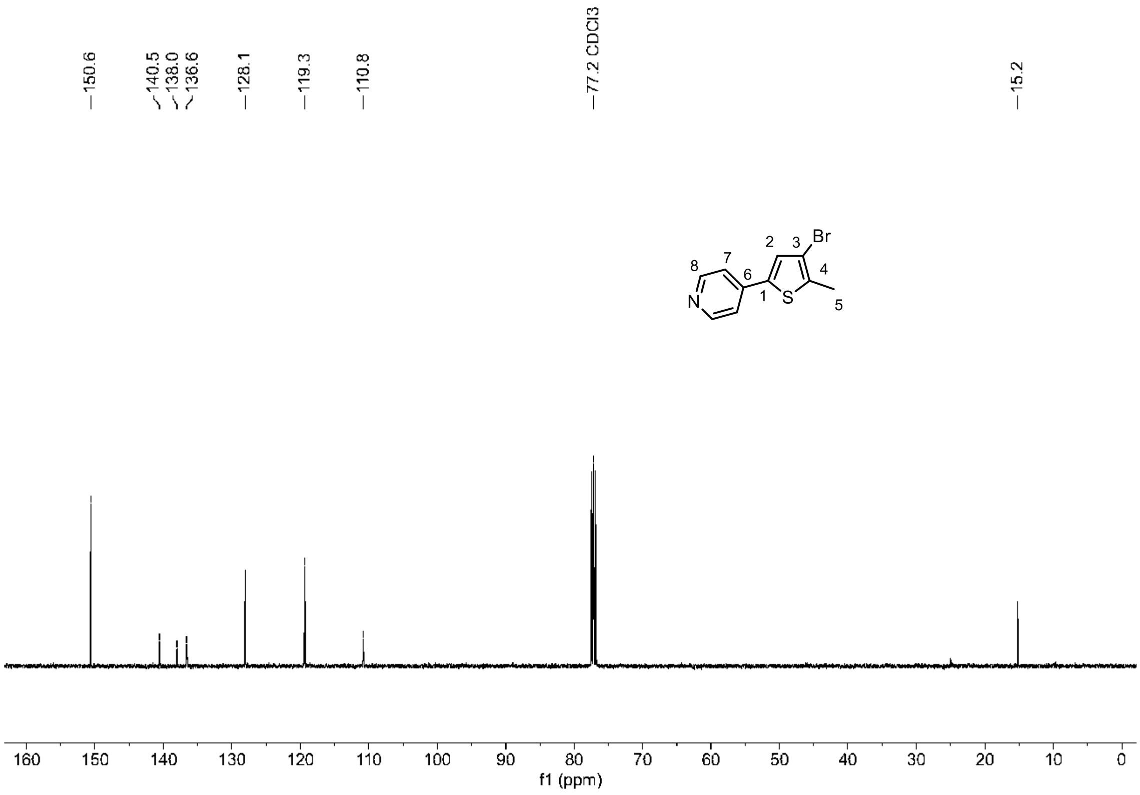

Figure S114 ^{13}C NMR spectrum (101 MHz, $CDCl_3$) of **9**.

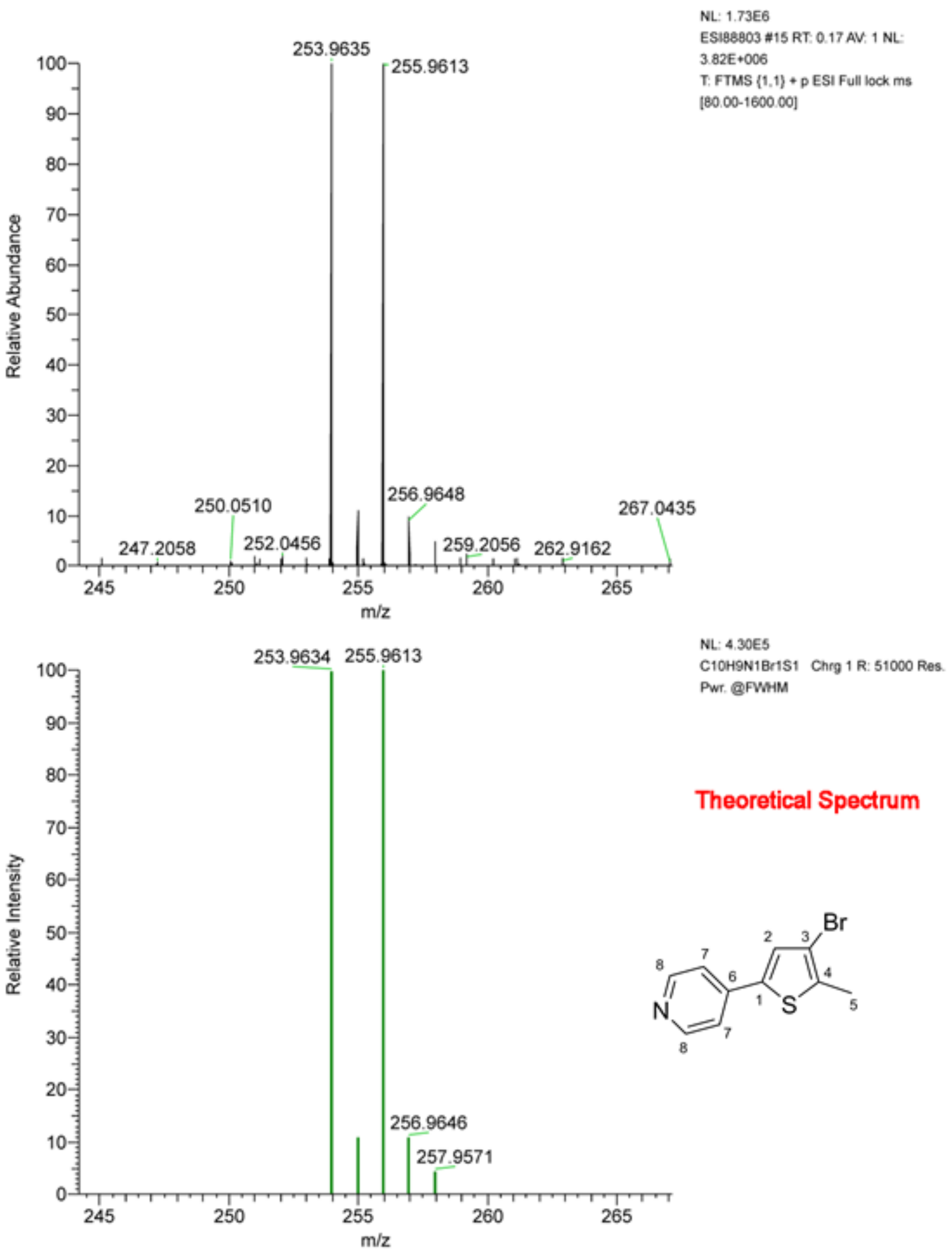

Figure S115 HRMS (ESI$^+$) of **9** *m/z*: $[M+H]^+$ calcd. for $C_{10}H_9NBrS$ 253.9634; found 253.9635.

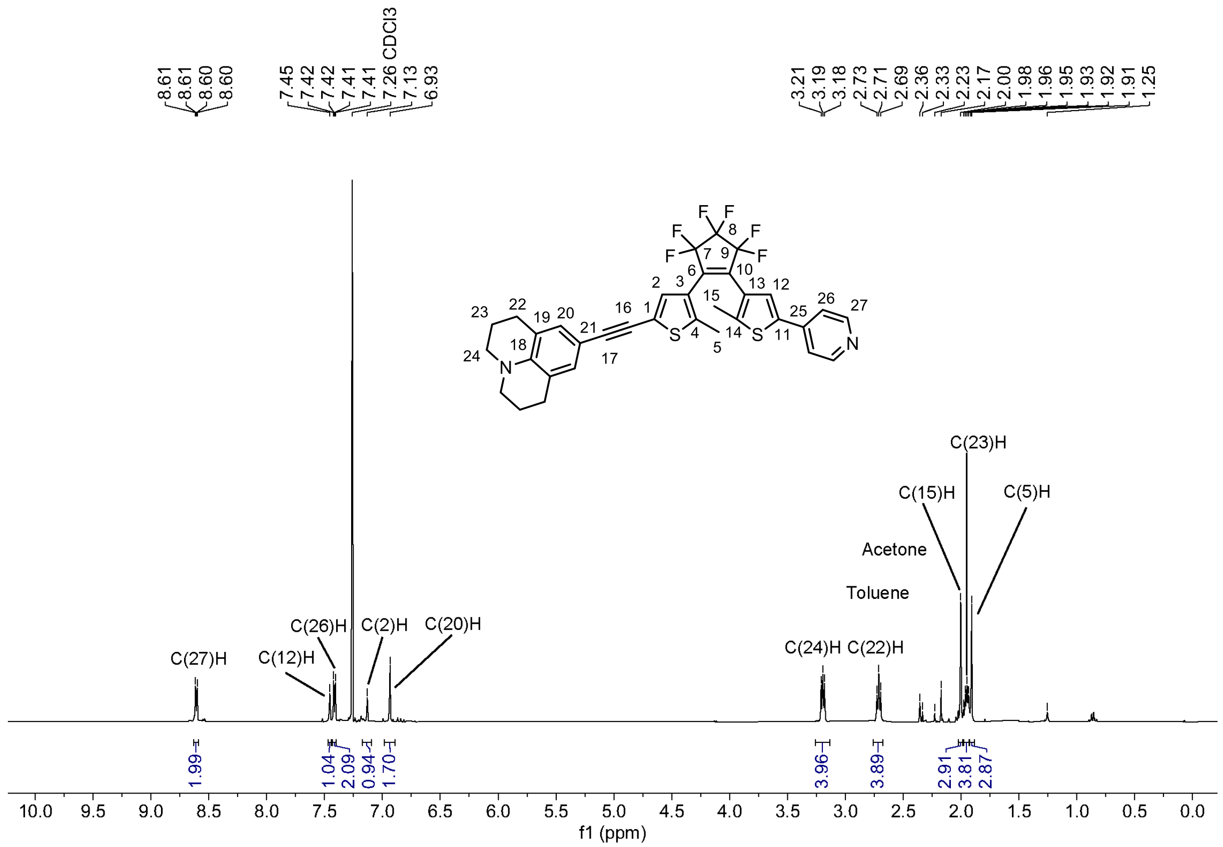

Figure S116 ^{1}H NMR spectrum (400 MHz, $CDCl_3$) of **Dy-A(p)**.

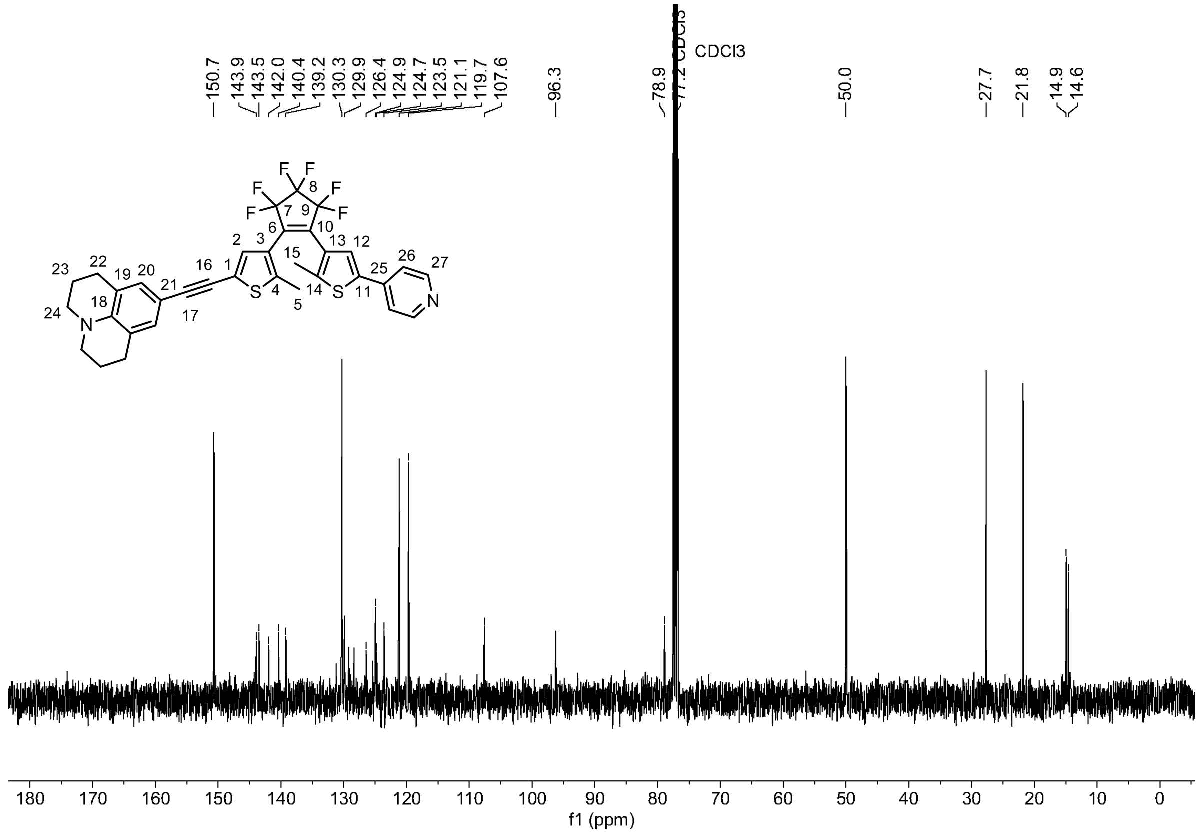

Figure S117 ^{13}C NMR spectrum (101 MHz, $CDCl_3$) of **Dy-A(p)**.

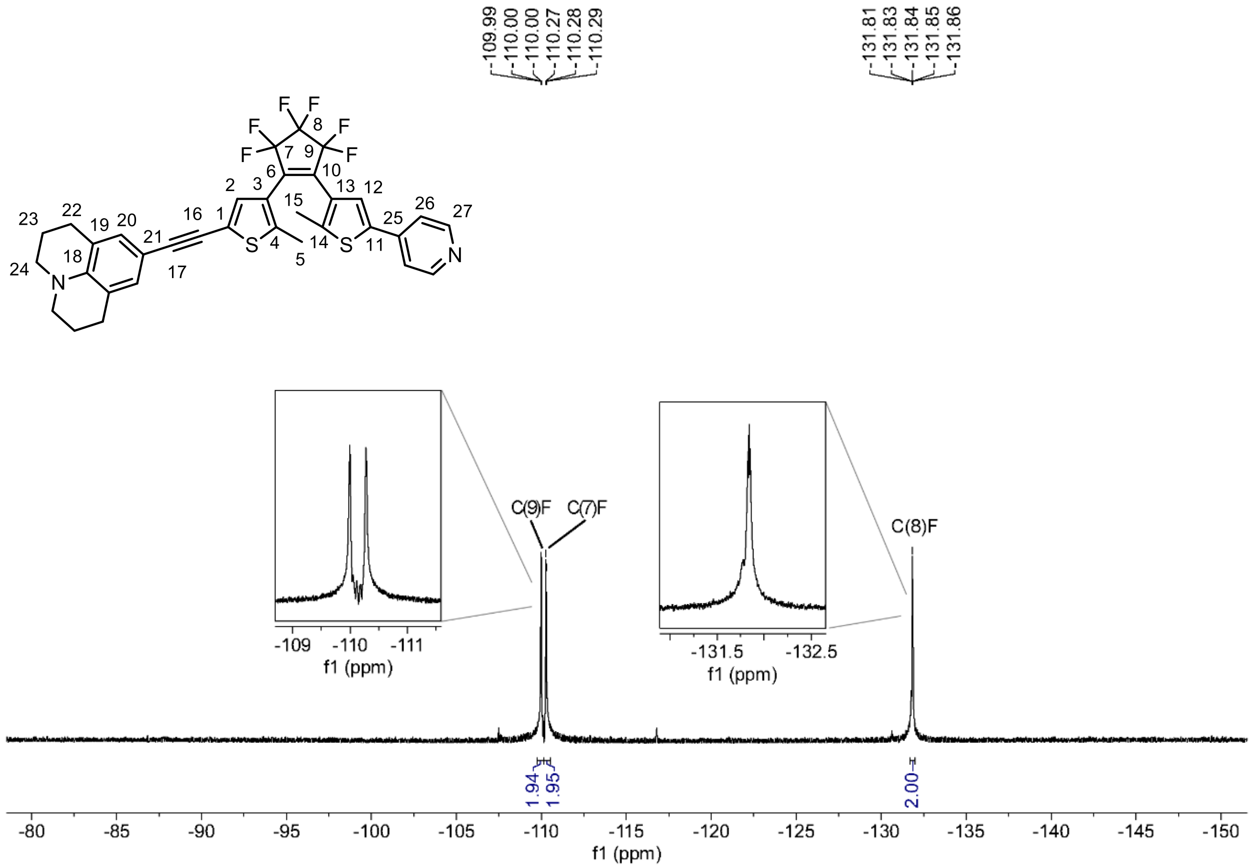

Figure S118 ^{19}F NMR spectrum (376 MHz, $CDCl_3$) of **Dy-A(p)**.

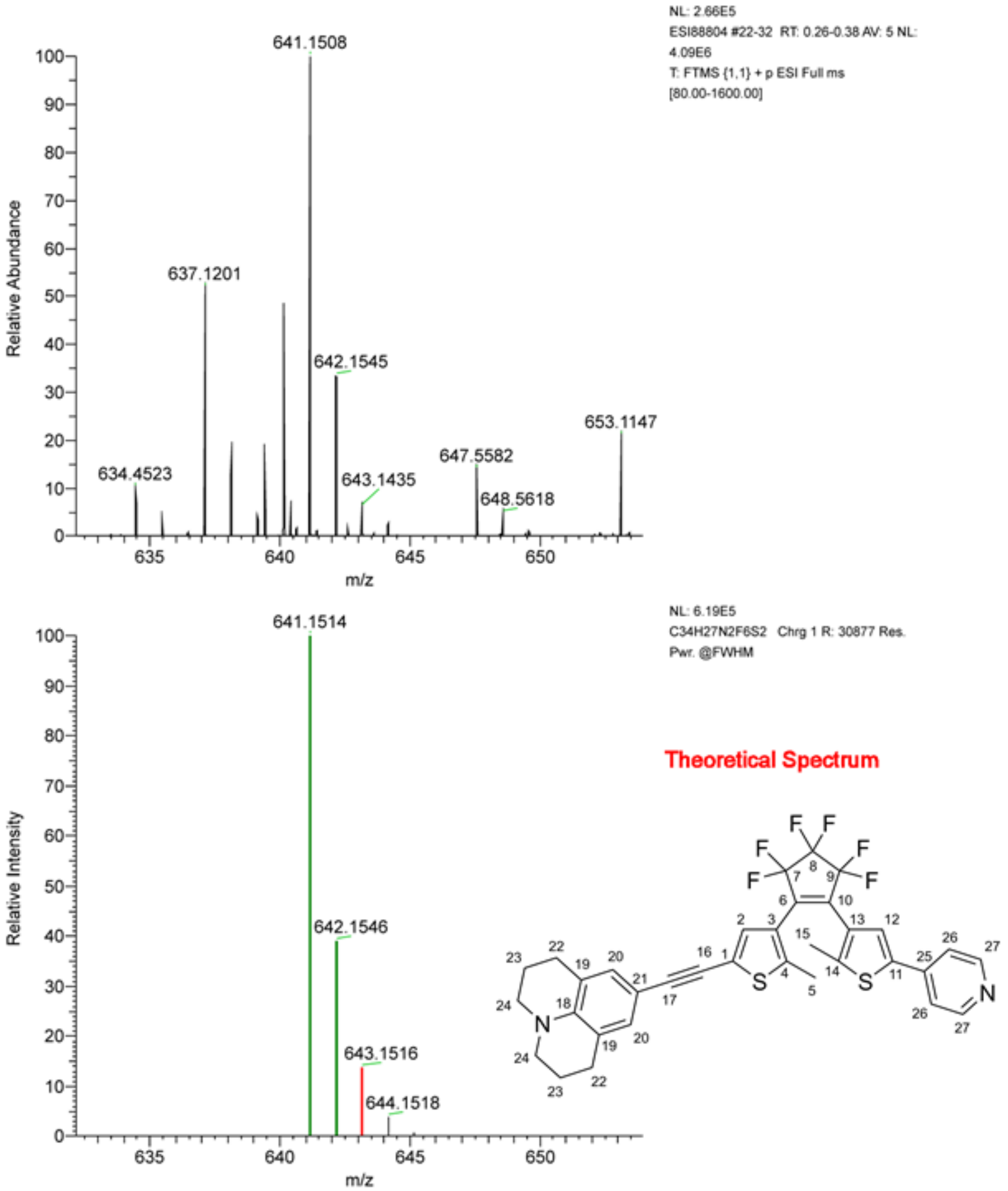

Figure S119 HRMS (ESI^{+}) of **Dy-A(p)** *m/z*: $[M+H]^{+}$ calcd. for $C_{34}H_{27}F_6N_2S_2^{+}$ 641.1514; found 641.1508.

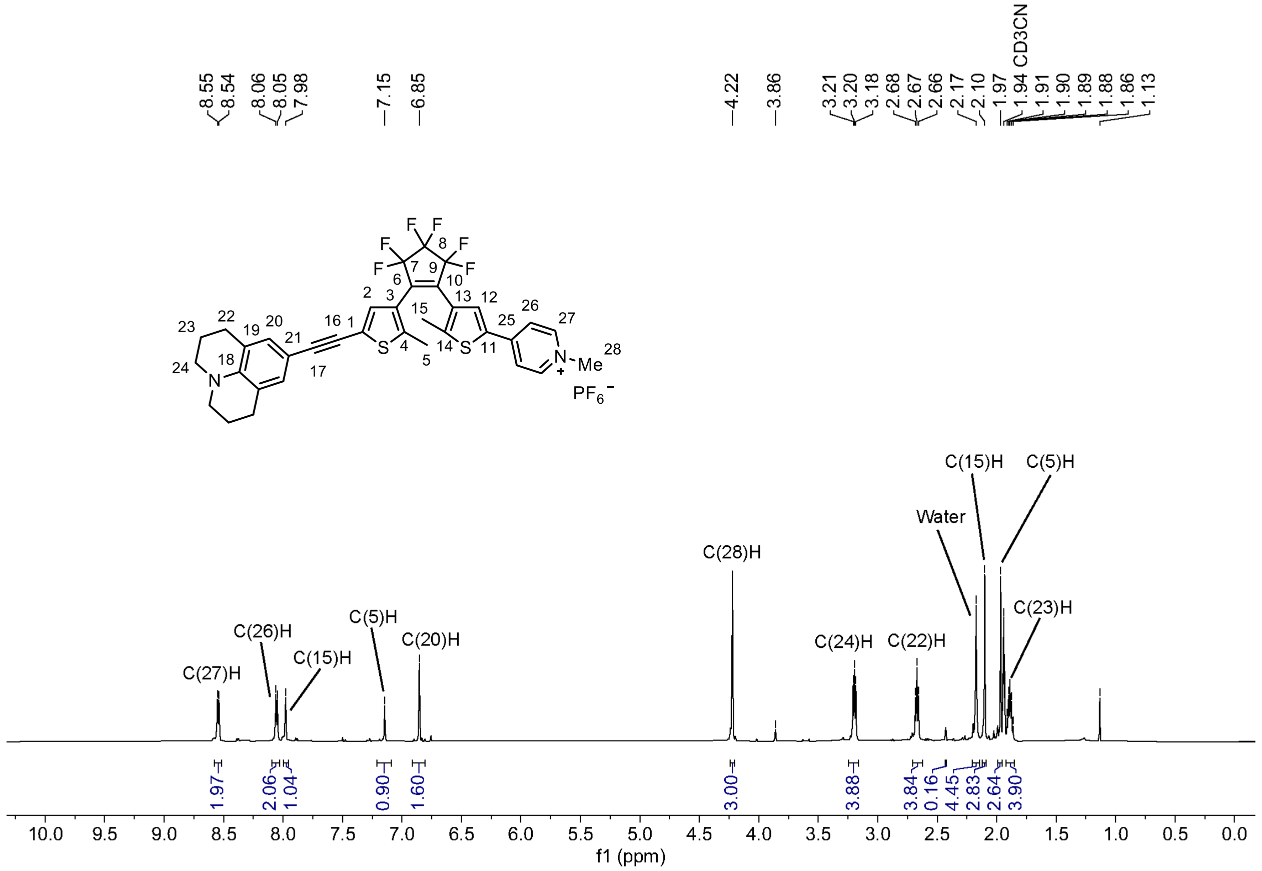

Figure S120 ^{1}H NMR spectrum (400 MHz, CD_3CN) of **Dy-A**.

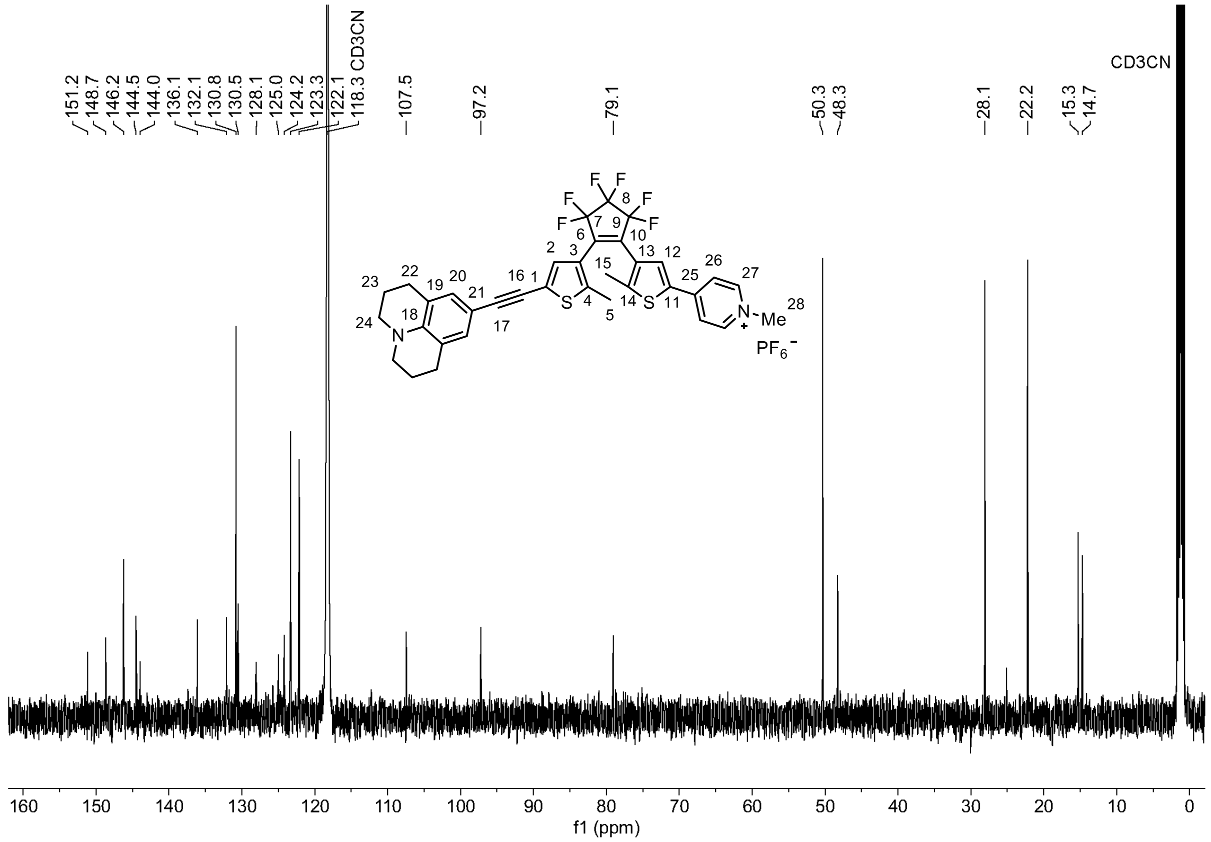

Figure S121 ^{13}C NMR spectrum (101 MHz, CD_3CN) of **Dy-A**.

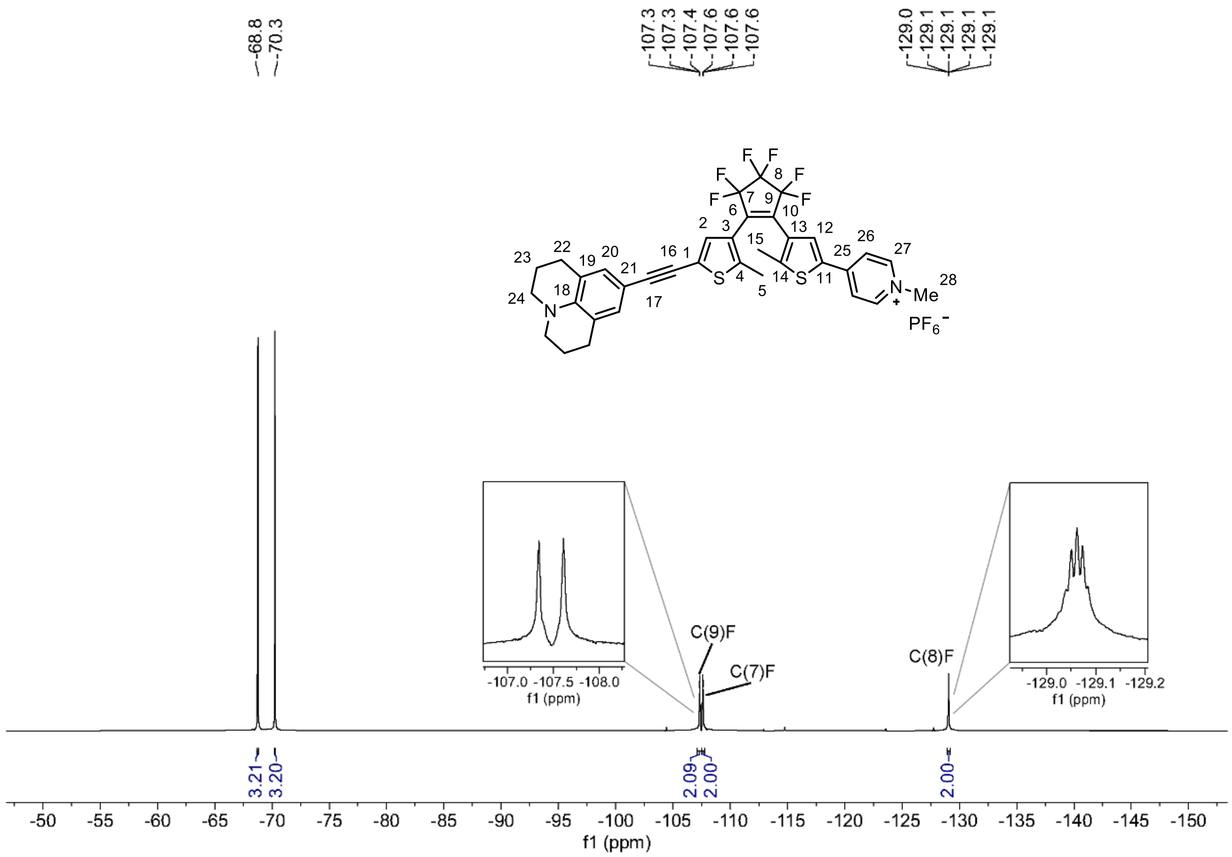

Figure S122 ^{19}F NMR spectrum (376 MHz, $CDCl_3$) of **Dy-A**.

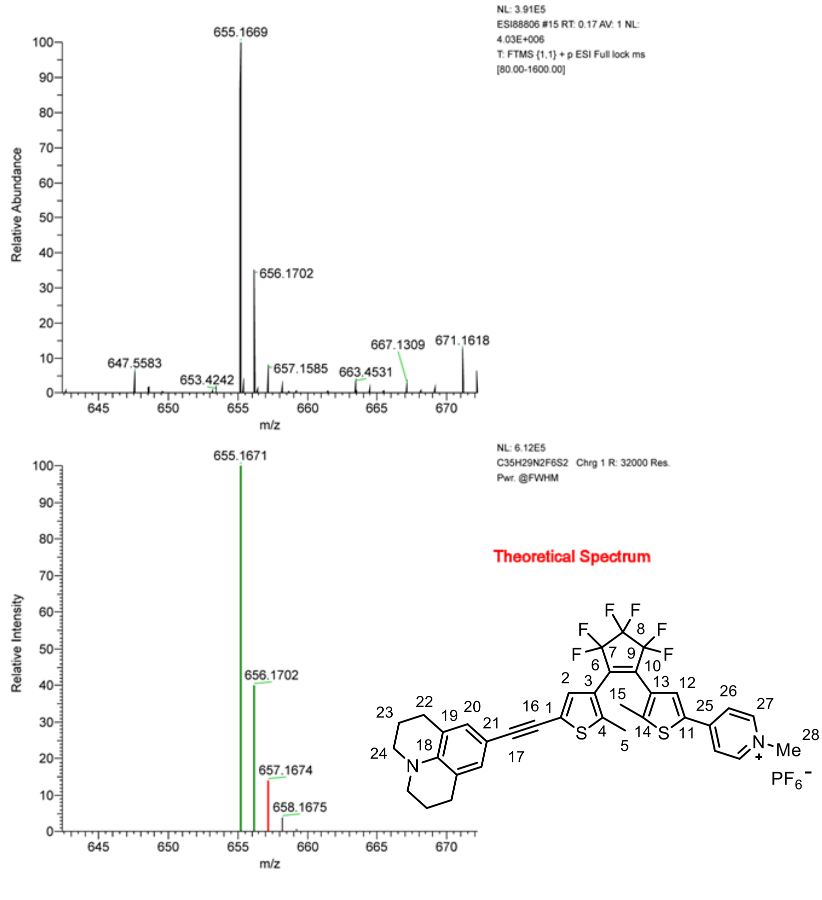

Figure S123 HRMS (ESI$^+$) of **Dy-A**, *m/z*: $[M]^+$ calcd. for $C_{35}H_{29}F_6N_2S_2^+$ 655.1671; found 655.1669.

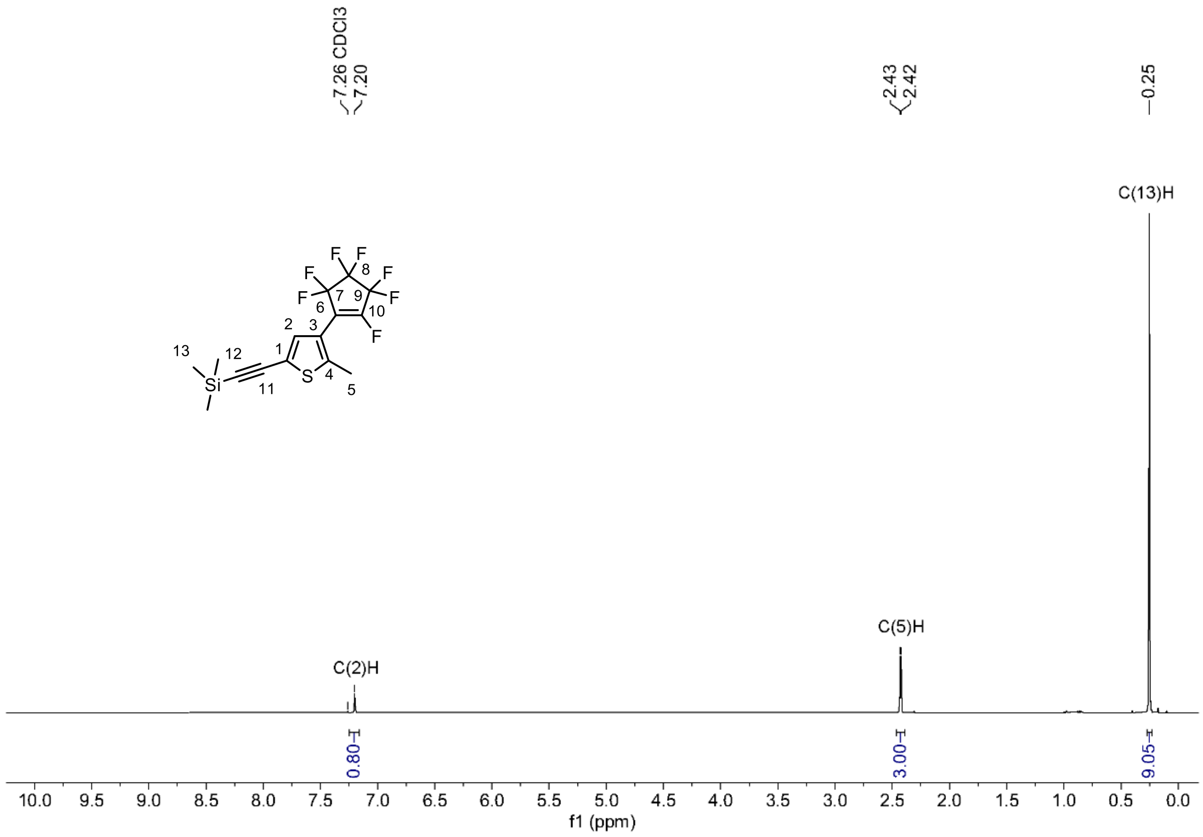

Figure S124 ^{1}H NMR spectrum (400 MHz, $CDCl_3$) of **13**.

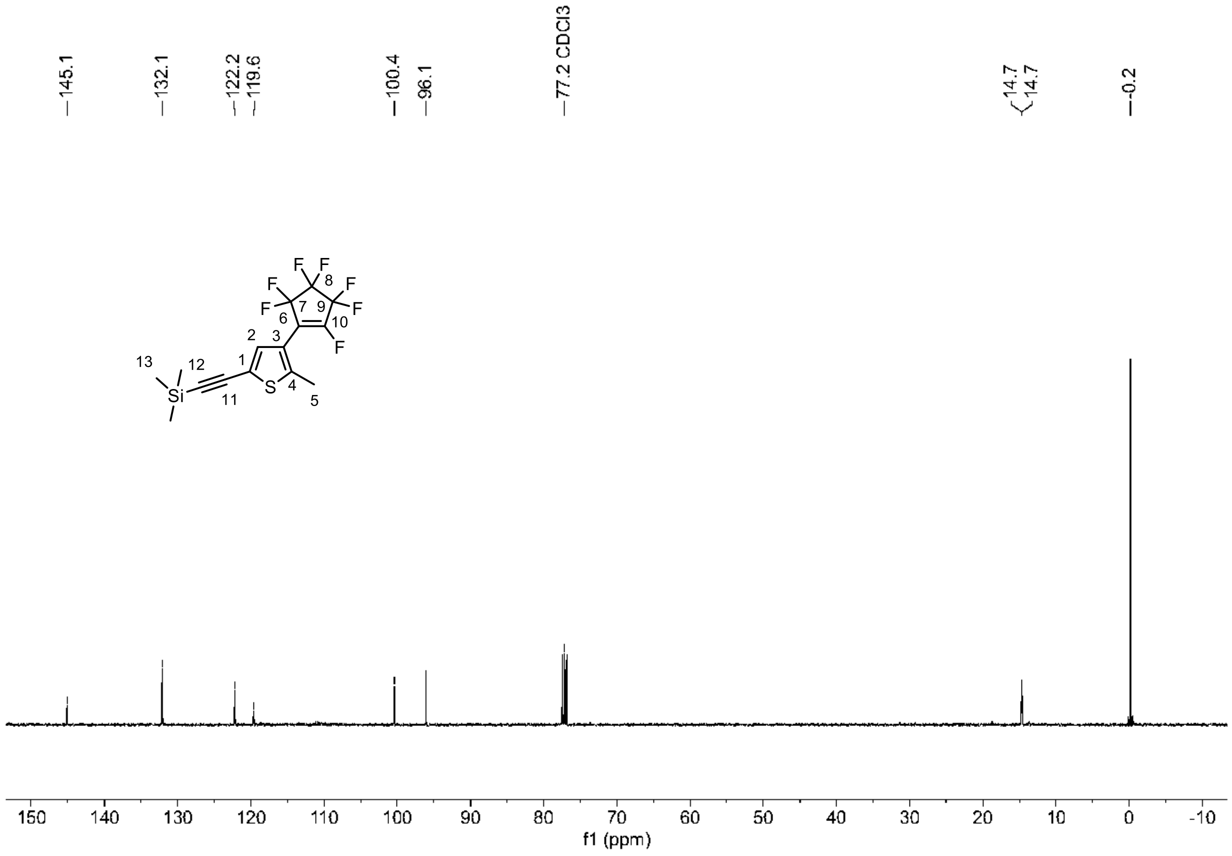

Figure S125 ^{13}C NMR spectrum (101 MHz, $CDCl_3$) of **13**.

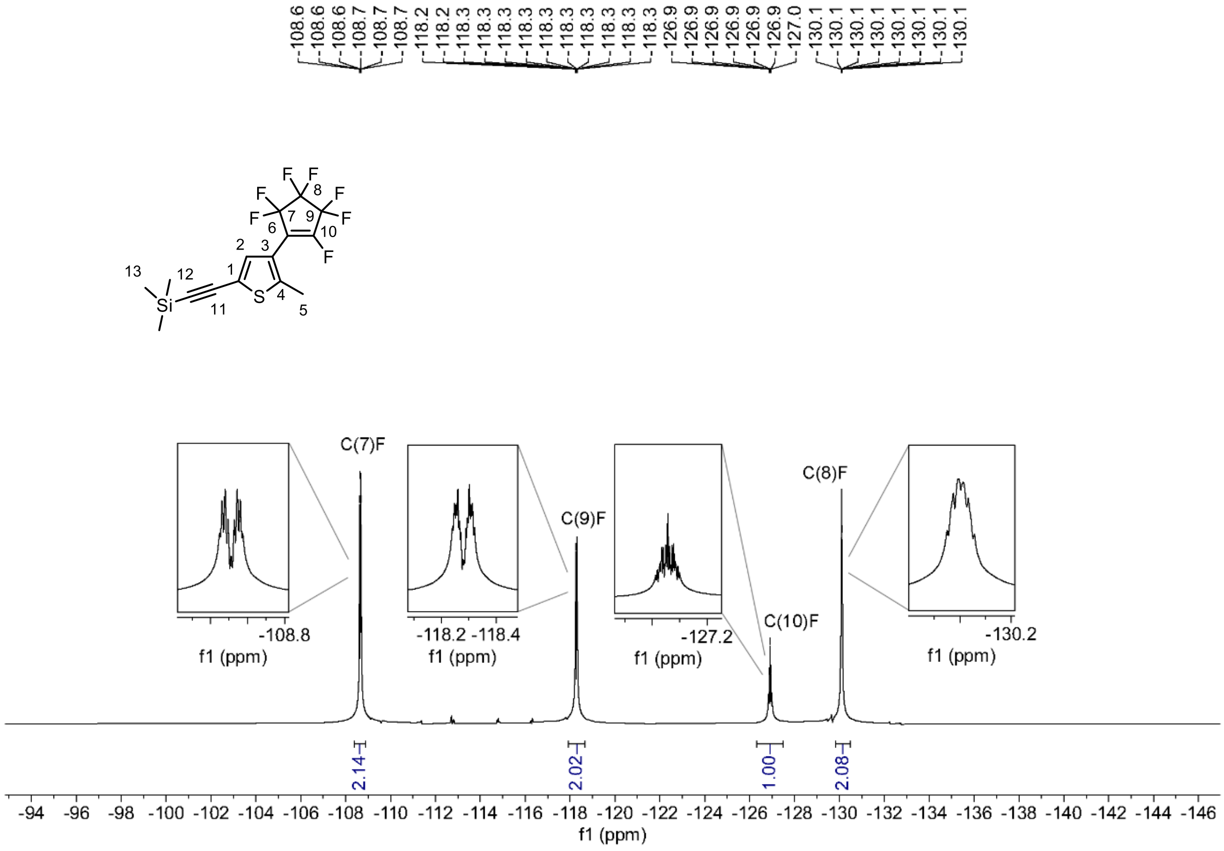

Figure S126 ^{19}F NMR spectrum (376 MHz, $CDCl_3$) of **13**.

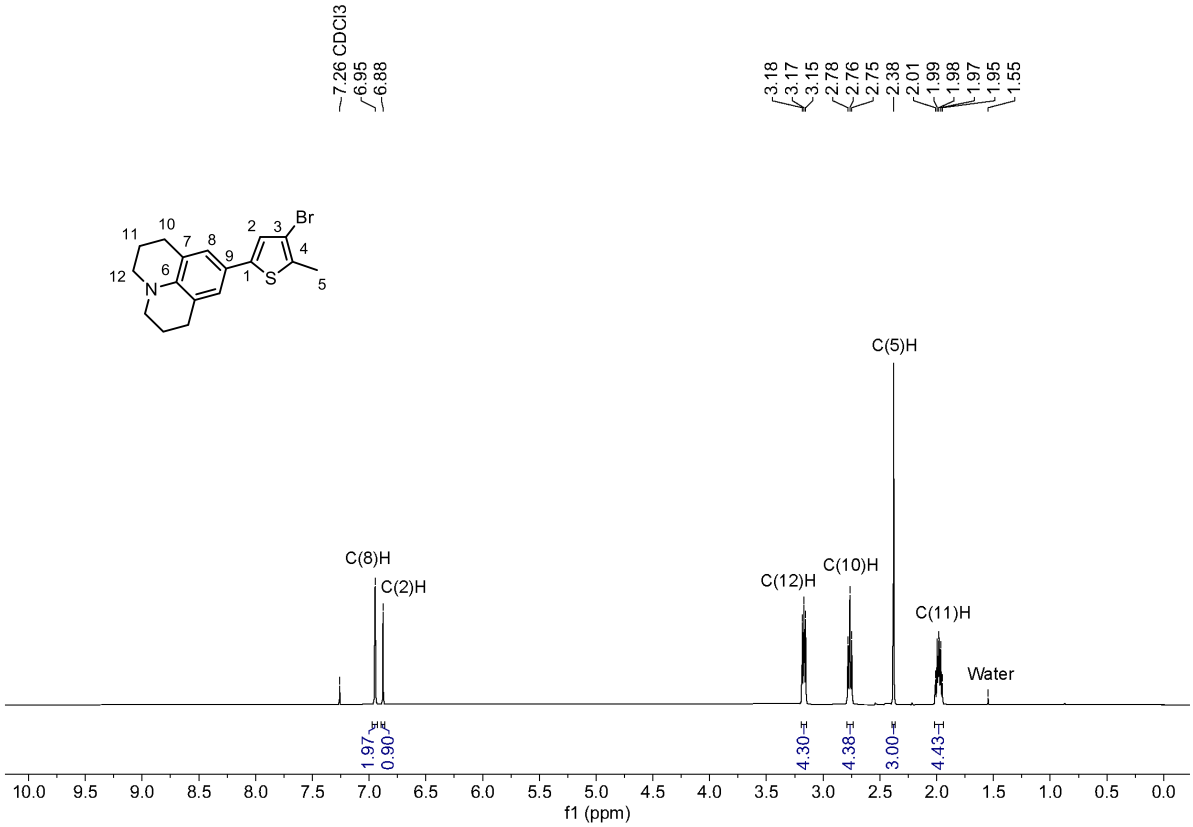

Figure S127 ^{1}H NMR spectrum (400 MHz, $CDCl_3$) of **14**.

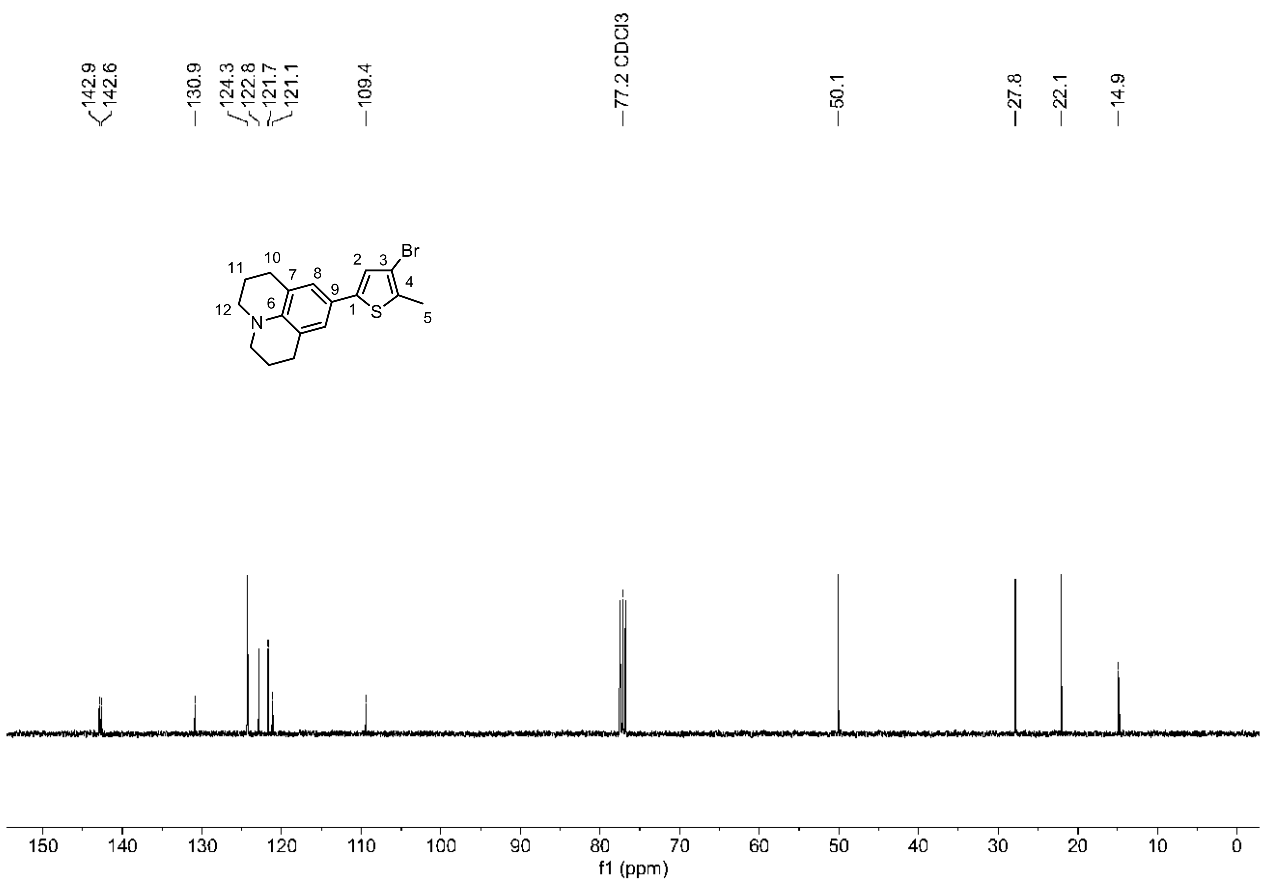

Figure S128 ^{13}C NMR spectrum (101 MHz, $CDCl_3$) of **14**.

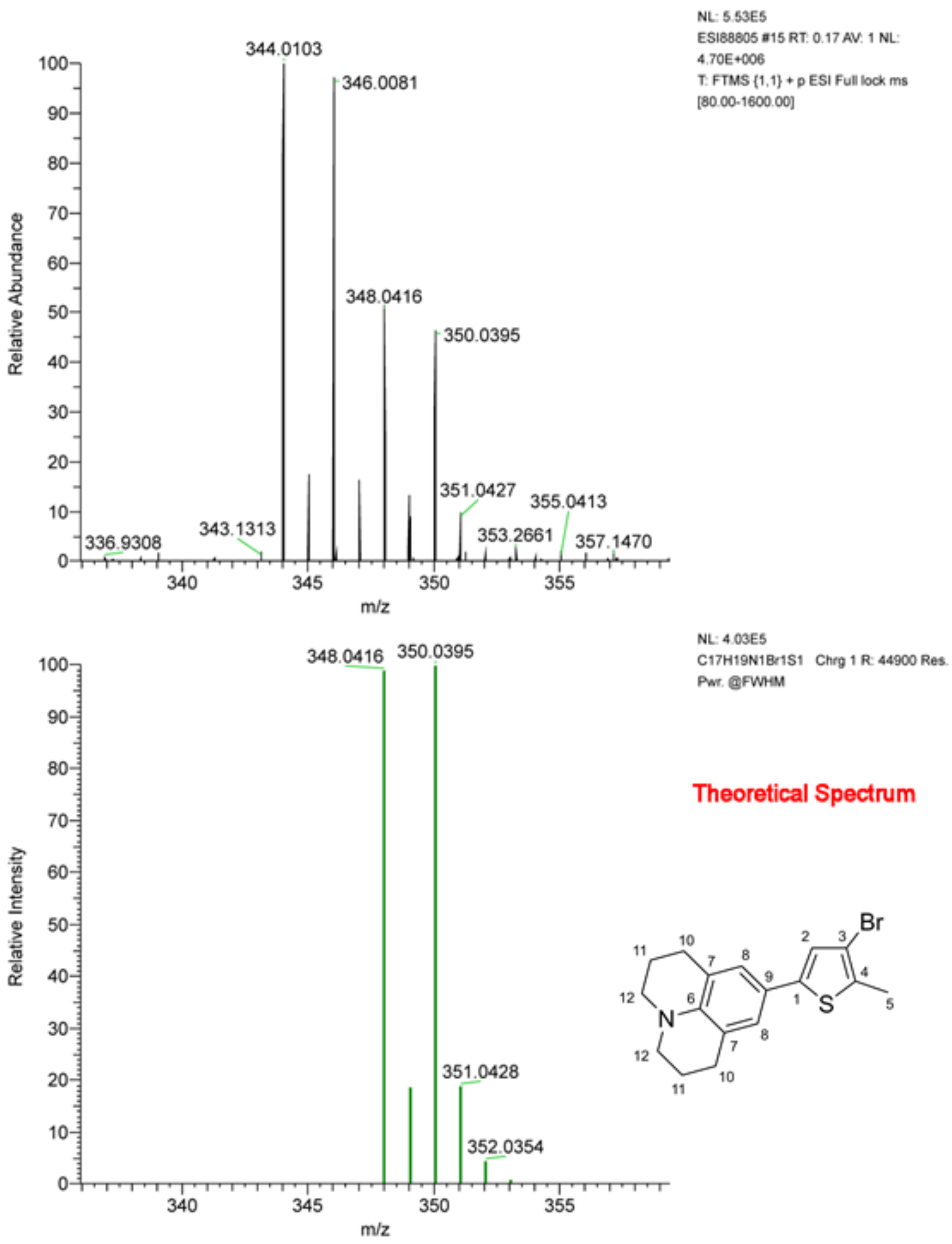

Figure S129 HRMS (ESI$^+$) of **14**, *m/z*: $[M]^+$ calcd. for $C_{17}H_{19}NBrS$ 348.0416; found 348.0416.

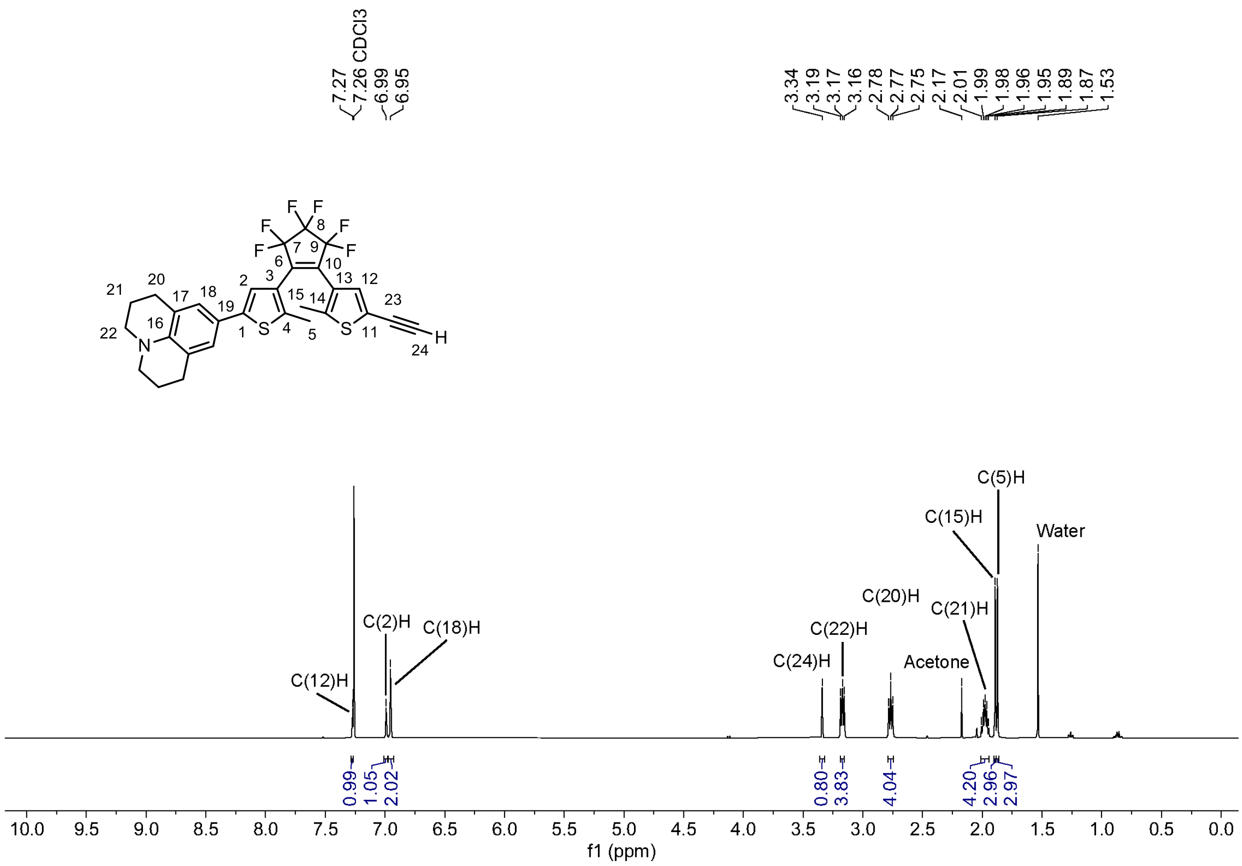

Figure S130 ^{1}H NMR spectrum (400 MHz, $CDCl_3$) of **15**.

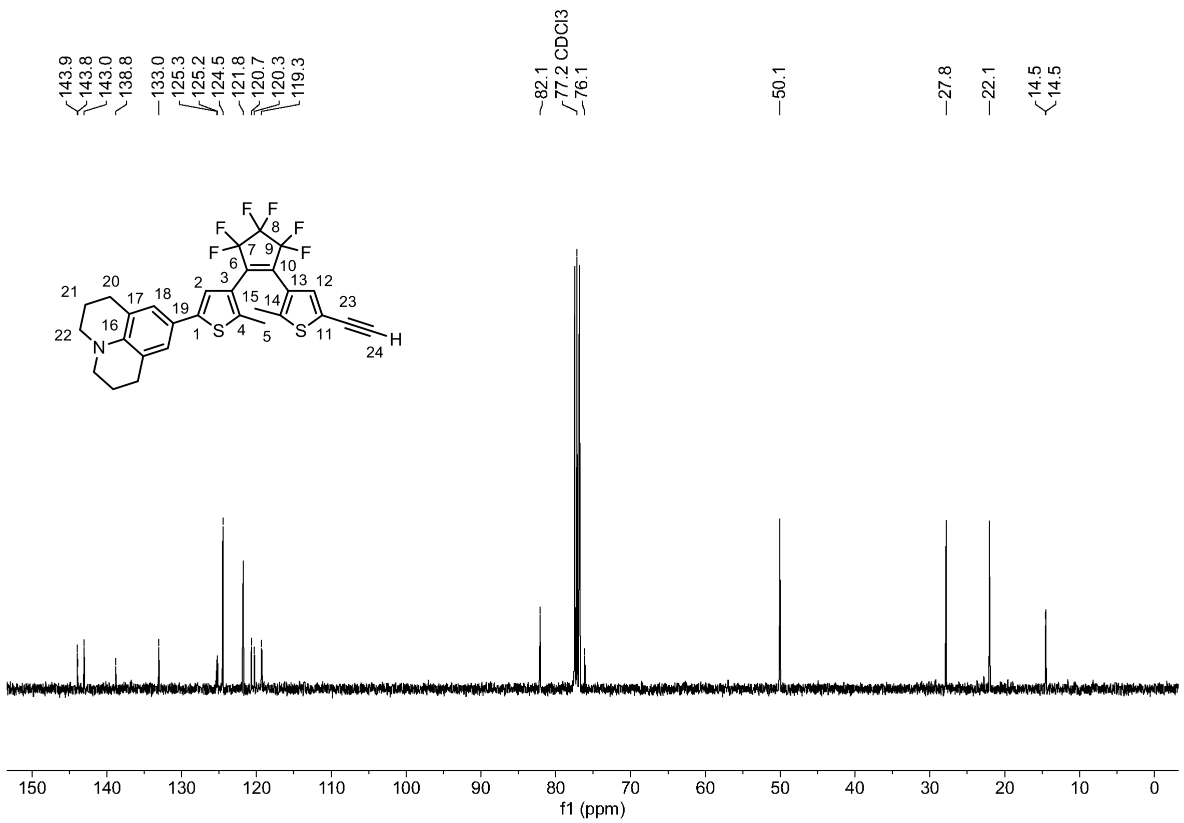

Figure S131 ^{13}C NMR spectrum (101 MHz, $CDCl_3$) of **15**.

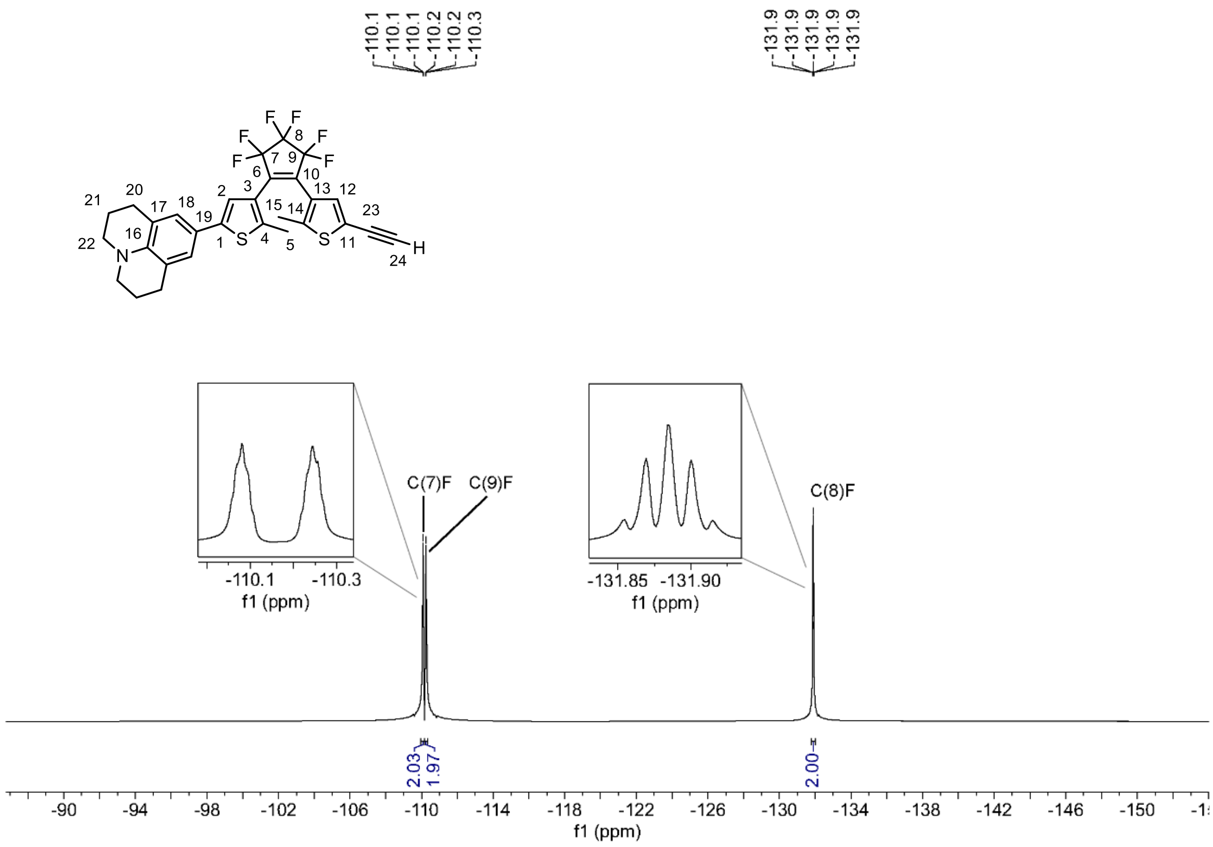

Figure S132 ^{19}F NMR spectrum (376 MHz, $CDCl_3$) of **15**.

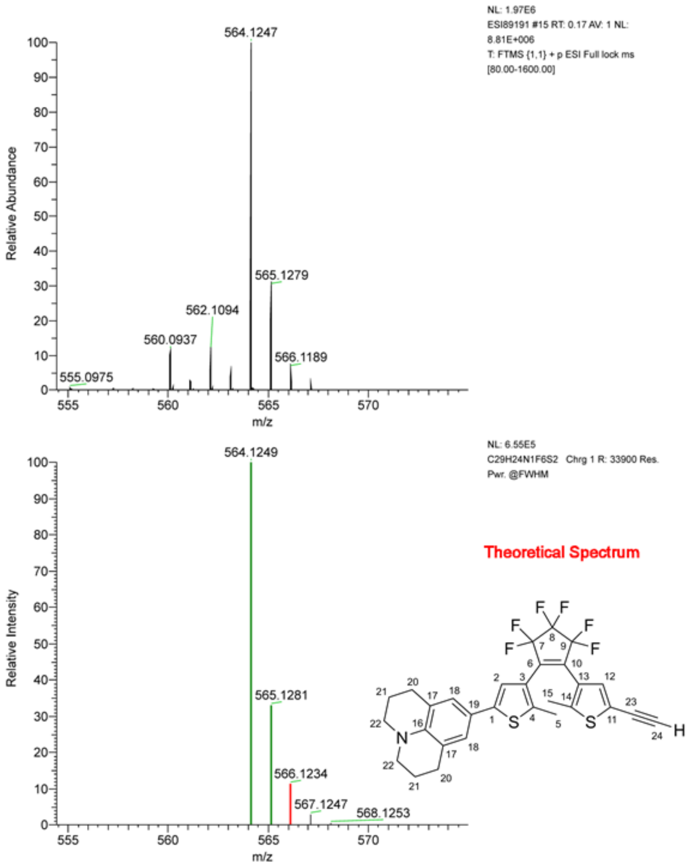

Figure S133 HRMS (ESI$^+$) of **15**, *m/z*: [M]$^+$ calcd. for $C_{29}H_{24}F_6NS_2^+$ 564.1249; found 564.1247.

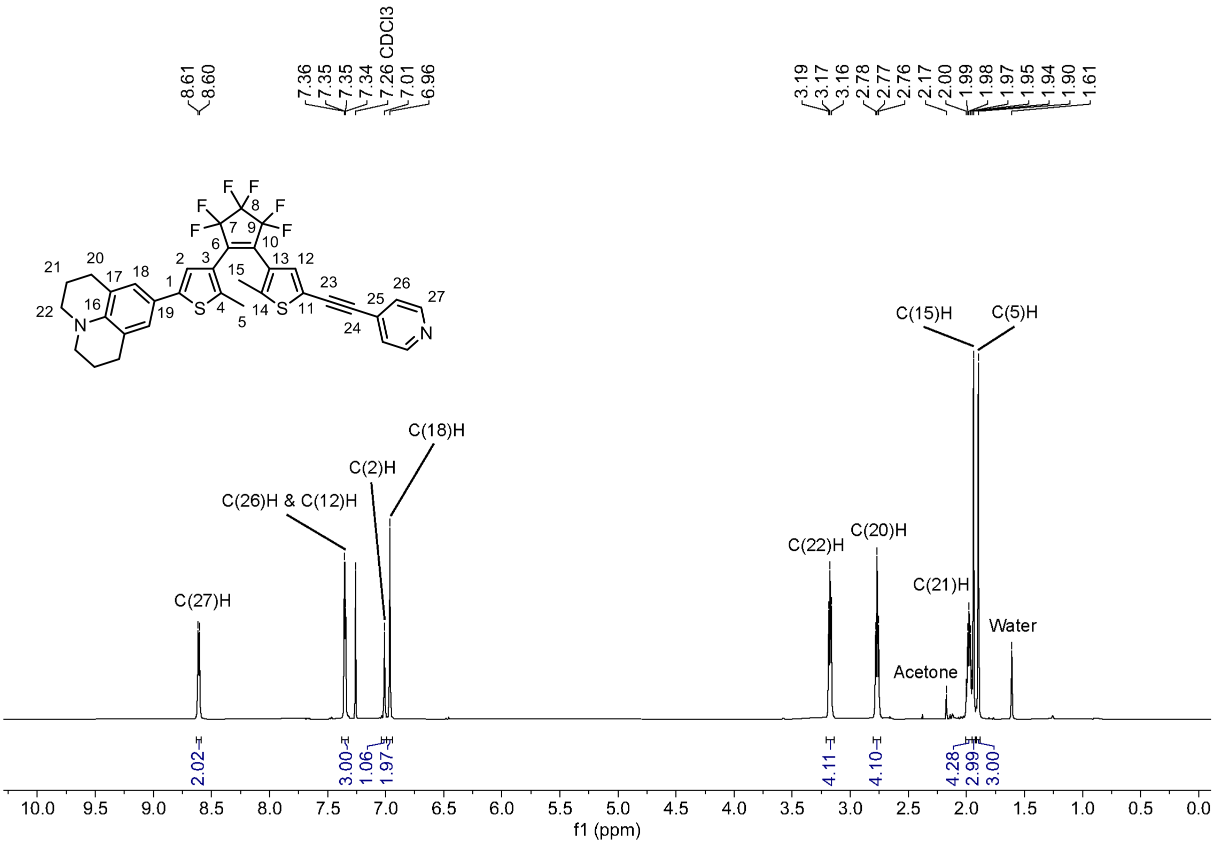

Figure S134 ^{1}H NMR spectrum (400 MHz, $CDCl_3$) of **D-yA(p)**.

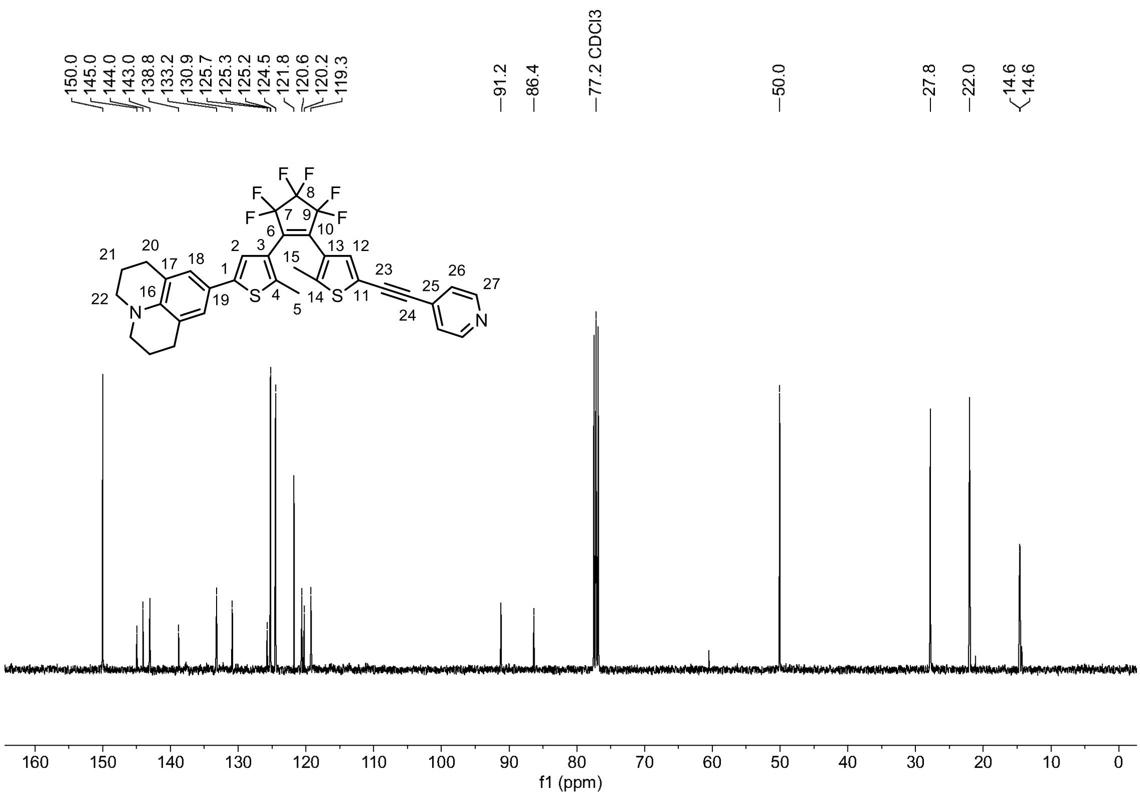

Figure S135 ^{13}C NMR spectrum (101 MHz, $CDCl_3$) of **D-yA(p)**.

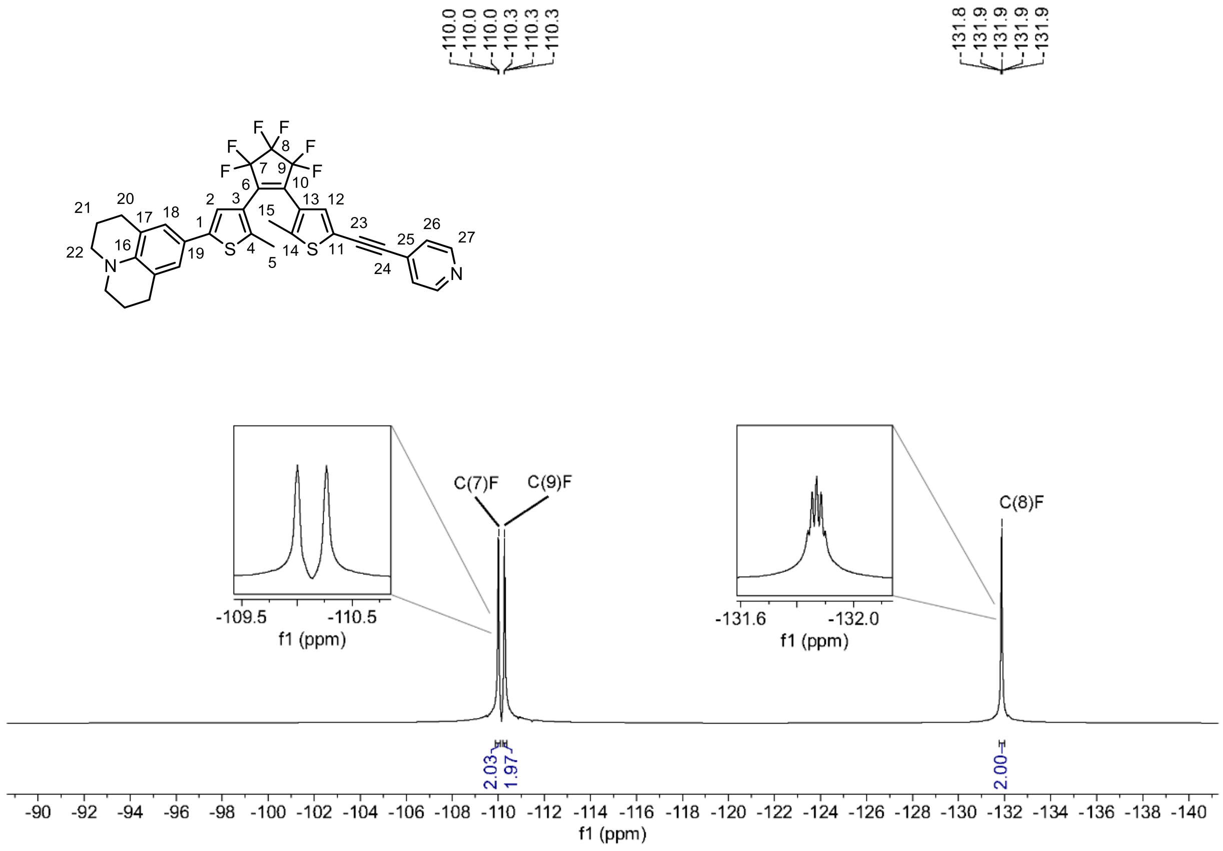

Figure S136 ^{19}F NMR spectrum (376 MHz, $CDCl_3$) of **D-yA(p)**.

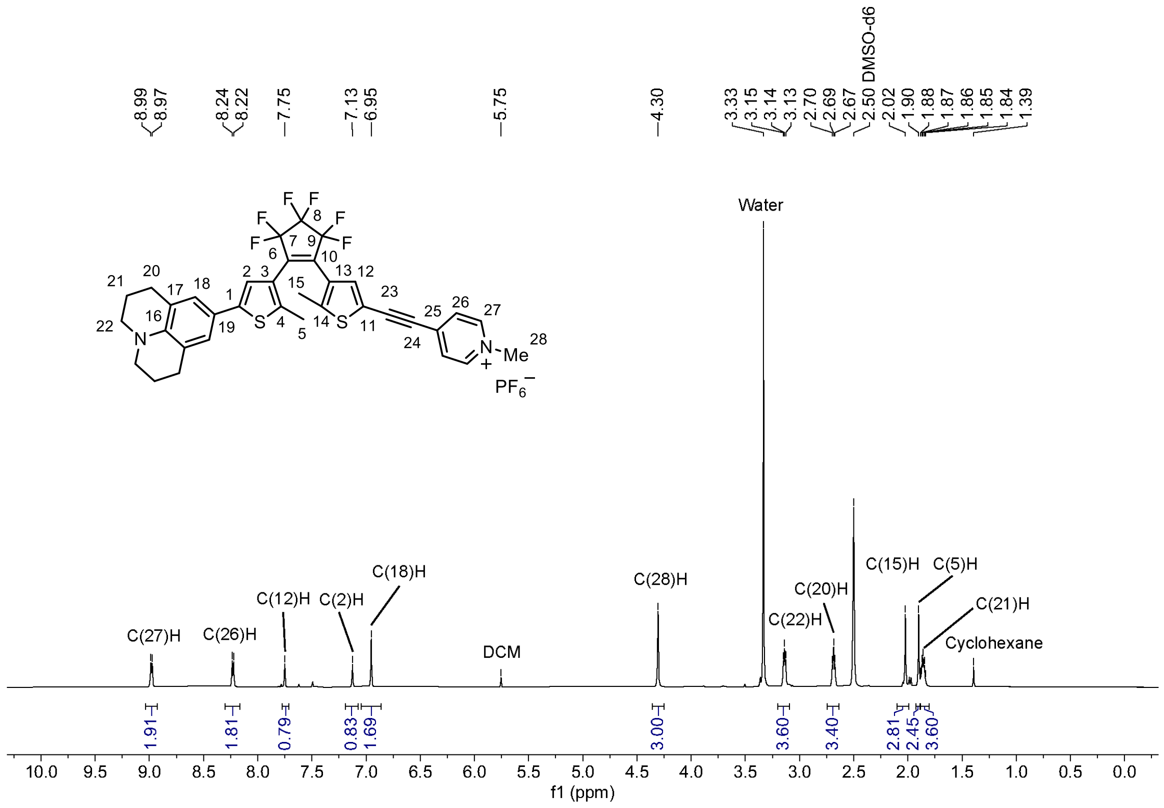

Figure S137 ^{1}H NMR spectrum (400 MHz, DMSO-d_6) of **D-yA**.

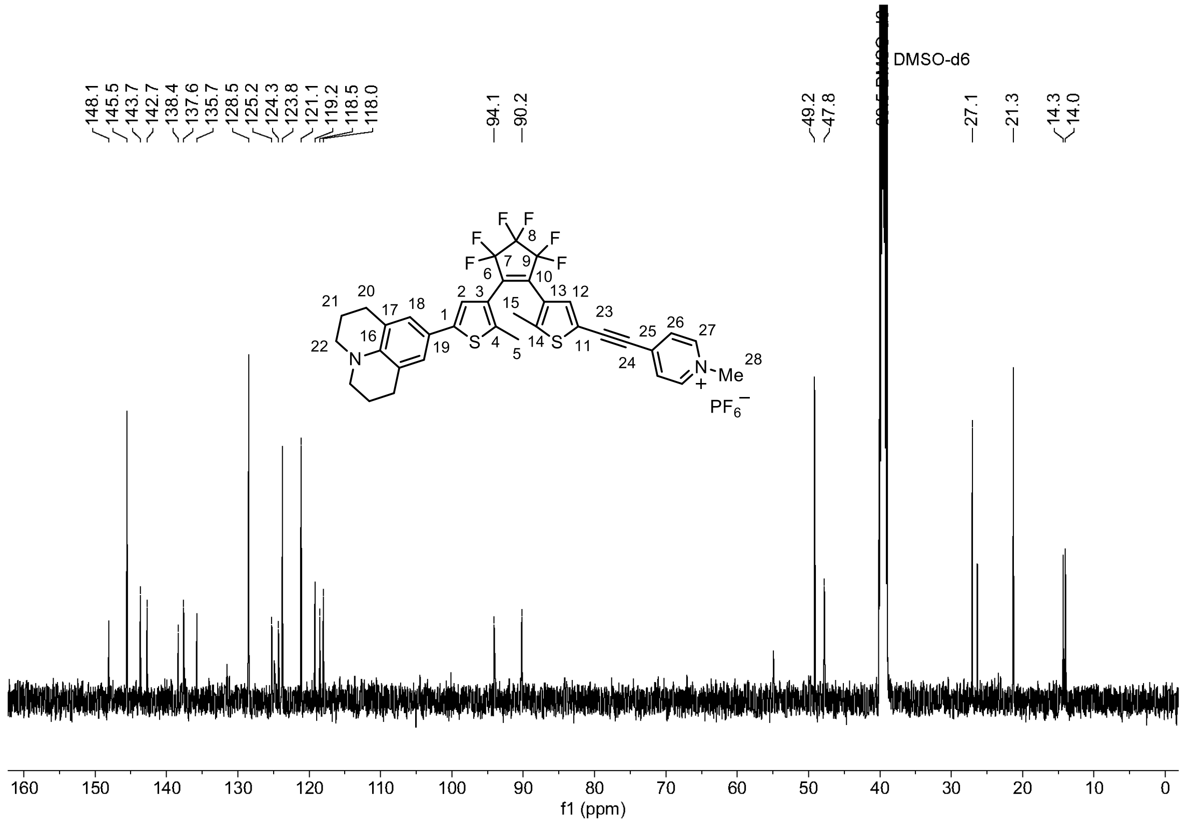

Figure S138 ^{13}C NMR spectrum (101 MHz, DMSO-d$_6$) of **D-yA**.

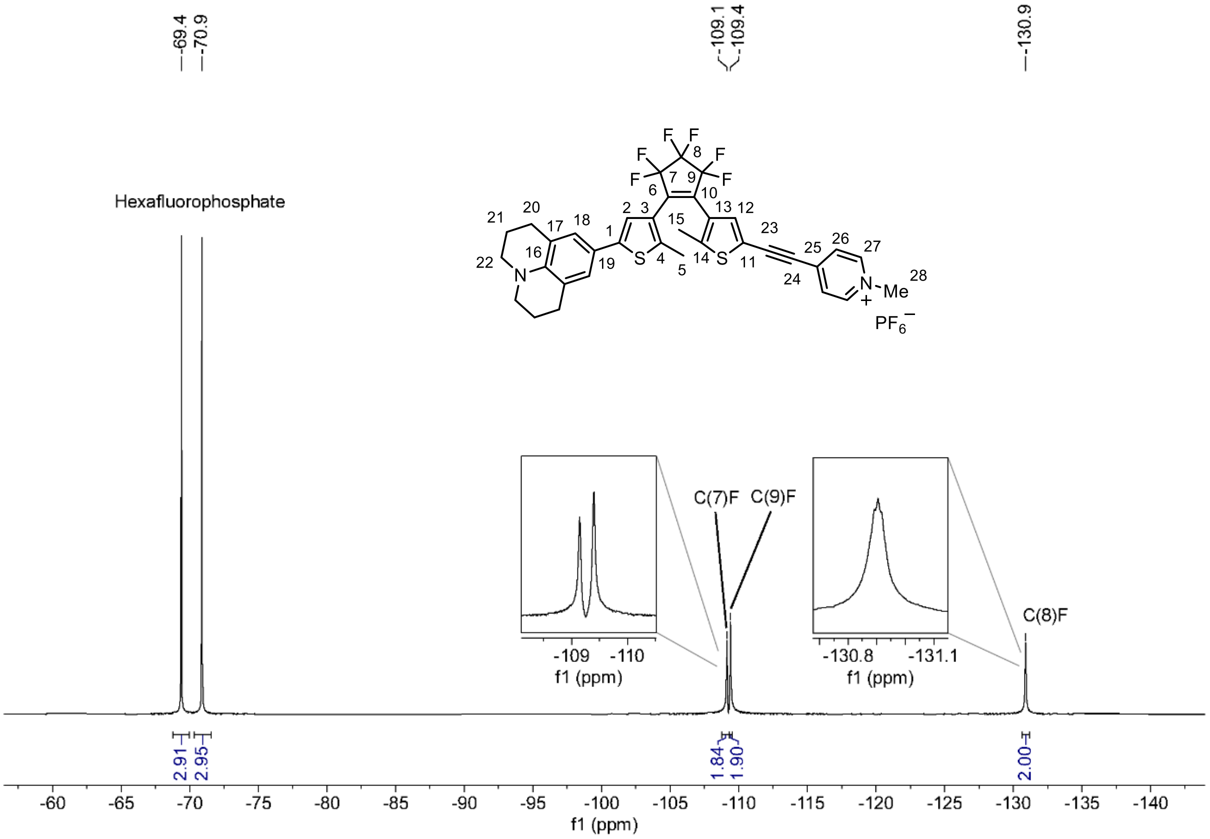

Figure S139 ^{19}F NMR spectrum (376 MHz, DMSO-d$_6$) of **D-yA**.

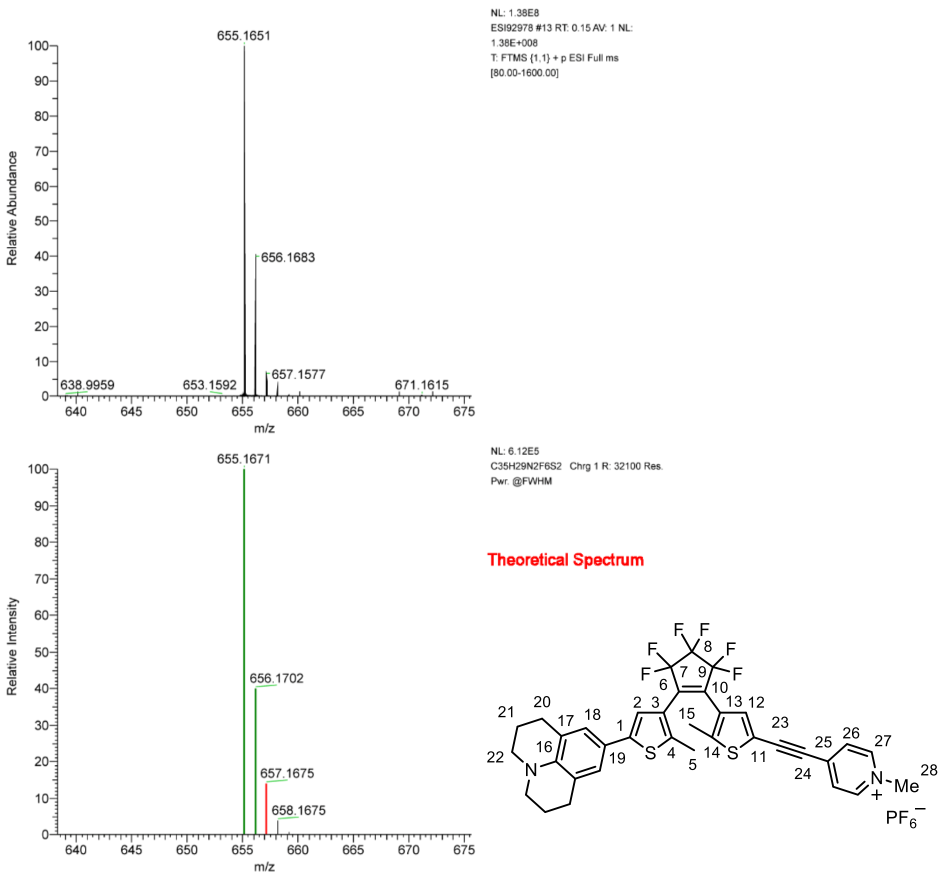

Figure S140 HRMS (ESI$^+$) of **D-yA**, *m/z*: $[M]^+$ calcd. for $C_{35}H_{29}F_6N_2S_2^+$ 655.1671; found 655.1651.

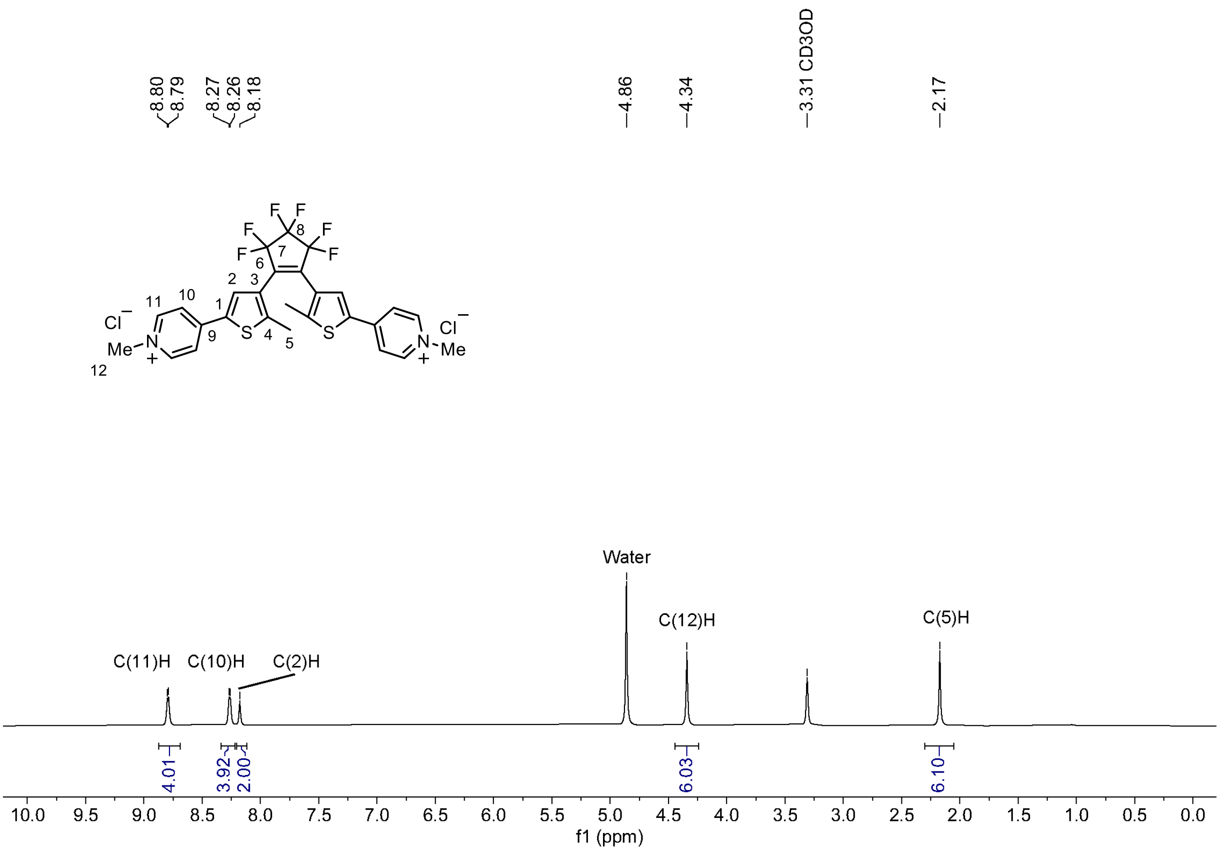

Figure S141 ^{1}H NMR spectrum (400 MHz, CD_3OD) of **A-A** (chloride).

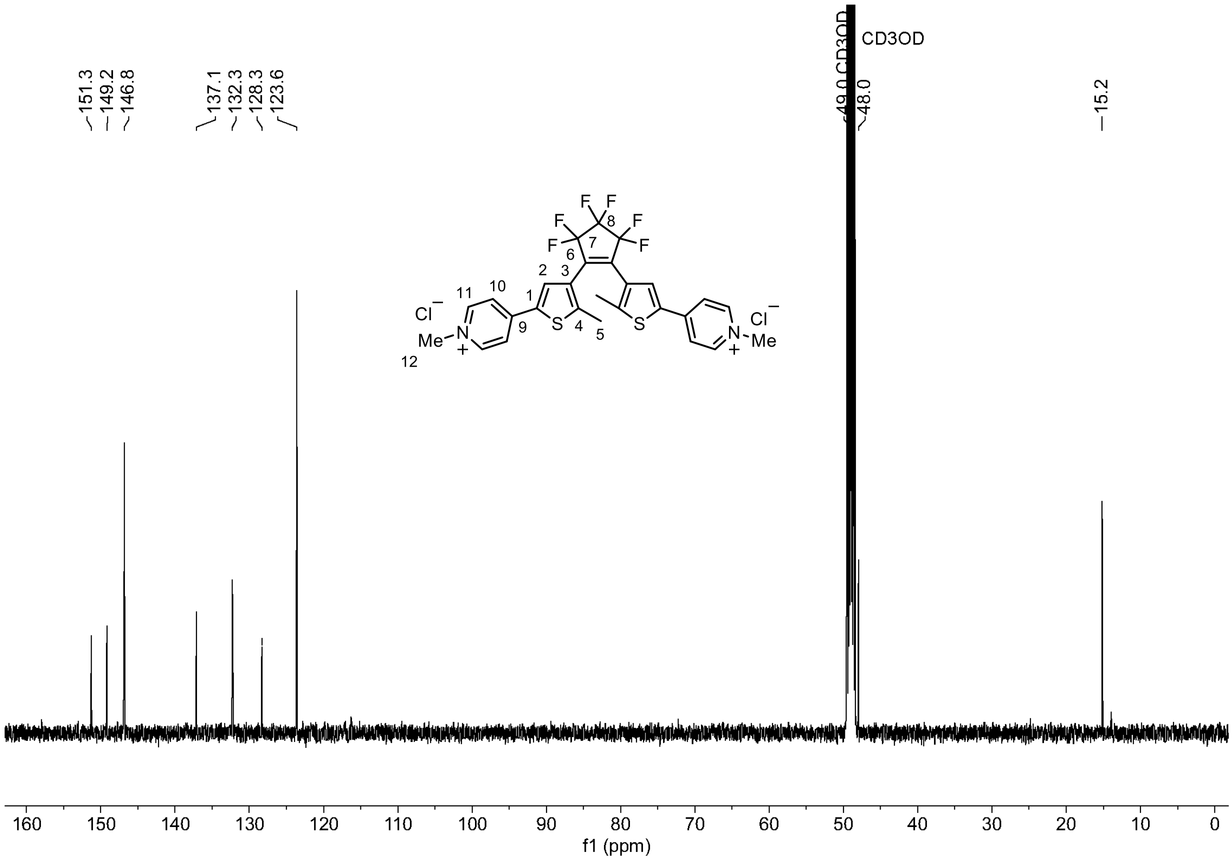

Figure S142 ^{13}C NMR spectrum (101 MHz, CD_3OD) of **A-A** (chloride).

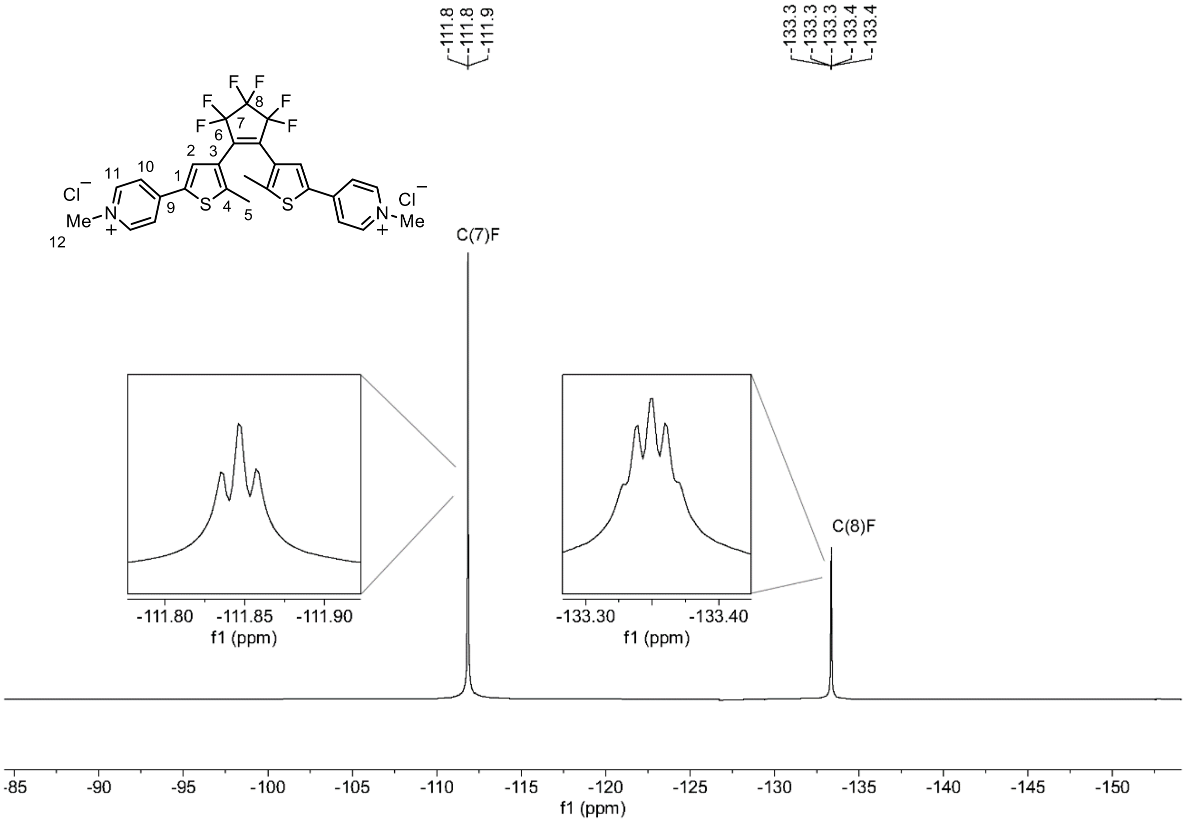

Figure S143 ^{19}F NMR spectrum (376 MHz, CD_3OD) of **A-A** (chloride).

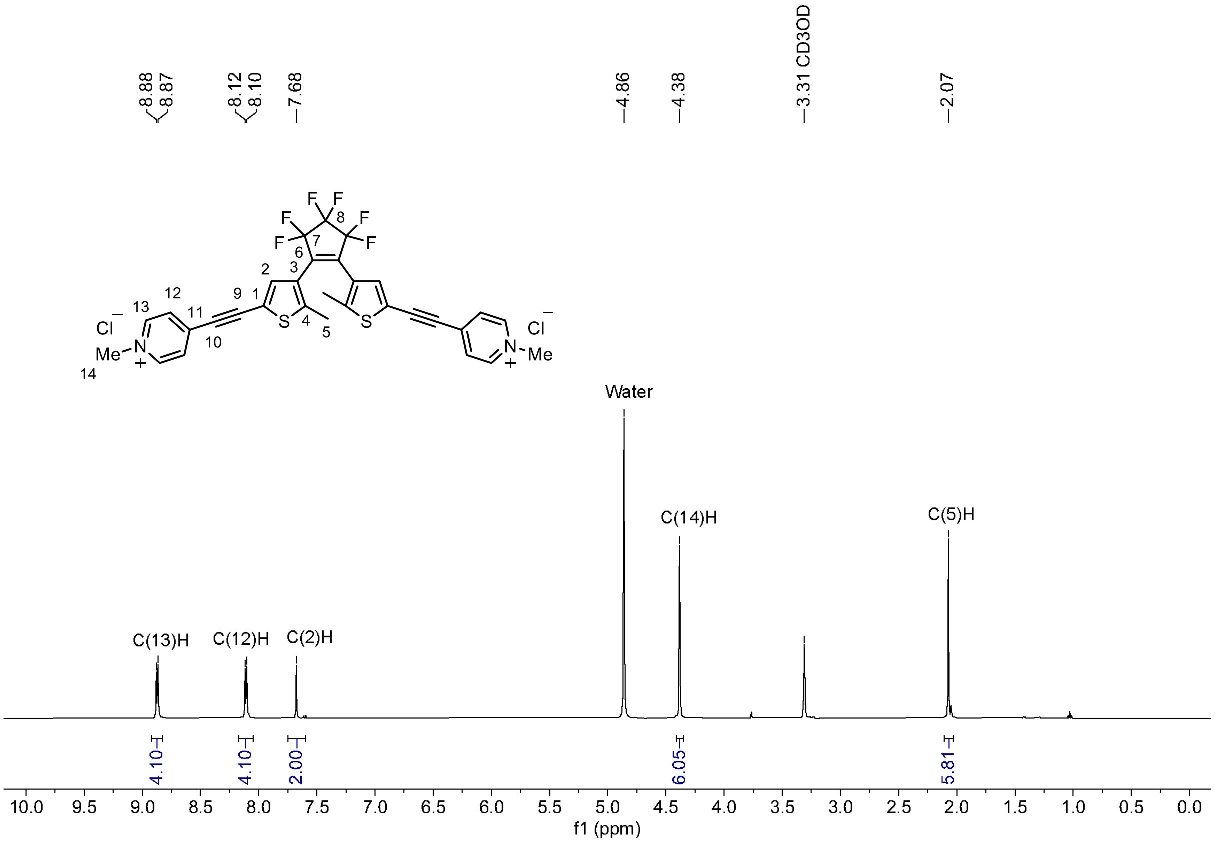

Figure S144 ^{1}H NMR spectrum (400 MHz, CD_3OD) of **Ay-yA** (chloride).

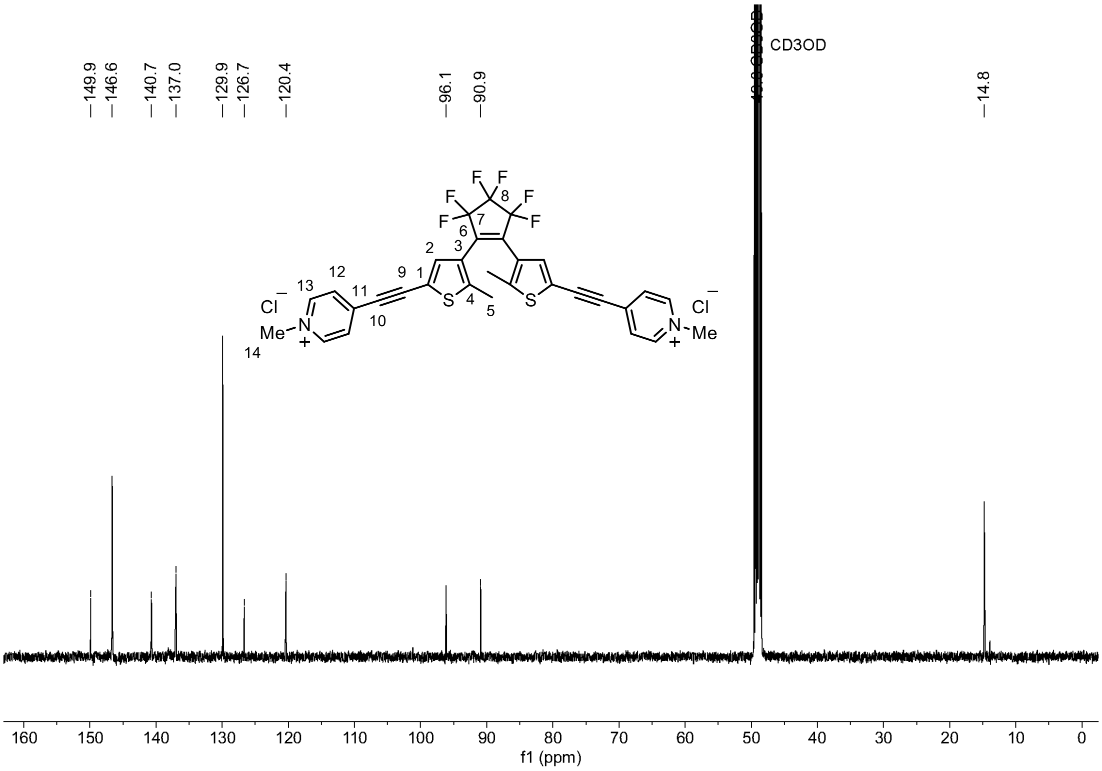

Figure S145 ^{13}C NMR spectrum (101 MHz, CD_3OD) of **Ay-yA** (chloride).

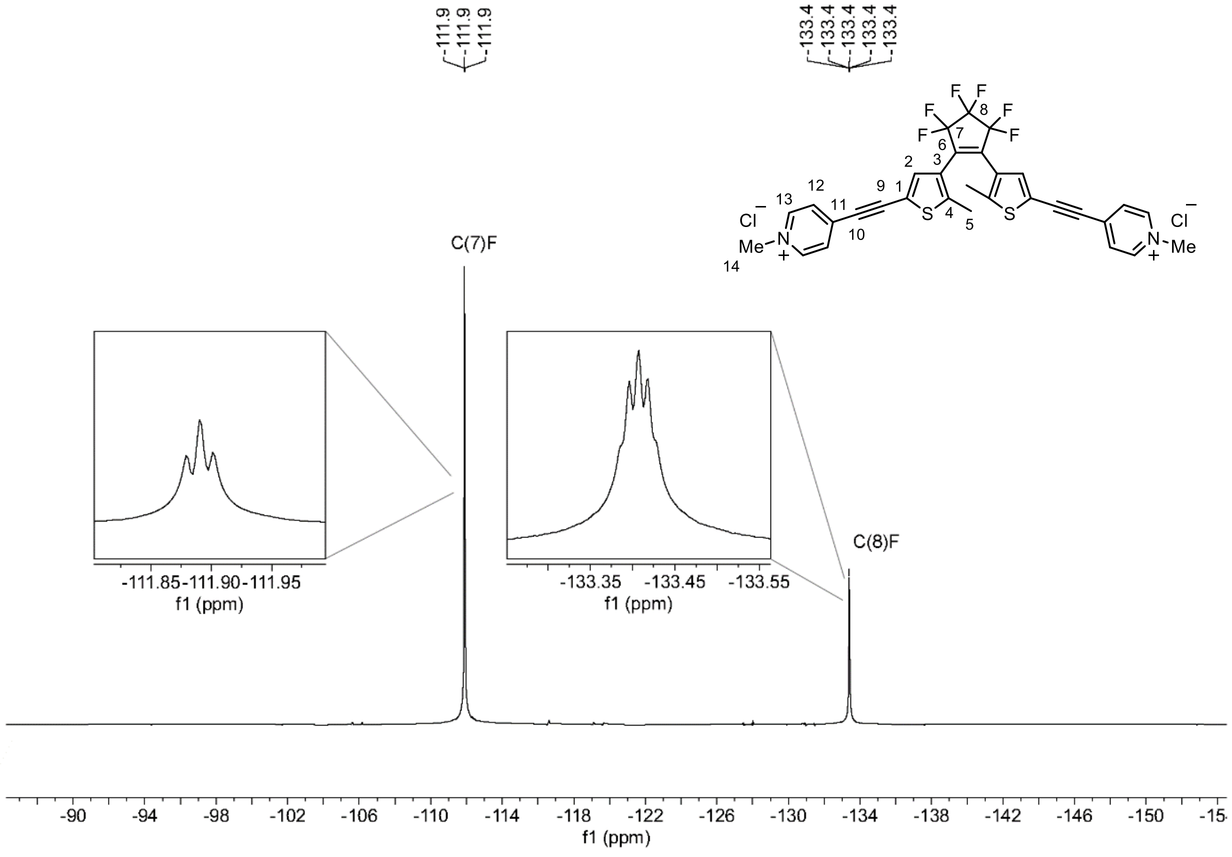

Figure S146 ^{19}F NMR (376 MHz, CD_3OD) of **Ay-yA** (chloride).

S19. Coordinates of Optimized Geometries of DTEs (ωB97X-D/Def2SVP)

Calculated Cartesian coordinates (*x*, *y*, *z*; Å).

Geometry of **D-D** (open):

```
C 0.63559500 2.17127100 0.25269200
C 1.09340400 3.59244800 0.47346800
C 0.02184700 4.45494500 -0.23720100
C -1.21553800 3.52722600 -0.28167800
C -0.63192200 2.13784100 -0.21770700
F -0.21485400 5.60753400 0.39035100
F 0.41898500 4.71813700 -1.49023000
F 2.31922400 3.84189200 -0.02866200
F 1.13381900 3.89524400 1.78617600
F -1.95670500 3.74490400 -1.37721800
F -2.00576900 3.77740700 0.79157200
C -1.45259900 0.98667700 -0.59692900
C 1.49350900 1.04672700 0.63890800
C -2.82792800 0.86832000 -0.20846300
C -3.42022000 -0.29227200 -0.62681000
S -2.28348400 -1.23832700 -1.54032900
C -1.01092000 -0.08825300 -1.33895300
C 1.07285200 0.00407400 1.43775000
S 2.34770500 -1.14155100 1.64759700
C 3.45602500 -0.23890300 0.65881600
C 2.85472500 0.90663100 0.21127800
C 0.33101100 -0.33785500 -1.94893500
C -0.25525300 -0.21552400 2.08889400
C -4.79938500 -0.74953900 -0.40973200
C 4.82589800 -0.71096900 0.41526800
C -5.13312700 -2.10820600 -0.36660400
C -6.43653600 -2.54543100 -0.16315900
C -7.46949700 -1.60008300 0.04161400
C -7.14581400 -0.22189900 0.01415000
C -5.83621000 0.17375500 -0.22601100
C 5.15115700 -2.07235600 0.40485200
C 6.44558300 -2.52369700 0.17632700
C 7.47678900 -1.59149800 -0.08880900
C 7.16123300 -0.21121400 -0.09494700
C 5.86147600 0.19983500 0.17190000
C -6.77472700 -4.01460100 -0.13348500
C -7.92738100 -4.26235200 0.83203000
C -9.12155900 -3.40815300 0.43921800
N -8.77037700 -2.01385200 0.26155300
C -9.88014800 -1.08444000 0.32632000
C -9.55894700 0.24990700 -0.32655700
C -8.24396300 0.79040000 0.22175100
C 6.77499200 -3.99518000 0.18264800
C 9.58591100 0.25609900 0.15920000
C 9.10688800 -3.42124500 -0.47783100
N 8.76838400 -2.01990800 -0.33448000
C 9.88062700 -1.09987400 -0.46077800
C 7.89720300 -4.28006000 -0.80812600
C 8.25802800 0.78730600 -0.36668300
H -3.34136100 1.61447000 0.39750600
H 3.35052800 1.62223800 -0.44284100
H 0.84766400 0.61310400 -2.13697600
H 0.97477200 -0.93139200 -1.27999900
H 0.24092900 -0.87550000 -2.90370400
H -0.76937800 0.74379400 2.23753200
H -0.91066700 -0.84470300 1.46515300
H -0.14430100 -0.70149500 3.06880100
H -4.34870000 -2.86150800 -0.48882600
H -5.61874300 1.24378300 -0.28178300
H 4.36674400 -2.81653200 0.57404200
H 5.65106300 1.27234500 0.20140500
H -5.88859200 -4.60140400 0.15059600
H -7.06616200 -4.35529800 -1.14348600
H -7.61031600 -4.00540900 1.85610500
H -8.22600600 -5.32098000 0.83739800
H -9.57930300 -3.80965400 -0.48804500
H -9.89768300 -3.47324800 1.22172400
H -10.18294800 -0.92145900 1.38092400
H -10.74754700 -1.54504800 -0.17801300
H -10.38737300 0.95031400 -0.14499000
H -9.47483000 0.11672100 -1.41763100
H -8.35978400 1.00533900 1.29945600
H -7.97650800 1.74252800 -0.25988400
H 5.87771600 -4.58496500 -0.05678700
H 7.09429500 -4.30615100 1.19381300
H 10.41224500 0.94539800 -0.06856200
H 9.53349600 0.15745600 1.25590700
H 9.85929500 -3.51587300 -1.28024400
H 9.58963600 -3.79605100 0.44793600
H 10.15308000 -0.97162400 -1.52838900
H 10.76012400 -1.54987700 0.03206400
H 7.55125600 -4.05342100 -1.83000900
H 8.19007400 -5.34013700 -0.78895100
```

H 8.34291300 0.96775200 -1.45357000
H 8.01002900 1.75565400 0.09251800

Geometry of **D-D** (closed):

C -1.24284200 3.90787400 0.07367000
C -0.72492600 2.50385600 0.11952400
C 0.72709800 2.50520200 0.05409600
C 1.21862700 3.91263400 0.16917200
C 0.00487300 4.74540200 -0.33428400
F -0.01825800 5.98393700 0.15898400
F 2.32649400 4.18160800 -0.54609300
F 0.06205800 4.81464100 -1.67451200
F -2.25644000 4.09477400 -0.79620400
F -1.68437400 4.33389500 1.27992300
F 1.48040500 4.26364800 1.45170700
C -1.42605800 1.35221800 0.24935200
C -0.62329500 0.07438400 0.50136800
C 0.62674400 0.08810400 -0.40133900
C 1.42994900 1.36048200 -0.11785600
C -2.83860800 1.11637700 0.24010000
C -3.18945500 -0.19871900 0.17271700
S 1.80803200 -1.28863900 -0.05241600
C 3.19496200 -0.19167600 -0.10771700
S -1.80246600 -1.29463900 0.11669000
C 2.84358600 1.12408300 -0.14160400
C -0.29141400 0.01822600 2.00513200
C 0.29467200 0.06900300 -1.90605200
H -3.56239100 1.93009400 0.25681500
C -4.54233000 -0.75346000 0.11606500
C 4.54860900 -0.74757400 -0.08596600
H 3.56632700 1.93833100 -0.16042300
H 0.22465200 -0.91591200 2.26086600
H -1.22256200 0.08215400 2.58429900
H 0.35298100 0.86421300 2.28632900
H 1.22571900 0.14852400 -2.48343400
H -0.35096100 0.92064900 -2.16642600
H -0.21989100 -0.85931800 -2.18486700
C -4.79615500 -2.10634600 0.38155300
C -6.07338500 -2.64647600 0.32977900
C -7.17531200 -1.81225700 0.01935500
C 5.65664100 0.05561400 0.22625100
C 6.95121800 -0.43867800 0.23303000
C 7.18255800 -1.80822300 -0.05641500
C -6.93806600 -0.43874400 -0.24579400
C -5.64380500 0.05482200 -0.20622200
H -3.96714400 -2.76711200 0.65039900
C -6.31905500 -4.10717800 0.61167700
C -9.17307400 -0.34833000 -1.32109900
C -9.58908900 -1.54545100 -0.48294500
H -5.48984100 1.10968000 -0.44576900
C 6.07440900 -2.63752200 -0.35723500
C 4.79659100 -2.09638200 -0.37618400
H 5.50785800 1.10705400 0.48338400
C 8.12700100 0.44680100 0.56026100
C 7.66793800 -4.26442100 -1.34262900
C 8.76033500 -3.67602500 -0.46538600
H 3.96201800 -2.75289200 -0.63822200
C -8.76094500 -3.68527800 0.36990800
N -8.45376800 -2.32489200 -0.02794700
H -5.52128600 -4.50743200 1.25446300
H -6.28305000 -4.68435300 -0.32983600
H -10.05862300 0.26697200 -1.53730700
H -8.77061100 -0.69513100 -2.28698400
H -9.72637900 -3.67799400 0.90467300
H -8.90485400 -4.32082700 -0.52714200
N 8.46159600 -2.32172900 -0.04141400
C -7.68623700 -4.28701800 1.25981500
C -8.10692400 0.45197600 -0.58349700
H -10.19007000 -1.20581900 0.38465600
H -10.24445000 -2.20598800 -1.07646600
H -7.69660100 -3.78641000 2.24188900
H -7.91250000 -5.34953200 1.43152900
H -8.54102900 0.87038800 0.34241500
H -7.76839800 1.30831000 -1.18504700
C 9.60582000 -1.54945600 0.40327700
H 7.80078400 1.29494500 1.17994900
H 8.54349800 0.87771400 -0.36800800
H 7.89035000 -5.32430700 -1.53449500
H 7.65905500 -3.74933600 -2.31719900
H 10.27174100 -2.21867500 0.97495700
H 10.19075300 -1.19776800 -0.47049100
C 9.20659500 -0.36412700 1.26605300
C 6.31387200 -4.09401500 -0.66506100
H 9.71497800 -3.66097100 -1.01907000
H 8.92180000 -4.32490900 0.41907200
H 10.09649700 0.24775300 1.47397100
H 8.82238200 -0.72437200 2.23444800
H 5.50342300 -4.48481000 -1.29773100
H 6.29627800 -4.68484300 0.26847900

Geometry of **A-A** (open):

C -0.65241900 1.35101300 0.16309400
C -1.24397300 2.74535900 0.16633800
C -0.01244500 3.68071400 0.25082800
C 1.12842100 2.81692200 -0.34577800
C 0.65681700 1.38745900 -0.16073700
F -0.18661100 4.82149400 -0.39942600
F 0.25276800 3.92685000 1.53743900
F -2.09760100 2.94240700 1.17558100
F -1.91749700 2.96992000 -0.98614300
F 2.29642500 3.05788600 0.27898400
F 1.29926000 3.09131700 -1.64720600
C 1.58104100 0.28712100 -0.46447900
C -1.53828600 0.22013400 0.45839000
C 2.91149800 0.22428900 0.03273700
C 3.61875100 -0.86692600 -0.42322600
S 2.63091000 -1.81925900 -1.48929700
C 1.28916400 -0.76246200 -1.32487900
C -1.21850300 -0.87867700 1.24292500
C -2.88721100 0.18839900 0.00799000
C -3.58090800 -0.92696600 0.42107600
S -2.55739600 -1.94272600 1.39079700
C 0.03120500 -1.02322100 -2.08665300
C 0.06020500 -1.18797600 1.94967400
C 5.00104600 -1.20883100 -0.14128400
H -3.31480700 0.98218900 -0.60410300
C 5.60645300 -2.37593200 -0.65062200
C 6.92691900 -2.65626100 -0.37972000
N 7.67924300 -1.83247400 0.37882300
C 7.13120500 -0.70543300 0.88853300
C 5.81963100 -0.37319600 0.65089200
C -4.97277500 -1.25114900 0.16532600
H 0.57718400 -0.25587300 2.21512500
H -0.11795400 -1.76037800 2.86992000
H 0.73467700 -1.77561100 1.30687600
C -5.56355000 -2.44661900 0.62284500
H 3.31717000 0.97726400 0.70627600
C -6.89252900 -2.71025700 0.37750300
H -0.48029400 -0.07479500 -2.30025900
H 0.23528100 -1.52972100 -3.03951600
H -0.65849600 -1.65492300 -1.50441100
N -7.66684200 -1.84232900 -0.30588800
C -7.13337300 -0.68653400 -0.76391400
C -5.81422700 -0.36921700 -0.54850100
H 5.05148700 -3.08115200 -1.27106200
H 7.41706400 -3.55050800 -0.76655600
H 7.78822900 -0.07637600 1.49124200
H 5.44087800 0.55269600 1.08174700
H -4.99025500 -3.18803000 1.18132800
H -7.37184000 -3.62631400 0.72487100
C -9.09286200 -2.12204000 -0.54199900
H -7.80786800 -0.02251100 -1.30684500
H -5.44695700 0.58027200 -0.93575200
H -9.31984100 -3.14344000 -0.21992100
H -9.70489000 -1.41402600 0.03182800
H -9.31141900 -2.02450200 -1.61245200
C 9.09683700 -2.12866700 0.64346600
H 9.72661800 -1.38080500 0.14431900
H 9.27803400 -2.10946700 1.72515000
H 9.33749400 -3.12374200 0.25564500

Geometry of **A-A** (closed):

C -0.72129600 1.75798500 0.12226700
C -1.24141600 3.17446400 0.07913600
C 0.01096800 4.00529500 -0.34038100
C 1.21811400 3.17828200 0.19910900
C 0.72321200 1.75980100 0.08037000
F -0.01414500 5.24383900 0.12067700
F 0.08074900 4.01364700 -1.67828100
F -2.25662500 3.33306800 -0.78331500
F -1.66443300 3.57027900 1.29066700
F 2.33942700 3.41525800 -0.49446300
F 1.44370900 3.50404000 1.48377200
C -1.41516000 0.60234200 0.22523900
C -0.63747000 -0.69080900 0.48202100
C 0.64013300 -0.67221800 -0.38628400
C 1.41817200 0.61223600 -0.08621200
C -2.83492400 0.39833800 0.16887100
C -3.19041700 -0.91076000 0.07321700
S 1.83896900 -2.03017100 0.01895600
C 3.19822800 -0.90262300 -0.03280000
S -1.83134000 -2.03704300 0.02520400
C 2.83978400 0.40829200 -0.08001100
C -0.36459600 -0.78417300 1.99574300
C 0.36555100 -0.71070700 -1.90242100
C -4.56566100 -1.41098800 0.01454800
C 4.57397400 -1.40367400 -0.02713000
C -4.87058600 -2.75512100 0.29067400
C -6.17704000 -3.19677300 0.23925200
N -7.18525600 -2.36136300 -0.07915500
C 4.86781500 -2.74656400 -0.32149200
C 6.17462900 -3.18997200 -0.31828100
N 7.19459800 -2.35755600 -0.03107900
C -6.92559400 -1.06350300 -0.35782700
C -5.64306800 -0.56713600 -0.32205500
C 6.94640600 -1.06070500 0.26286900
C 5.66421000 -0.56288100 0.27491100
C -8.57692600 -2.84232800 -0.13871800
H -3.53793200 1.23120200 0.18882500
H 3.53898700 1.24410400 -0.10212200
H -1.31098200 -0.70648900 2.54746100
H 0.11556400 -1.73565800 2.25634600
H 0.28713700 0.04043100 2.32020100
H -0.28711500 0.12463900 -2.19616500
H 1.31115500 -0.61206900 -2.45211300
H -0.11368700 -1.65270200 -2.19676000
H -4.08995600 -3.46602400 0.56557900
H -6.44612800 -4.23136400 0.45566200
H 4.07733100 -3.45544900 -0.57234000
H 6.43447200 -4.22378900 -0.54917000
H -7.78357900 -0.44085900 -0.61648300
H -5.48971900 0.48157800 -0.57535400
H 7.81375600 -0.44012500 0.49371600
H 5.52193600 0.48509800 0.53711300
H -8.60797700 -3.89806900 0.14874400
H -9.19309100 -2.25804100 0.55608000
H -8.95667700 -2.73147300 -1.16238900
C 8.58679600 -2.84031200 -0.02273600
H 9.17866100 -2.25451600 -0.73713300
H 9.00298400 -2.73332000 0.98707900
H 8.60624000 -3.89515800 -0.31449000

Geometry of **D-A** (open):

C 0.73986600 1.80523400 0.28172200
C 0.36539900 3.27232800 0.28578300
C 1.73034100 4.00685100 0.24835900
C 2.67762800 2.97224300 -0.41034000
C 2.00642700 1.64656900 -0.15962300
F 1.68405200 5.15567900 -0.41744000
F 2.13699200 4.23690000 1.50306800
F -0.35950500 3.62745000 1.34867100
F -0.34333600 3.58141300 -0.82280300
F 3.91764800 3.03597300 0.12681600
F 2.80795300 3.21728900 -1.72617900
C 2.69928100 0.39792400 -0.50389900
C -0.23696600 0.79257000 0.66778400
C 4.01498800 0.09199900 -0.08371100
C 4.47085100 -1.13541700 -0.52947800
S 3.24901300 -1.90850300 -1.49558700
C 2.15434400 -0.60289400 -1.30269700
C 0.04207000 -0.31069000 1.44938300
S -1.37352400 -1.27960200 1.63354600
C -2.35105600 -0.22360800 0.65463200
C -1.60226300 0.84378500 0.23363700
C 0.81689300 -0.64414900 -1.96060300
C 1.31511000 -0.69670600 2.13433900
C 5.75441500 -1.73805300 -0.28456800
C -3.76650700 -0.51170400 0.40343800
C -4.27435600 -1.81690600 0.42405200
C -5.60926000 -2.09605100 0.16571500
C -6.50850600 -1.03427900 -0.10687900
C -6.01108700 0.29434700 -0.11715600
C -4.66538800 0.52710000 0.12515800
C -6.13234400 -3.50994000 0.17390200
C -8.05896700 0.96480400 -1.34538900
C -8.42144800 -2.60805700 -0.20629800
N -7.83870700 -1.28691700 -0.35311300
C -8.76584100 -0.25041300 -0.76946100
C -7.57563500 -3.52340100 0.66267600
C -6.96716200 1.42779400 -0.38861400
C 6.09089300 -3.02619900 -0.76247900
C 7.32941600 -3.56556300 -0.51470600
N 8.26536900 -2.89281800 0.19077800
C 7.97979700 -1.65656400 0.66680500
C 6.76203300 -1.06591100 0.45127100
H 4.59514800 0.77139500 0.53902900
H -1.99696800 1.63396200 -0.40422300
H 0.47249500 0.37668800 -2.17305300
H 0.84550300 -1.21290100 -2.89961800
H 0.07177500 -1.10685400 -1.29373000
H 1.94898400 0.18773800 2.28788800
H 1.11598800 -1.14437400 3.11850800
H 1.89330400 -1.42757800 1.54451000
H -3.60423400 -2.65830600 0.62746100
H -4.31273000 1.56201000 0.12608600
H -5.49527400 -4.14639400 0.80577800
H -6.08915900 -3.93409300 -0.84547000
H -8.79672000 1.75980600 -1.52584500
H -7.61103900 0.70658000 -2.31894900
H -9.42442200 -2.49421700 0.23897000
H -8.57188900 -3.06854400 -1.20305500
H -9.40125100 0.05435200 0.08570100
H -9.44513000 -0.67992500 -1.52537900
H -7.61293400 -3.17970900 1.70927800
H -7.99815500 -4.53819300 0.63654600
H -7.42905500 1.76402600 0.55690600
H -6.42413600 2.29302600 -0.79637500
H 5.37781800 -3.61940600 -1.33652500
H 7.60845500 -4.55612400 -0.87551700
H 8.77329000 -1.15950900 1.22683700
H 6.59667800 -0.06809000 0.85507900
C 9.59075700 -3.47307700 0.44199000
H 9.62653700 -4.48695500 0.03028300
H 10.36105700 -2.85925500 -0.04265400
H 9.77504100 -3.51748200 1.52287200

Geometry of **D-A** (closed):

C 2.10997000 1.83830700 -0.08172900
C 2.86554100 3.14390600 -0.13489900
C 1.81271800 4.19511400 0.32445800
C 0.44231600 3.54714900 -0.03363300
C 0.73784400 2.08401400 0.03865000
F 1.99374000 5.37980900 -0.24829000
F 1.89161700 4.32860700 1.65527400
F 3.95549900 3.15748000 0.65203100
F 3.26617100 3.41643400 -1.38942900
F -0.52526800 3.94506400 0.81104200
F 0.07018400 3.94046800 -1.27252600
C 2.62977100 0.55929500 -0.19675300
C 1.63254200 -0.57934000 -0.42558900
C 0.42291900 -0.34027700 0.50650200
C -0.16943000 1.02665100 0.18736000
C 3.96217200 0.14979800 -0.15808500
C 4.14748500 -1.22548200 -0.11405300
S -0.97454900 -1.51442100 0.25275500
C -2.14873000 -0.22987900 0.15396200
C -1.55040800 1.04315500 0.12975300
S 2.59712900 -2.10592300 -0.01948500
C 1.24319300 -0.61350800 -1.91405400
C 0.78567000 -0.36393900 2.00612900
C 5.38817900 -1.90482000 -0.09575600
C -3.54734100 -0.51200100 0.08712900
C 5.46589000 -3.32887000 -0.05198300
C 6.67445000 -3.96802400 -0.02647400
N 7.84717400 -3.28554400 -0.04076600
C -4.50391200 0.53113700 0.03771500
C -5.85671500 0.29052800 -0.01318700
C -6.33177400 -1.05928400 -0.04403800
C 7.81807700 -1.92359500 -0.08556600
C 6.64613200 -1.22676200 -0.11497200
C -5.38188500 -2.12959700 -0.00743200
C -4.04014500 -1.83844500 0.06818000
C -6.85836500 1.41624300 -0.03506300
C -8.08030000 1.00482500 -0.84515400
C -8.20473300 -2.66991400 -0.01191400
C -8.66326500 -0.27390500 -0.27322000
N -7.65996200 -1.32000800 -0.10635600
C -7.23714600 -3.64330100 0.63506600
C -5.88196800 -3.55029700 -0.05324800
H 4.78160000 0.86911200 -0.13044700
H -2.11812300 1.96482800 0.01857700
H 2.14982200 -0.71871400 -2.52430300
H 0.57641300 -1.45801600 -2.13310700
H 0.73766700 0.31834900 -2.20977700
H 1.56940500 0.37649100 2.22003900
H -0.10002300 -0.11726400 2.60651700
H 1.15526600 -1.35445800 2.29943800
H 4.55918400 -3.93451100 -0.04590100
H 6.74844000 -5.05565900 0.00549900
H -4.17595400 1.57202200 0.05292700
H 8.78786800 -1.42423300 -0.09754300
H 6.70017900 -0.13971800 -0.15382200
H -3.33848000 -2.67663700 0.10598000
H -6.39686400 2.32521800 -0.44631000
H -7.16752000 1.65877300 0.99699800
H -7.79421200 0.84556900 -1.89744200
H -8.85071700 1.78823900 -0.83204900
H -8.48537100 -3.02023900 -1.02259800
H -9.13573500 -2.61292800 0.57402400
H -9.14592400 -0.07493600 0.70162500
H -9.44622600 -0.66729300 -0.94075400
H -7.65073600 -4.65916700 0.56792200
H -7.12909300 -3.40452800 1.70550500
H -5.98161600 -3.87560100 -1.10392800
H -5.15070700 -4.22360800 0.41619100
C 9.13470900 -3.97764300 -0.00902300
H 9.69662900 -3.68838700 0.88937400
H 8.96535700 -5.05955000 0.01180100
H 9.71949400 -3.72695700 -0.90428900

Geometry of **Dy-yD** (open):

C 0.62118300 2.69034300 -0.25896900
C -0.62334800 2.72776100 0.26675400
C -1.06961900 4.15072300 0.50148200
C -0.03306600 5.00762000 -0.26627000
C 1.20148200 4.07916700 -0.36300000
F 1.88729600 4.28493200 -1.49533800
F 2.04238000 4.33558100 0.66842800
F 0.23328200 6.16410100 0.34044700
F -0.49015000 5.25976600 -1.50068700
F -2.31794200 4.39580900 0.05909400
F -1.04361400 4.45888800 1.81284400
C 1.42241100 1.53224400 -0.66111100
C -1.46244800 1.60324300 0.69445500
C 2.80728100 1.40860400 -0.32265800
C 3.36912700 0.23667300 -0.76284900
S 2.19221700 -0.71095500 -1.62649400
C 0.94730300 0.45819600 -1.38781100
C -1.00623000 0.57007500 1.49008200
S -2.25470100 -0.58853000 1.75713800
C -3.40738500 0.30739600 0.81005900
C -2.83632000 1.45699500 0.32357100
C -0.41641500 0.22618600 -1.95541000
C 0.34642100 0.37387300 2.09710600
C 4.69571700 -0.23114700 -0.59390600
C -4.72746000 -0.17358600 0.62682900
C 5.83026500 -0.64510800 -0.45223800
C -5.85614300 -0.59877900 0.47210900
C 7.16431700 -1.12789400 -0.28379600
C -7.18330500 -1.09458100 0.28734200
C 7.56744400 -2.34929300 -0.84428000
C 8.85470000 -2.84049800 -0.67961500
C 9.80741500 -2.08604700 0.04999700
C 9.41554000 -0.84413600 0.60976000
C 8.11231900 -0.39614600 0.44723400
C -8.11704000 -0.39201900 -0.48927100
C -9.41346400 -0.85289700 -0.66928300
C -9.81229200 -2.07899100 -0.08041000
C -8.87387100 -2.80408100 0.69592800
C -7.59365100 -2.30005600 0.87631500
C 9.27089300 -4.16235900 -1.27382900
C 10.74115800 -4.11097900 -1.67038900
C 11.58619700 -3.73819500 -0.46375800
N 11.09611400 -2.55327300 0.21267700
C 12.06058600 -1.89499800 1.07196100
C 11.40292600 -0.97702600 2.08867500
C 10.42963100 -0.04034200 1.38381800
C -10.41257100 -0.08014600 -1.49280900
C -11.36238500 -1.04470500 -2.19206300
C -12.04017900 -1.93480100 -1.16383900
N -11.09402700 -2.55914100 -0.26002700
C -11.59424200 -3.72525500 0.44114200
C -10.77747700 -4.05457400 1.67948200
C -9.29748400 -4.10910700 1.32134600
H 3.36372000 2.15639300 0.24060600
H -3.37837400 2.16977100 -0.29492200
H -0.92537000 1.18373400 -2.13010400
H -0.36316200 -0.31483800 -2.91077500
H -1.04533800 -0.35900200 -1.26555300
H 0.85309500 1.34107300 2.21722600
H 0.27523800 -0.10251900 3.08512800
H 0.98599200 -0.25595500 1.45796400
H 6.84651000 -2.93462200 -1.42038900
H 7.81971200 0.55707000 0.89437800
H -7.81869700 0.54845500 -0.95903300
H -6.88398000 -2.86259600 1.48801600
H 8.63627500 -4.40448800 -2.13897500
H 9.12030900 -4.97178900 -0.53686300
H 10.88117700 -3.36120100 -2.46624500
H 11.08433600 -5.07772100 -2.06708200
H 11.62648500 -4.59154100 0.24334400
H 12.62620700 -3.54899000 -0.78196900
H 12.78489300 -1.31915300 0.46079700
H 12.64423500 -2.67238200 1.59515600
H 12.18169300 -0.41658000 2.62639200
H 10.85855000 -1.57981000 2.83400500
H 10.99084900 0.61986200 0.69822500
H 9.92092500 0.61664500 2.10450500
H -9.88991400 0.55761200 -2.22075300
H -10.99462900 0.59718300 -0.84218100
H -10.79588100 -1.66684800 -2.90425300
H -12.13058000 -0.50645200 -2.76641600
H -12.78336700 -1.34504900 -0.58971000
H -12.60558900 -2.73219100 -1.67688400
H -11.61215400 -4.60068000 -0.23942300
H -12.64290600 -3.53275700 0.72739500
H -11.12508400 -5.01052200 2.09788700
H -10.94145500 -3.28107500 2.44756700
H -9.12400600 -4.94047000 0.61443100
H -8.68337500 -4.31974000 2.20924400

Geometry of **Dy-yD** (closed):

C 1.24356900 4.48086600 -0.07688700
C 0.72434700 3.07550400 -0.12499800
C -0.72485700 3.07685800 -0.04533800
C -1.21844400 4.48608300 -0.14877300
C -0.00013000 5.31559200 0.34918200
F 0.01822100 6.55664300 -0.13587500
F -2.31940800 4.74799500 0.57708500
F -0.04445400 5.37378000 1.69004300
F 2.26519600 4.66042400 0.78280900
F 1.66993200 4.91016200 -1.28615300
F -1.48910200 4.84040700 -1.42727700
C 1.42386300 1.92365400 -0.26737800
C 0.61822600 0.64549600 -0.51763000
C -0.62150300 0.65556700 0.39956000
C -1.42655100 1.93122600 0.12944700
C 2.83700100 1.69326700 -0.25944200
C 3.17449600 0.37272800 -0.20233500
S -1.80430200 -0.72865900 0.06436600
C -3.18009100 0.38146400 0.11671700
S 1.79778800 -0.73560600 -0.15896000
C -2.84092800 1.70186700 0.15313600
C 0.27008600 0.59805900 -2.01847600
C -0.27320100 0.63158300 1.90099900
C 4.47968500 -0.16534300 -0.15700900
C -4.48624700 -0.15637600 0.09977700
C 5.59597200 -0.64954200 -0.11425000
C 6.90526800 -1.20877200 -0.06562800
C -6.91263300 -1.20134700 0.05800200
C -5.60289000 -0.64109800 0.07989000
C -7.10340200 -2.59176900 0.01162900
C -8.36993800 -3.15410300 -0.02325500
C -9.51344000 -2.31460000 0.01450900
C 7.09594100 -2.59876700 -0.00668100
C 8.36196700 -3.15997400 0.05366700
C 9.50549600 -2.31987400 0.03007500
C -9.33296700 -0.90836900 0.07438700
C -8.05135900 -0.38007900 0.08210600
C -8.56273300 -4.64799400 -0.09010300
C -9.83194100 -5.03937800 0.65616700
C -11.97531600 -2.04257200 -0.11792500
C 9.32530900 -0.91412000 -0.04211600
C 8.04376700 -0.38681300 -0.07550300
C 8.55443000 -4.65325800 0.13357700
C 9.83652400 -5.04859500 -0.58816300
C 11.96453000 -2.04527300 0.20286500
C -11.01867000 -4.28497700 0.08114500
N -10.77977000 -2.85676400 -0.00660700
C -11.68625400 -0.65644600 -0.66858300
C -10.54849000 -0.01729200 0.11756300
C 11.01267000 -4.28961700 0.00232100
N 10.77147600 -2.86098200 0.07647900
C 11.66537600 -0.65579500 0.73947800
C 10.54089200 -0.02251400 -0.07018200
H 3.57648600 2.49257400 -0.24685700
H -3.57923700 2.50207000 0.14989500
H 1.19511300 0.65677000 -2.60784400
H -0.25534300 -0.33129500 -2.27248400
H -0.37062700 1.44983500 -2.29038100
H 0.25007900 -0.29476200 2.16989700
H 0.36930700 1.48616900 2.15943700
H -1.19808400 0.70158700 2.48935400
H -6.22996000 -3.24819500 -0.00523700
H 6.22269600 -3.25563900 -0.00051200
H -7.92427200 0.70470800 0.11582800
H -7.68410800 -5.16125200 0.32726100
H -8.64473500 -4.97083500 -1.14358400
H -9.72053100 -4.79508900 1.72529300
H -10.02231900 -6.12024800 0.58540800
H -12.47030200 -1.95840100 0.87045300
H -12.68727700 -2.56569400 -0.77940200
H 7.91661000 0.69763200 -0.11858300
H 7.68341900 -5.16980900 -0.29551200
H 8.61844500 -4.96917000 1.19039400
H 9.74343100 -4.81136900 -1.66062600
H 10.02630400 -6.12883800 -0.50706600
H 12.47620800 -1.96726000 -0.77747300
H 12.66535100 -2.56365700 0.87977000
H -11.27177400 -4.68944000 -0.91952600
H -11.90669800 -4.44674500 0.71624700
H -12.60143700 -0.04883500 -0.61623700
H -11.40274500 -0.73172800 -1.73115700
H -10.86816100 0.13384200 1.16432900
H -10.30196600 0.97754700 -0.28131300
H 11.24862700 -4.68723100 1.00989000
H 11.91166400 -4.45490100 -0.61621000
H 12.58098700 -0.04794700 0.69882500
H 11.36376500 -0.72434700 1.79751000
H 10.87837600 0.12206300 -1.11227200
H 10.28697300 0.97470800 0.31795000

Geometry of **Ay-yA** (open):

C 0.64396000 1.68783900 -0.19644800
C -0.64483200 1.72717700 0.20022200
C -1.10593700 3.15615300 0.40196000
C -0.00067300 4.01392900 -0.26534200
C 1.23491700 3.08049600 -0.24178100
F 2.03028500 3.27261100 -1.29901400
F 1.96972000 3.31725200 0.86942000
F 0.20875300 5.16286300 0.36314100
F -0.33808500 4.25139400 -1.53750800
F -2.30775800 3.39475900 -0.15365200
F -1.19890200 3.44300200 1.71017000
C 1.50278100 0.54696400 -0.52940600
C -1.54497600 0.62230200 0.55364400
C 2.86514500 0.48967600 -0.12572300
C 3.50980800 -0.64715800 -0.56389800
S 2.43414700 -1.64559100 -1.49649800
C 1.12960100 -0.54681800 -1.29905800
C -1.20039100 -0.41817900 1.40700200
S -2.51053600 -1.50455700 1.62950600
C -3.55010100 -0.57294900 0.59262500
C -2.89027000 0.53512500 0.10336800
C -0.18468500 -0.82618700 -1.95278000
C 0.09458500 -0.64896000 2.11622400
C 4.85890900 -0.99370100 -0.36435300
C -4.89117800 -0.93448500 0.36840900
C 6.03258800 -1.28261900 -0.20553600
C -6.05884400 -1.23576000 0.18854800
C 7.40095600 -1.58760200 -0.04080100
C -7.42110700 -1.55355500 0.00073700
C 7.96390700 -2.76407000 -0.57964700
C 9.30507300 -3.02296400 -0.40853500
N 10.10571200 -2.17259700 0.27006500
C -8.26132600 -0.73950400 -0.78883700
C -9.58858500 -1.06999500 -0.94298200
N -10.11470900 -2.16580100 -0.35371800
C 9.59392200 -1.03744900 0.80015900
C 8.26384500 -0.71900300 0.66571100
C -9.33322700 -2.96673600 0.40737400
C -8.00045000 -2.69397400 0.60222400
C 11.54080300 -2.45562700 0.43359900
C -11.53985700 -2.49696100 -0.51339600
H 3.35659400 1.26128700 0.46616200
H -3.35952700 1.26285100 -0.55645500
H -0.69604700 0.11685200 -2.18879700
H -0.05607100 -1.39360200 -2.88443900
H -0.84283700 -1.40684000 -1.28717100
H 0.76736200 -1.28076000 1.51466800
H 0.60251600 0.30912500 2.29186000
H -0.06047100 -1.14209400 3.08533000
H 7.35050600 -3.47347600 -1.13460300
H 9.77564300 -3.92007500 -0.81257100
H -7.87380300 0.15359100 -1.27843800
H -10.26820200 -0.46353800 -1.54287700
H 10.29428600 -0.39390600 1.33499100
H 7.88808200 0.20502700 1.10453300
H -9.82021300 -3.83436300 0.85537300
H -7.40328600 -3.36138300 1.22311500
H 11.76762500 -3.43751500 0.00605100
H 12.12719600 -1.68797500 -0.08758100
H 11.79327100 -2.45947900 1.50127500
H -11.99502500 -1.80411300 -1.22836200
H -11.63662700 -3.52179000 -0.89266900
H -12.04804500 -2.40603800 0.45533500

Geometry of **Ay-yA** (closed):

C -1.24061200 3.55439800 0.09106300
C -0.71940600 2.14001200 0.13423100
C 0.72329100 2.14116400 0.06282000
C 1.22097000 3.55870100 0.16122800
C 0.00299600 4.38332000 -0.35462700
F -0.01306600 5.62483900 0.10504500
F 2.32567600 3.79533800 -0.55891500
F 0.04579700 4.39451000 -1.69419000
F -2.27055100 3.71209400 -0.75436800
F -1.64287300 3.96249900 1.30738500
F 1.47626200 3.89696700 1.43876900
C -1.41335900 0.98517000 0.26212100
C -0.62752100 -0.30532600 0.51221500
C 0.62954800 -0.29361000 -0.38429100
C 1.41694700 0.99205700 -0.11002000
C -2.83141500 0.78062200 0.23405500
C -3.17669300 -0.53588400 0.15750200
S 1.82768200 -1.66367500 -0.01067100
C 3.18196400 -0.53089100 -0.07854700
S -1.82288400 -1.66899900 0.10711400
C 2.83593400 0.78660700 -0.12716700
C -0.31616800 -0.37852600 2.02034300
C 0.31697100 -0.33472300 -1.89368900
C -4.50791700 -1.01131000 0.09962900
C 4.51390200 -1.00710000 -0.06377600
C -5.65301200 -1.41950100 0.04690600
C -6.99436300 -1.87144300 -0.00980300
C 7.00272000 -1.86642000 -0.03161500
C 5.66010300 -1.41545900 -0.04680800
C 8.07696900 -0.95055700 -0.03426700
C 9.37092800 -1.41688200 -0.01995800
N 9.64179200 -2.74199600 -0.00327300
C -7.30510000 -3.24516000 -0.05087800
C -8.62211900 -3.64710300 -0.10482000
N -9.63034700 -2.74940000 -0.11958600
C 8.63441800 -3.64081300 -0.00024000
C 7.31602500 -3.24012000 -0.01390700
C -9.36188300 -1.42428000 -0.08116400
C -8.06936800 -0.95675000 -0.02652200
H -3.55529500 1.59521400 0.22765600
H 3.55968800 1.60097300 -0.13581500
H -1.25018700 -0.30435600 2.59328600
H 0.17943500 -1.32268000 2.27853700
H 0.33610300 0.45540400 2.31915300
H -0.17954400 -1.27290200 -2.17097000
H -0.33446900 0.50597200 -2.17482000
H 1.25059800 -0.24948700 -2.46572400
H 7.89537800 0.12399300 -0.04752600
H 10.23132300 -0.74569400 -0.02146900
C 11.04666900 -3.18393000 0.01028000
H -6.51690500 -3.99774800 -0.04020000
H -8.90374100 -4.70024500 -0.13748500
C -11.03379900 -3.19276000 -0.17470200
H 8.91792500 -4.69386600 0.01339800
H 6.52864400 -3.99362400 -0.01121700
H -10.22290900 -0.75405900 -0.09578200
H -7.88964100 0.11779000 0.00320200
H 11.08238100 -4.27778500 0.03005400
H 11.55205200 -2.82006700 -0.89327100
H 11.54468700 -2.78806400 0.90435100
H -11.51167500 -2.77905300 -1.07163600
H -11.06709200 -4.28600500 -0.21818100
H -11.56056700 -2.84836900 0.72430100

Geometry of **Dy-yA** (open):

C 2.22088900 2.06015000 -0.12750300
C 0.98577400 2.24636200 0.38340200
C 0.72679000 3.71786000 0.63147400
C 1.87380900 4.44157400 -0.12108600
C 2.97756700 3.35991200 -0.22088000
F 3.69017600 3.47147000 -1.35105600
F 3.84795700 3.48957300 0.81134700
F 2.28654100 5.54200800 0.50046200
F 1.46500800 4.75686300 -1.35585800
F -0.47280000 4.12201900 0.18721500
F 0.79345900 3.99256200 1.94576900
C 2.88197900 0.81272100 -0.52884700
C 0.00736800 1.23234700 0.77968000
C 4.22986200 0.53390400 -0.19578200
C 4.66431600 -0.69110100 -0.66854100
S 3.39563200 -1.49410300 -1.54753400
C 2.29943800 -0.20112400 -1.28289700
C 0.31879800 0.14515500 1.57396000
S -1.06556200 -0.85875600 1.79271400
C -2.07824900 0.17541400 0.82352300
C -1.36323100 1.25663300 0.36994500
C 0.92258100 -0.26597100 -1.85477200
C 1.61666300 -0.20068300 2.23388500
C 5.93415300 -1.25805500 -0.51523600
C -3.43847600 -0.14492800 0.60386200
C 7.04543400 -1.75051900 -0.38589800
C -4.60876500 -0.42435600 0.42256400
C 8.32896800 -2.29886300 -0.24353600
C -5.98294100 -0.74033800 0.20884000
C 9.35477500 -1.60757400 0.44349300
C 10.60232500 -2.17071100 0.56194700
N 10.88386500 -3.38424800 0.03478200
C 9.92255800 -4.07151600 -0.62834500
C 8.65606800 -3.56755700 -0.78337100
C 12.22550400 -3.96956300 0.16039600
C -6.81228600 0.11405800 -0.53524300
C -8.15467300 -0.16549200 -0.73814800
C -8.71347200 -1.35760200 -0.20708600
C -7.88041400 -2.23573500 0.53481700
C -6.54850600 -1.91054100 0.73972100
C -9.04010600 0.77040100 -1.52131400
C -10.10851000 -0.02487400 -2.26088200
C -10.89792000 -0.86578200 -1.27237700
N -10.04131100 -1.65695600 -0.40781600
C -10.69412300 -2.78248900 0.23561700
C -9.93597900 -3.27428800 1.45669200
C -8.47339600 -3.50307300 1.09655100
H 4.86302200 1.20946500 0.37888700
H -1.80210700 2.03601600 -0.24996400
H 0.54858900 0.74703200 -2.05363900
H 0.90247600 -0.84007000 -2.79092500
H 0.22633600 -0.73807000 -1.14361600
H 2.24056300 0.69733800 2.34073100
H 1.45272200 -0.61995600 3.23677400
H 2.18675900 -0.94124600 1.64900800
H 9.16690600 -0.62765300 0.88145500
H 11.41583900 -1.66521700 1.08350300
H 10.21378500 -5.04335400 -1.02980400
H 7.90987800 -4.14988400 -1.32329300
H 12.85292700 -3.31091500 0.76958900
H 12.15504200 -4.95034200 0.64747700
H 12.67551900 -4.07853200 -0.83491400
H -6.39258000 1.03030800 -0.95776600
H -5.92233300 -2.58800600 1.32559500
H -8.43561700 1.36677400 -2.22034900
H -9.52770300 1.48747500 -0.83679100
H -9.63024900 -0.68095300 -3.00651600
H -10.79834800 0.63796800 -2.80298200
H -11.55268200 -0.21287800 -0.66153400
H -11.56529800 -1.55472500 -1.81761200
H -10.81978500 -3.61169300 -0.48885500
H -11.71014400 -2.46811200 0.52919100
H -10.40691800 -4.19632100 1.82712200
H -10.00426000 -2.52483700 2.26207000
H -8.40461600 -4.31444600 0.34974500
H -7.89723900 -3.83398200 1.97309400

Geometry of **Dy-yA** (closed):

C 3.05709500 3.66610300 -0.11784200
C 2.33812100 2.33972500 -0.10028100
C 0.93961200 2.54935600 0.00410200
C 0.62150900 4.00945500 -0.07639500
C 1.96577100 4.67861600 0.33750800
F 2.13474400 5.88402700 -0.19494200
F -0.38420400 4.39275900 0.72668300
F 2.00313600 4.77475400 1.67412600
F 4.13152500 3.69976800 0.68892300
F 3.47221500 3.97419000 -1.36039000
F 0.30249600 4.39931200 -1.33075700
C 2.88335600 1.08808000 -0.22250200
C 1.91749800 -0.07864300 -0.45163700
C 0.69850400 0.12386600 0.47224900
C 0.07216300 1.48475600 0.16534100
C 4.23982400 0.70437700 -0.19849100
C 4.42974500 -0.66022400 -0.14061200
S -0.66232200 -1.09578600 0.20266200
C -1.86106700 0.17731000 0.15649200
S 2.91476600 -1.59101100 -0.04781100
C -1.32870200 1.46072900 0.14741700
C 1.54474800 -0.12033200 -1.94527800
C 1.05028000 0.10390000 1.97546000
C 5.66975000 -1.28746900 -0.11442000
C -3.21446700 -0.15413000 0.11505900
C 6.75679600 -1.85695500 -0.08438400
C 7.99360300 -2.49889200 -0.05491300
C -5.76692400 -0.79480500 0.03785700
C -4.40049700 -0.45511500 0.07787900
C 8.09486900 -3.91679700 -0.00446400
C 9.32471800 -4.51697800 0.02240100
N 10.47368900 -3.79305400 0.00202600
C 10.41222600 -2.43951900 -0.04513000
C 9.21457100 -1.77337000 -0.07446700
C 11.76232400 -4.49061000 0.04794600
C -6.17758400 -2.14371800 0.01560600
C -7.51130000 -2.49580600 -0.03398300
C -8.50915800 -1.47640400 -0.03698500
C -8.10567200 -0.10859800 -0.00289500
C -6.76163600 0.20485600 0.02090300
C -7.94218000 -3.93950800 -0.07997600
C -9.27169000 -4.10528900 0.64415700
C -10.88741100 -0.81168300 -0.21217700
C -10.30545300 -3.17855100 0.03031600
N -9.83333100 -1.80564300 -0.07185700
C -10.37908900 0.49822400 -0.78650800
C -9.16331800 0.96537000 0.00285000
H 5.06625800 1.41571300 -0.19021100
H -1.95227800 2.35015100 0.07421500
H 2.45970800 -0.20375000 -2.54676000
H 0.89859600 -0.97857000 -2.17111400
H 1.02111400 0.80065200 -2.24261100
H 1.44673900 -0.87568900 2.27027600
H 1.80560700 0.87005100 2.20201100
H 0.15029400 0.31847800 2.56692100
H 7.19674300 -4.53357300 0.01184900
H 9.44007300 -5.60117800 0.06117000
H 11.36585200 -1.91065100 -0.05890600
H 9.20990100 -0.68453600 -0.11353700
H 12.57309000 -3.76344400 -0.06575500
H 11.87405400 -5.00644200 1.01099900
H 11.81929000 -5.21885500 -0.77122100
H -5.41977700 -2.93070700 0.02958600
H -6.46056000 1.25502500 0.03644200
H -7.16600900 -4.57951300 0.36378200
H -8.05515900 -4.26283000 -1.13010300
H -9.14493400 -3.86520700 1.71235600
H -9.63479600 -5.14100700 0.58304100
H -11.36483300 -0.63685500 0.77102900
H -11.66483200 -1.23500000 -0.86910300
H -10.59823900 -3.54524400 -0.97220900
H -11.22096000 -3.17571400 0.64430600
H -11.18690900 1.24298000 -0.75538700
H -10.10354300 0.35821300 -1.84443900
H -9.46505600 1.18710700 1.04196300
H -8.75588400 1.89877400 -0.41138500

Geometry of **Dy-A** (open)

C 2.95826400 1.58825600 -0.11412700

C 1.74624700 1.90463800 0.38744300

C 1.63636700 3.39909000 0.61358900

C 2.85684000 3.99256100 -0.13923200

C 3.84393100 2.80175800 -0.21968800

F 4.57090900 2.82325300 -1.34630100

F 4.72055900 2.85251000 0.81605200

F 3.37775200 5.05036400 0.47349800

F 2.49090900 4.33247700 -1.38029800

F 0.48742000 3.91446300 0.15409500

F 1.72305900 3.68164400 1.92435400

C 3.49196600 0.27508700 -0.49418800

C 0.67142600 0.99824300 0.79180300

C 4.80644600 -0.12840100 -0.15905700

C 5.13156600 -1.39519900 -0.60660600

S 3.78740400 -2.08303300 -1.46860500

C 2.81445300 -0.69039900 -1.23100100

C 0.87271000 -0.10201800 1.60383400

S -0.60271300 -0.96548900 1.82544800

C -1.50590100 0.14673900 0.83387700

C -0.68788500 1.14875600 0.37158100

C 1.43906500 -0.63765700 -1.80457000

C 2.12682000 -0.55765100 2.28186200

C 6.37773900 -2.09552600 -0.43554700

C -2.88774600 -0.04892000 0.60708300

C -4.07752200 -0.22425400 0.42055100

C -5.47240400 -0.42102600 0.20265900

C -6.20360200 0.44518800 -0.62628600

C -7.56387400 0.28110600 -0.83436900

C -8.24436100 -0.80310500 -0.21934500

C -7.51144800 -1.69349400 0.60939000

C -6.15719600 -1.48306300 0.81510600

C -8.34291000 1.22981900 -1.70977900

C -9.47475500 0.48291900 -2.40401900

C -10.35546900 -0.19198300 -1.36670900

N -9.59163700 -0.98773100 -0.42260000

C -10.36099300 -1.98660900 0.29747100

C -9.67082700 -2.44603900 1.57021000

C -8.23378000 -2.84583100 1.26020000

H 5.48080500 0.51419800 0.40703200

H -1.04634600 1.95829100 -0.26131500

H 1.16164600 0.40237100 -2.02097200

H 1.36534000 -1.22474600 -2.72981700

H 0.70238000 -1.02915100 -1.08503000

H 2.83379800 0.27821300 2.37578100

H 1.91653800 -0.93677800 3.29206000

H 2.62604600 -1.36337300 1.71830800

C 6.57875600 -3.41246800 -0.91012800

C 7.78480900 -4.04564900 -0.73320800

N 8.81537100 -3.44013800 -0.10296100

H -5.69035100 1.27815000 -1.11305900

H -5.60846900 -2.16675900 1.46760200

H -7.67384900 1.70455200 -2.44237100

H -8.76643400 2.04513300 -1.09632500

H -9.05402900 -0.27625000 -3.08356100

H -10.08910400 1.16205300 -3.01259600

H -10.95029200 0.56969400 -0.82461100

H -11.08032700 -0.85626700 -1.86713000

H -10.55871200 -2.85690200 -0.35937500

H -11.34436700 -1.55076300 0.54263400

H -10.23528100 -3.28349000 2.00506200

H -9.67474300 -1.62888800 2.30992300

H -8.23639000 -3.71918300 0.58345900

H -7.70412400 -3.15743000 2.17238900

C 8.66065400 -2.17866900 0.36702300

C 7.48141600 -1.49567700 0.21996700

H 5.78485900 -3.95487400 -1.42529600

H 7.96033000 -5.06021400 -1.09253000

C 10.10468400 -4.12116900 0.07256000

H 9.52612800 -1.73902700 0.86501800

H 7.42148200 -0.48267300 0.61539700

H 10.03156300 -5.14165200 -0.31729500

H 10.88635200 -3.57995500 -0.47617000

H 10.35793800 -4.16137900 1.13953100

Geometry of **Dy-A** (closed):

C -3.94454700 3.01982300 0.13925500

C -3.08987200 1.77656100 0.09121000

C -1.73377000 2.12999400 -0.03066400

C -1.56053700 3.61429300 0.04406600

C -2.97671000 4.14587900 -0.32833000

F -3.25556900 5.31961900 0.22671700

F -0.62250100 4.09191100 -0.78981200

F -3.05876600 4.25341200 -1.66153500

F -5.03442700 2.94424200 -0.64448700

F -4.36231700 3.26742600 1.39345600

F -1.24044700 4.03460900 1.28773000

C -3.50490100 0.46663600 0.20726900

C -2.42195100 -0.59288500 0.42361500

C -1.23923200 -0.25951500 -0.51138900

C -0.75346500 1.15358500 -0.19368700

C -4.80696700 -0.04882700 0.18243200

C -4.87859000 -1.42944500 0.12718300

S 0.24267500 -1.33445900 -0.26862600

C 1.30189300 0.04899800 -0.18983100

S -3.26897600 -2.18781000 0.01319100

C 0.63162300 1.27685300 -0.16115200

C -2.02768000 -0.60628200 1.91155800

C -1.60394600 -0.30295300 -2.01086600

C -6.06656400 -2.20891800 0.11268600

C 2.67696000 -0.12996100 -0.14203400

C 5.28359600 -0.46357600 -0.04757800

C 3.89256000 -0.29023900 -0.09739100

C 5.85578100 -1.75517200 -0.05270500

C 7.21970400 -1.94400600 0.00735900

C 8.08693400 -0.80900200 0.05166000

C 7.52011500 0.50277600 0.04518600

C 6.15041800 0.65073300 0.00847100

C 7.82319000 -3.32500400 0.02488900

C 9.17259400 -3.30822100 -0.68065900

C 10.36234800 0.13534000 0.28099700

C 10.07569900 -2.27855400 -0.02701300

N 9.43694600 -0.97533800 0.09935500

C 9.69257900 1.35895100 0.87867800

C 8.43998600 1.69611500 0.08115800

H -5.68110300 0.60321600 0.17278200

H 1.15954400 2.22425400 -0.06510800

H -2.92154700 -0.78525800 2.52355800

H -1.29573100 -1.39667700 2.12356800

H -1.59569000 0.36067600 2.21065800

H -1.90337800 -1.31563100 -2.30855400

H -2.43660200 0.38339300 -2.22112100

H -0.73846000 0.00664000 -2.61158800

C -6.02772900 -3.63138100 0.06303500

C -7.18234200 -4.36659500 0.04076600

N -8.40386400 -3.77957600 0.06429100

H 5.19891700 -2.62710000 -0.09691800

H 5.72319300 1.65624900 0.01382900

H 7.13702000 -4.04271500 -0.44708500

H 7.96024300 -3.65911400 1.06866500

H 9.03287500 -3.05843300 -1.74495700

H 9.65912800 -4.29280800 -0.63765200

H 10.82650200 0.38946000 -0.69069800

H 11.17592300 -0.21032200 0.93904300

H 10.39558000 -2.62962300 0.97234400

H 10.99266700 -2.14618000 -0.62372500

H 10.40493800 2.19588200 0.87773700

H 9.42294800 1.16005800 1.92862200

H 8.72581600 1.97782800 -0.94778500

H 7.91719000 2.56284500 0.51013400

C -8.48499500 -2.42137300 0.11491200

C -7.37095900 -1.63265100 0.14122700

H -5.07522600 -4.16204200 0.04990700

H -7.16909600 -5.45649000 0.00420700

C -9.63293500 -4.57330500 0.03458000

H -9.49161300 -2.00147800 0.13375500

H -7.51128000 -0.55354600 0.18416700

H -9.37739300 -5.63806300 0.01458000

H -10.23360500 -4.36799400 0.93063000

H -10.21602600 -4.32934700 -0.86356300

Geometry of **D-yA** (open):

C -1.23898300 1.99383700 0.11316300
C 0.02082300 2.06039100 -0.36717600
C 0.41166400 3.49812300 -0.63996400
C -0.69052000 4.33536900 0.06040700
C -1.88472000 3.35405400 0.15290000
F -2.61637100 3.55567100 1.25864800
F -2.71258500 3.53067100 -0.90715200
F -0.99047800 5.45052000 -0.59888100
F -0.29092500 4.64764100 1.29924900
F 1.62771800 3.81188500 -0.16680200
F 0.40782100 3.74543500 -1.96129000
C -2.01232800 0.81749500 0.52833800
C 0.91568200 0.95464400 -0.70904700
C -3.37024900 0.64213000 0.16782900
C -3.91699800 -0.52943300 0.65995500
S -2.74076100 -1.41059500 1.59115000
C -1.53478100 -0.22046300 1.32250200
C 0.52860800 -0.12623500 -1.47495100
S 1.82929700 -1.25195500 -1.61413500
C 2.91392100 -0.27206500 -0.66963800
C 2.27878500 0.87558800 -0.27312300
C -0.18147400 -0.38480200 1.92941300
C -0.77597200 -0.38480300 -2.16104800
C -5.22520200 -0.99273400 0.48600400
C 4.28550600 -0.70947500 -0.38768900
C -6.37054100 -1.39345000 0.33694600
C -7.69141900 -1.83443800 0.16918500
C -8.14275400 -3.05499200 0.72496200
C -9.44515100 -3.45256900 0.54286600
N -10.32236700 -2.70231600 -0.16241300
C -9.92046600 -1.52864600 -0.70786000
C -8.63483900 -1.07250800 -0.56453900
C -11.71332300 -3.13792900 -0.34554400
C 4.97608600 -1.57991000 -1.24042800
C 6.27527300 -1.99478400 -0.98149800
C 6.95312600 -1.50696600 0.16394200
C 6.27060300 -0.61654300 1.03135600
C 4.96094100 -0.25255000 0.75168900
C 6.99618200 -2.93965300 -1.90918000
C 8.48650200 -2.62234000 -1.91679300
C 8.93270600 -1.52569700 1.65604400
C 9.03254400 -2.68283800 -0.50028600
N 8.24842000 -1.89231700 0.43035900
C 7.96821700 -1.14202600 2.76550900
C 6.98379400 -0.09745700 2.25421200
H -3.93109600 1.35146200 -0.44028400
H 2.76747000 1.65637000 0.30738500
H 0.26959100 0.59874900 2.11601300
H -0.23027700 -0.93784000 2.87715800
H 0.48959500 -0.92659600 1.24404300
H -1.42243400 -1.05622100 -1.57178100
H -1.32015200 0.55756500 -2.31327800
H -0.62119400 -0.84812900 -3.14590700
H -7.46654600 -3.68699900 1.29997400
H -9.82617100 -4.38635200 0.95782800
H -10.67564400 -0.97094700 -1.26387600
H -8.34921500 -0.12321700 -1.01699300
H -11.85642800 -4.10833500 0.14068200
H -12.39247000 -2.40397800 0.10706500
H -11.93070900 -3.23640300 -1.41676500
H 4.49235200 -1.94435000 -2.15201900
H 4.44761300 0.40892200 1.45498800
H 6.57269100 -2.87255100 -2.92216300
H 6.84743400 -3.98210300 -1.57447400
H 8.64607000 -1.61405900 -2.33252800
H 9.04316700 -3.32873900 -2.54938900
H 9.63643100 -0.69107400 1.46452300
H 9.54692000 -2.38415600 1.97695800
H 9.07825900 -3.73652400 -0.15940700
H 10.06893400 -2.30483200 -0.48476100
H 8.53888700 -0.76631600 3.62707500
H 7.41688000 -2.03554600 3.10156800
H 7.53255000 0.82807000 2.00363900
H 6.25446300 0.17301200 3.03217100

Geometry of **D-yA** (closed):

```
C 1.85464900 3.67855900 -0.12692300
C 1.28089600 2.28330100 -0.08776900
C -0.12246900 2.34404400 0.02242200
C -0.60267800 3.75673600 -0.06128800
C 0.66452400 4.57625300 0.32242800
F 0.69537200 5.78095000 -0.23770900
F -1.63137100 4.03090300 0.76035700
F 0.70486100 4.70688900 1.65580000
F 2.92290200 3.83743500 0.67268400
F 2.22825400 4.01140000 -1.37605000
F -0.99334900 4.09954700 -1.31003900
C 1.96135300 1.09094200 -0.20326000
C 1.12349700 -0.17184800 -0.43022900
C -0.10935500 -0.09427700 0.49614300
C -0.87673800 1.18478800 0.17883100
C 3.34382900 0.85666000 -0.17389600
C 3.68708500 -0.48363200 -0.12099000
S -1.33707200 -1.44364500 0.23473000
C -2.67334100 -0.32135600 0.15462900
S 2.27443800 -1.56859500 -0.02518200
C -2.25625100 1.01291300 0.13763300
C 0.74964800 -0.25453600 -1.92151600
C 0.24672300 -0.07077900 1.99761300
C 4.98344700 -0.96671400 -0.09978700
C -4.02581900 -0.79689900 0.09287600
C 6.13216300 -1.40724400 -0.07469300
C 7.43238300 -1.89496900 -0.05312800
C 7.70765100 -3.29250800 -0.01029100
C 8.99994800 -3.73905600 0.00760800
N 10.05509000 -2.88226000 -0.01504400
C 9.82855000 -1.54445900 -0.05403500
C 8.56100800 -1.02775200 -0.07451900
C 11.41822200 -3.41523900 0.03955700
C -5.11699600 0.10121600 0.06506600
C -6.42477900 -0.32698600 0.01532800
C -6.70630000 -1.72694800 -0.03729800
C -5.61757300 -2.65168800 -0.02129600
C -4.32710900 -2.17611700 0.05467100
C -7.57414700 0.64788500 0.01435200
C -8.72937500 0.08335500 -0.80167900
C -8.33725300 -3.58431100 -0.02670400
C -9.12751200 -1.27352100 -0.25071400
N -7.98902500 -2.17122400 -0.10090400
C -7.23860500 -4.42140800 0.60241300
C -5.91283800 -4.12804600 -0.08733300
H 4.08896500 1.65286600 -0.16027400
H -2.94402400 1.85033400 0.03885200
H 1.66579800 -0.24676900 -2.52672400
H 0.19359600 -1.17523000 -2.14214800
H 0.13389700 0.60743900 -2.21989200
H 0.74341600 -1.00315600 2.29360100
H 0.92246500 0.76831100 2.21622200
H -0.66784000 0.05444200 2.59233700
H 6.89128600 -4.01393700 0.00612600
H 9.24480000 -4.80168200 0.04040900
H 10.71138900 -0.90435000 -0.06915900
H 8.42550300 0.05274300 -0.10893300
H 12.13095500 -2.61457800 -0.18517400
H 11.62792700 -3.81418000 1.04156800
H 11.53484000 -4.21161300 -0.70623600
H -4.93764200 1.17752600 0.09650500
H -3.51457700 -2.90812400 0.07627100
H -7.24585000 1.61944200 -0.38170900
H -7.91126000 0.82751700 1.05060400
H -8.42574000 -0.01781300 -1.85628000
H -9.60126200 0.75187400 -0.77616100
H -8.57072000 -3.95817400 -1.04142000
H -9.26395900 -3.67029700 0.56310000
H -9.63317900 -1.15725900 0.72631400
H -9.85049800 -1.75942300 -0.92547500
H -7.50511000 -5.48453000 0.52115700
H -7.15987600 -4.18511000 1.67600100
H -5.97000500 -4.44991200 -1.14231300
H -5.09223700 -4.69839400 0.37098600
```

S20. References

1. K. Stranius and K. Borjesson, *Sci. Rep.*, 2017, **7**, 41145.
2. S. Hermes, G. Dassa, G. Toso, A. Bianco, C. Bertarelli and G. Zerbi, *Tetrahedron Lett.*, 2009, **50**, 1614–1617.
3. E. Zysman-Colman, K. Arias and J. S. Siegel, *Can. J. Chem.*, 2009, **87**, 440–447.
4. R. Saito, *Heterocycles*, 2010, **81**, 2831–2840.
5. A. Osuka, D. Fujikane, H. Shinmori, S. Kobatake and M. Irie, *J. Org. Chem.*, 2001, **66**, 3913–3923.
6. T. C. Pace, V. Muller, S. Li, P. Lincoln and J. Andreasson, *Angew. Chem. Int. Ed.*, 2013, **52**, 4393–4396.
7. E. Stadler, A. Eibel, D. Fast, H. Freissmuth, C. Holly, M. Wiech, N. Moszner and G. Gescheidt, *Photochem. Photobiol. Sci.*, 2018, **17**, 660–669.
8. C. Ozcoban, T. Halbritter, S. Steinwand, L. M. Herzig, J. Kohl-Landgraf, N. Askari, F. Groher, B. Furtig, C. Richter, H. Schwalbe, B. Suess, J. Wachtveitl and A. Heckel, *Org. Lett.*, 2015, **17**, 1517–1520.
9. B. Maerz, S. Wiedbrauk, S. Oesterling, E. Samoylova, A. Nenov, P. Mayer, R. de Vivie-Riedle, W. Zinth and H. Dube, *Chem. Eur. J.*, 2014, **20**, 13984–13992.
10. J. H. Blanch, *J. Chem. Soc. B*, 1966, 937–939.
11. M. Irie, T. Lifka, K. Uchida, S. Kobatake and Y. Shindo, *Chem. Commun.*, 1999, 747–748.
12. M. Herder, B. M. Schmidt, L. Grubert, M. Patzel, J. Schwarz and S. Hecht, *J. Am. Chem. Soc.*, 2015, **137**, 2738–2747.
13. D. Mendive-Tapia, A. Perrier, M. J. Bearpark, M. A. Robb, B. Lasorne and D. Jacquemin, *Phys. Chem. Chem. Phys.*, 2014, **16**, 18463–18471.
14. G. Pariani, M. Quintavalla, L. Colella, L. Oggioni, R. Castagna, F. Ortica, C. Bertarelli and A. Bianco, *J. Phys. Chem. C*, 2017, **121**, 23592–23598.
15. M. J. Kamlet, J. L. M. Abboud, M. H. Abraham and R. W. Taft, *J. Org. Chem.*, 1983, **48**, 2877–2887.
16. K. Ponnusamy, S. Chellappan, C. M. Singaravelu and J. Kandasamy, *J. Luminescence*, 2018, **202**, 253–262.
17. L. G. Nandi, F. Facin, V. G. Marini, L. M. Zimmermann, L. A. Giusti, R. da Silva, G. F. Caramori and V. G. Machado, *J. Org. Chem.*, 2012, **77**, 10668–10679.
18. K. Okuno, Y. Shigeta, R. Kishi, H. Miyasaka and M. Nakano, *J. Photochem. Photobiol. A: Chem.*, 2012, **235**, 29–34.
19. K. Matsuda, Y. Shinkai, T. Yamaguchi, K. Nomiyama, M. Isayama and M. Irie, *Chem. Lett.*, 2003, **32**, 1178–1179.
20. R. Castagna, V. Nardone, G. Pariani, E. Parisini and A. Bianco, *J. Photochem. Photobiol. A: Chem.*, 2016, **325**, 45–54.
21. M. Morimoto, S. Kobatake and M. Irie, *Chem. Eur. J.*, 2003, **9**, 621–627.
22. L. N. Lucas, J. van Esch, R. M. Kellogg and B. L. Feringa, *Tetrahedron Lett.*, 1999, **40**, 1775–1778.
23. M. Han, Y. Luo, B. Damaschke, L. Gómez, X. Ribas, A. Jose, P. Peretzki, M. Seibt and G. H. Clever, *Angew. Chem. Int. Ed.*, 2016, **55**, 445–449.
24. U. Al-Atar, R. Fernandes, B. Johnsen, D. Baillie and N. R. Branda, *J. Am. Chem. Soc.*, 2009, **131**, 15966–15967.
25. S. L. Gilat, S. H. Kawai and J.-M. Lehn, *J. Chem. Soc. Chem. Comm.*, 1993, 1439–1442.
26. S. L. Gilat, S. H. Kawai and J.-M. Lehn, *Chem. Eur. J.*, 1995, **1**, 275–284.
27. Y. M. Hervault, C. M. Ndiaye, L. Norel, C. Lagrost and S. Rigaut, *Org. Lett.*, 2012, **14**, 4454–4457.
28. P. Pracht, F. Bohle and S. Grimme, *Phys. Chem. Chem. Phys.*, 2020, **22**, 7169–7192.

29. H. J. Kuhn, S. E. Braslavsky, R. Schmidt, *Chem. Eur. J.*, 1995, **1**, 187–210.
30. M. Montalti, A. Credi, L. Prodi, M. Teresa Gandolfi, *Handbook of Photochemistry*, CRC Press, **2006**.
